# General Alkene 1,2-*syn*-Cyano-Hydroxylation Procedure Via Electrochemical Activation of Isoxazoline Cycloadducts

**DOI:** 10.1021/jacs.4c13682

**Published:** 2024-11-13

**Authors:** Taciano A. S. Wanderley, Roberto Buscemi, Órla Conboy, Benjamin Knight, Giacomo E. M. Crisenza

**Affiliations:** Department of Chemistry, The University of Manchester, Oxford Road, Manchester M13 9PL, U.K.

## Abstract

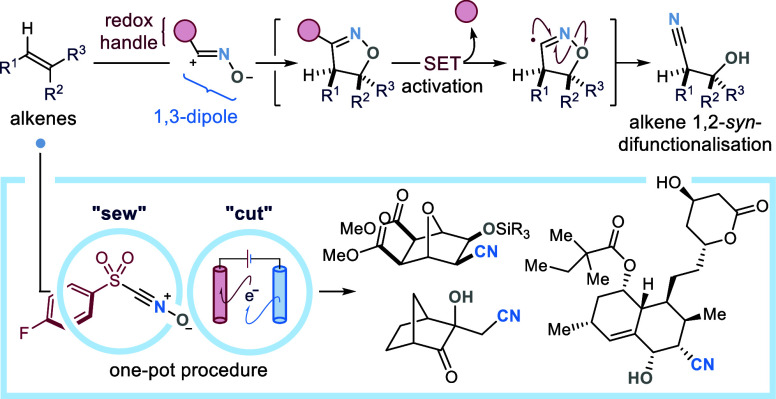

Stereoselective alkene 1,2-difunctionalization is a privileged strategy to access three-dimensional C(sp^3^)-rich chiral molecules from readily available “flat” carbon feedstocks. State-of-the-art approaches exploit chiral transition metal-catalysts to enable high levels of regio- and stereocontrol. However, this is often achieved at the expense of a limited alkene scope and reduced generality. 1,3-Dipolar cycloadditions are routinely used to form heterocycles from alkenes with high levels of regioselectivity and stereospecificity. Nevertheless, methods for the ring-opening of cycloadducts to reveal synthetically useful functionalities require the use of hazardous reagents or forcing reaction conditions; thus limiting their synthetic applications. Herein, we describe the implementation of a practical, general and selective electrosynthetic strategy for olefin 1,2-*syn*-difunctionalization, which hinges on the design of novel reagents–consisting of a nitrile oxide 1,3-dipole precursor, equipped with a sulfonyl-handle. These can selectively difunctionalize alkenes via “click” 1,3-dipolar cycloadditions, and then facilitate the telescoped electrochemical single electron transfer activation of the ensuing isoxazoline intermediate. Cathodic reduction of the cycloadduct triggers a radical fragmentation pathway delivering sought-after stereodefined 1,2-*syn*-hydroxy nitrile derivatives. Our telescoped electrochemical procedure tolerates a wide range of functionalities, and—crucially—enables the difunctionalization of both electron-rich, electron-poor and unactivated olefins, with diverse degree of substitution; thus providing a robust, general and selective metal-free alternative to current alkene difunctionalization strategies. Capitalizing on these features, we employed our electrosynthetic method to enable the late-stage *syn*-hydroxy-cyanation of natural products and bioactive compounds, and streamline the *de novo* synthesis of pharmaceutical agents.

## Introduction

The identification and development of increasingly complex three-dimensional drugs and agrochemicals demand ready access to ever-growing libraries of stereodefined molecular fragments.^1^ Here, the simultaneous installation of multiple functionalities across alkenes’ C=C bonds stands as one of the swiftest ways to convert ubiquitous^2^ “flat” hydrocarbons into C(sp^3^)-rich chiral building blocks. This aspect keeps driving the investigation of innovative and general synthetic technologies for the stereoselective poly functionalization of olefins.^3^

**Scheme 1:**
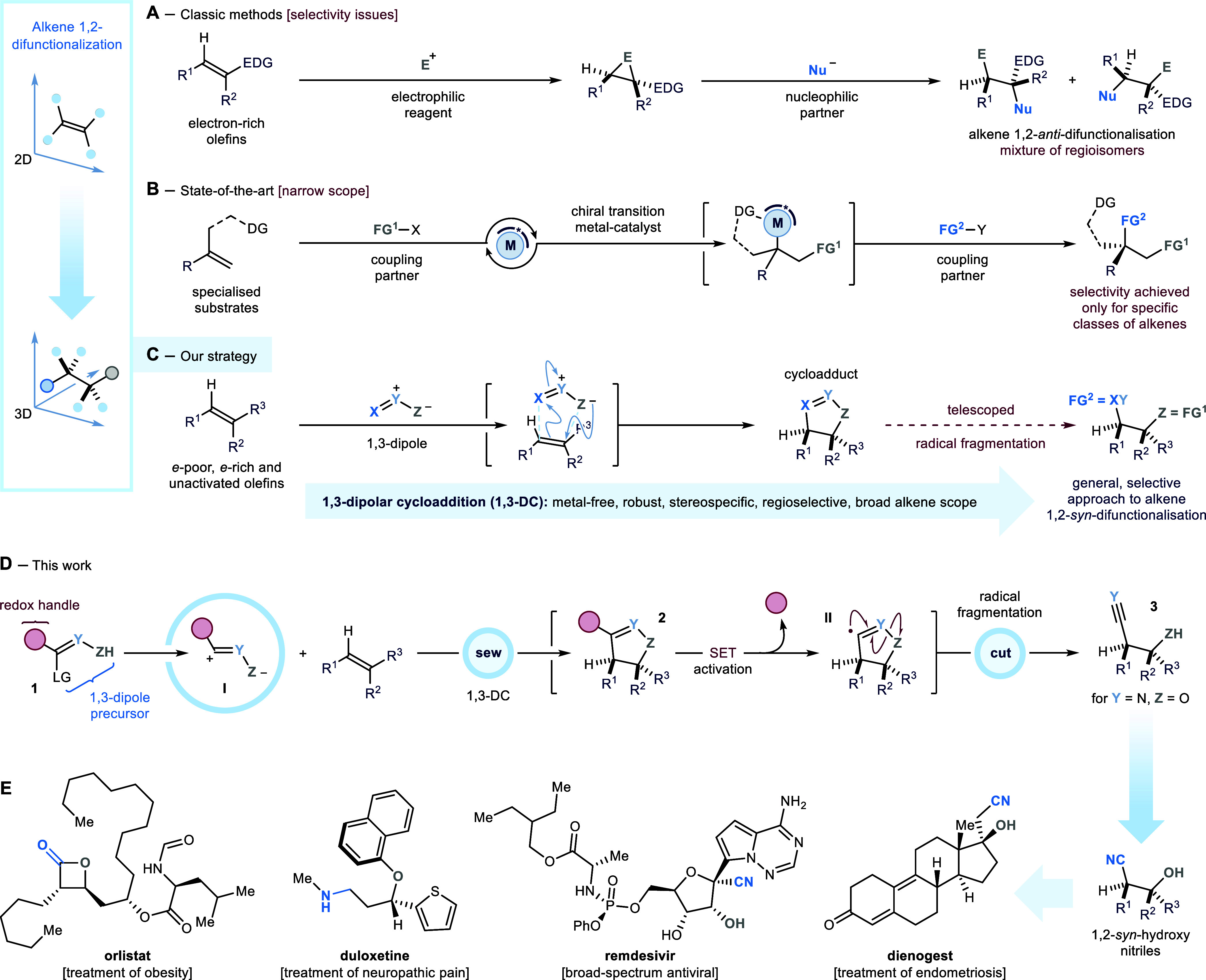
Strategies for Stereoselective Olefin 1,2-Difunctionalization: (A) Classic *anti*-Selective Methods. (B) State-of-the-Art Transition Metal-Catalyzed Protocols. (C) Our Design: Implementation of Telescoped 1,3-DC/Ring-Opening Procedures to Enable General Alkene 1,2-*syn*-Difunctionalization Reactions. (D) This Work: Development of a Radical-Mediated “Sew & Cut” Approach to the 1,2-*syn*-Cyano-Hydroxylation of Alkenes. (E) Pharmaceutical Agents Containing 1,2-Hydroxy Nitriles, or Accessible through Their Downstream Manipulation API, active pharmaceutical ingredient; 1,3-DC, 1,3-dipolar cycloaddition; DG, directing group; FG, functional group; LG, leaving group; SET, single electron transfer.

For alkene 1,2-difunctionalization, classic approaches proceed through the formation of electrophilic three-membered heterocycles (e.g., halonium ions, epoxides, aziridines),^4^ which undergo S_N_2-ring-opening reactions with nucleophiles to produce alkene 1,2-*anti*-difunctionalized products–often as mixtures of regioisomers (Scheme 1A). Despite recent elegant examples,^5^ the generality of these methods is inherently hamstrung by the electronics of the alkene substrate (i.e., use of nucleophilic olefins), and limited to a handful of electrophilic partners. To expand the breadth of alkene difunctionalization reactions, state-of-the-art strategies use chiral transition metal catalysts to orchestrate the stereoselective addition of a broad range of functional groups across C=C bonds, using combinations of electrophilic and nucleophilic coupling partners (Scheme 1B).^6^ More recently, the scope of these strategies has been expanded to the use of radical precursors and intermediates, by capitalizing on the ability of metal complexes to trap and tame open-shell species^7^—usually in combination with photochemical^8^ or electrochemical settings.^9^ Despite these advances, these protocols often present a limited alkene scope, where high levels of regio- and stereoselectivity are achieved only for specific classes of substrates (e.g., either electron-rich or electron-poor olefins) with distinct substitution patterns (e.g., monosubstituted alkenes; use of styrenes leading to stabilized benzylic radical intermediates).^6−9^ On the other hand, for unactivated alkenes, specialized directing groups—covalently tailored to the substrates’ double bond—are usually required to direct the metal insertion regioselectively.^10^ Besides this, the more frequent use of transition metals raises concerns about their cost, toxicity, abundance and market availability; thus fostering—when convenient—the development of metal-free alternatives.

Seeking the development of a general, metal-free and stereoselective alkene difunctionalization strategy, we considered that one of the most powerful means to difunctionalize alkenes is their use as dipolarophiles in Huisgen 1,3-dipolar cycloadditions (1,3-DCs) to form heterocyclic cycloadducts (Scheme 1C).^11^ These reactions are fast and efficient–often proceeding in the absence of any catalyst–and their pericyclic character offers *stereospecificity* (selective *syn*-addition, as opposed to the *anti*-selectivity described in Scheme 1A); programmable *regioselectivity* (controlled by both steric and electronic factors); *robustness* (no need for inert atmosphere or anhydrous conditions); and, crucially, a *broad alkene scope* (including olefins of diverse electronics and degree of substitution). Such features have promoted the use of these transformations in diverse contexts, spanning from materials science^12^ to bio-orthogonal click chemistry (2022 Nobel Prize in Chemistry).^13^ To exploit the remarkable synthetic potential of 1,3-DCs beyond heterocycle formation, we wondered whether the stereodefined cycloadduct products could be then shaped into synthetically useful functionalities by means of telescoped radical-mediated ring-opening processes (Scheme 1C, *red arrow*).^14^ This approach would convert–in a single operation–a wide variety of olefins into the corresponding 1,2-*syn*-difunctionalized products; thus providing a robust, general, and stereoselective metal-free platform for alkene difunctionalization.

### Design Plan

To realize this strategy, we conceived the design of reagents of type **1** (Scheme 1D)—consisting of a 1,3-dipole precursor equipped with a redox-handle.^15^ Upon in situ activation, **1** can be converted into 1,3-dipole **I** and engage alkenes in stereospecific 1,3-DCs (*sew step*), delivering cycloadducts **2**. The presence of a redox-auxiliary within **2** is key to facilitating the single electron transfer (SET) activation of the cycloadduct, and deliver–upon extrusion of the redox-handle–open-shell intermediate **II**. Radical fragmentation of the heterocyclic core of **II** (*cut step*) reveals the desired synthetic functionalities within product **3**, with retention of the *syn*-stereochemistry. In this article, we demonstrate the successful implementation of this design plan. Building on groundbreaking contributions reported in the 1980s by De Sarlo,^16a^ Kozikowski^16b,16c^ and Wade^16d^—we identified the cycloaddition between olefins and nitrile oxides to produce isoxazolines (*Y*=N, *Z*=O in Scheme 1D) as a general, efficient and robust 1,3-DC process for our endeavors. This has brought about the development of novel sulfonyl-tailored nitrile oxide precursors of type **1**, and their use in telescoped electrosynthetic procedures with a variety of electron-rich, electron-poor and unactivated alkenes to furnish a diverse array of 1,2-*syn*-hydroxy-nitrile derivatives. Crucially, our method enables the swift, stereoselective installation of versatile CN and OH functionalities under mild aerobic conditions, and bypassing the use of transition metals and hazardous cyanide reagents.^17^ Furthermore, in stark contrast with previous applications of the “isoxazoline route”,^16^ our protocol leverages electrochemical activation to circumvent the use of highly energetic reagents to form reactive isoxazoline intermediates (e.g., trimethylsilanecarbonitrile from mercury fulminate or dibromoformaldoxime), and the employment of multistep, harsh experimental procedures to promote their fragmentation (e.g., pyrolysis at 200 °C, reduction with sodium amalgam). Thus, compared to existing methods, our electrosynthetic “*sew & cut*” approach offers a broader alkene scope (currently limited to unactivated alkenes and styrenes),^16^ a wider functional group tolerance, user-friendly conditions, and–crucially–enhanced synthetic applicability. The latter aspect is particularly important considering the ubiquity of stereodefined *syn*-β-hydroxy nitriles–and their derivatives (e.g., β-hydroxy acids, β-lactones, γ-amino alcohols)—in drug candidates and pharmaceutical agents (Scheme 1E).

## Results and Discussion

### Reagents Design

**Scheme 2:**
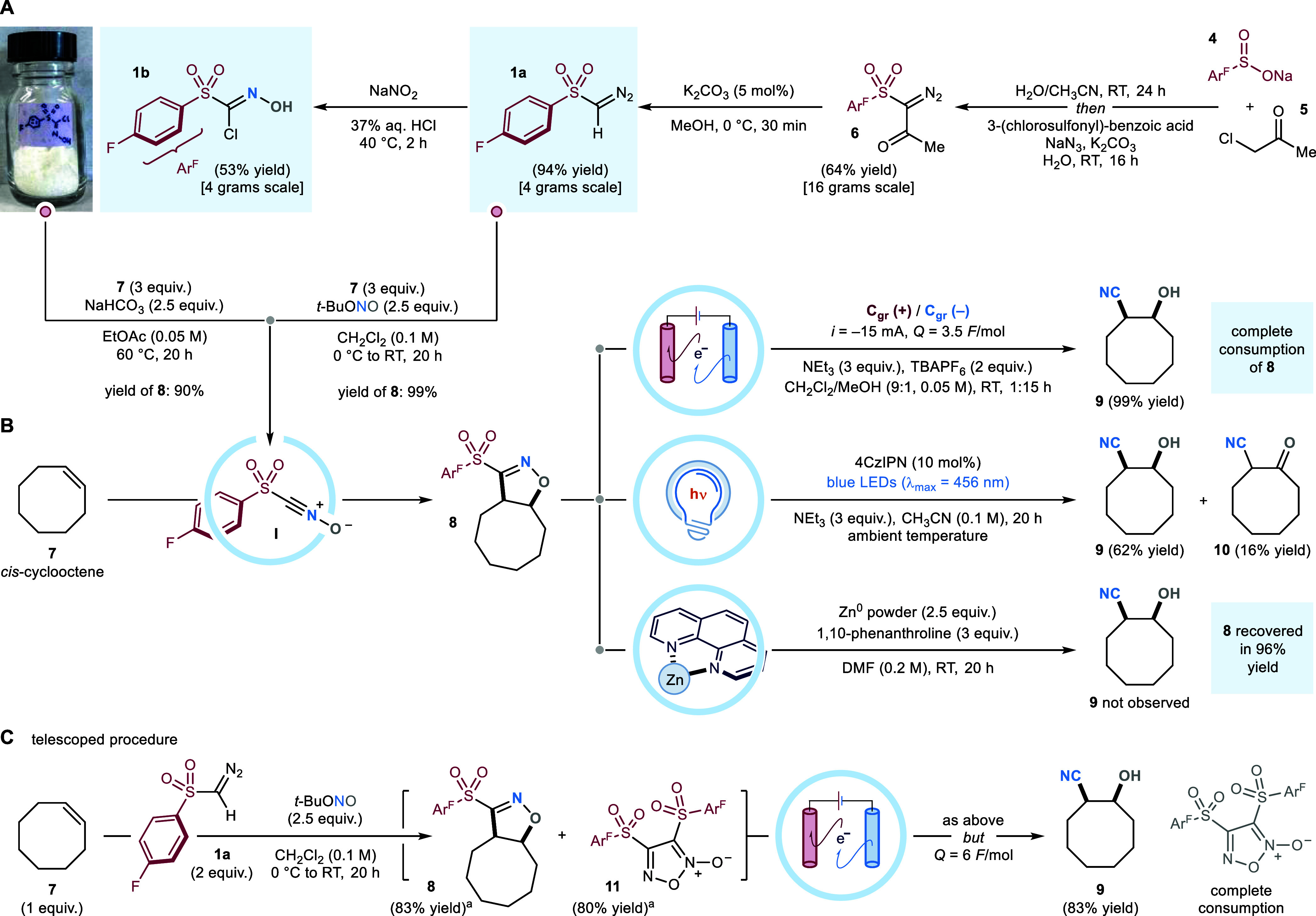
(A) Synthesis of Reagents **1a** and **1b**. (B) Optimized Conditions for the 1,3-DC Step, and Evaluation of a Suitable Reductive Radical Manifold for the Fragmentation of Cycloadduct 8. (C) Development of a Telescoped Electrochemical Alkene 1,2-*syn*-Cyano-Hydroxylation Procedure RT, room temperature; *C*_gr_, carbon graphite; *i*, current intensity; *Q*, quantity of charge; TBAPF_6_, tetrabutylammonium hexafluorophosphate; 4CzIPN, 1,2,3,5-tetrakis(carbazol-9-yl)-4,6-dicyanobenzene; LEDs, light-emitting diodes.

At the outset of our investigations, we sought to identify suitable nitrile oxide precursors **1** that generate inoffensive byproducts, thus enabling follow-up radical transformations in a single telescoped procedure. These endeavors have led to the development of 1-(diazomethylsulfonyl)-4-fluorobenzene **1a** and 1-(4-fluorophenyl-sulfonyl)-*N*-hydroxymethanimidoyl chloride **1b** (Scheme 2A). These compounds feature both a 1,3-dipole precursor (i.e., diazo-group^18^ for **1a**, and chloroxime^11^ for **1b**) and an aryl sulfone moiety; which is suitable for direct SET reduction,^19^ but also able to impart lower reduction potentials to the ensuing cycloadduct.^15,20^ Reagent **1a** was synthesized through a two-step procedure from cheap, commercially available starting materials. Specifically, the addition of sodium sulfinate **4** to chloroacetone **5**, followed by telescoped diazo-group transfer to the ensuing α-sulfonyl-ketone, produced intermediate **6**. This was swiftly converted into **1a** via base-assisted deacetylation. Reagent **1a** can be stored for up to 2 weeks at −20 °C (without degradation occurring), or turned into chloroxime **1b**, upon treatment with NaNO_2_ in aqueous HCl. Reagent **1b** is a stable, easy-to-handle, colorless solid–whose structure has been corroborated by X-ray crystallographic analysis. Of note, the synthesis of both reagents does not involve chromatographic purification procedures, and it can be performed in multigram scale, without loss of efficiency (see Supporting Information).

### Process Optimization

The ability of **1a** and **1b** to serve as competent reagents in the *sew* step was tested in a 1,3-DC with *cis*-cyclooctene **7** (Scheme 2B). Treatment with either *tert*-butyl nitrite–when using **1a**—or sodium bicarbonate–employing **1b**—converted the 1,3-dipole precursors into sulfonyl-nitrile oxide **I**. In both cases, **I** efficiently clicked onto the olefinic bond of **7** to provide cycloadduct **8** in excellent yield. Isoxazoline **8** was then isolated and submitted to different reductive conditions to promote its radical fragmentation via SET activation (*cut* step). Using constant current electrolysis (graphite electrodes, −15 mA, 3.5 F/mol) with a TBAPF_6_ electrolyte and sacrificial reductant NEt_3_, **8** was successfully converted to the desired 1,2-*syn*-cyano-hydroxylation product **9** in quantitative yield, as a single diastereomer. Product **9** was also obtained, under photoredox conditions, by exposing **8** to organic photocatalyst 4CzIPN under blue-light irradiation (λ_max_ centered at 456 nm). However, in this case, **9** was isolated in a reduced 62% yield alongside α-cyano-ketone **10** (16% yield)—derived from the overoxidation of alcohol **9**. Conversely, treatment of **8** with superstoichiometric amounts of highly reducing zinc(0)/phenanthroline complex^21^ did not deliver any product. Here, isoxazoline **8** was recovered quantitatively, even when performing the reaction at 60 °C (see Supporting Information). It is worth mentioning that the presence of a fluorine atom at the aryl moiety of reagents **1a**–**b** is not necessary to enable either step of the “*sew & cut*” protocol (i.e., the use of ((diazomethyl)sulfonyl)benzene **1c** converts **7** into **9** with analogous efficiency, see Supporting Information); but offers improved experimental ease and higher yields for the synthesis of **1a**, as well as provides a handle for monitoring 1,3-DC reactions by NMR.

Having identified efficient electrochemical conditions to promote the radical fragmentation of the cycloadduct, we implemented a telescoped procedure for the direct conversion of alkene **7** into 1,2-*syn*-hydroxy nitrile **9** (Scheme 2C). For these endeavors, we decided to use substrate **7** as the limiting reagent. Under the previously optimized 1,3-DC conditions (*cf.*Scheme 2B), but increasing the loading of **1a** to 2 equiv, cycloadduct **8** was obtained in 83% NMR yield, together with consistent amounts of furoxan **11** (80% NMR yield)—formed via dimerization of nitrile oxide **I** in excess.^22^ At this stage, the crude reaction mixture was directly transferred into the electrochemical cell–fitted with graphite electrodes and containing TBAPF_6_ and NEt_3_—and constant current was applied to the resulting solution. Crucially, by increasing the quantity of charge (*Q*) of the electrolysis to 6 F/mol, we secured the quantitative formation of **9** (83% overall yield) and the complete consumption of byproduct **11**. It is noteworthy that our telescoped procedure runs “open-flask”, employs “wet” laboratory-grade solvents and reagents, and provides the desired 1,2-*syn*-hydroxy nitrile product upon removal of the electrolyte salt.

### Mechanistic Investigations

**Scheme 3:**
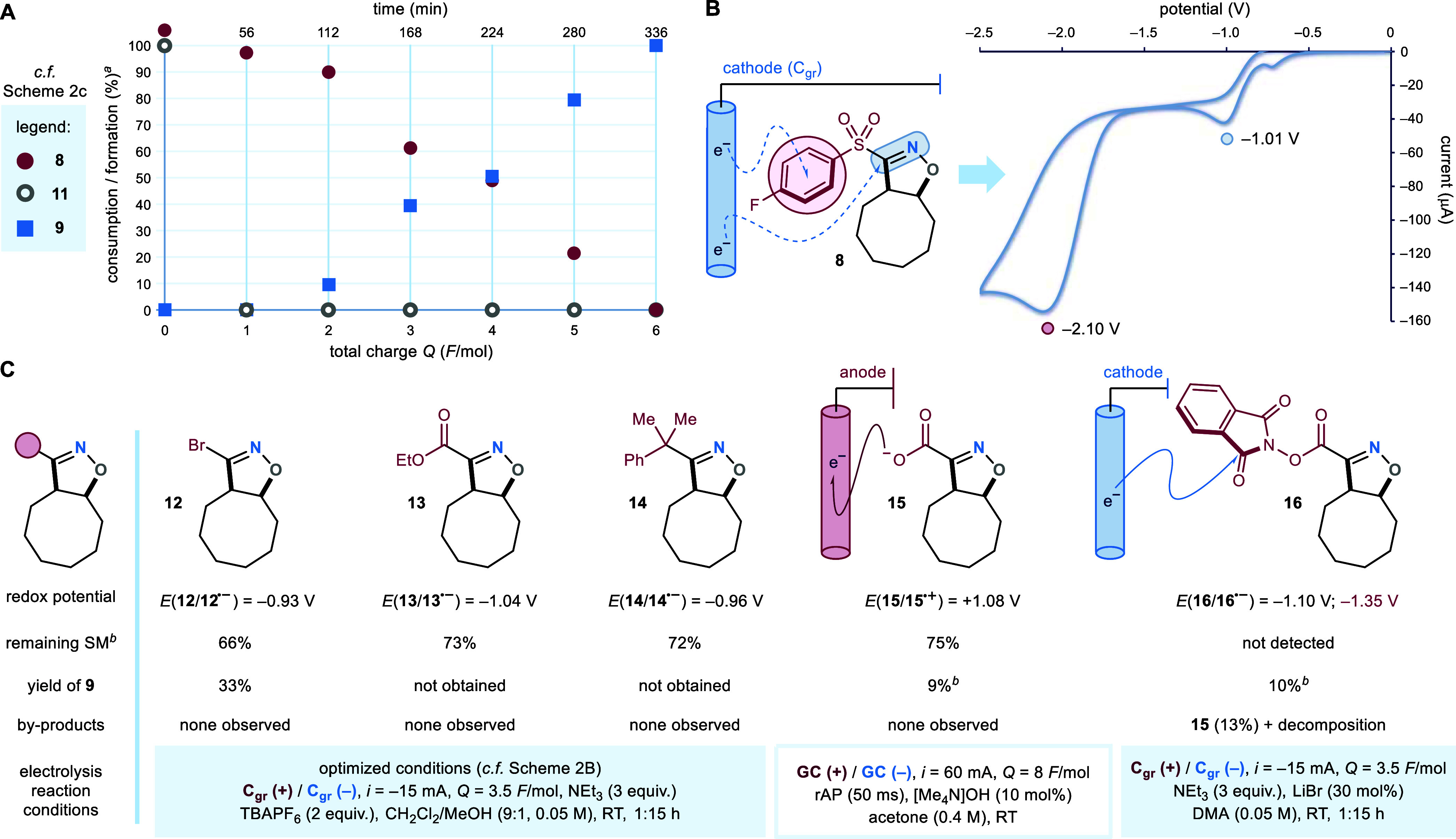
(A) Total Charge (*Q*) Dosage Study. (B) Electrochemical Characterization of Cycloadduct 8.^*a*^ (C) Influence of C3-Substitution on the Electrochemical Radical Ring-Opening of Isoxazoline Cycloadducts All reactions were performed on a 0.2 mmol scale. Cyclic voltammograms were recorded on a 0.005 M solution of the analyte in [0.1 M] TBAPF_6_ in CH_3_CN, under a sweep rate of 25 mV/s, and using a glassy carbon working electrode, an Ag/AgCl (NaCl saturated) reference electrode, and a Pt wire as auxiliary electrode. All potentials (*E*) are reported versus Ag/AgCl. ^*b*^Yields and conversions were determined by ^1^H NMR spectroscopy, using mesitylene as the internal standard. SM, starting material; GC, glassy carbon electrode; rAP, rapid alternating polarity.

**Scheme 4:**
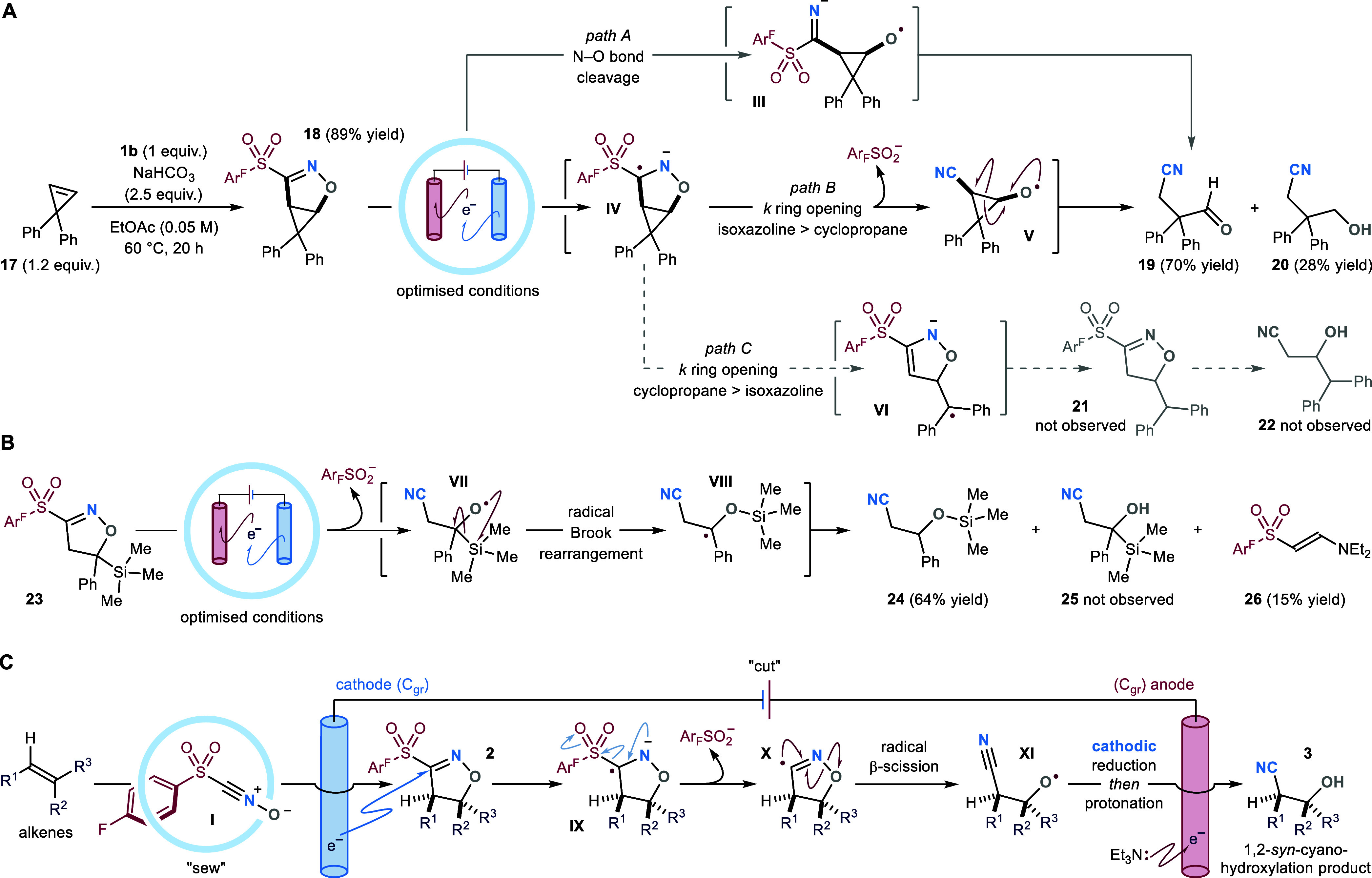
(A) Radical Clock Experiment. (B) Cascade Brook-Reactivity Observed for Cycloadduct 22. (C) Proposed Mechanistic Pathway for the Electrochemical “Sew & Cut” Protocol All reactions were performed on a 0.2 mmol scale. Optimized conditions: *C*_gr_ (+)/*C*_gr_ (−), *i* = −15 mA, *Q* = 3.5 F/mol, NEt_3_ (3 equiv), TBAPF_6_ (2 equiv), CH_2_Cl_2_/MeOH (9:1, 0.05 M), room temperature, 1:15 h. *k*, reaction rate constant.

Before exploring the scope of the methodology, we decided to gather further insights into the mechanism underlying the electrochemical radical fragmentation of the isoxazoline heterocycle. First–to ascertain the optimal *Q* for the *cut* step–six aliquots of the same crude 1,3-DC reaction mixture (containing **8** and **11**) were submitted to the optimized electrosynthetic conditions, varying the applied total charge (Scheme 3A). This study revealed that the first equimolar amount of electrons is consumed to ensure the complete degradation of furoxan **11**. After this, the electrolysis of **8** requires further 5 F/mol to reach completion–presumably, due to additional charge dissipated by the decomposition of fragmentation byproducts into volatile compounds. Accordingly, no side-product deriving from the consumption of **11** was ever observed. Occasionally, low amounts of olefin **26** (vide infra, Scheme 4B)—produced from the elimination of the *p*-fluorophenyl-sulfonyl handle^23^—were detected.

To assess at which site of the cycloadduct occurs the cathodic SET reduction, we recorded the voltammogram of **8**, which showed two reduction peaks (Scheme 3B). By comparison with literature data, the first peak at *E*_pc_ = −1.01 V is ascribable to the reduction of the isoxazoline’s oxime portion.^24^ While, the second peak at *E*_pc_ = −2.10 V is comparable to reduction potentials reported for aryl sulfone moieties.^19,25^ The CV data suggests that the SET reduction of the heterocyclic C=N bond occurs first, and this event triggers the fragmentation of **8**. To investigate this further, we tested the influence of different substituents at the C3-position of the isoxazoline ring on the electrochemical ring opening process (Scheme 3C). To this end, cyclooctene-fused isoxazolines **12**–**16** were prepared–via 1,3-DC between **7** and the corresponding halo-oxime nitrile oxide precursor–and submitted to cyclic voltammetry analyses. For all cycloadducts besides **15**, which is prone to SET oxidation, the first measured reduction potential (*E*_pc_) was consistent with that of **8**. Subsequently, we tested their behavior under the optimized electrochemical conditions. When 3-bromo-isoxazoline **12** was employed, product **9** was isolated in 33% yield together with 66% of unreacted **8** (accounting for the rest of the mass balance). Conversely, both ester derivative **13** and C3-alkyl isoxazoline **14** (which could expel, upon SET reduction, a tertiary benzylic radical leaving group)^26^ failed to deliver product **9**. In both cases, we did not observe any side reactivity, and the majority of the heterocyclic starting material was recovered at the end of the reaction. These experiments indicate that the presence of a good anionic leaving group (i.e., ArSO_2_, Br) at C3 is key to promote the desired fragmentation pathway.

Following these results, we were keen to establish whether the direct SET activation of a pedant redox-handle could also trigger an analogous radical ring-opening process. For these endeavors, we used isoxazolines **15** and **16**, whose carboxylic functionalities should prevent the radical fragmentation of the cycloadduct upon cathodic reduction of its C=N bond (*cf.* experiment with **13**). **15** and **16** were submitted to modified literature procedures–developed for the electrochemical activation of carboxylates^27^ and *N*-hydroxy-phthalimide (NHPI) esters,^28^ respectively. In the first case, electrolysis under rapid alternating polarity^29^ enabled the anodic oxidation of carboxylate **15** and delivered product **9** in 9% NMR yield. Likewise, cathodic reduction of **16** afforded *syn*-hydroxy nitrile **9**, albeit in a complex mixture with hydrolyzed substrate **15** and unidentified degradation byproducts. Despite their low efficiency (it is worth noting that both electrolysis conditions were not optimized), these experiments demonstrate that both the direct reduction of the isoxazoline ring, and the SET activation of its pendant C3-redox-handle are productive pathways toward the desired fragmentation process. More importantly, this study showcases the ability of our electrochemical strategy to promote the radical activation of isoxazoline cycloadducts under both oxidative and reductive regimes–highlighting the versatility of our approach.

### Radical Clock Experiments

Next, to both assess the rate of cycloadduct fragmentation and trace the formation of radical intermediates, we prepared cyclopropyl-fused isoxazoline **18** (via 1,3-DC between cyclopropene **17** and chloroxime **1b**) and submitted it to the electrochemical step (Scheme 4A). For this reaction, we postulated three mechanistic scenarios: *path A*, where cathodic reduction of **18** prompts the cleavage of the isoxazoline’s N–O bond, forming distonic radical anion **III**. This would then undergo sequential sulfinate elimination and radical β-scission^30^ to release the three-membered ring strain and deliver β-cyano-aldehyde **19**. Alternatively, SET to the C=N bond of **18** would generate radical anion **IV**. From **IV**, following *path B*, if the fragmentation of the isoxazoline heterocycle overcomes the rate of the cyclopropane radical ring-opening, oxygen-centered radical **V** would be produced. Even in this case, radical β-scission from **V** would yield aldehyde **19**. Conversely, following *path C*, should the fragmentation of the cyclopropyl ring dominate, the highly stabilized tertiary radical **VI** would be formed. Through downstream radical and polar reactivity, **VI** would deliver either sulfonyl-isoxazoline **21** or β-hydroxy nitrile **22** (upon further electrochemical reduction). In practice, the electrolysis of cyclopropyl-fused cycloadduct **18** afforded aldehyde **19** in 70% yield, alongside over-reduced alcohol **20** (28% yield). Based on our previous studies (Scheme 3C), we believe that the reaction proceeds through *path B*. In fact, for the electrolysis of isoxazolines **13** and **14**, no products deriving from intermediates of type **III** (i.e., β-hydroxy-imines)—nor from their over-reduction (β-hydroxy-amines)—were detected; thus ruling out *path A*. Crucially, this study suggests that the electrochemical fragmentation of C3-sulfonyl-isoxazolines is extremely fast (*N.B.* the *k*_*(20 °C)*_ for the radical ring-opening of diphenyl-cyclopropanes is 5 × 10^11^ s^–1^).^31^ From a synthetic perspective, it is worth noting that the conversion of **17** into β-hydroxy-aldehyde **19** stands as a challenging simultaneous oxidative alkene cleavage and one-carbon homologation process.

To gain further evidence of the formation of oxygen-centered radical intermediates, we submitted trimethylsilyl-substituted cycloadduct **23** to our electrosynthetic procedure (Scheme 4B). In this case, SET-promoted radical fragmentation of **23** would deliver oxygen-centered radical **VII**, which–due to the presence of a vicinal silyl group–would trigger a radical Brook rearrangement.^32^ This reactivity would lead first to α-oxy-radical **VIII**, and ultimately to silyl-ether **24**. As postulated, rearrangement product **24** was isolated in 64% yield; whereas alkene 1,2-hydroxy-cyanation product **25** was not observed. Besides **24**, the electrolysis delivered only low amounts of byproduct **26** (see discussion above). Interestingly, when vinyltrimethylsilane was submitted to the telescoped “*sew & cut*” procedure (vide infra, Table 1), the desired 1,2-hydroxy-nitrile **41** was successfully obtained, together with the corresponding Brook silyl-migration product (observed by ^1^H NMR, the compound’s instability on SiO_2_ thwarted its isolation). We believe that the predominance of the radical Brook pathway observed for **23** is ascribable to its C5-phenyl substituent, providing α-oxy-radical **VIII** with enhanced stabilization.

**Table 1:**
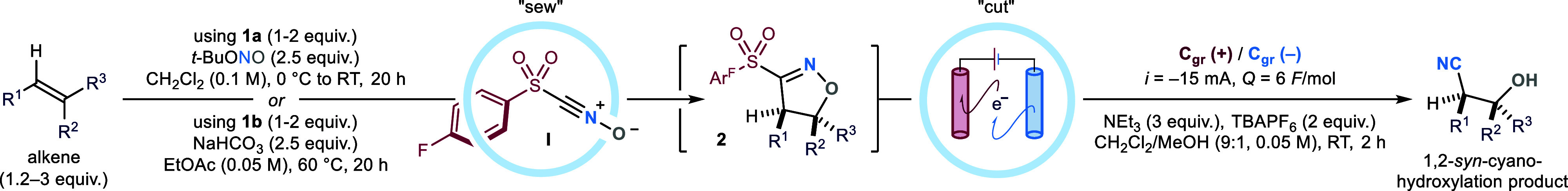

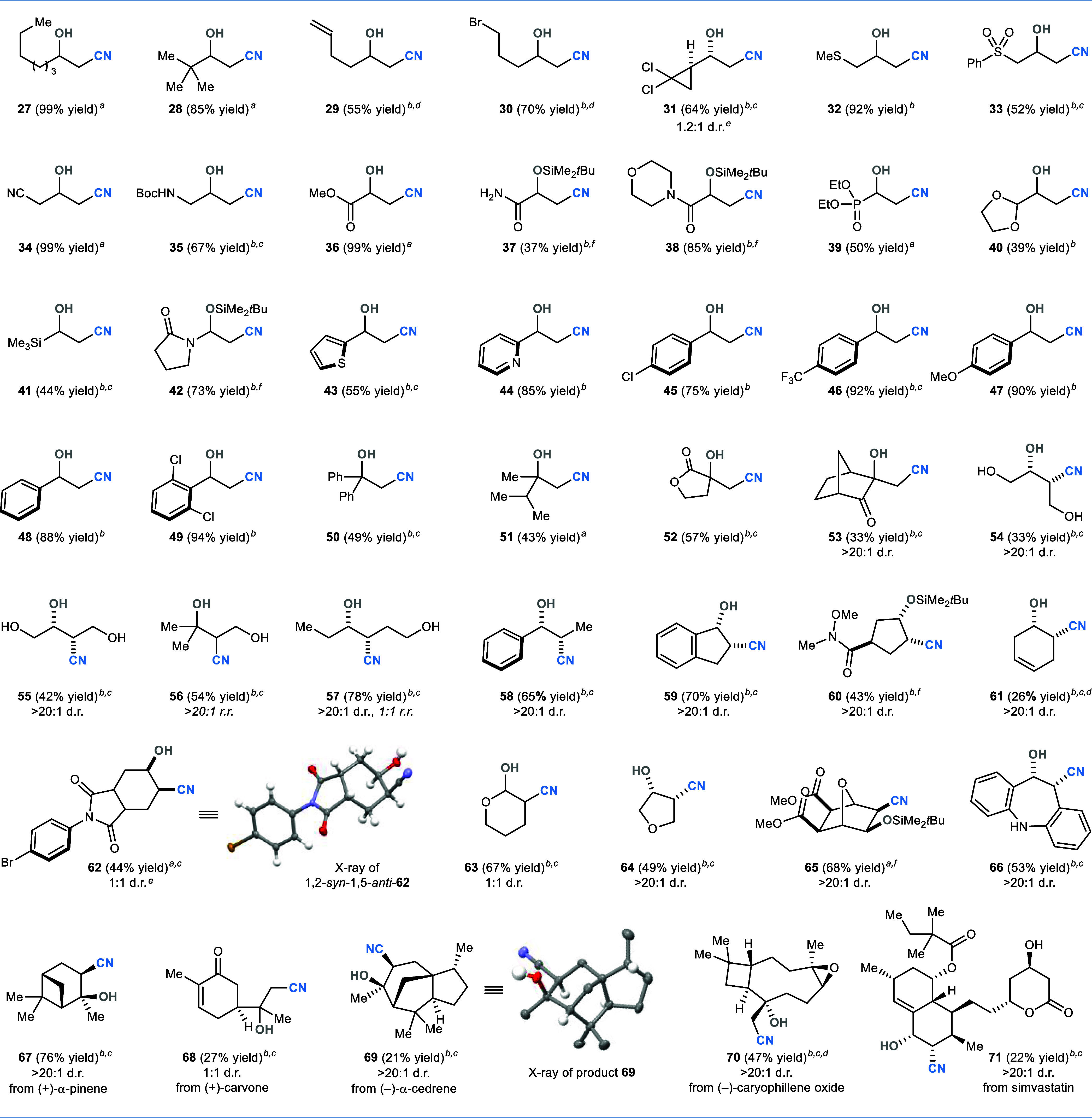
Substrate Scope for the Telescoped Alkene 1,2-*syn*-Cyano-Hydroxylation Process^a^

a All reactions were performed on a 0.2 mmol scale–besides entries **31** (0.6 mmol); and **54**, **55**, and **57** (0.4 mmol). The amount of alkene substrate used for each entry is reported in the Supporting Information

b The 1,3-DC step was performed using α-diazo-sulfone **1a**.

c The 1,3-DC step was performed using chloroxime **1b**.

d For this entry, removal of the remainder/excess olefin from the 1,3-DC crude mixture–by rapid filtration over SiO_2_—provided a higher yield for the corresponding alkene cyano-hydroxylation product (see Supporting Information).

e 1,4-Cyclohexadiene (3.5 equiv) was used as sacrificial reductant, instead of Et_3_N.

f The two diastereoisomers can be isolated separately by chromatography over silica gel.

g Due to either the instability of the hydroxy-nitrile product to SiO_2_, or to avoid its coelution with the electrolyte salt, this compound was protected as the corresponding silyl ether, prior isolation (see Supporting Information). *d.r.* diastereomeric ratio. All products were obtained as single regioisomers (*r.r.* > 20:1), unless otherwise stated (*cf.* entry **57**).

### Reaction Mechanism

Collectively, the insights gathered from the above studies support the following mechanistic proposal (Scheme 4C). Sulfonyl-nitrile oxide **I**—generated in situ from either **1a** or **1b**—engages alkene substrates in 1,3-DCs (*sew step*), forming 3-sulfonyl-isoxazoline cycloadducts **2**. Under constant current electrolysis–cathodic SET reduction of the oxime moiety of **2** forms radical anion **IX**. Ensuing elimination of the aryl-sulfinate anionic leaving group generates imidoyl radical **X**. This event triggers the fragmentation of the heterocyclic core of **X** by radical β-scission (*cut step*), delivering oxygen-centered radical **XI**. The latter is further reduced by the cathode to the corresponding anion, and later protonated by the reaction media to yield the desired 1,2-*syn*-cyano-hydroxylated product **3**. The presence of Et_3_N ensures an efficient oxidation semireaction at the anode, thus closing the electric circuit. It was later found that 1,4-cyclohexadiene also serves as a competent sacrificial reductant. Crucially, its use facilitates the purification of our products, by circumventing the formation of nonvolatile contaminants (including **26**).

### Scope of the Methodology

Using the optimized conditions reported in Scheme 2, we tested the generality of our telescoped alkene 1,2-*syn*-cyano-hydroxylation procedure (Table 1). Terminal unactivated alkenes–featuring a diverse range of functional groups on their alkyl chain–efficiently delivered hydroxy-nitriles **27**–**35**. Here, both redox-active functionalities–susceptible to either oxidation (**32**, **35**) or reduction (**30**, **33**)—and versatile synthetic handles for further derivatization (**29**–**31**, **34**) were well tolerated. Of note, dichloro-cyclopropyl product **31** was obtained as a mixture of diastereomers, which were isolated separately via chromatography. Crucially, besides unactivated alkenes, our electrochemical procedure facilitates the *syn*-difunctionalization of both electron-poor (**36**–**39**) and electron-rich olefins (**40**–**42**); bearing esters, amides, phosphates, protected aldehydes and amines, and silyl-groups. (Hetero)arenes of different electronic nature can also be accommodated: electron-rich thiophenes (**43**), electron-deficient pyridines (**44**) and various styrene derivatives–substituted at their *ortho*- and *para*-position (**45**–**49**)—all performed well in the electrochemical “*sew & cut*” procedure. The scope of the methodology was later extended to 1,1-disubstituted terminal olefins (**50**–**53**). Remarkably, bicyclic 1,2-hydroxy-nitrile **53** was obtained as a single diastereoisomer. For terminal alkenes, the regioselectivity of the cyano-hydroxylation process completely favors the regioisomer bearing of the hydroxy-group at the most substituted olefinic carbon, regardless of the C=C bond’s electronics.

Having identified the classes of olefins and functionalities tolerated by our procedure, we targeted the stereoselective *syn*-difunctionalization of internal olefins. Capitalizing on the stereospecificity of 1,3-DCs, diastereoisomers **54** and **55** were obtained selectively, by submitting either *cis*- or *trans*-2-butene-1,4-ol to our telescoped electrochemical protocol. Similarly, *trans*-β-methyl-styrene and indene successfully provided *syn*-addition products **58** and **59**, respectively, in good yields. Unsymmetrical internal linear alkenes were trialed to evaluate the regioselectivity of our protocol. For 3-methyl-2-buten-1-ol, the reaction afforded selectively product **56**—bearing the hydroxy functionality at the most substituted olefinic site. While, when using *trans*-3-hexen-1-ol, compound **57** was isolated as a 1:1 mixture of regioisomers. Diversely decorated cycloalkenes were also efficiently *syn*-difunctionalized, delivering products **60**–**62**. Here, Weinreb amide-substituted cyclopentane **60** was obtained a single diastereoisomer; whereas *syn*-hydroxy nitrile **62** was afforded as a 1:1 diastereomeric mixture. Even in this case, separation of the two diastereoisomers by column chromatography was possible, and crystallographic analysis on the 1,2-*syn*-1,5-*anti*-isomer of **62** enabled the determination of their relative stereochemistry. Next, we tested the participation of C=C bonds embedded in 5-, 6- and 7-membered oxygen- and nitrogen-heterocycles. These entries delivered stereodefined, saturated heterocycles **64**–**66** as single regio- and diastereoisomers. Conversely, racemization at the anomeric position of tetrahydropyran derivative **63** was observed. Finally, we sought to exploit our 1,2-*syn*-cyano-hydroxylation protocol for the late-stage functionalization of chiral pool molecules, natural products and pharmaceutical ingredients. Pleasingly, (+)-α-pinene, (+)-carvone, (−)-α-cedrene and (−)-caryophillene oxide all performed well under the optimized conditions, affording products **67**–**70**. For trisubstituted alkenes (**67**, **69**), the regioselectivity of the 1,2-*syn*-addition completely favors the installation of the hydroxy group at the most substituted olefinic carbon (as corroborated by X-ray analyses on cedrene derivative **69**). While, for unsymmetrical dienes (**68**, **71**), the chemoselectivity of the cyano-hydroxylation process is controlled by both the degree of alkene substitution (cyano-hydroxylation occurring at the least substituted olefin of simvastatin, vide infra) and the relative reactivity of the different C=C bonds (cyclic α,β-unsaturated carbonyls do not participate to the 1,3-DC step,^33^ as observed for (+)-carvone). To explore API manipulation, we submitted dyslipidemia treatment simvastatin to the telescoped *syn*-cyano-hydroxylation protocol. Here, in the presence of a 1,3-diene system, the reaction delivered chemo- and regioselectively product **71**, as a single stereoisomer.

### Synthetic Applications

**Scheme 5:**
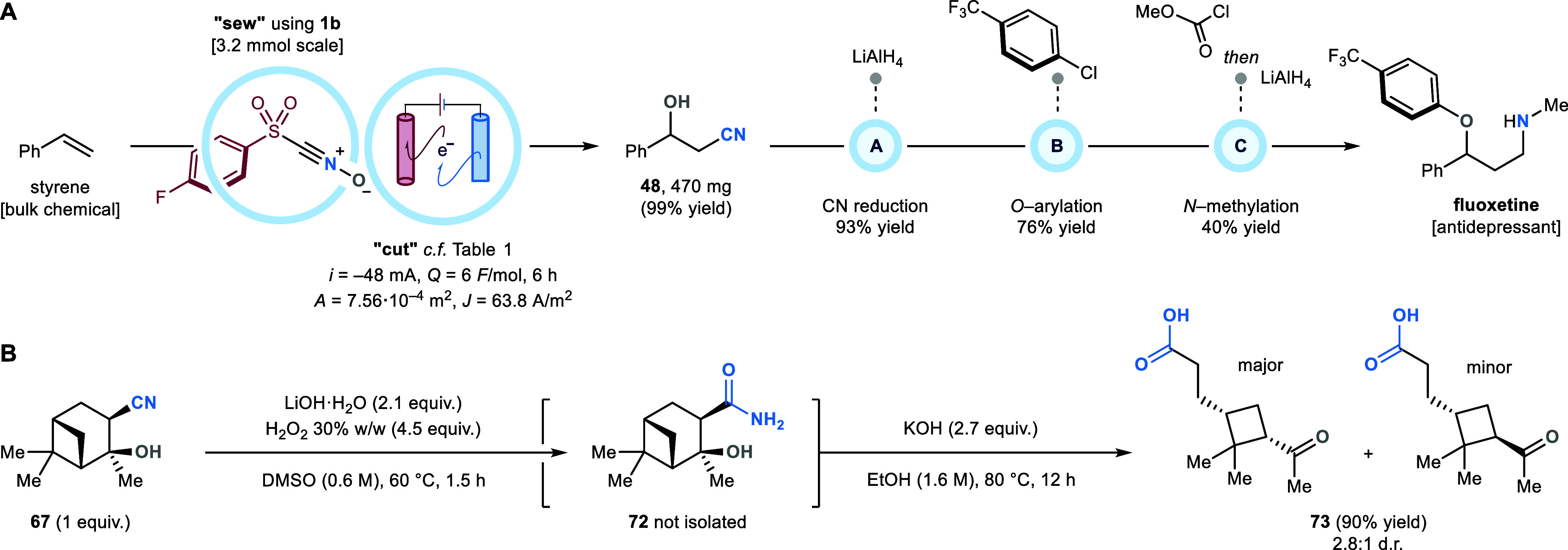
(A) Process Scale-Up, and Its Application to the Synthesis of API Fluoxetine.^*a*^ (B) Synthetic Manipulations of 1,2-*syn*-Cyano-Hydroxylation Product 67 *i*, current intensity; *Q*, quantity of charge; *A*, electrode surface; *J*, current density. Reaction Conditions: (A) LiAlH_4_ (2 equiv), THF, 0 °C to room temperature, 2 h; (B) 4-chlorobenzotrifluoride (1.5 equiv), NaH (1.5 equiv), DMSO, 90 °C, 2 h; (C) methyl chloroformate (1.3 equiv), K_2_CO_3_ (5 equiv), H_2_O:CH_2_Cl_2_ (2:1), room temperature, 30 min *then* LiAlH_4_ (2 equiv), THF, room temperature, 2 h. ^*b*^Procedure conducted on a 837 μmol scale.

To showcase the synthetic utility of our approach, we scaled the “*sew & cut*” protocol between nitrile oxide precursor **1b** and styrene up to 3.2 mmol (Scheme 5A). By adjusting the electrochemical conditions to maintain constant the current density (*J*) within the electrochemical cell, product **48** (470 mg) was obtained in quantitative yield. We then sought to exploit the newly installed OH- and CN-functionalities of **48** to convert it into biologically active compounds. To this end, we reduced its nitrile group to a primary amine by treatment with LiAlH_4_, and then *O*-arylated its hydroxy moiety via S_N_Ar with 4-chlorobenzotrifluoride to deliver selective serotonin reuptake inhibitor seproxetine. This was then *N*-monomethylated (through sequential *N*-protection with methyl chloroformate and LiAlH_4_ reduction) to afford antidepressant fluoxetine in 28% yield, over four synthetic steps from styrene. Alternative manipulation procedures were conducted on (+)-α-pinene derivative **67**. This was first exposed LiOOH to convert its nitrile moiety into primary amide **72** (Scheme 5B, this compound was not isolated), and later treated with KOH in EtOH at 80 °C. Crucially, the latter step promoted both the hydrolysis of the amide group the corresponding carboxylate, and the C–C bond cleavage of the original olefinic carbons of (+)-α-pinene; delivering stereodefined cyclobutene **73** in 90% yield over two telescoped steps from **67**. Compound **73** was isolated as a 2.8:1 mixture of diastereoisomers, presumably due to KOH-assisted epimerization of the secondary α-keto position of **73**, under the hydrolysis conditions.

## Conclusions

This study demonstrates that the robustness, stereospecificity and generality of 1,3-DCs can be exploited in combination with radical activation to realize broad-scope, regio- and stereoselective alkene 1,2-*syn*-difunctionalization processes, bypassing the use of transition metal-catalysis. Through the design of novel bifunctional reagents, comprising a nitrile oxide 1,3-dipole precursor linked to an aryl-sulfonyl moiety, we have developed efficient electrochemical conditions that facilitate the controlled radical fragmentation of isoxazoline cycloadducts–via their direct cathodic SET reduction. These conditions have been implemented into a telescoped procedure that converts a variety of electron-rich, electron-poor and unactivated olefins–featuring a broad range of functional groups–into 1,2-*syn*-hydroxy nitrile derivatives; with high levels of chemo-, regio- and diastereo-selectivity. Our electrochemical approach unlocks the full synthetic potential of the “isoxazoline route”, by (*i*) expanding its generality to all classes of alkenes; (*ii*) broadening its functional group tolerance; and enhancing its (*iii*) robustness, (*iv*) experimental ease and (*v*) synthetic applicability. Capitalizing on these features, we have applied our method to the late-stage functionalization of natural products, and the manipulation/preparation of pharmaceutical ingredients. We believe that our investigations will inspire the design of alternative reagents and reactions exploiting the untapped potential of 1,3-DCs (and, in general, of pericyclic reactions) in the radical domain;^14^ by taking advantage of the state-of-the-art technologies for radical generation and reactivity.^34,35^

## Supporting Information Available

Additional experimental procedures and details, materials and methods, ^1^H NMR and ^13^C NMR spectra for all compounds, cyclic voltammetry profiles, and crystallographic data. Correspondence and requests for materials and raw data should be addressed to giacomo.crisenza@manchester.ac.uk (PDF)
