# Supplementary material for: General Alkene 1,2-syn-Cyano-Hydroxylation Procedure Via Electrochemical Activation of Isoxazoline Cycloadducts
Source: J Am Chem Soc. 2024 Nov 13;146(47):32848–58. doi: 10.1021/jacs.4c13682 (PMC11613428; doi:10.1021/jacs.4c13682)

Supplementary Information for

A General Alkene 1,2-syn-Cyano-Hydroxylation Procedure via Electrochemical Activation of Isoxazoline Cycloadducts.

Taciano A. S. Wanderley,[1] Roberto Buscemi,[1] Órla Conboy,[1] Benjamin Knight[1] and Giacomo E. M. Crisenza[1]*

[1]*Department of Chemistry – The University of Manchester*
Oxford Road, Manchester, M13 9PL (UK).

*Correspondence to giacomo.crisenza@manchester.ac.uk

Table of Contents

S.1 – General Information 1

S.2 – Substrate Synthesis 2

2.1 – Synthesis of Alkene Substrates

2.2 – Synthesis of 1,3-Dipole Precursors **1**

S.3 – Optimization Studies 5

3.1 – Optimization of the 1,3-Dipolar Cycloaddition Step (*sew*)

■ ***General Procedures A-B***

3.2 – Optimization of the Electrochemical Ring-Opening Step (*cut*)

■ ***General Procedure C***

3.3 – Photochemical Ring-Opening of Isoxazoline **8**

3.4 – Zinc-mediated Ring-Opening of Isoxazoline **8**

3.5 – Development of a Telescoped "*Sew & Cut*" Protocol

■ ***General Procedures D-E***

S.4 – Synthesis of Isoxazoline Cycloadducts 10

■ Synthesis and Characterization of Compounds **8**, **11**, **12**, **13**, **14**, **15**, **16**, **18** and **23**

S.5 – Mechanistic Investigations 15

5.1 – Total Charge (*Q*) Dosage Study (*Scheme 3A*)

5.2 – Voltammetric Analysis

■ CV Characterization of **8**, **9**, **11**, **12**, **13**, **14**, **15** and **16**

5.3 – Influence of C3-Substitution on the Electrochemical Radical Ring-Opening of Isoxazoline Cycloadducts (*Scheme 3C*)

5.4 – Radical Clock Experiments (*Scheme 4A-B*)

■ Electrolysis of **18** and **23** | Characterization Data of Compounds **19**, **20**, **24** and **26**

S.6 – Scope of the Methodology 25

6.1 – Scope Limitations

6.2 – Characterization Data of Compounds **9** and **27-71** (*Table 1*)

S.7 – Synthetic Applications 45

7.1 – Scale-Up of the Telescoped Process Between **1b** and styrene to give **48**

7.2 – Synthetic Route to Fluoxetine

7.3 – Synthetic Manipulations of **67**

S.8 – X-ray Crystallographic Data 49

■ Single Crystal X-ray Diffraction Data for Compounds **1b**, **62** and **69**

S.9 – References 54

S.10 – NMR Spectra (^{1}H, ^{13}C, ^{19}F and ^{31}P Traces) 56

S.1 – General Information

Analytical Information: Novel compounds were characterized by NMR spectroscopy and HRMS spectrometry. The NMR spectra were recorded at 400 MHz and 500 MHz for ^{1}H; at 101 MHz and 125 MHz for ^{13}C; at 376 MHz for ^{19}F and at 162 and 202 MHz for ^{31}P. The chemical shift (δ) values are reported in parts per million (ppm) relative to the residual solvent signals (using $CDCl_3$: signal @ 7.26 ppm for ^{1}H NMR, and 77.16 ppm for ^{13}C NMR; using CD_3CN: signal @ 1.96 ppm for ^{1}H NMR, and 118.26 ppm for ^{13}C NMR; using MeOD: signal @ 3.31 ppm for ^{1}H NMR, and 49.00 ppm for ^{13}C NMR). All coupling constants (*J*) are reported in Hertz (Hz). Splitting patterns are assigned s = singlet, d = doublet, t = triplet, q = quartet, sext = sextet, hept = heptuplet and m = multiplet. Additionally, signals can be described as broad (br.) and apparent (app).

Mass spectra were obtained using positive and/or negative electrospray (ESI±), atmospheric-pressure chemical ionisation (APCI) or gas chromatography (GC) techniques. Measurements were carried out by the Mass spectrometry Service of Department of Chemistry at the University of Manchester. Cyclic voltammetry studies were conducted using a PalmSens MultiEmStat4 multi-channel potentiostat offering compliance voltage up to ± 3 V (available at the counter electrode), ± 3 V scan range and ± 30 mA current range. Cyclic voltammograms were recorded on a 0.005M solution of the analyte (unless otherwise stated) in [0.1 M] $TBAPF_6$ in CH_3CN, under a sweep rate of 25 mV/s, and using a glassy carbon working electrode, an Ag/AgCl (NaCl saturated) reference electrode, and a Pt wire as auxiliary electrode.

Experimental Information and Materials: Unless otherwise stated, all experiments were performed “open-flask” (*i.e.* without taking any precaution to secure an inert atmosphere nor anhydrous conditions), using laboratory grade solvents and standard glassware. Solvents and reagents were purchased from commercial sources (Sigma Aldrich, Fluka, Acros Organics, TCI, Apollo Scientific, Fluorochem or Alfa Aesar) and used as supplied.

Electrosynthetic experiments were performed on an IKA ElectraSyn 2.0 electrochemical station, using pre-fabricated electrodes [*dimensions (W x H x D): 3 x 56 x 1 mm for microelectrodes; and 8 x 52.5 x 2 mm for standard electrodes*] and fitted reaction vials – purchased from IKA. Photochemical reactions were subjected to irradiation from a 34W Kessil blue LED lamp, with the reaction tube placed approximately 2 cm from the bulb. Organic solutions were concentrated under reduced pressure on standard rotary evaporators (*in vacuo* at 40 °C, ~5 mbar). Chromatographic purification of products was accomplished using force-flow flash chromatography (FC) on silica gel (35-70 mesh). Thin layer chromatography (TLC) analyses were carried out using precoated aluminum sheets (silica gel 60 F254, 0.2 mm), and viewed under a 254 nm UV-lamp, and/or visualized by staining with common developing solutions (potassium permanganate, *p*-anisaldehyde, phosphomolybdic acid, or vanillin).

S.2 – Substrate Synthesis

S.2.1 – Synthesis of Alkene Substrates: Olefins that were not available from commercial sources were synthesized following literature procedures – providing the desired alkene substrates with akin levels of purity and efficiency, as previously reported. This is the case of cycloprop-2-ene-1,1-diyldibenzene **17**,[1] trimethyl(1-phenylvinyl)silane (precursor to cycloadduct **23**),[2] *N*-methoxy-*N*-methylcyclopent-3-ene-1-carboxamide (precursor to product **58**),[3] *cis*-1,2,3,6-*N*-(4-bromophenyl)-tetrahydrophthalimide (precursor to product **59**)[4] and methyl *exo*-3,6-epoxy-1,2,3,6-tetrahydrophthalate (precursor to product **63**).[5]

S.2.2 – Synthesis of 1,3-Dipole Precursors 1

S.2.2.1 – *Synthetic Route to α-Diazo-Sulfone Reagent **1a**:*

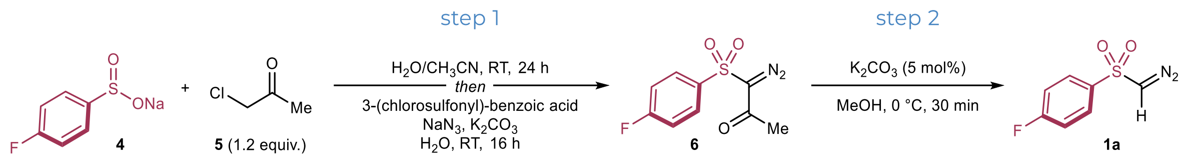

■ **Step 1** – A solution of the sulfinate salt **4** (1 equiv.) and chloroacetone **5** (1.2 equiv.) in a 2:1 mixture of CH_3CN and water (0.5 M) was stirred at 60 °C for 24 h. The reaction mixture was then cooled down to room temperature and transferred to a well-stirred solution of (3-chlorosulfonyl)-benzoic acid (1.25 equiv.), NaN_3 (1.2 equiv.) and K_2CO_3 (1.5 equiv.) in water (0.25 M). Completed the addition, the reaction mixture was further diluted with CH_3CN (1 mL/mmol), and stirred at room temperature for 16 hours. The crude mixture was extracted with CH_2Cl_2. The combined organic fractions were washed with brine, dried over $MgSO_4$ and the solvent removed under reduced pressure. Purification of the crude material by recrystallisation (from CH_2Cl_2:MeOH=1:5) delivered diazo-compound **6** (64% yield) as a yellow solid. *This compound was stored in the dark, at –20 °C.* The spectroscopic properties of this compound were consistent with the data available in the literature.[6]

When the procedure was conducted on a 100 mmol-scale – using 18.2 g of sulfinate salt **4** and 10.5 mL of chloroacetone **5** – the reaction afforded 15.2 g of product **6** (63% yield).

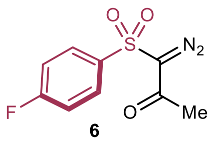

6

^{1}H NMR (400 MHz, $CDCl_3$) δ 8.32 – 7.76 (m, 2H), 7.27 (app t, J = 8.5 Hz, 2H), 2.29 (s, 3H). **^{13}C NMR** (101 MHz, $CDCl_3$) δ 185.4, 165.9 (d, $^1J_{C\text{–}F}$ = 257.7 Hz), 137.8 (d, $^4J_{C\text{–}F}$ = 3.3 Hz), 130.5 (d, $^3J_{C\text{–}F}$ = 9.7 Hz), 116.8 (d, $^2J_{C\text{–}F}$ = 22.7 Hz), 27.0; $\underline{C}_qN_2$ signal not observed. **^{19}F NMR** (376 MHz, $CDCl_3$) δ –102.10.

■ **Step 2** – To a stirring solution of compound **6** (1 equiv.) in anhydrous MeOH (0.06 M), at 0 °C and under nitrogen atmosphere, was added K_2CO_3 (0.05 equiv.). The resulting mixture was stirred for 30 min at 0 °C, and poured into a separating funnel containing water. The crude mixture was extracted with CH_2Cl_2, and the combined organic layers were washed

with brine, dried over Na_2SO_4. Removal of solvent afforded product **1a** (94% yield) as an amber oil. No further purification procedures were required. ***N.B.*** *This compound can be stored in the dark, at –20 °C, up to two weeks. After this time, its use provides reduced yields for 1,3-DC reactions, under optimized conditions. Nevertheless, the "expired" material can be converted into chloroxime **1b** (see next section) with akin efficiency.* The spectroscopic properties of this compound were consistent with the data available in the literature.[7]

When the procedure was conducted on a 24 mmol-scale – using 5.88 g of diazo-compound **6** and 1.20 g of K_2CO_3 – the reaction afforded 4.53 g of product **1a** (94% yield).

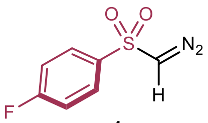

1a

^{1}H NMR (400 MHz, $CDCl_3$) δ 7.96 – 7.84 (m, 2H), 7.33 – 7.17 (m, 2H), 5.34 (s, 1H). **^{13}C NMR** (101 MHz, $CDCl_3$) δ 165.4 (d, $^1J_{C\text{–}F}$ = 256.0 Hz), 140.2 (d, $^4J_{C\text{–}F}$ = 3.3 Hz), 129.1 (d, $^3J_{C\text{–}F}$ = 9.7 Hz), 116.7 (d, $^2J_{C\text{–}F}$ = 22.7 Hz), 57.8. **^{19}F NMR** (376 MHz, $CDCl_3$) δ –103.9.

S.2.2.2 – *Synthesis of Chloroxime Reagent **1b**:*

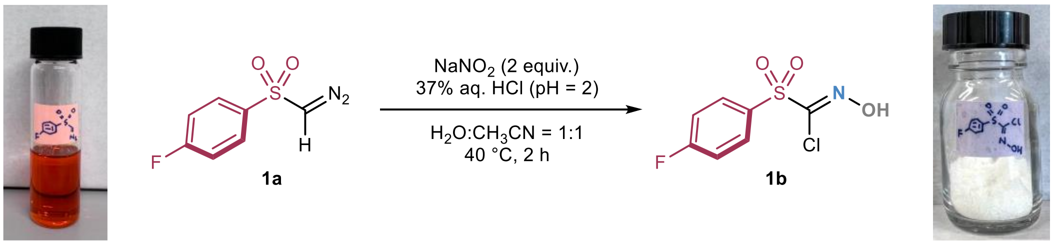

A stirring solution of diazo compound **1a** (1 equiv.) and $NaNO_2$ (2 equiv.) in a 1:1 mixture of water and CH_3CN (0.06 M), at 40 °C, was acidified to pH 2.0 with concentrated HCl (~ 0.6 mL/mmol); and stirred for 2 hours. After this time, the reaction was quenched by addition of sulfamic acid (1 equiv.), and the resulting mixture was extracted with CH_2Cl_2. The combined organic layers were washed with brine, dried over $MgSO_4$, filtered, and the solvent removed in vacuo. The residue was purified by recrystallisation (from boiling $CHCl_3$) to afford chloroxime **1b** (53% yield) as a colorless solid. ***N.B.*** *This compound can be stored under air and at room temperature, indefinitely (longest time in the Crisenza lab: 9 months).* The title compound was previously unknown.

When the procedure was conducted on a 33.4 mmol-scale – using 6.69 g of diazo-sulfone **1a** and 4.61 g of $NaNO_2$ – the reaction afforded 4.16 g of product **1b** (53% yield)

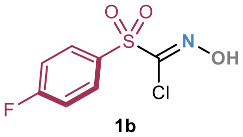

1b

^{1}H NMR (500 MHz, $CDCl_3$) δ 9.37 (s, 1H), 8.08 – 8.00 (m, 2H), 7.35 – 7.28 (m, 2H). **^{1}H NMR** (500 MHz, DMSO-d_6) δ 13.91 (s, 1H), 8.11 – 8.04 (m, 2H), 7.62 – 7.54 (m, 2H). **^{13}C NMR** (126 MHz, DMSO-d_6) δ 166.5 (d, $^1J_{C\text{–}F}$ = 255.8 Hz), 137.4, 133.0 (d, $^3J_{C\text{–}F}$ = 10.3 Hz), 132.5 (d, $^4J_{C\text{–}F}$ = 3.0 Hz), 117.9 (d, $^2J_{C\text{–}F}$ = 23.0 Hz). **^{19}F NMR** (376 MHz, DMSO-d_6) δ –101.7. **HRMS** (APCI$^-$) Exact mass calculated for $C_7H_4ClFNO_3S$ $[M–H]^-$: 235.9590, found: 235.9583. Compound **1b** was further characterized by X-ray crystallographic analysis (*see Section S8.1*).

S.2.2.3 – *Synthetic Route to α-Diazo-Sulfone Reagent* ***1c****:*

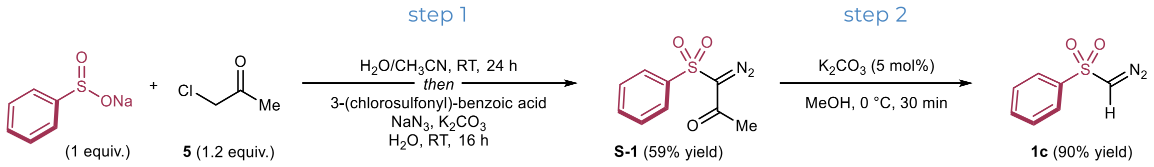

■ **Step 1** – A solution of the benzenesulfinic acid sodium salt (1.64 g, 10.0 mmol) and chloroacetone **5** (1.15 mL, 13.0 mmol) in a 2:1 mixture of CH_3CN and water (15 mL) was stirred at 60 °C for 24 h. The reaction mixture was then cooled down to room temperature and transferred to a well-stirred solution of (3-chlorosulfonyl)-benzoic acid (2.76 g, 12.5 mmol), NaN_3 (975 mg, 15.0 mmol) and K_2CO_3 (2.07 g, 15.0 mmol) in water (20 mL). Completed the addition, the reaction mixture was further diluted with CH_3CN (20 mL), and stirred at room temperature for 16 hours. The crude mixture was extracted with CH_2Cl_2. The combined organic fractions were washed with brine, dried over $MgSO_4$ and the solvent removed under reduced pressure. The crude material was purified by flash column chromatography (*isocratic in* 50% EtOAc in hexane) to provide diazo-compound **S-1** (1.42 g, 59% yield) as a yellow solid. *This compound was stored in the dark, at –20 °C.* The spectroscopic properties of this compound were consistent with the data available in the literature.[8]

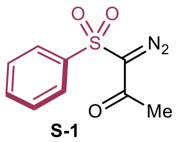

S-1

^{1}H NMR (500 MHz, $CDCl_3$) δ 8.01 – 7.95 (m, 2H), 7.73 – 7.65 (m, 1H), 7.60 (dd, *J* = 8.5, 7.1 Hz, 2H), 2.30 (s, 3H). **^{13}C NMR** (126 MHz, $CDCl_3$) δ 185.7, 142.0, 134.2, 129.6, 127.3, 85.9, 27.1.

■ **Step 2** – To a stirring solution of compound **S-1** (977 mg, 4.00 mmol) in anhydrous MeOH (60 mL), at 0 °C and under nitrogen atmosphere, was added K_2CO_3 (28.0 mg, 0.20 mmol). The resulting mixture was stirred for 30 min at 0 °C, and poured into a separating funnel containing water. The crude mixture was extracted with CH_2Cl_2, and the combined organic layers were washed with brine and dried over Na_2SO_4. Removal of the solvent under reduced pressure delivered product **1c** (725 mg, 90% yield) as a yellow oil. No further purification procedures were required. *This compound can be stored in the dark, at –20 °C, up to two weeks.* The spectroscopic properties of this compound were consistent with the data available in the literature.[9]

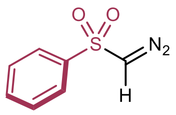

1c

^{1}H NMR (400 MHz, $CDCl_3$) δ 7.92 – 7.84 (m, 2H), 7.66 – 7.60 (m, 1H), 7.59 – 7.53 (m, 2H), 5.31 (s, 1H). **^{13}C NMR** (101 MHz, $CDCl_3$) δ 144.1, 133.4, 129.5, 126.2, 57.7.

S.3 – Optimization Studies

S.3.1 – Optimization of 1,3-Dipolar Cycloaddition Step (*sew*):

S.3.1.1 – We optimized the reaction between diazo-compound **1a** (as the nitrile oxide precursor) and *cis*-cyclooctene **7** (as the dipolarophile). The key findings of this study are summarized in the table below:

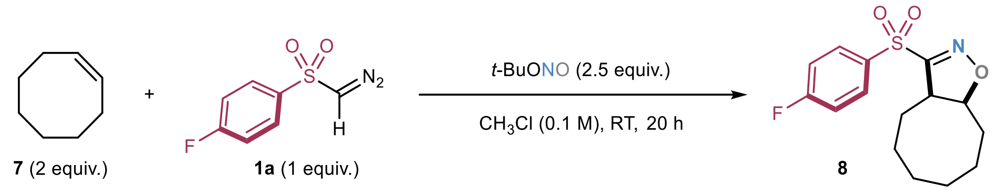

entry	variation from standard conditions	yield of 8 (%)[a]
1	None	**80**
2	1.2 equiv. of olefin	**60**
3	3 equiv. of olefin	**98**
4	5 equiv. of olefin	**91**
5	2 equiv. of **1a** and 1 equiv. of olefin **7**	**78**
6	CH_2Cl_2 as solvent	**94 (97)**
7	1,2-DCE as solvent	**92**
8	CH_3CN as solvent	**75**
9	DMF as solvent	**69**
10	THF as solvent	**91**
11	PhMe as solvent	**62**
12	CH_2Cl_2:MeOH 9:1 as solvent	**47**
13	2 equiv. of **1a** and 1 equiv. of olefin **7**, CH_2Cl_2 as solvent	**95**
**14**	**3 equiv. of olefin 7, CH_2Cl_2 as solvent**	**99 (99)**
15	$BF_3.Et_2O$ (10 mol%) as additive	**41**

[a]Yields were determined by ^{1}H NMR spectroscopy, using mesitylene as the internal standard. Isolated yield reported in parenthesis.

Following this study, we implemented a general procedure for the **synthesis of isoxazoline cycloadducts from alkenes and α-diazo-sulfone 1a:**

■ **General Procedure A:** To a solution of the α-diazo-sulfone **1a** (1 equiv.) and alkene substrate (3 equiv.) in CH_2Cl_2 (0.1 M), at 0 °C, was slowly added a solution of *t*-BuONO (2.5 equiv.) in CH_2Cl_2 (0.1 M), over 1 hour [*N.B. the use of a syringe pump, to control the rate of addition of t-BuONO, is crucial to achieve high yields and limit the formation of dimerization byproducts*]. Completed the addition, the resulting solution was allowed to warm up to room temperature, and stirred for 20 hours. At the end of the reaction, the solvent was removed under reduced pressure, and the crude mixture was purified by flash column chromatography on silica gel support (under the conditions reported for each entry) to yield the desired isoxazoline cycloadduct.

S.3.1.2 – Nucleophilic olefins or alkenes bearing nucleophilic functionalities react with *t*-BuONO, thus preventing the formation of the reactive nitrile oxide intermediate from **1a**. To overcome this issue and provide an alternative set of 1,3-DC conditions, we optimized the reaction between chloroxime **1b** (as the nitrile oxide precursor) and styrene (as the dipolarophile). The key findings of this study are summarized in the table below:

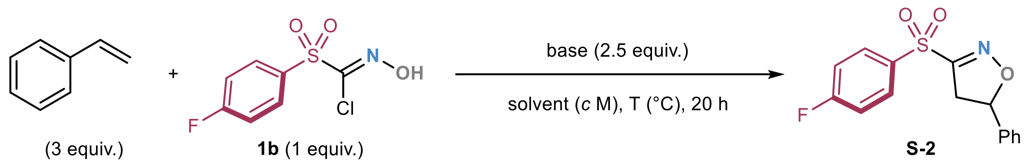

entry	base	solvent (*c* M)	T (°C)	yield of S-2 (%)[a]
1	Et_3N	CH_2Cl_2 (0.1 M)	RT	**24**
2	Et_3N	Et_2O (0.1 M)	RT	**5**
3	Et_3N	PhMe (0.1 M)	RT	**25**
4	Et_3N	PhMe (0.1 M)	60 °C	**68**
5	$NaHCO_3$	CH_3CN:H_2O 1:1 (0.1 M)	RT	**33**
6	$NaHCO_3$	DMF:H_2O 1:1 (0.1 M)	RT	**8**
7	$NaHCO_3$	THF:H_2O 1:1 (0.1 M)	RT	**61**
8	$NaHCO_3$	EtOAc (0.1 M)	RT	**75**
9	$NaHCO_3$	CH_3CN (0.1 M)	RT	**10**
10	$NaHCO_3$	EtOAc (0.1 M)	60 °C	**89**
11	$NaHCO_3$	EtOAc (0.1 M)	60 °C	**38**
**12**	**$NaHCO_3$**	**EtOAc (0.05 M)**	**60 °C**	**98 (93)**
13	$NaHCO_3$	EtOAc (0.02 M)	60 °C	**94 (85)**
14[b]	$NaHCO_3$	EtOAc (0.02 M)	60 °C	**56**
**15[c]**	**$NaHCO_3$**	**EtOAc (0.02 M)**	**60 °C**	**93**

[a]Yields were determined by ^1H NMR spectroscopy, using mesitylene as the internal standard. Isolated yield reported in parenthesis. [b]Reaction run using 2 equiv. of **1b** and 1 equiv. of styrene. [c]Reaction run using 1 equiv. of **1b** and 1.2 equiv. of styrene.

Following this study, we implemented a general procedure for the **synthesis of isoxazoline cycloadducts from alkenes and chloroxime 1b:**

■ **General Procedure B:** To a vigorously stirring solution of $NaHCO_3$ (2.5 equiv.) and alkene substrate (1.2-3 equiv., as reported for each entry) in EtOAc (0.05 M), at room temperature, was added chloroxime **1b** (1 equiv.) in one portion. The resulting mixture was then heated to 60 °C, and stirred for 20 hours. After this time, the reaction was allowed to cool down to room temperature and the crude mixture was filtered through a short pad of celite. The filtrate was concentrated under reduced pressure, and the residue was purified by flash column chromatography on silica gel support (under the conditions reported for each entry) to yield the desired isoxazoline cycloadduct.

S.3.2 – Optimization of the Electrochemical Ring-Opening Step (*cut*): To facilitate the radical fragmentation of the isooxazoline ring, we optimized the electrolysis, under reductive regime, of cycloadduct **8**. The key findings of this study are summarized in the table below:

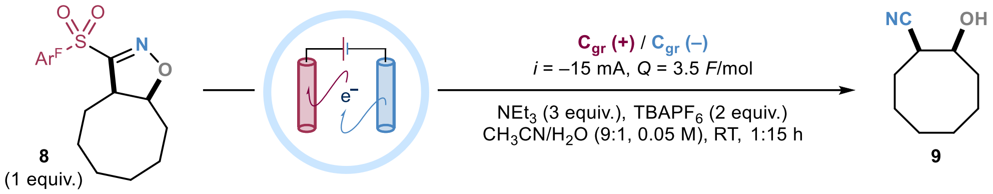

entry	variation from standard conditions	remaining 8 (%)[a]	yield of 9 (%)[a]
1	None	ND	99 (95)
2	$TBABF_4$ as electrolyte	ND	99
3	TBAOH as electrolyte	ND	**54**
4	Ph_3N (3 equiv.) as sacrificial reductant	8	**74**
5	Et_3SiH (1.5 equiv.) as sacrificial reductant	43	**51**
6	1,4-CHD (3 equiv.) as sacrificial reductant	10	**85**
7	CH_3CN as solvent	25	**63**
8	DMF:H_2O 9:1 as solvent	ND	98
9	THF:H_2O 9:1 as solvent	13	**86**
10	MeOH as solvent	8	**91**
11	CH_2Cl_2:MeOH 9:1 as solvent	ND	95 (94)
12	as above, 1 equiv. of $TBAPF_6$, 1.5 equiv. Et_3N	18	**81**
13	CH_2Cl_2:MeOH 9:1 solvent, 1 equiv. of $TBAPF_6$	10	**90**

[a]Yields were determined by ^{1}H NMR spectroscopy, using mesitylene as the internal standard. Isolated yield reported in parenthesis. ND, not detected; 1,4-CHD, 1,4-cyclohexadiene.

Following this study, we implemented a general procedure for the **electrochemical radical ring-opening of isoxazoline cycloadducts to deliver 1,2-*syn*-hydroxy nitriles:**

■ **General Procedure C:** A 5 mL IKA ElectraSyn® vial – fitted with carbon graphite microelectrodes (both working and counter electrode) – was charged with the isoxazoline substrate (0.2 mmol, 1 equiv.), $TBAPF_6$ (2 equiv.), Et_3N (3 equiv.) and 4 mL of a CH_3CN:H_2O 9:1 solution. The vial was sealed and mounted on an IKA ElectraSyn® station. The electrolysis was conducted under constant current regime (–15 mA), with a total charge (*Q*) of 3.5 F/mol. Completed the electrolysis, the crude mixture was concentrated under reduced pressure, and the residue was purified by flash column chromatography on silica gel support (under the conditions reported for each entry) to yield the desired 1,2-*syn*-hydroxy nitrile product.

Commands Workflow: select "*New Experiment*" > select "*Constant Current*" > set current to –15 mA > select "*No*" for reference electrode > select "*Total Charge*" > set 0.20 mmol of substrate and 3.5 F/mol > select "*No*" for alternating polarity > save the experimental conditions, if desired > select "*Start*" to initiate the electrolysis > set stirring to 1000 rpm.

Useful Information: At the end of the reaction, the electrodes were washed with CH_2Cl_2, then water, then acetone – gently cleaning their surface with a clean tissue.

S.3.3 – Photochemical Ring-Opening of Isoxazoline 8:

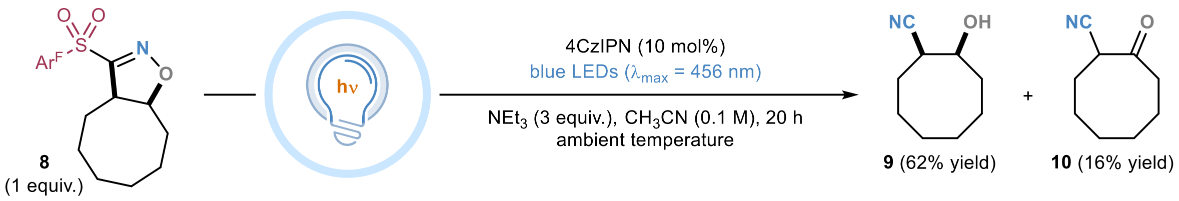

An oven-dried vial was charged with isoxazoline **8** (62.3 mg, 0.20 mmol), photocatalyst 4CzIPN (CAS: 1416881-52-1, 15.7 mg, 20.0 μmol) and NEt_3 (83.0 μL, 0.60 mmol). The tube was sealed with a rubber septum and flushed with nitrogen, through a needle inserted into the cap, for 5 minutes. Nitrogen-spurged CH_3CN (2 mL) was then added to the vessel, and the resulting mixture was irradiated using a blue LEDs Kessil lamp (λ_{max} centred at 456 nm), and stirred at ambient temperature (as enabled by the use of a fan) for 20 hours. After this time, the crude mixture was transferred into a round-bottomed flask and the volatiles were removed under reduced pressure. Purification of the crude material by flash column chromatography on silica gel support (*gradient from* hexane 100% *to* 30% EtOAc in hexane) delivered a 4:1 mixture of 1,2-*syn*-hydroxy nitrile **9** and α-cyano ketone **10** (23.0 mg, 75% overall yield) as an amber oil.

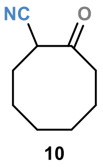

*Characteristic signals for byproduct **10** – these were consistent with the data available in the literature.*[10] **^{1}H NMR** (400 MHz, $CDCl_3$, *resolved characteristic signals only*) δ 3.66 (dd, J = 7.5, 3.5 Hz, 1H), 2.64 – 2.45 (m, 2H). **^{13}C NMR** (101 MHz, $CDCl_3$) δ 206.7, 117.1, 44.1, 40.3, 28.9, 26.9, 26.3, 24.6, 24.2.

S.3.4 – Zinc-Mediated Ring-Opening of Isoxazoline 8:

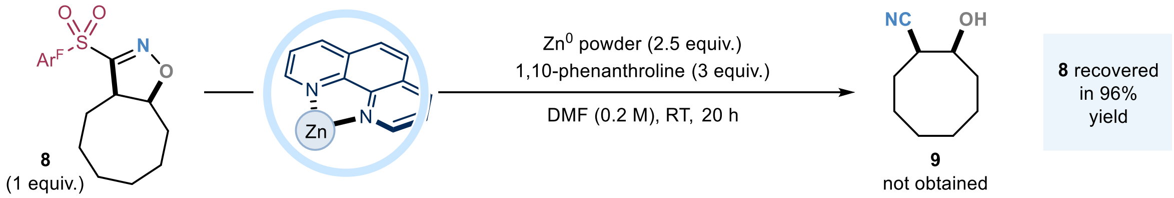

An oven-dried vial was charged with Zn powder (*pre-activated by 1M aq. HCl wash* – 32.7 mg, 0.50 mmol). This was dried under high-vacuum for 10 minutes, while heating with an heat gun, and then back-filled with nitrogen. A solution of isoxazoline **8** (62.3 mg, 0.20 mmol) and 1,10-phenanthroline (108 mg, 0.60 mmol) in anhydrous DMF (1 mL) was added to the vessel. The resulting mixture was stirred at room temperature for 20 hours. The reaction was quenched by addition of water (100 μL) and diluted with EtOAc (1 mL). The crude mixture was extracted with CH_2Cl_2 (3 x 10 mL), and the combined organic portions were dried over Na_2SO_4, and concentrated in vacuo. ^{1}H NMR analysis on the crude reaction mixture (using mesitylene as the internal standard) revealed that no product **9** was formed, and isoxazoline **8** was quantitatively recovered (96% yield). *This reaction was repeated heating the reaction mixture at 60 °C for 20 hours. Even in this case, the starting material **8** was quantitatively recovered and no product was observed.*

S.3.5 – Development of a Telescoped "*Sew & Cut*" Protocol: Capitalizing on the optimization studies described in *Sections S3.1–2*, we implemented two general procedures for the **direct conversion of alkenes into 1,2-*syn*-hydroxy nitriles – via 1,3-DC and telescoped electrolysis of the ensuing cycloadduct:**

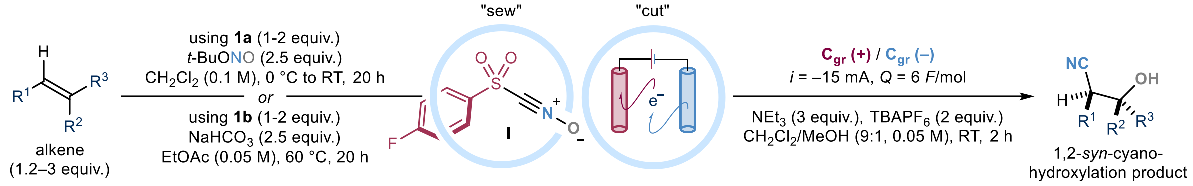

■ **General Procedure D – using 1a:** *Reactions performed on a 0.20 mmol scale, unless reported otherwise*. To a solution of the α-diazo-sulfone **1a** (1-2 equiv., as specified for each entry) and alkene substrate (1-3 equiv.) in CH_2Cl_2 (1 mL), at 0 °C, was slowly added a solution of *t*-BuONO (59.5 μL, 0.50 mmol) in CH_2Cl_2 (1 mL), over 1 hour [*N.B. the use of a syringe pump, to control the rate of addition of t-BuONO, is crucial to achieve high yields and limit the formation of dimerization byproducts*]. Completed the addition, the resulting solution was allowed to warm up to room temperature, and stirred for 20 hours. After this time, the crude reaction mixture was diluted with CH_2Cl_2 (2 mL) and MeOH (0.5 mL), and transferred into a 5 mL IKA ElectraSyn® vial – fitted with carbon graphite microelectrodes (both working and counter electrode) – containing $TBAPF_6$ (155 mg, 0.40 mmol) and Et_3N (83.6 μL, 0.60 mmol). The vial was sealed and mounted on an IKA ElectraSyn® station. The electrolysis was conducted under constant current regime (–15 mA), with a total charge (*Q*) of 6 F/mol. Completed the electrolysis, the crude mixture was concentrated under reduced pressure, and the residue was purified by flash column chromatography on silica gel support (under the conditions reported for each entry) to yield the desired 1,2-*syn*-hydroxy nitrile product.

■ **General Procedure E – using 1b:** *Reactions performed on a 0.20 mmol scale, unless reported otherwise*. To a vigorously stirring solution of $NaHCO_3$ (42.0 mg, 0.50 mmol) and alkene substrate (1.2-3 equiv., as specified for each entry) in EtOAc (4 mL), at room temperature, was added chloroxime **1b** (1-2 equiv.) in one portion. The resulting mixture was then heated to 60 °C, and stirred for 20 hours. After this time, the reaction mixture was allowed to cool down to room temperature and then concentrated under reduced pressure. The crude material was dissolved in a 9:1 mixture of CH_2Cl_2 and MeOH (4 mL), and transferred into a 5 mL IKA ElectraSyn® vial – fitted with carbon graphite microelectrodes (both working and counter electrode) – containing $TBAPF_6$ (155 mg, 0.40 mmol) and the sacrificial reductant [either Et_3N (83.6 μL, 0.60 mmol) or 1,4-cyclohexadiene (66.2 μL, 0.70 mmol)]. The vial was sealed and mounted on an IKA ElectraSyn® station. The electrolysis was conducted under constant current regime (–15 mA), with a total charge (*Q*) of 6 F/mol. Completed the electrolysis, the crude mixture was concentrated under reduced pressure, and the residue was purified by flash column chromatography on silica gel support (under the conditions reported for each entry) to yield the desired 1,2-*syn*-hydroxy nitrile product.

ElectraSyn Commands Workflow: select "*New Experiment*" > select "*Constant Current*" > set current to –15 mA > select "*No*" for reference electrode > select "*Total Charge*" > set 0.20 mmol of substrate and 6 F/mol > select "*No*" for alternating polarity > save the experimental conditions, if desired > select "*Start*" to initiate the electrolysis > set stirring to 1000 rpm.
Useful Information: For the electrodes washing protocol, see *Section S3.2*. In some cases, removal of the remainder/excess olefin from the 1,3-DC crude mixture provided a higher yield for the corresponding 1,2-hydroxy nitrile product. This was often achieved by rapid filtration of the 1,3-DC crude mixture over SiO_2 – flushing first with 15% Et_2O in hexane to remove the remainder alkene and, then, with EtOAc to elute the cycloadduct intermediate.

S.4 – Synthesis of Isoxazoline Cycloadducts

cis-3-((4-fluorophenyl)sulfonyl)-3a,9a-dihydrocycloocta[*d*]isoxazole (8)

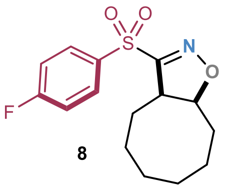

8

Prepared according to ***General Procedure A*** – using α-diazo-sulfone **1a** (45 mg, 0.2 mmol, 1 equiv.) as the nitrile oxide precursor, and *cis*-cyclooctene (87 μL, 0.6 mmol, 3 equiv.) as the olefin substrate. The crude mixture was purified by flash column chromatography (*isocratic in* 30% EtOAc in hexane) to provide isoxazoline **8** (63 mg, 99 % yield) as a colorless solid. Compound **8** was also obtained following ***General Procedure B*** – using chloroxime **1b** (48 mg, 0.2 mmol, 1 equiv.) as the nitrile oxide precursor, and *cis*-cyclooctene (87.0 μL, 0.6 mmol, 3 equiv.) as the olefin substrate. Here, purification of the crude reaction mixture (same conditions as above) delivered **8** (56 mg) in 90% yield. The title compound was previously unknown.

^{1}H NMR (400 MHz, $CDCl_3$) δ 8.05 – 7.98 (m, 2H), 7.30 – 7.23 (m, 2H), 4.62 (ddd, J = 10.0, 8.3, 5.5 Hz, 1H), 3.52 (ddd, J = 10.3, 10.0, 1.4 Hz, 1H), 2.23 – 2.15 (m, 1H), 2.02 – 1.95 (m, 2H), 1.85 – 1.73 (m, 2H), 1.70 – 1.58 (m, 2H), 1.58 – 1.51 (m, 1H), 1.49 – 1.35 (m, 3H), 1.32 – 1.21 (m, 1H). **^{13}C NMR** (126 MHz, $CDCl_3$) δ 166.4 (d, $^1J_{C\text{–}F}$ = 257.9 Hz), 164.5, 134.4 (d, $^4J_{C\text{–}F}$ = 3.2 Hz), 131.9 (d, $^3J_{C\text{–}F}$ = 10.1 Hz), 116.7 (d, $^2J_{C\text{–}F}$ = 22.8 Hz), 89.3, 49.5, 29.5, 25.6, 25.4, 25.2, 25.0, 24.1. **^{19}F NMR** (471 MHz, $CDCl_3$) δ –101.7. **HRMS** (ESI$^+$) Exact mass calculated for $C_{15}H_{19}O_3NFS$ [M+H]$^+$: 312.1064, found: 312.1049.

3,4-bis((4-fluorophenyl)sulfonyl)-1,2,5-oxadiazole 2-oxide (11)

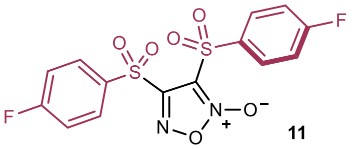

11

During the formation of isoxazoline cycloadducts, under the reaction conditions described both in ***General Procedure A*** and ***B***, variable amounts of furoxan **11** were obtained. This byproduct arises from the dimerization of the sulfonyl-nitrile oxide intermediate (*c.f.* structure **I** in Scheme 2B) formed *in situ* during the course of the cycloaddition process.[11] In multiple occasions, compound **11** has been isolated as a colorless solid, and its spectroscopic properties characterized. These are listed below. The title compound was previously unknown.

^{1}**H NMR** (400 MHz, $CDCl_3$) δ 8.27 – 8.17 (m, 4H), 7.40 – 7.31 (m, 4H). ^{13}**C NMR** (126 MHz, $CDCl_3$, all $\underline{C}_q$ were not detected) δ 133.5 (d, $^3J_{C\text{–}F}$ = 10.3 Hz), 132.5 (d, $^3J_{C\text{–}F}$ = 10.3 Hz), 117.5 (d, $^2J_{C\text{–}F}$ = 22.8 Hz), 117.2 (d, $^2J_{C\text{–}F}$ = 23.1 Hz). ^{19}**F NMR** (376 MHz, $CDCl_3$) δ –97.9, –98.6. **HRMS** (ESI^+) Exact mass calculated for $C_{14}H_8F_2N_2O_6S_2$ $[M+Na]^+$: 401.9792, found: 401.9800.

3-((4-fluorophenyl)sulfonyl)-5-phenyl-4,5-dihydroisoxazole (S-2)

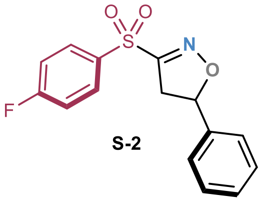

S-2

Prepared according to ***General Procedure B*** – using chloroxime **1b** (48 mg, 0.2 mmol, 1 equiv.) as the nitrile oxide precursor, and styrene (76 µL, 0.6 mmol, 3 equiv.) as the olefin substrate. The crude mixture was purified by flash column chromatography (*isocratic in* 30% EtOAc in hexane) to provide isoxazoline **S-2** (57 mg, 93 % yield) as a colorless solid. The title compound was previously unknown.

^{1}**H NMR** (400 MHz, $CDCl_3$) δ 8.09 – 7.99 (m, 2H), 7.41 – 7.34 (m, 3H), 7.32 – 7.22 (m, 4H), 5.81 (dd, *J* = 11.4, 9.1 Hz, 1H), 3.75 (dd, *J* = 17.4, 11.4 Hz, 1H), 3.34 (dd, *J* = 17.4, 9.1 Hz, 1H). ^{13}**C NMR** (101 MHz, $CDCl_3$) δ 166.6 (d, $^1J_{C\text{–}F}$ = 258.6 Hz), 159.8, 138.3, 133.3 (d, $^4J_{C\text{–}F}$ = 3.3 Hz), 132.1 (d, $^3J_{C\text{–}F}$ = 10.0 Hz), 129.2, 129.1, 126.0, 117.0 (d, $^2J_{C\text{–}F}$ = 22.9 Hz), 86.2, 40.1. ^{19}**F NMR** (376 MHz, $CDCl_3$) δ –100.87. **HRMS** (ESI^+) Exact mass calculated for $C_{15}H_{12}O_3NFNaS$ $[M+Na]^+$: 328.0414, found: 328.0406.

***cis*-3-(phenylsulfonyl)-3a,9a- dihydrocycloocta[*d*]isoxazole (S-3)**

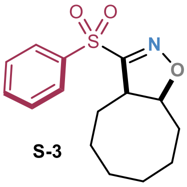

S-3

Prepared according to ***General Procedure A*** – using α-diazo-sulfone **1c** (136 mg, 0.6 mmol, 1 equiv.) as the nitrile oxide precursor, and *cis*-cyclooctene (260 µL, 1.8 mmol, 3 equiv.) as the olefin substrate. The crude mixture was purified by flash column chromatography (*isocratic in* 40% EtOAc in hexane) to provide isoxazoline **S-3** (175 mg, 99 % yield) as a yellow oil. The title compound was previously unknown.

^{1}**H NMR** (400 MHz, $CDCl_3$) δ 8.03 – 7.96 (m, 2H), 7.73 – 7.66 (m, 1H), 7.63 – 7.55 (m, 2H), 4.60 (ddd, *J* = 10.0, 8.4, 5.7 Hz, 1H), 3.50 (app td, *J* = 10.3, 1.3 Hz, 1H), 2.23 – 2.12 (m, 1H), 2.01 – 1.93 (m, 2H), 1.81 – 1.72 (m, 2H), 1.68 – 1.60 (m, 2H), 1.57 – 1.51 (m, 1H), 1.48 – 1.41 (m, 1H), 1.41 – 1.33 (m, 2H), 1.33 – 1.27 (m, 1H). ^{13}**C NMR** (101 MHz, $CDCl_3$) δ 164.5, 138.5, 134.6, 129.3, 128.9, 89.2, 49.6, 29.5, 25.6, 25.4, 25.3, 25.0, 24.1. **HRMS** (ESI^+) Exact mass calculated for $C_{15}H_{20}O_3NS$ $[M+H]^+$: 294.1158, found: 294.1145.

***cis*-3-bromo-3a,4,5,6,7,8,9,9a-octahydrocycloocta[*d*]isoxazole (12)**

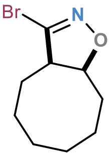

12

Prepared according to ***General Procedure B*** – using 1,1-dibromoformaldoxime (811 mg, 4.0 mmol, 1 equiv.) as the nitrile oxide precursor, and *cis*-cyclooctene (1.64 mL, 12 mmol, 3 equiv.) as the olefin substrate. The crude mixture was purified by flash column chromatography (*isocratic in* 30% EtOAc in hexane)

to provide isoxazoline **12** (687 mg, 74 % yield) as a colorless solid. The title compound was previously unknown.

^{1}**H NMR** (500 MHz, $CDCl_3$) δ 4.59 (td, *J* = 10.1, 3.4 Hz, 1H), 3.08 (app t, *J* = 10.2 Hz, 1H), 2.03 – 1.94 (m, 2H), 1.89 – 1.77 (m, 2H), 1.73 – 1.51 (m, 4H), 1.51 – 1.38 (m, 2H), 1.34 – 1.28 (m, 1H), 1.24 – 1.19 (m, 1H). ^{13}**C NMR** (126 MHz, $CDCl_3$) δ 144.6, 85.5, 55.6, 29.5, 26.3, 25.8, 25.4, 25.1, 24.0.

cis-ethyl 3a,4,5,6,7,8,9,9a-octahydrocycloocta[*d*]isoxazole-3-carboxylate (13)

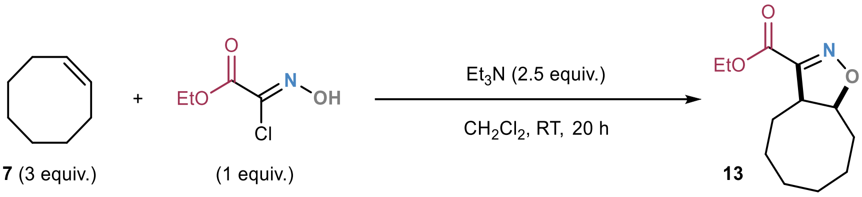

7 (3 equiv.) (1 equiv.) **13**

To a stirring solution of ethyl chlorooximidoacetate (1.94 g, 12.8 mmol) and *cis*-cyclooctene **7** (5.0 mL, 38.4 mmol) in CH_2Cl_2 (110 mL), at room temperature, was added a solution of Et_3N (4.46 mL, 32 mmol) in CH_2Cl_2 (20 mL), dropwise over an hour. The resulting mixture was stirred at room temperature for 20 hours. After this time, the crude mixture was concentrated under reduced pressure, and the residue was purified by flash column chromatography (*isocratic in* 30% EtOAc in hexane) to provide isoxazoline **13** (2.24 g, 78 % yield) as a colorless oil. The spectroscopic properties of this compound were consistent with the data available in the literature.[12]

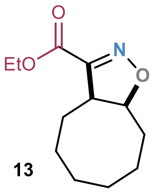

13

^{1}**H NMR** (400 MHz, $CDCl_3$) δ 4.57 (ddd, *J* = 13.0, 10.1, 3.1 Hz, 1H), 4.34 (q, *J* = 7.1 Hz, 2H), 3.29 (app t, *J* = 9.9 Hz, 1H), 2.12 – 1.91 (m, 2H), 1.82 – 1.73 (m, 2H), 1.72 – 1.60 (m, 4H), 1.55 – 1.43 (m, 1H), 1.40 – 1.31 (m, 5H), 1.30 – 1.19 (m, 1H). ^{13}**C NMR** (101 MHz, $CDCl_3$) δ 160.9, 156.3, 88.1, 61.9, 49.0, 29.8, 25.6, 25.4, 25.1, 25.1, 24.6, 14.1.

cis-3-(2-phenylpropan-2-yl)-3a,4,5,6,7,8,9,9a-octahydrocycloocta[*d*]isoxazole (14)

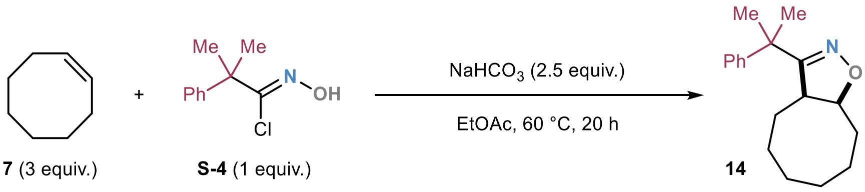

7 (3 equiv.) **S-4** (1 equiv.) **14**

For the synthesis of **14**, we prepared chloroxime **S-4** – from 2-methyl-2-phenylpropanal – following a reported literature protocol.[13] The procedure delivered a pale yellow solid (isolated as a mixture of *E* and *Z* geometric isomers), for which we recorded the following spectroscopic properties: ^{1}**H NMR** (400 MHz, $CDCl_3$) δ 8.47 (s, 1H), 7.44 – 7.35 (m, 3H), 7.34 – 7.27 (m, 2H), 1.74 (s, 3H), 1.66 (s, 3H). ^{13}**C NMR** (101 MHz, $CDCl_3$) δ 148.9 (2 signals), 144.3, 142.7, 129.0, 128.5, 127.8, 127.0, 126.0, 125.0, 60.6, 47.6, 39.2, 30.2, 27.6 (2 signals), 21.1, 14.2.

Isoxazoline **14** was prepared according to ***General Procedure B*** – using chloroxime **S-4** (226 mg, 1 mmol, 1 equiv.) as the nitrile oxide precursor, and *cis*-cyclooctene (650 µL, 5 mmol, 5 equiv.) as the olefin substrate. The crude mixture was purified by flash column

chromatography (*isocratic in* 20% EtOAc in hexane) to provide isoxazoline **14** (171 mg, 63 % yield) as a colorless solid. The title compound was previously unknown.

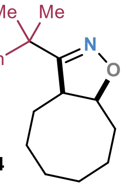

14

^{1}H NMR (400 MHz, $CDCl_3$) δ 7.43 – 7.37 (m, 2H), 7.36 – 7.29 (m, 2H), 7.28 – 7.20 (m, 1H), 4.37 (ddd, *J* = 10.6, 9.4, 3.2 Hz, 1H), 2.89 (td, *J* = 9.8, 1.4 Hz, 1H), 1.92 – 1.85 (m, 1H), 1.80 – 1.71 (m, 1H), 1.63 (s, 3H), 1.60 (s, 3H), 1.45 – 1.29 (m, 8H), 1.29 – 1.20 (m, 1H), 1.07 (ddt, *J* = 15.2, 4.2, 1.8 Hz, 1H). **^{13}C NMR** (101 MHz, $CDCl_3$) δ 168.1, 146.1, 128.4, 126.5, 125.8, 85.3, 51.3, 40.5, 30.3, 29.6, 26.8, 26.5, 26.2, 25.3, 25.2, 23.0. **HRMS** (ESI$^+$) Exact mass calculated for $C_{18}H_{25}ONNa$ [M+Na]$^+$: 294.1828, found: 294.1825.

cis-3a,4,5,6,7,8,9,9a-octahydrocycloocta[*d*]isoxazole-3-carboxylic acid (15)

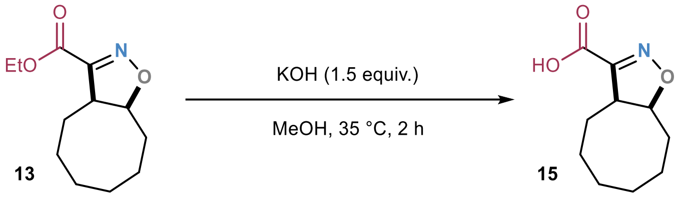

A mixture of ester **13** (1.35 g, 6.0 mmol) and KOH (505 mg, 9.0 mmol) in MeOH (24 mL) was stirred at 35 °C for 2 hours. After this time, the mixture was allowed to cool down to room temperature, and partitioned between water (25 mL) and CH_2Cl_2 (25 mL). The aqueous phase was collected and acidified by addition of an *aq.* HCl 1M solution, until pH ~2. This solution was then re-extracted with CH_2Cl_2 (3 x 20 mL), and the combined organic portions were washed with brine, dried over $MgSO_4$ and filtered. Evaporation of the solvent under reduced pressure delivered isoxazoline **15** (808 mg, 68 % yield) as a colorless oil, in high purity. *This material was used without any further purification procedure both in the electrochemical ring-opening reaction (see Section 5.3.4) and in the synthesis of compound **16***. The title compound was previously unknown.

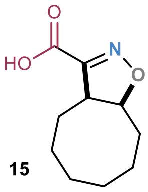

15

^{1}H NMR (400 MHz, $CDCl_3$) δ 6.61 (s, 1H), 4.66 (ddd, *J* = 11.3, 10.2, 3.2 Hz, 1H), 3.36 – 3.26 (m, 1H), 2.14 – 1.94 (m, 2H), 1.88 – 1.58 (m, 6H), 1.49 (ddt, *J* = 12.3, 9.2, 4.6 Hz, 1H), 1.41 – 1.31 (m, 2H), 1.31 – 1.16 (m, 1H). **^{13}C NMR** (126 MHz, DMSO-d_6) δ 162.2, 157.4, 87.4, 48.8, 29.8, 25.5, 25.4, 25.2, 24.9, 24.4. **HRMS** (ESI$^+$) Exact mass calculated for $C_{10}H_{14}O_3NNa$ [M-H]$^+$: 196.0979, found: 196.0974.

cis-phthalimidyl 3a,4,5,6,7,8,9,9a-octahydrocycloocta[*d*]isoxazole-3-carboxylate (16)

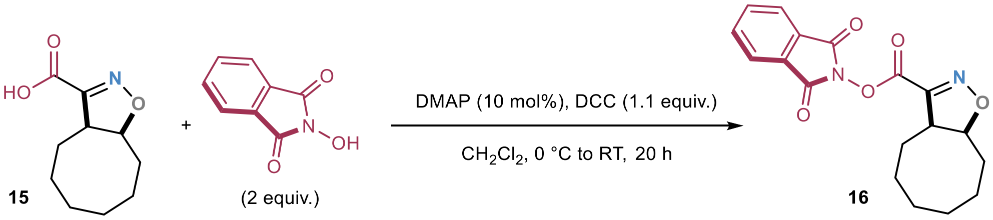

To a stirring solution of carboxylic acid **15** (296 mg, 1.5 mmol), 4-(dimethylamino)pyridine (DMAP, 20 mg, 0.15 mmol) and *N*-hydroxyphthalimide (489 mg, 3.0 mmol) in anhydrous CH_2Cl_2 (1.5 mL), at 0 °C and under nitrogen atmosphere, was added dropwise a solution of

N,*N*'-dicyclohexylcarbodiimide (DCC, 340 mg, 1.65 mmol) in CH_2Cl_2 (0.5 mL). The resulting mixture was allowed to warm up to room temperature, and stirred for 20 hours. After this time, the crude mixture was quenched by addition of water (10 mL), and extracted with CH_2Cl_2 (3 x 20 mL). The combined organic portions were washed with water, dried over $MgSO_4$, filtered and the solvent removed in vacuo. The residue was purified by flash column chromatography (*isocratic in* 40% EtOAc in hexane) to provide isoxazoline **16** (382 mg, 74 % yield) as a colorless oil. The title compound was previously unknown.

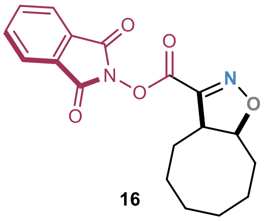
16

^{1}H NMR (400 MHz, $CDCl_3$) δ 7.92 (dd, *J* = 5.5, 3.1 Hz, 2H), 7.82 (dd, *J* = 5.5, 3.1 Hz, 2H), 4.72 (ddd, *J* = 11.5, 10.2, 2.8 Hz, 1H), 3.41 (td, *J* = 10.2, 1.5 Hz, 1H), 2.19 – 2.01 (m, 2H), 1.94 – 1.84 (m, 1H), 1.83 – 1.74 (m, 2H), 1.74 – 1.64 (m, 3H), 1.56 – 1.46 (m, 1H), 1.38 – 1.23 (m, 3H). **^{13}C NMR** (126 MHz, DMSO-d_6) δ 162.0, 157.9, 153.2, 136.2, 128.6, 124.7, 89.4, 48.1, 29.5, 25.5, 25.2, 25.1, 24.8, 24.4. **HRMS** (ESI$^+$) Exact mass calculated for $C_{18}H_{18}O_5N_2Na$ [M+Na]$^+$: 365.1108, found: 365.1100.

cis-4-((4-fluorophenyl)sulfonyl)-6,6-diphenyl-2-oxa-3-azabicyclo[*3.1.0*]hex-3-ene (18)

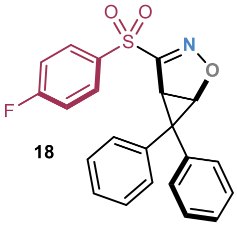
18

Prepared according to ***General Procedure B*** – using chloroxime **1b** (380 mg, 1.6 mmol, 1 equiv.) as the nitrile oxide precursor, and cycloprop-2-ene-1,1-diyldibenzene **17** (369 mg, 1.92 mmol, 1.2 equiv.) as the olefin substrate. The crude mixture was purified by flash column chromatography (*isocratic in* 30% EtOAc in hexane) to provide isoxazoline **18** (559 mg, 89 % yield) as a colorless solid. The title compound was previously unknown.

^{1}H NMR (500 MHz, $CDCl_3$) δ 7.83 – 7.76 (m, 2H), 7.40 – 7.32 (m, 2H), 7.31 – 7.20 (m, 6H), 7.20 – 7.14 (m, 2H), 7.08 – 7.02 (m, 2H), 5.48 (d, *J* = 5.5 Hz, 1H), 3.91 (d, *J* = 5.5 Hz, 1H). **^{13}C NMR** (126 MHz, $CDCl_3$) δ 166.5 (d, $^1J_{C-F}$ = 258.5 Hz), 162.6, 140.5, 133.3, 133.2 (d, $^4J_{C-F}$ = 2.4 Hz), 132.1 (d, $^3J_{C-F}$ = 10.1 Hz), 131.0, 128.9, 28.7, 128.0, 127.3, 126.9, 116.7 (d, $^2J_{C-F}$ = 22.9 Hz), 77.7, 40.9, 30.6. **^{19}F NMR** (471 MHz, $CDCl_3$) δ –101.2. **HRMS** (ESI$^+$) Exact mass calculated for $C_{22}H_{16}O_3NFNaS$ [M+Na]$^+$: 416.0727, found: 416.0715.

3-((4-fluorophenyl)sulfonyl)-5-phenyl-5-(trimethylsilyl)-4,5-dihydroisoxazole (23)

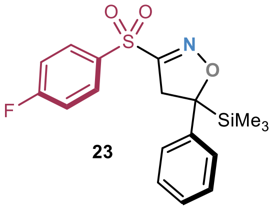
23

Prepared according to ***General Procedure B*** – using chloroxime **1b** (536 mg, 1.5 mmol, 1 equiv.) as the nitrile oxide precursor, and trimethyl(1-phenylvinyl)silane (793 mg, 4.5 mmol, 3 equiv.) as the olefin substrate. The crude mixture was purified by flash column chromatography (*isocratic in* 10% EtOAc in hexane) to provide isoxazoline **23** (333 mg, 59% yield) as a pale yellow oil. The title compound was previously unknown.

^{1}H NMR (500 MHz, $CDCl_3$) δ 7.99 – 7.90 (m, 2H), 7.34 – 7.28 (m, 2H), 7.25 – 7.19 (m, 3H), 7.18 – 7.12 (m, 2H), 3.62 (s, 2H), 0.01 (s, 9H). **^{13}C NMR** (126 MHz, $CDCl_3$) δ 166.4 (d, $^1J_{C-F}$ = 258.2 Hz), 159.3, 142.6, 133.5 (d, $^4J_{C-F}$ = 3.1 Hz), 131.9 (d, $^3J_{C-F}$ = 10.1 Hz), 128.3, 126.8, 123.8, 116.8 (d, $^2J_{C-F}$ = 22.9 Hz), 90.0, 42.0, –4.6. **^{19}F NMR** (471 MHz, $CDCl_3$) δ –101.4. **HRMS** (ESI^+) Exact mass calculated for $C_{18}H_{20}O_3NFNaSSi$ $[M+Na]^+$: 400.0809, found: 400.0799.

S.5 – Mechanistic Investigations

S.5.1 – Total Charge (Q) Dosage Experiment (*Scheme 3A*): For this study, we performed the 1,3-dipolar cycloaddition reaction between α-diazo-sulfone **1a** and *cis*-cyclooctene **7** (following ***General Procedure A***) on a 1.6 mmol scale. The experiment was conducted using 834 μL of **7** (1.60 mmol, 1 equiv.), 727 mg of **1a** (3.20 mmol, 2 equiv.), 867 μL of *t*-BuONO (5.60 mmol, 3.5 equiv.) and 16 mL of CH_2Cl_2.

At the end of the reaction, the crude reaction mixture was divided in ***eight aliquots of 2 mL each***. In this way, each aliquot contained same amount of products and/or unreacted starting materials in ~ 0.20 mmol scale. ***One aliquot*** was analyzed by ^{1}H NMR (using mesitylene as the internal standard) and revealed that isoxazoline **8** was formed in 83% yield, and furoxan byproduct **11** in 80% yield. Whereas, substrates **1a** and **7** were not detected. ***Six of the remainder aliquots*** were diluted with CH_2Cl_2 (2 mL) and MeOH (0.5 mL), and submitted to the optimized electrochemical conditions (as described in ***General Procedure C***); varying the applied total charge (*Q*). Specifically, for the electrolysis of the first aliquot a Q = 1 F/mol was applied, while the last one was conducted at a Q = 6 F/mol; with intervals of 1 F/mol between each run. At the end of each electrolysis, the reaction mixture was concentrated under reduced pressure, and the crude material was analyzed by ^{1}H NMR (using mesitylene as the internal standard) – in order to quantify the amount of hydroxy nitrile product **9** formed, and the consumption of isoxazoline **8** and furoxan **11**.

The results of this study are depicted in the graph below (next page). These indicates that the first equimolar amount of electrons is consumed to ensure the complete degradation of furoxan **11**. After this, the electrolysis of **8** requires further 5 *F*/mol to reach completion – presumably, due to additional charge dissipated by the decomposition of fragmentation byproducts into volatile compounds.

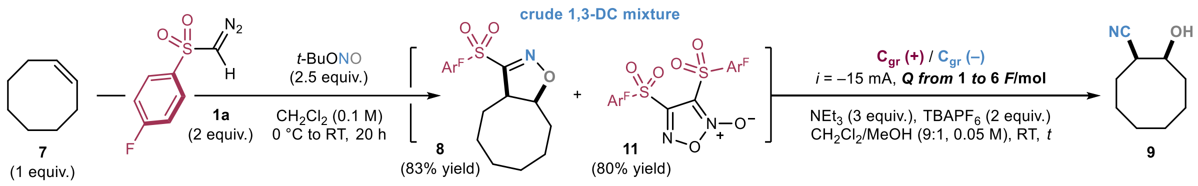

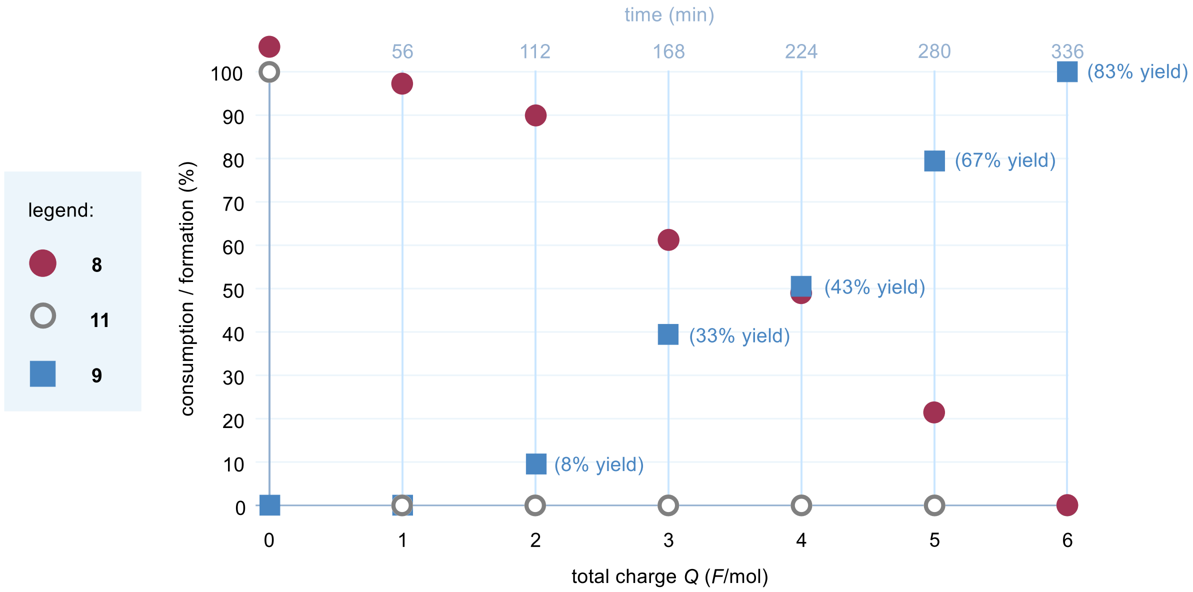

S.5.2 – Voltammetric Analysis

S.5.2.1 – *Electrochemical Characterization of the Reaction Components (Scheme 2C):*

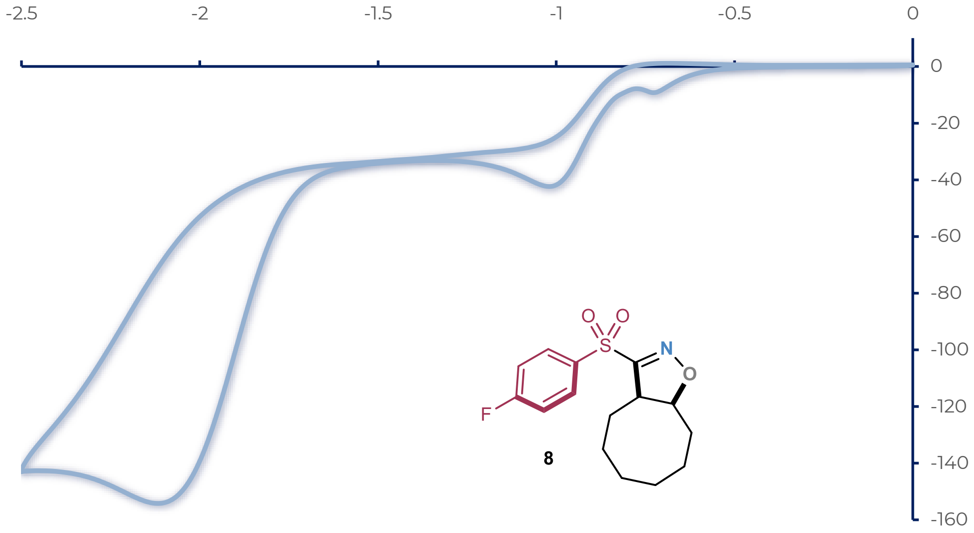

Cyclic voltammogram for isoxazoline **8** [0.005 M] in [0.1 M] $TBAPF_6$ in CH_3CN. Sweep rate: 25 mV/s. Glassy carbon working electrode, Ag/AgCl (NaCl saturated) reference electrode, Pt wire auxiliary electrode. Irreversible reduction, E_p^C = E(**8**/**8**$^-$) = –1.01 V, where E_p^C refers to the cathodic peak potential, while the E value describes the electrochemical properties of **8**.

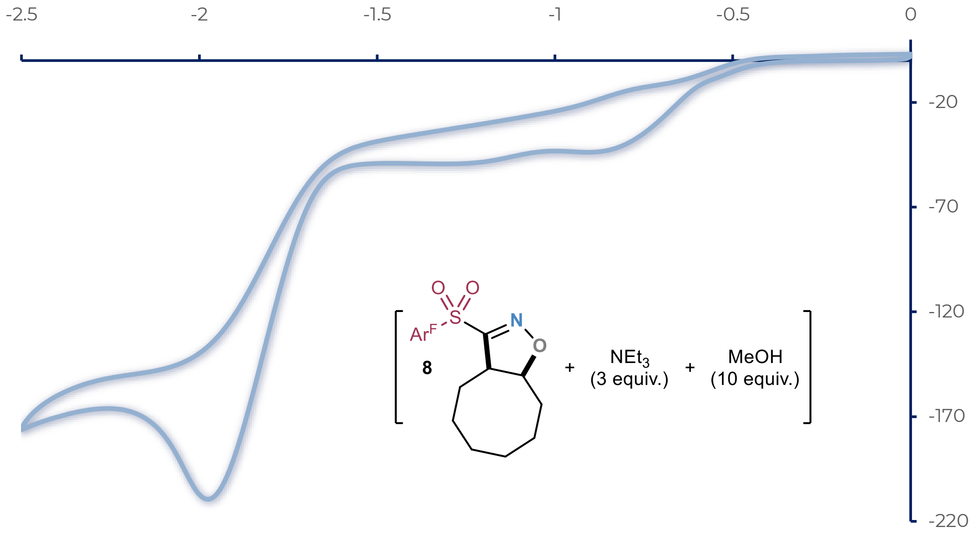

Cyclic voltammogram for a mixture of isoxazoline **8** [0.005 M], NEt_3 [0.015 M] and MeOH [0.05 M] in [0.1 M] $TBAPF_6$ in CH_3CN. Sweep rate: 25 mV/s. Glassy carbon working electrode, Ag/AgCl (NaCl sat.) reference electrode, Pt wire auxiliary electrode. Irreversible reduction, E_p^C = E(**8**/**8**$^{•-}$) = –0.85 V, where E_p^C refers to the cathodic peak potential, while the E value describes the electrochemical properties of **8** in the presence of NEt_3 and MeOH.

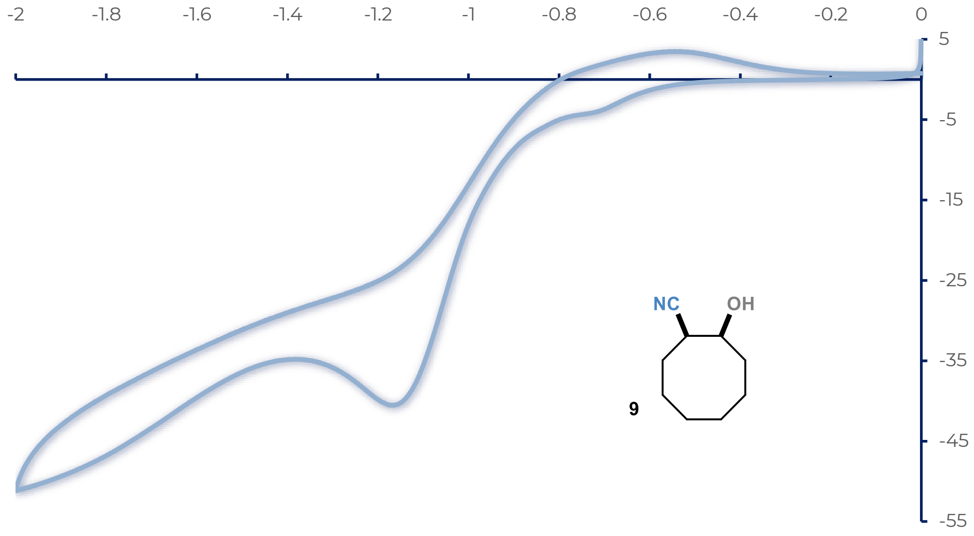

Cyclic voltammogram for 1,2-*syn*-hydroxy-nitrile product **9** [0.01 M] in [0.1 M] $TBAPF_6$ in CH_3CN. Sweep rate: 50 mV/s. Glassy carbon working electrode, Ag/AgCl (NaCl sat.) reference electrode, Pt wire auxiliary electrode. Irreversible reduction, E_p^C = E(**9**/**9**$^{•-}$) = –1.16 V, where E_p^C refers to the cathodic peak potential, while the E value describes the electrochemical properties of **9**.

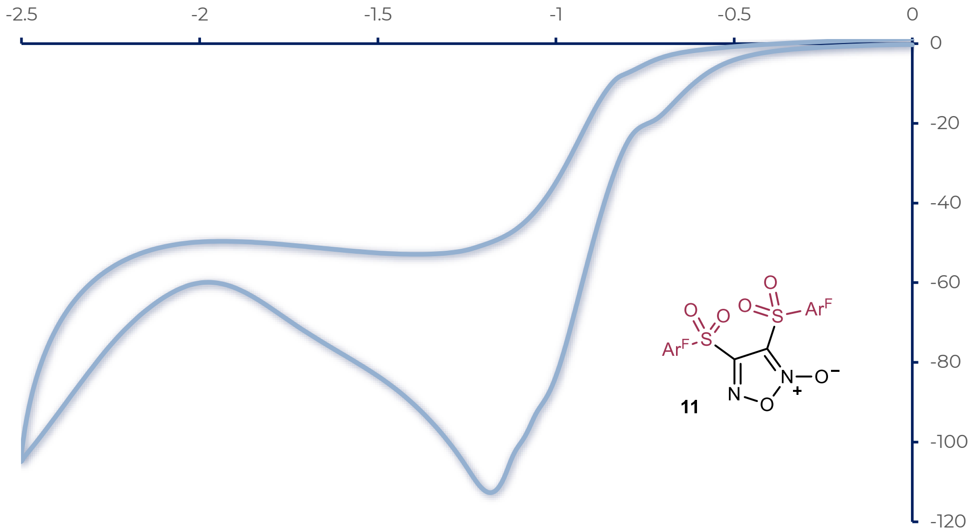

Cyclic voltammogram for furoxan **11** [0.01 M] in [0.1 M] TBAPF$_6$ in CH$_3$CN. Sweep rate: 50 mV/s. Glassy carbon working electrode, Ag/AgCl (NaCl sat.) reference electrode, Pt wire auxiliary electrode. Irreversible reduction, E_p^C = E(**11**/**11**$^{\bullet-}$) = –1.18 V, where E_p^C refers to the cathodic peak potential, while the E value describes the electrochemical properties of **11**.

S.5.2.2 – *Electrochemical Characterization of Isoxazolines* ***12-16*** *(Scheme 3C):*

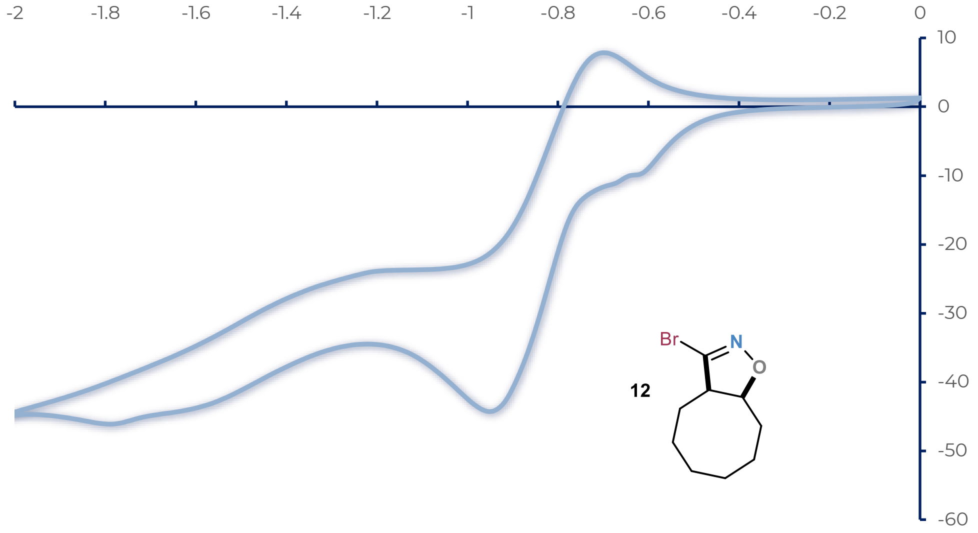

Cyclic voltammogram for isoxazoline **12** [0.005 M] in [0.1 M] TBAPF$_6$ in CH$_3$CN. Sweep rate: 25 mV/s. Glassy carbon working electrode, Ag/AgCl (NaCl sat.) reference electrode, Pt wire auxiliary electrode. Irreversible reduction, E_p^C = E(**12**/**12**$^{\bullet-}$) = –0.93 V, where E_p^C refers to the cathodic peak potential, while the E value describes the electrochemical properties of **12**.

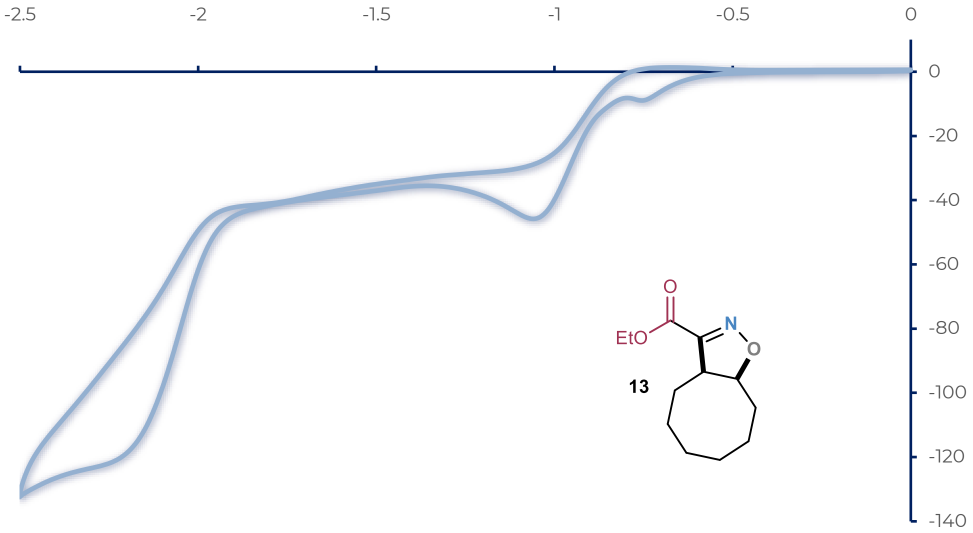

Cyclic voltammogram for isoxazoline **13** [0.005 M] in [0.1 M] $TBAPF_6$ in CH_3CN. Sweep rate: 25 mV/s. Glassy carbon working electrode, Ag/AgCl (NaCl sat.) reference electrode, Pt wire auxiliary electrode. Irreversible reduction, E_p^C = E(**13**/**13**$^{\bullet-}$) = –1.04 V, where E_p^C refers to the cathodic peak potential, while the E value describes the electrochemical properties of **13**.

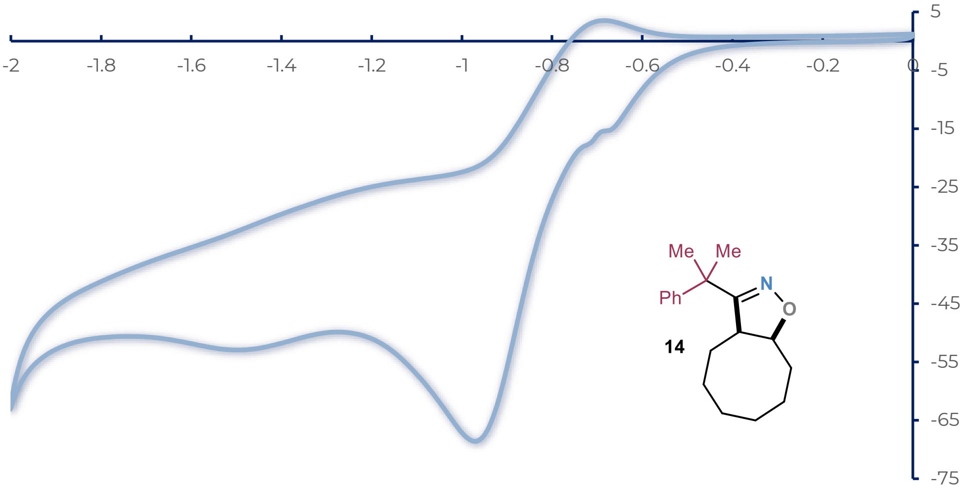

Cyclic voltammogram for isoxazoline **14** [0.005 M] in [0.1 M] $TBAPF_6$ in CH_3CN. Sweep rate: 50 mV/s. Glassy carbon working electrode, Ag/AgCl (NaCl sat.) reference electrode, Pt wire auxiliary electrode. Irreversible reduction, E_p^C = E(**14**/**14**$^{\bullet-}$) = –0.96 V, where E_p^C refers to the cathodic peak potential, while the E value describes the electrochemical properties of **14**.

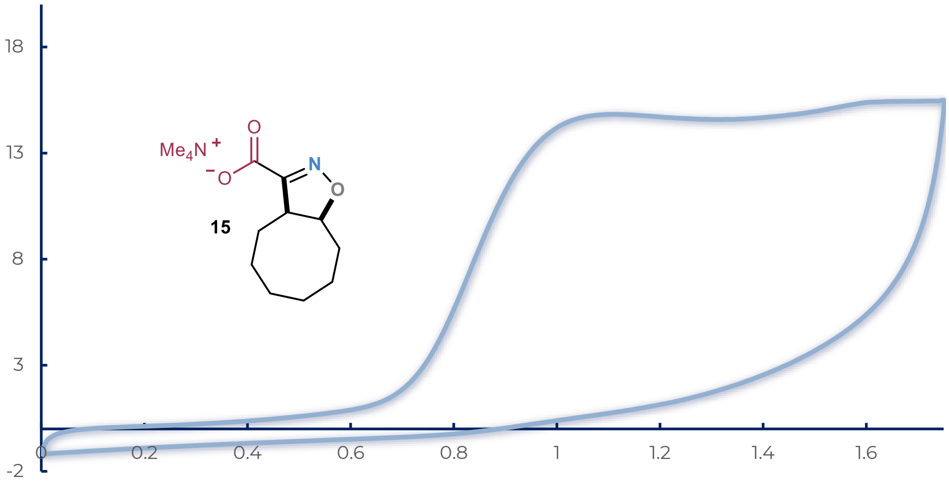

Cyclic voltammogram for isoxazoline **15** [0.005 M] in [0.1 M] $TBAPF_6$ in CH_3CN. Sweep rate: 50 mV/s. Glassy carbon working electrode, Ag/AgCl (NaCl sat.) reference electrode, Pt wire auxiliary electrode. Irreversible oxidation, E_p^A = E(**15**/**15**$^{\cdot+}$) = +1.08 V, where E_p^A refers to the anodic peak potential, while the E value describes the electrochemical properties of **15**.

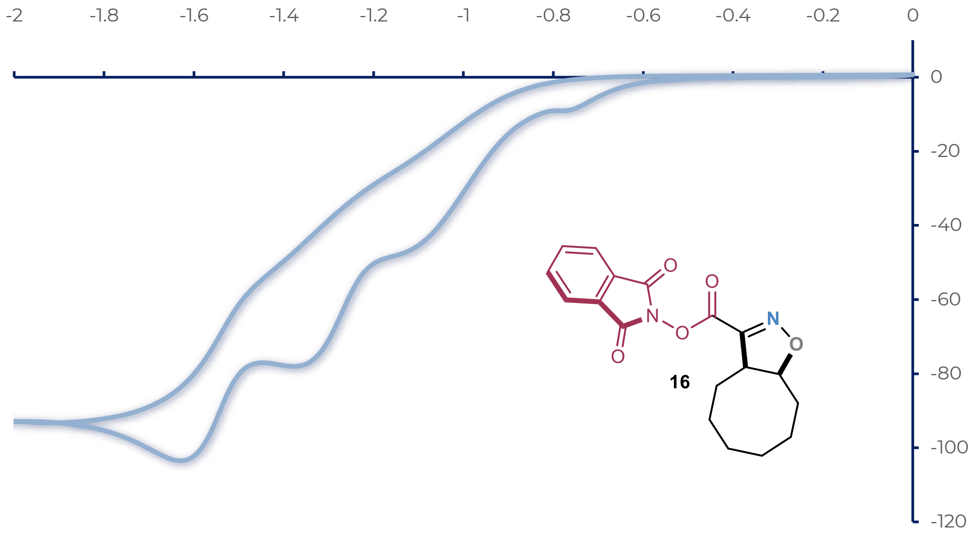

Cyclic voltammogram for isoxazoline **16** [0.005 M] in [0.1 M] $TBAPF_6$ in CH_3CN. Sweep rate: 25 mV/s. Glassy carbon working electrode, Ag/AgCl (NaCl sat.) reference electrode, Pt wire auxiliary electrode. Irreversible reduction, E_p^C = E(**16**/**16**$^{\cdot-}$) = –1.10 V, where E_p^C refers to the cathodic peak potential, while the E value describes the electrochemical properties of **16**. A second irreversible reduction peak is observed at E^{red} = –1.35 V.

S.5.3 – Influence of C3-Substitution on the Electrochemical Radical Ring-Opening of Isoxazoline Cycloadducts (*Scheme 3C*)

S.5.3.1 – *Electrolysis of C3-Phenylsulfonyl-Isoxazoline **S-3**:*

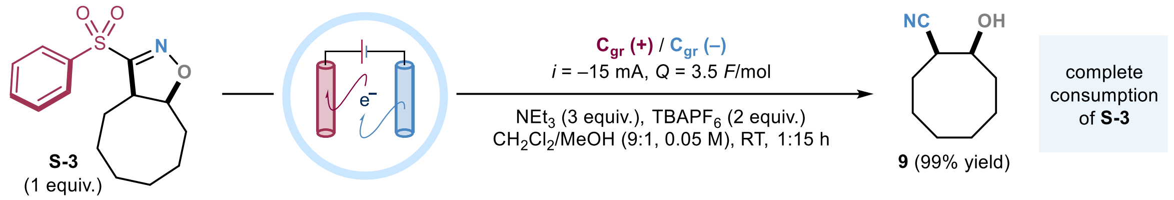

This experiment was conducted following ***General Procedure C*** – using isoxazoline **S-3** (67.5 mg, 0.20 mmol) as the substrate. The crude reaction mixture was concentrated under reduced pressure and analyzed by ^{1}H NMR (using mesitylene as internal standard). Hydroxy nitrile product **9** was observed in quantitative yield, while isoxazoline **S-3** was not observed. Isolation of product **9** by flash column chromatography (*isocratic in* 60% Et_2O in hexane) confirmed the yield of 99%.

Discussion: The synthesis of **S-3** (*Section S.4*) and the result reported above suggest that the presence of a fluorine atom on the aromatic portion of sulfones **1a-b** is not required to enable the occurrence of either the 1,3-DC reactivity or the ensuing electrochemical isoxazoline ring-opening step. Nevertheless – as reported in *Section S.2.2.3* – compared to the synthesis of **1a**, the route to **1c** is less efficient and requires chromatographic purification, after the first step, in order to obtain diazo-compound intermediate **S-1** in high purity. Nevertheless, no difference in stability between **1a** and **1c** was observed. At the outset of our investigations, we have selected the *p*-fluorophenyl-sulfonyl group as privileged redox handle, since previous reports described its use as redox active moiety to produce C(sp^3)-centered radicals, under SET reductive conditions.[14] Alternative redox-active sulfonyl moieties were trialed (*i.e.* bearing pentafluorophenyl, benzothiazolyl or 1-phenyl-1*H*-tetrazolyl rings; instead of *p*-fluorophenyl), but the synthesis of the corresponding reagents **1** proved either unfeasible or extremely inefficient. Furthermore, the presence of a fluorine atom on the aryl-sulfonyl moiety of reagents **1** turned out to be a precious handle for reaction monitoring. In several cases, we ascertained the successful formation of the isoxazoline cycloadduct by submitting the crude 1,3-DC reaction mixture to both ^{1}H and ^{19}F NMR analyses.

S.5.3.2 – *Electrolysis of C3-Bromo-Isoxazoline **12**:*

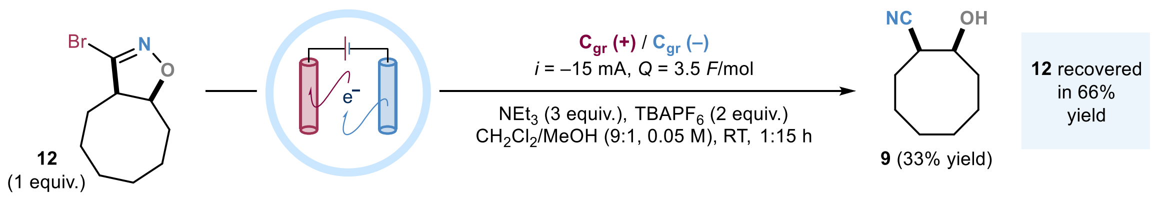

This experiment was conducted following ***General Procedure C*** – using isoxazoline **12** (46.4 mg, 0.20 mmol) as the substrate. The crude reaction mixture was concentrated under

reduced pressure and analyzed by ^{1}H NMR (using mesitylene as internal standard). Hydroxy nitrile product **9** was observed in 33% yield, while isoxazoline **12** was recovered in 66% yield. Isolation of product **9** by flash column chromatography (*isocratic in* 60% Et_2O in hexane) confirmed the yield of 33%.

S.5.3.3 – *Electrolysis of C3-Ethyl Ester-Isoxazoline **13**:*

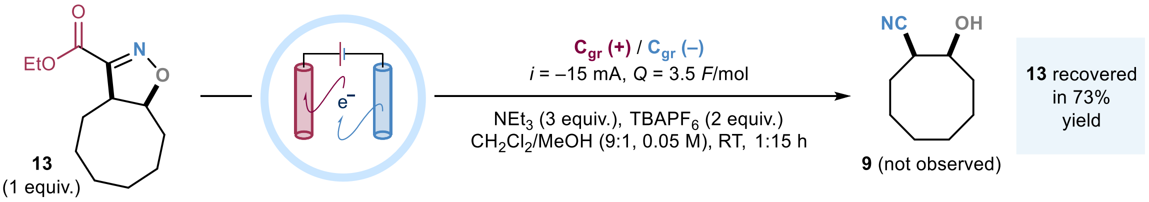

This experiment was conducted following ***General Procedure C*** – using isoxazoline **13** (45.1 mg, 0.20 mmol) as the substrate. The crude reaction mixture was concentrated under reduced pressure and analyzed by ^{1}H NMR (using mesitylene as internal standard). In this case, hydroxy nitrile product **9** was not detected, whereas isoxazoline **13** was observed in 73% yield. The electrochemical reaction delivered no other product – since the crude ^{1}H NMR spectrum contained only **13** and solvent peaks.

S.5.3.4 – *Electrolysis of C3-Alkyl-Isoxazoline **14**:*

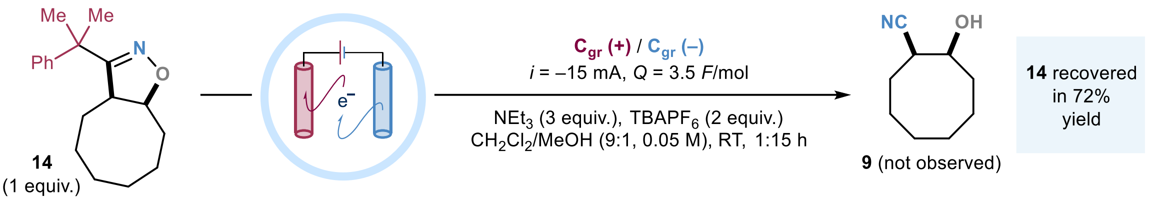

This experiment was conducted following ***General Procedure C*** – using isoxazoline **14** (65.1 mg, 0.20 mmol) as the substrate. The crude reaction mixture was concentrated under reduced pressure and analyzed by ^{1}H NMR (using mesitylene as internal standard). Even in this case, hydroxy nitrile product **9** was not detected, and isoxazoline **13** was recovered in 72% yield. The electrochemical reaction delivered no other product – since the crude ^{1}H NMR spectrum contained only **14** and solvent peaks.

S.5.3.5 – *Electrolysis of C3-Carboxyl-Isoxazoline **15**:*

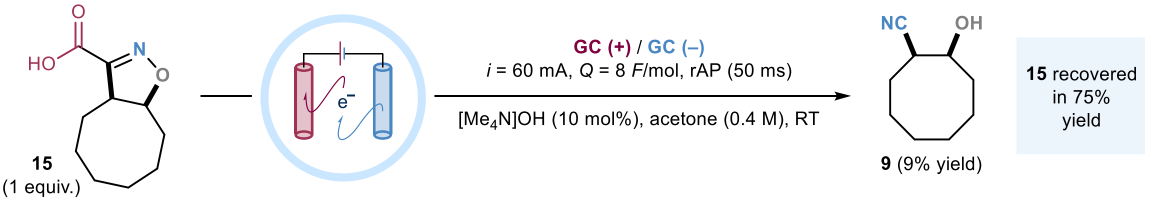

This experiment was conducted following a modified literature procedure.[15] A 2 mL IKA ElectraSyn® vial – fitted with glassy carbon microelectrodes (both working and counter electrode) – was charged with isoxazoline **15** (291 mg, 0.85 mmol), $[Me_4N]OH$ (31.0 mg, 85.0 μmol) and acetone (2 mL). The vial was sealed, mounted on an IKA ElectraSyn® station, and

spurged with nitrogen for 5 minutes. Maintaining a positive nitrogen atmosphere, through balloon pressure, the electrolysis was conducted under constant current regime (–15 mA), and rapid alternating polarity [rAP frequency: 50 ms (10Hz)], applying a total charge (*Q*) of 8 F/mol. During the electrolysis, a water bath was used to keep constant the reaction temperature. Completed the reaction, the electrodes were rinsed into the vial using EtOAc, and the crude mixture was concentrated under reduced pressure. The crude reaction mixture was analyzed by ^{1}H NMR (using mesitylene as internal standard), revealing the presence of hydroxy nitrile product **9** in 9% yield. The only other product detected in the ^{1}H NMR spectrum was isoxazoline **15**, observed in 75% yield.

S.5.3.6 – *Electrolysis of C3-NHPI Ester-Isoxazoline **16**:*

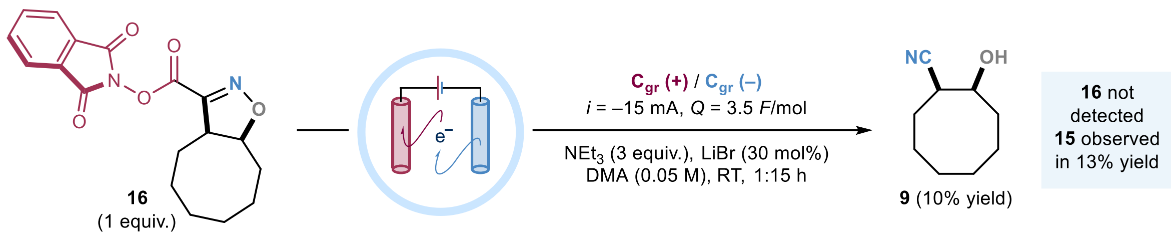

This experiment was conducted following a modified literature procedure.[16] A 5 mL IKA ElectraSyn® vial – fitted with carbon graphite microelectrodes (both working and counter electrode) – was charged with isoxazoline **16** (68.5 mg, 0.20 mmol), LiBr (5.20 mg, 60.0 μmol) and DMA (4 mL). The vial was sealed, mounted on an IKA ElectraSyn® station, and spurged with nitrogen for 5 minutes. After this time, anhydrous NEt_3 (83.6 μL, 0.60 mmol) was added to the vessel. Maintaining a positive nitrogen atmosphere, through balloon pressure, the electrolysis was conducted under constant current regime (–15 mA), and applying a total charge (*Q*) of 3.5 F/mol. Completed the electrolysis, the electrodes were rinsed into the vial using EtOAc, and the crude mixture was concentrated under reduced pressure. The crude reaction mixture was analyzed by ^{1}H NMR (using mesitylene as internal standard), revealing the presence of hydroxy nitrile product **9** in 10% yield. Whereas, isoxazoline **16** was not detected. The several peaks observed in the ^{1}H NMR spectrum of the crude mixture indicated that **16** underwent decomposition under the reaction conditions. The only identifiable compound was hydrolysis side-product **15**.

S.5.4 – Radical Clock Experiments (*Scheme 4A-B*)

S.5.4.1 – *Electrolysis of Isoxazoline **18** (Scheme 4A):*

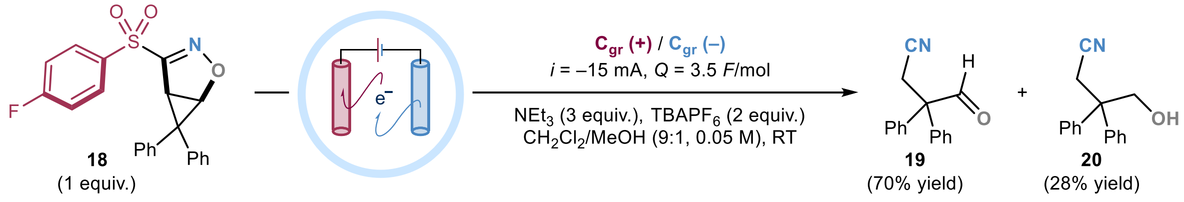

This experiment was conducted following ***General Procedure C*** – using isoxazoline **18** (79 mg, 0.2 mmol) as the substrate. The crude material was purified by flash column chromatography (*gradient from* hexane *to* 20% Et_2O in hexane) to provide β-cyano aldehyde **19** (33 mg, 70% yield) as a colorless oil. Continued elution delivered γ-hydroxy nitrile **20** (13 mg, 28% yield) as a colorless oil. Both compounds were previously unknown.

19 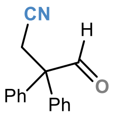

*Characterization Data for **19*** – **^{1}H NMR** (500 MHz, $CDCl_3$) δ 9.79 (s, 1H), 7.46 – 7.37 (m, 6H), 7.24 – 7.20 (m, 4H), 3.24 (s, 2H). **^{13}C NMR** (126 MHz, $CDCl_3$) δ 195.1, 137.0, 129.4, 128.7, 128.4, 117.3, 61.8, 24.6. **HRMS** (ESI$^+$) Exact mass calculated for $C_{16}H_{13}NO$ $[M+Na]^+$: 235.0997, found: 235.1007.

20 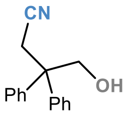

*Characterization Data for **20*** – **^{1}H NMR** (500 MHz, $CDCl_3$) δ 7.39 – 7.33 (m, 4H), 7.33 – 7.27 (m, 2H), 7.22 – 7.17 (m, 4H), 4.32 (d, *J* = 6.1 Hz, 2H), 3.25 (s, 2H), 1.41 (t, *J* = 6.4 Hz, 1H). **^{13}C NMR** (126 MHz, $CDCl_3$) δ 142.6, 128.8, 127.5 (2 signals), 118.0, 68.2, 50.8, 26.7. **HRMS** (ESI$^+$) Exact mass calculated for $C_{16}H_{15}ONNa$ $[M+Na]^+$: 260.1046, found: 260.1040.

S.5.4.2 – *Electrolysis of Isoxazoline **23** (Scheme 4B):*

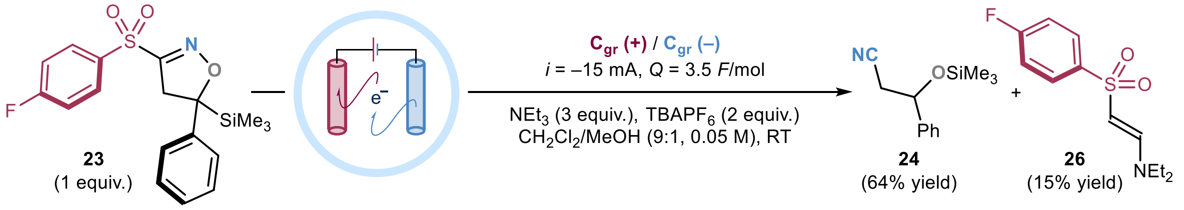

This experiment was conducted following ***General Procedure C*** – using isoxazoline **23** (145 mg, 0.4 mmol) as the substrate. The crude material was purified by flash column chromatography on activated Al_2O_3 support (*isocratic in* 10% Et_2O in hexane. *N.B. the silyl ether product is unstable on silica*) to provide β-cyano-silyl enol ether **24** (56 mg, 64% yield) as a colorless oil. Continued elution delivered sulfonyl enamine byproduct **26** (15 mg, 15% yield) as an amber oil. The spectroscopic properties of compounds **24**[17] and **26**[18] were consistent with the data available in the literature.

24 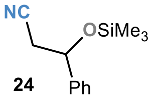

*Characterization Data for **24*** – **^{1}H NMR** (400 MHz, $CDCl_3$) δ 7.41 – 7.36 (m, 4H), 7.36 – 7.30 (m, 1H), 4.99 (dd, *J* = 7.2, 5.2 Hz, 1H), 2.76 – 2.63 (m, 2H), 0.12 (s, 9H). **^{13}C NMR** (101 MHz, $CDCl_3$) δ 142.0, 128.7, 128.4, 125.6, 117.7, 70.9, 29.7, 0.0.

26 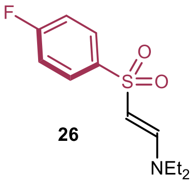

*Characterization Data for **26*** – **^{1}H NMR** (400 MHz, $CDCl_3$) δ 7.85 (dd, *J* = 8.9, $J_{H\text{–}F}$ = 5.1 Hz, 2H), 7.30 (d, *J* = 12.7 Hz, 1H), 7.12 (app t, *J* = 8.6 Hz, 2H), 4.87 (d, *J* = 12.6 Hz, 1H), 3.37 – 2.97 (m, 4H), 1.36 – 0.98 (m, 6H). **^{13}C NMR** (101 MHz, $CDCl_3$) δ 164.4 (d, $^1J_{C\text{–}F}$ = 252.4 Hz), 149.1, 141.5 (d, $^4J_{C\text{–}F}$ = 3.1 Hz), 128.7 (d, $^3J_{C\text{–}F}$ = 9.2 Hz), 115.9 (d, $^2J_{C\text{–}F}$ = 22.4 Hz), 91.2, 50.1, 42.7, 14.4, 11.1. **^{19}F NMR** (376 MHz, $CDCl_3$) δ –107.8.

S.6 – Scope of the Methodology

S.6.1 – Scope Limitations: The following substrates and functionalities were either not compatible, or did not perform well in the "*sew & cut*" protocol. In most cases, the reduced/lack of reactivity is ascribable to an inefficient 1,3-DC step – often due to the presence of functional groups that thwart the formation of nitrile oxide **I** (*c.f.* Scheme 2B). In general, when isoxazoline cycloadducts are successfully made, their electrochemical fragmentation occurs in high yields. Some classes of olefins (*e.g.* vinyl/allyl boronates, enamines) successfully underwent the *syn*-cyano-hydroxylation process, although the instability of the corresponding products hampered their isolation by chromatography.

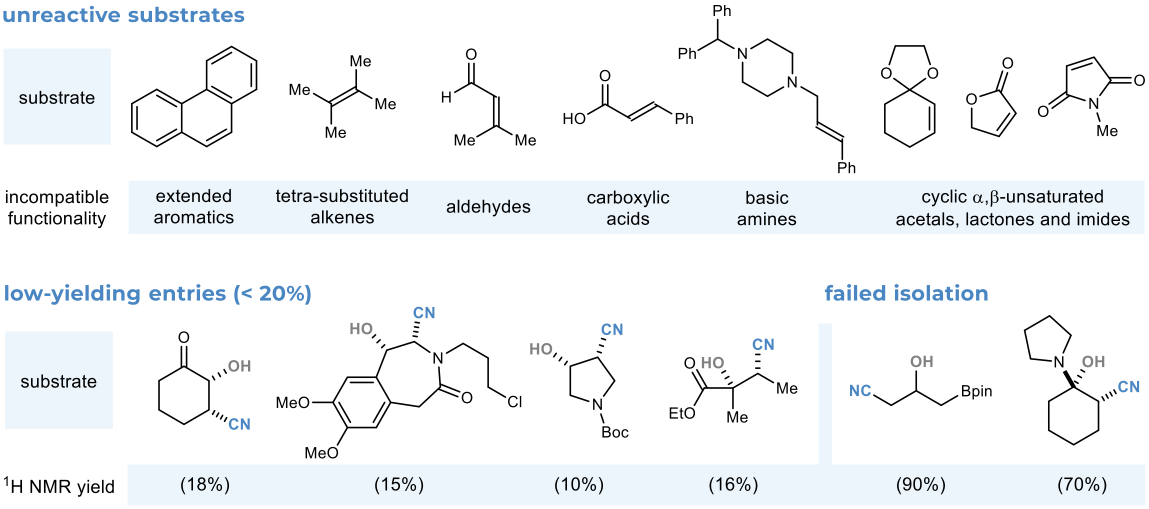

S.6.2 – Characterization Data of Compounds 9 and 27-71 (*Table 1*)

(1*S**,2*S**)-2-hydroxycyclooctane-1-carbonitrile (9)

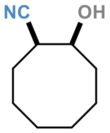

9

Prepared according to ***General Procedure D*** – using α-diazo-sulfone **1a** (48.05 mg, 0.24 mmol, 1.2 equiv.) as the nitrile oxide precursor, *cis*-cyclooctene (26.1 µL, 0.20 mmol, 1 equiv.) as the olefin substrate, and NEt_3 as the sacrificial reductant in the electrochemical step. The crude mixture was purified by flash column chromatography (*isocratic in* 60% Et_2O in hexane) to provide product **9** (25.5 mg, 83% yield, *single diastereoisomer*) as a pale yellow oil. The title compound was previously unknown. The spectroscopic properties of **9** differ from those reported for its *anti*-diastereoisomer.[19]

¹H NMR (500 MHz, $CDCl_3$) δ 4.03 (td, *J* = 6.9, 3.4 Hz, 1H), 3.08 (dt, *J* = 9.2, 3.5 Hz, 1H), 2.58 (s, 1H), 2.06 (dtd, *J* = 15.0, 8.8, 3.3 Hz, 1H), 1.93 – 1.82 (m, 3H), 1.80 – 1.70 (m, 2H), 1.69 – 1.44 (m, 6H). **¹³C NMR** (126 MHz, $CDCl_3$) δ 121.5, 70.4, 53.5, 37.4, 33.0, 26.2, 26.0, 24.9, 23.2. **HRMS** (ESI^+) Exact mass calculated for $C_9H_{16}ON$ $[M+H]^+$: 154.1226, found: 154.1220.

3-hydroxynonanenitrile (27)

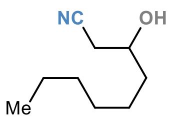

27

Prepared according to ***General Procedure D*** – using α-diazo-sulfone **1a** (40.0 mg, 0.20 mmol, 1 equiv.) as the nitrile oxide precursor, 1-octene (93.5 µL, 0.60 mmol, 3 equiv.) as the olefin substrate, and NEt_3 as the sacrificial reductant in the electrochemical step. The crude mixture was purified by flash column chromatography (*isocratic in* 80% Et_2O in hexane) to provide product **27** (31 mg, 99% yield) as a pale yellow oil. The spectroscopic properties of this compound were consistent with the data available in the literature.[20]

^{1}H NMR (500 MHz, $CDCl_3$) δ 3.98 – 3.91 (m, 1H), 2.57 (dd, *J* = 16.7, 4.8 Hz, 1H), 2.49 (dd, *J* = 16.7, 6.4 Hz, 1H), 2.17 (br s, 1H), 1.66 – 1.53 (m, 2H), 1.48 – 1.40 (m, 1H), 1.39 – 1.30 (m, 7H), 0.89 (t, *J* = 6.7 Hz, 3H). **^{13}C NMR** (126 MHz, $CDCl_3$) δ 117.7, 67.8, 36.6, 31.7, 29.0, 26.1, 25.3, 22.5, 14.0.

3-hydroxy-4,4-dimethylpentanenitrile (28)

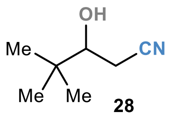

28

Prepared according to ***General Procedure D*** – using α-diazo-sulfone **1a** (40.0 mg, 0.20 mmol, 1 equiv.) as the nitrile oxide precursor, 3,3-dimethyl-1-butene (77.3 µL, 0.60 mmol, 3 equiv.) as the olefin substrate, and NEt_3 as the sacrificial reductant in the electrochemical step. The crude mixture was purified by flash column chromatography (*isocratic in* 50% Et_2O in hexane) to provide product **28** (21.0 mg, 85% yield) as a colorless oil. The spectroscopic properties of this compound were consistent with the data available in the literature.[21]

^{1}H NMR (400 MHz, $CDCl_3$) δ 3.71 – 3.65 (m, 1H), 2.57 (dd, *J* = 16.6, 3.0 Hz, 1H), 2.44 (dd, *J* = 16.6, 9.7 Hz, 1H), 2.27 (d, *J* = 5.1 Hz, 1H), 0.96 (s, 9H). **^{13}C NMR** (101 MHz, $CDCl_3$) δ 119.1, 75.6, 35.0, 25.3, 21.6.

3-hydroxyhept-6-enenitrile (29)

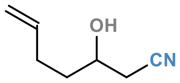

29

Prepared according to ***General Procedure E*** – using chloroxime **1b** (47.5 mg, 0.20 mmol, 1 equiv.) as the nitrile oxide precursor, 1,5-hexadiene (28.5 µL, 0.24 mmol, 1.2 equiv.) as the olefin substrate, and 1,4-cyclohexadiene as the sacrificial reductant in the electrochemical step. The crude mixture was purified by flash column chromatography (*isocratic in* 70% Et_2O in hexane) to provide product **29** (14.0 mg, 55% yield) as a colorless oil. The spectroscopic properties of this compound were consistent with the data available in the literature.[22]

^{1}H NMR (500 MHz, $CDCl_3$) δ 5.87 – 5.76 (m, 1H), 5.09 (app dq, *J* = 17.1, 1.7 Hz, 1H), 5.03 (d, *J* = 10.2 Hz, 1H), 4.03 – 3.94 (m, 1H), 2.57 (dd, *J* = 16.7, 4.9 Hz, 1H), 2.50 (dd, *J* = 16.7, 6.4 Hz, 1H), 2.29 – 2.14 (m, 2H), 2.14 – 2.10 (m, 1H), 1.74 – 1.67 (m, 2H). **^{13}C NMR** (126 MHz, $CDCl_3$) δ 137.3, 117.6, 116.0, 67.4, 35.5, 29.8, 26.3.

6-bromo-3-hydroxyhexanenitrile (30)

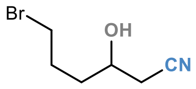

30

Prepared according to ***General Procedure E*** – using chloroxime **1b** (47.5 mg, 0.20 mmol, 1 equiv.) as the nitrile oxide precursor, 5-bromo-1-pentene (28.4 μL, 0.24 mmol, 1.2 equiv.) as the olefin substrate, and 1,4-cyclohexadiene as the sacrificial reductant in the electrochemical step. The crude mixture was purified by flash column chromatography (*isocratic in* 70% Et_2O in hexane) to provide product **30** (27.0 mg, 70% yield) as a pale yellow oil. The title compound was previously unknown.

^{1}H NMR (500 MHz, $CDCl_3$) δ 4.09 – 3.97 (m, 1H), 3.51 – 3.45 (m, 2H), 2.62 (dd, J = 16.7, 4.8 Hz, 1H), 2.55 (dd, J = 16.7, 6.6 Hz, 1H), 2.29 – 2.24 (m, 1H), 2.17 – 2.05 (m, 1H), 2.04 – 1.91 (m, 1H), 1.87 – 1.70 (m, 2H). ^{13}C NMR (126 MHz, CDCl3) δ 117.5, 67.2, 35.0, 33.1, 28.6, 26.6. **HRMS** (ESI$^-$) Exact mass calculated for $C_6H_{10}ONBrCl$ $[M+Cl]^-$: 225.9629, found: 225.9631.

3-(2,2-dichlorocyclopropyl)-3-hydroxypropanenitrile (31)

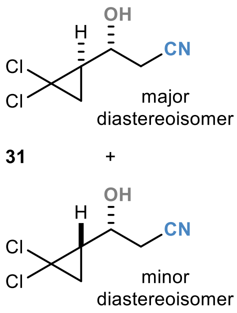

31 +

Prepared according to ***General Procedure E*** – using chloroxime **1b** (143 mg, 0.60 mmol, 1 equiv.) as the nitrile oxide precursor, 1,1-dichloro-2-vinylcyclopropane (85.7 μL, 0.72 mmol, 1.2 equiv.) as the olefin substrate, and NEt_3 (25.1 μL, 1.80 mmol, 3 equiv.) as the sacrificial reductant in the electrochemical step. In this case, the crude 1,3-DC mixture was filtered through a short pad of silica gel (*flushing first with 15% Et_2O in hexane to remove the remainder alkene and, then, with EtOAc to elute the cycloadduct intermediate*) prior submission to the electrolysis. Purification of the crude material by flash column chromatography (*isocratic in* 70% Et_2O in hexane) provided product ***31-major*** *diastereoisomer* (32.0 mg, 35% yield) as a colorless oil. Continued elution delivered product ***31-minor*** *diastereoisomer* (27.0 mg, 29% yield) as a colorless oil. Both diastereoisomers of the title compound were previously unknown. The diastereoisomeric ratio was determined by ^{1}H NMR analysis of the crude reaction mixture. The relative stereochemistry of both diastereoisomers has been inferred by ^{1}H NOESY NMR analysis.

Characterization Data for ***31-major*** – **^{1}H NMR** (500 MHz, $CDCl_3$) δ 3.83 – 3.77 (m, 1H), 2.86 (dd, *J* = 16.8, 4.1 Hz, 1H), 2.78 (dd, *J* = 16.7, 6.6 Hz, 1H), 2.41 (d, *J* = 5.0 Hz, 1H), 1.86 (app dt, *J* = 10.4, 7.6 Hz, 1H), 1.79 (dd, *J* = 10.3, 7.1 Hz, 1H), 1.56 (app t, *J* = 7.3 Hz, 1H). **^{13}C NMR** (126 MHz, $CDCl_3$) δ 116.8, 68.2, 57.9, 34.1, 25.9, 25.4. **HRMS** (ESI$^-$) Exact mass calculated for $C_7H_8O_3NCl_2$ $[M+CO_2H]^-$: 223.9887, found: 223.9878.

Characterization Data for ***31-minor*** – **^{1}H NMR** (400 MHz, $CDCl_3$) δ 3.84 – 3.73 (m, 1H), 2.74 (d, *J* = 5.8 Hz, 2H), 2.68 – 2.65 (m, 1H), 1.97 (app td, *J* = 10.1, 7.8 Hz, 1H), 1.82 (dd, *J* = 10.4, 7.4 Hz, 1H), 1.44 (app t, *J* = 7.5 Hz, 1H). **^{13}C NMR** (101 MHz, $CDCl_3$) δ 116.6, 69.5, 58.6, 35.0, 25.6, 24.4. **HRMS** (ESI$^-$) Exact mass calculated for $C_6H_7ONCl_3$ $[M+Cl]^-$: 213.9588, found: 213.9588.

3-hydroxy-4-(methylthio)butanenitrile (32)

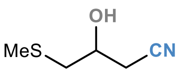

32

Prepared according to ***General Procedure E*** – using chloroxime **1b** (47.5 mg, 0.20 mmol, 1 equiv.) as the nitrile oxide precursor, allyl methyl sulfide (26.4 μL, 0.24 mmol, 1.2 equiv.) as the olefin substrate, and Et_3N as the sacrificial reductant in the electrochemical step. The crude mixture was purified by flash column chromatography (*isocratic in* 50% Et_2O in hexane) to provide product **32** (24 mg, 92% yield) as an amber oil. The title compound was previously unknown.

^{1}**H NMR** (400 MHz, $CDCl_3$) δ 4.04 (ddt, *J* = 10.1, 8.3, 5.1 Hz, 1H), 2.92 – 2.87 (m, 1H), 2.79 (dd, *J* = 13.9, 4.4 Hz, 1H), 2.67 (dd, *J* = 5.7, 4.7 Hz, 2H), 2.65 – 2.61 (m, 1H), 2.16 (s, 3H). ^{13}**C NMR** (101 MHz, $CDCl_3$) δ 117.0, 65.3, 40.6, 24.6, 15.7.

3-hydroxy-4-(phenylsulfonyl)butanenitrile (33)

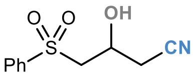

33

Prepared according to ***General Procedure E*** – using chloroxime **1b** (47.5 mg, 0.20 mmol, 1 equiv.) as the nitrile oxide precursor, allyl phenyl sulfone (36.8 μL, 0.24 mmol, 1.2 equiv.) as the olefin substrate, and Et_3N as the sacrificial reductant in the electrochemical step. In this case, the crude 1,3-DC mixture was filtered through a short pad of silica gel (*flushing first with 15% Et_2O in hexane to remove the remainder alkene and, then, with EtOAc to elute the cycloadduct intermediate*) prior submission to the electrolysis. Purification of the crude material by flash column chromatography (*isocratic in* Et_2O) provided product **33** (23.6 mg, 52% yield) as a pale yellow solid. The title compound was previously unknown.

^{1}**H NMR** (400 MHz, $CDCl_3$) δ 7.99 – 7.91 (m, 2H), 7.78 – 7.69 (m, 1H), 7.69 – 7.59 (m, 2H), 4.52 (dtt, *J* = 8.3, 5.4, 2.5 Hz, 1H), 3.77 (d, *J* = 3.2 Hz, 1H), 3.48 – 3.29 (m, 2H), 2.77 – 2.60 (m, 2H). ^{13}**C NMR** (101 MHz, $CDCl_3$) δ 138.6, 134.6, 129.8, 128.0, 116.0, 62.5, 60.6, 25.4. **HRMS (ESI$^+$)** Mass calculated for $C_{10}H_{11}O_3NNaS$ $[M+Na]^+$: 248.0352, found: 248.0346.

3-hydroxypentanedinitrile (34)

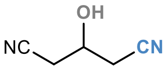

34

Prepared according to ***General Procedure D*** – using α-diazo-sulfone **1a** (40.0 mg, 0.20 mmol, 1 equiv.) as the nitrile oxide precursor, allyl cyanide (48.3 μL, 0.60 mmol, 3 equiv.) as the olefin substrate, and NEt_3 as the sacrificial reductant in the electrochemical step. The crude mixture was purified by flash column chromatography (*isocratic in* 80% Et_2O in hexane) to provide product **34** (23 mg, 99% yield) as an amber oil. The spectroscopic properties of this compound were consistent with the data available in the literature.[23]

^{1}**H NMR** (400 MHz, $CDCl_3$) δ 4.35 (q, *J* = 6.0 Hz, 1H), 3.37 (s, 1H), 2.72 (d, *J* = 5.9 Hz, 4H). ^{13}**C NMR** (101 MHz, $CDCl_3$) δ 116.0, 64.0, 25.4.

tert-butyl (3-cyano-2-hydroxypropyl)carbamate (35)

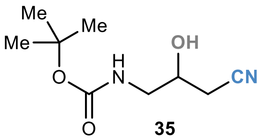

35

Prepared according to ***General Procedure E*** – using chloroxime **1b** (47.5 mg, 0.20 mmol, 1 equiv.) as the nitrile oxide precursor, *tert*-butyl allylcarbamate (105 mg, 0.60 mmol, 3 equiv.) as the olefin substrate, and Et_3N as the sacrificial reductant in the electrochemical step. In this case, the crude 1,3-DC mixture was filtered through a short pad of silica gel (*flushing first with 50% Et_2O in hexane to remove the remainder alkene and, then, with EtOAc to elute the cycloadduct intermediate*) prior submission to the electrolysis. Purification of the crude material by flash column chromatography (*isocratic in* Et_2O, *SiO_2 neutralized with 2% Et_3N prior loading*) provided product **35** (26.9 mg, 67% yield) as a yellow oil. The title compound was previously unknown.

^{1}H NMR (400 MHz, $CDCl_3$) δ 5.15 – 5.00 (m, 1H), 4.15 – 3.97 (m, 2H), 3.47 – 3.37 (m, 1H), 3.33 – 3.22 (m, 1H), 2.58 (d, *J* = 6.2 Hz, 2H), 1.47 (s, 9H). **^{13}C NMR** (101 MHz, $CDCl_3$) δ 157.7, 117.3, 80.8, 67.9, 45.8, 28.3, 23.3. **HRMS (ESI$^+$)** Mass calculated for $C_9H_{16}O_3N_2Na$ $[M+Na]^+$: 223.1053, found: 223.1046.

methyl 3-cyano-2-hydroxypropanoate (36)

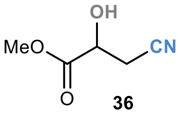

36

Prepared according to ***General Procedure D*** – using α-diazo-sulfone **1a** (40.0 mg, 0.20 mmol, 1 equiv.) as the nitrile oxide precursor, methyl acrylate (54.0 μL, 0.60 mmol, 3 equiv.) as the olefin substrate, and NEt_3 as the sacrificial reductant in the electrochemical step. The crude mixture was purified by flash column chromatography (*isocratic in* 80% Et_2O in hexane) to provide product **36** (26 mg, 99% yield) as a yellow oil. The spectroscopic properties of this compound were consistent with the data available in the literature.[24]

^{1}H NMR (400 MHz, $CDCl_3$) 4.46 (app t, *J* = 5.4 Hz, 1H), 3.89 (s, 3H), 3.30 (s, 1H), 2.89 (dd, *J* = 16.8, 5.0 Hz, 1H), 2.80 (dd, *J* = 16.8, 5.7 Hz, 1H). **^{13}C NMR** (101 MHz, $CDCl_3$) δ 172.0, 115.9, 66.5, 53.6, 23.5.

2-((*tert*-butyldimethylsilyl)oxy)-3-cyanopropanamide (37)

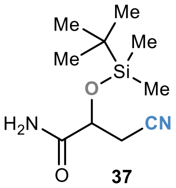

37

Prepared according to ***General Procedure E*** – using chloroxime **1b** (47.5 mg, 0.20 mmol, 1 equiv.) as the nitrile oxide precursor, acrylamide (17.1 mg, 0.24 mmol, 1.2 equiv.) as the olefin substrate, and Et_3N as the sacrificial reductant in the electrochemical step. Due to both its instability on silica gel and its high polarity, the hydroxy nitrile product was converted into the corresponding silyl ether, prior purification. This was achieved by concentrating the crude electrolysis mixture, adding a solution of *tert*-butyldimethylsilyl chloride (90.4 mg, 0.60 mmol, 3 equiv.) and imidazole (54.5 mg, 0.80 mmol, 4 equiv.) in DMF (0.6 mL), stirring the resulting mixture for 2 hours and concentrating again in vacuo. Purification of the crude

material by flash column chromatography (*isocratic in* 80% Et_2O in hexane) provided product **37** (17 mg, 37% yield) as a colorless solid. The title compound was previously unknown.

^{1}H NMR (400 MHz, $CDCl_3$) δ 6.62 (s, 1H), 5.66 (s, 1H), 4.34 (dd, J = 5.5, 4.6 Hz, 1H), 2.82 (app t, J = 5.0 Hz, 2H), 0.97 (s, 9H), 0.22 (s, 3H), 0.17 (s, 3H). **^{13}C NMR** (101 MHz, $CDCl_3$) δ 173.1, 116.4, 69.3, 25.6, 24.8, 18.0, –4.9, –5.2. **HRMS (ESI$^+$)** Mass calculated for $C_{10}H_{20}O_2N_2NaSi$ $[M+Na]^+$: 251.1186, found: 251.1177.

3-((*tert*-butyldimethylsilyl)oxy)-4-morpholino-4-oxobutanenitrile (38)

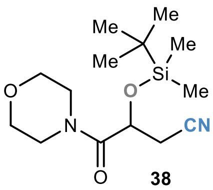

38

Prepared according to ***General Procedure E*** – using chloroxime **1b** (47.5 mg, 0.20 mmol, 1 equiv.) as the nitrile oxide precursor, 4-acryloylmorpholine (30.2 µL, 0.24 mmol, 1.2 equiv.) as the olefin substrate, and Et_3N as the sacrificial reductant in the electrochemical step. Due to both its instability on silica gel and its high polarity, the hydroxy nitrile product was converted into the corresponding silyl ether, prior purification. This was achieved by concentrating the crude electrolysis mixture, adding a solution of *tert*-butyldimethylsilyl chloride (90.4 mg, 0.60 mmol, 3 equiv.) and imidazole (54.5 mg, 0.80 mmol, 4 equiv.) in DMF (0.6 mL), stirring the resulting mixture for 2 hours and concentrating again in vacuo. Purification of the crude material by flash column chromatography (*isocratic in* 80% Et_2O in hexane) provided product **38** (51 mg, 85% yield) as a colorless oil. The title compound was previously unknown.

^{1}H NMR (400 MHz, $CDCl_3$) δ 4.73 (t, J = 6.3 Hz, 1H), 3.86 – 3.73 (m, 2H), 3.71 (t, J = 5.0 Hz, 4H), 3.66 – 3.59 (m, 2H), 2.82 – 2.74 (m, 2H), 0.92 (s, 9H), 0.16 (s, 3H), 0.15 (s, 3H). **^{13}C NMR** (101 MHz, $CDCl_3$) δ 168.1, 116.8, 70.0, 66.8 (2 signals), 46.0, 42.9, 25.6, 24.2, 18.1, –4.6, –4.9. **HRMS (ESI$^+$)** Mass calculated for $C_{14}H_{26}N_2O_3Si$ $[M+Na]^+$: 298.1713, found: 298.1719.

diethyl (2-cyano-1-hydroxyethyl)phosphonate (39)

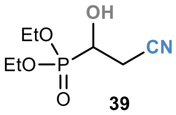

39

Prepared according to ***General Procedure D*** – using α-diazo-sulfone **1a** (80.0 mg, 0.40 mmol, 2 equiv.) as the nitrile oxide precursor, diethyl vinylphosphonate (31 µL, 0.20 mmol, 1 equiv.) as the olefin substrate, and NEt_3 as the sacrificial reductant in the electrochemical step. The crude mixture was purified by flash column chromatography (*isocratic in* 30% acetone in Et_2O) to provide product **39** (51 mg, 50% yield) as an amber oil. The title compound was previously unknown.

^{1}H NMR (400 MHz, $CDCl_3$) δ 5.18 (s, 1H), 4.33 – 4.11 (m, 5H), 2.88 – 2.69 (m, 2H), 1.37 (t, J = 7.1 Hz, 3H), 1.36 (t, J = 7.1 Hz, 3H). **^{13}C NMR** (101 MHz, $CDCl_3$) δ 116.8 (d, $^3J_{C\text{-}P}$ = 16.6 Hz), 64.3 (d, $^1J_{C\text{-}P}$ = 50.9 Hz), 63.7 (d, $^2J_{C\text{-}P}$ = 53.5 Hz), 63.1 (d, $^2J_{C\text{-}P}$ = 49.4 Hz), 21.4 (d, $^2J_{C\text{-}P}$ = 4.7 Hz), 16.5 (d, $^3J_{C\text{-}P}$ = 5.2 Hz),

16.4 (d, $^3J_{C\text{-}P}$ = 5.2 Hz). **^{31}P NMR** (162 MHz, $CDCl_3$) δ 20.4. **HRMS (ESI^+):** Mass calculated for $C_7H_{14}O_4NNaP$ $[M+Na]^+$: 230.0553, found: 230.0548.

3-(1,3-dioxolan-2-yl)-3-hydroxypropanenitrile (40)

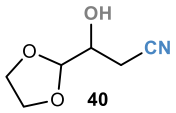

40

Prepared according to ***General Procedure E*** – using chloroxime **1b** (47.5 mg, 0.20 mmol, 1 equiv.) as the nitrile oxide precursor, 2-vinyl-1,3-dioxolane (24.0 µL, 0.24 mmol, 1.2 equiv.) as the olefin substrate, and Et_3N as the sacrificial reductant in the electrochemical step. The crude mixture was purified by flash column chromatography (*isocratic in* Et_2O, *SiO_2 neutralized with 2% Et_3N prior loading*) to provide product **40** (11 mg, 39% yield) as a yellow oil. The title compound was previously unknown.

^{1}H NMR (400 MHz, $CDCl_3$) δ 4.92 (d, *J* = 4.0 Hz, 1H), 4.07 – 3.90 (m, 5H), 2.70 – 2.57 (m, 2H), 2.51 (s, 1H). **^{13}C NMR** (101 MHz, $CDCl_3$) δ 117.1, 103.3, 68.2, 65.9, 65.6, 20.8. **HRMS ($APCI^+$)** Mass calculated for $C_6H_{10}O_3N$ $[M+H]^+$: 144.0655, found: 144.0654.

3-hydroxy-3-(trimethylsilyl)propanenitrile (41)

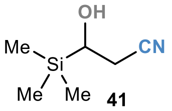

41

\+

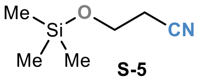

S-5

Prepared according to ***General Procedure E*** – using chloroxime **1b** (47.5 mg, 0.20 mmol, 1 equiv.) as the nitrile oxide precursor, vinyltrimethylsilane (35.2 µL, 0.24 mmol, 1.2 equiv.) as the olefin substrate, and Et_3N as the sacrificial reductant in the electrochemical step. In this case, the crude 1,3-DC mixture was filtered through a short pad of silica gel (*flushing first with 15% Et_2O in hexane to remove the remainder alkene and, then, with EtOAc to elute the cycloadduct intermediate*) prior submission to the electrolysis. Purification of the crude material by flash column chromatography (*isocratic in* 50% Et_2O in hexane) provided product **41** (25 mg, 44% yield) as a yellow solid. The spectroscopic properties of this compound were consistent with the data available in the literature.[25]

^{1}H NMR (400 MHz, $CDCl_3$) δ 3.50 (dt, *J* = 8.7, 3.9 Hz, 1H), 2.49 – 2.36 (m, 2H), 1.91 (s, 1H), 0.00 (s, 9H). **^{13}C NMR** (101 MHz, $CDCl_3$) δ 119.0, 61.7, 23.1, –4.0.

^{1}H NMR analysis of the crude reaction mixture (after electrolysis) revealed the presence of 3-((trimethylsilyl)oxy)propanenitrile **S-5** – formed via Brook rearrangement reactivity (*c.f.* Scheme 4B). However, isolation of **S-5** was thwarted by its instability on silica gel. *Observed characteristic signal:* **^{1}H NMR** (400 MHz, $CDCl_3$) δ 3.81 (t, *J* = 6.4 Hz).

3-((*tert*-butyldimethylsilyl)oxy)-3-(2-oxopyrrolidin-1-yl)propanenitrile (42)

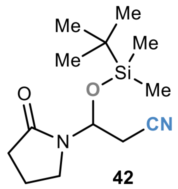

42

Prepared according to ***General Procedure E*** – using chloroxime **1b** (47.5 mg, 0.20 mmol, 1 equiv.) as the nitrile oxide precursor, 1-vinyl-2-pyrrolidinone (25.6 µL, 0.24 mmol, 1.2 equiv.) as the olefin substrate, and Et_3N as the sacrificial reductant in the electrochemical step. Due to both its instability on silica gel and its high polarity, the hydroxy nitrile product was converted into the corresponding silyl ether, prior purification. This was achieved by concentrating the crude electrolysis mixture, adding a solution of *tert*-butyldimethylsilyl chloride (90.4 mg, 0.60 mmol, 3 equiv.) and imidazole (54.5 mg, 0.80 mmol, 4 equiv.) in DMF (0.6 mL), stirring the resulting mixture for 2 hours and concentrating again in vacuo. Purification of the crude material by flash column chromatography on Al_2O_3 support (*isocratic in* 20% Et_2O in pentane) provided product **42** (34 mg, 73% yield) as a colorless oil. The title compound was previously unknown.

^{1}H NMR (400 MHz, $CDCl_3$) δ 5.87 (dd, *J* = 6.4, 5.3 Hz, 1H), 3.56 – 3.36 (m, 2H), 2.76 – 2.57 (m, 2H), 2.50 – 2.29 (m, 2H), 2.15 – 1.96 (m, 2H), 0.90 (s, 9H), 0.17 (s, 3H), 0.06 (s, 3H). **^{13}C NMR** (101 MHz, $CDCl_3$) δ 174.7, 116.1, 71.7, 41.1, 31.4, 25.5, 25.1, 18.3, 17.9, –5.0, –5.5. **HRMS (ESI$^+$)** Mass calculated for $C_{13}H_{24}O_2N_2NaSi$ [M+Na]$^+$: 291.1499, found: 291.1488.

3-hydroxy-3-(thiophen-2-yl)propanenitrile (43)

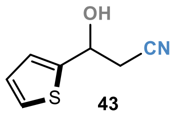

43

Prepared according to ***General Procedure E*** – using chloroxime **1b** (47.5 mg, 0.20 mmol, 1 equiv.) as the nitrile oxide precursor, 2-vinylthiophene (26.4 mg, 0.24 mmol, 1.2 equiv.) as the olefin substrate, and Et_3N as the sacrificial reductant in the electrochemical step. In this case, the crude 1,3-DC mixture was filtered through a short pad of silica gel (*flushing first with 15% Et_2O in hexane to remove the remainder alkene and, then, with EtOAc to elute the cycloadduct intermediate*) prior submission to the electrolysis. Purification of the crude material by flash column chromatography (*isocratic in* 80% Et_2O in hexane) provided product **43** (17 mg, 55% yield) as a yellow solid. The spectroscopic properties of this compound were consistent with the data available in the literature.[26]

^{1}H NMR (400 MHz, $CDCl_3$) δ 7.35 (dd, *J* = 5.0, 1.2 Hz, 1H), 7.12 (dt, *J* = 3.5, 1.0 Hz, 1H), 7.04 (dd, *J* = 5.0, 3.6 Hz, 1H), 5.34 (td, *J* = 6.0, 4.0 Hz, 1H), 2.98 – 2.84 (m, 2H), 2.64 (d, *J* = 4.2 Hz, 1H). **^{13}C NMR** (101 MHz, $CDCl_3$) δ 144.4, 127.2, 125.9, 124.8, 116.8, 66.4, 28.2.

3-hydroxy-3-(pyridin-2-yl)propanenitrile (44)

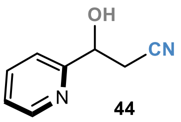

44

Prepared according to ***General Procedure E*** – using chloroxime **1b** (47.5 mg, 0.20 mmol, 1 equiv.) as the nitrile oxide precursor, 2-vinylpyridine (25.9 µL, 0.24 mmol, 1.2 equiv.) as the olefin substrate, and Et_3N as the sacrificial

reductant in the electrochemical step. The crude mixture was purified by flash column chromatography (*isocratic in* 80% Et_2O in hexane) to provide product **44** (25 mg, 85% yield) as a yellow oil. The spectroscopic properties of this compound were consistent with the data available in the literature.[27]

^{1}H NMR (400 MHz, $CDCl_3$) δ 8.60 (app dt, *J* = 4.9, 1.4 Hz, 1H), 7.78 (app td, *J* = 7.7, 1.7 Hz, 1H), 7.41 (d, *J* = 7.8 Hz, 1H), 7.32 (ddd, *J* = 7.6, 4.9, 1.1 Hz, 1H), 5.05 (t, *J* = 5.8 Hz, 1H), 4.59 (s, 1H), 2.86 (dd, *J* = 5.8, 0.6 Hz, 2H). **^{13}C NMR** (101 MHz, $CDCl_3$) δ 157.9, 148.8, 137.4, 123.7, 120.6, 117.1, 68.7, 27.2.

3-(4-chlorophenyl)-3-hydroxypropanenitrile (45)

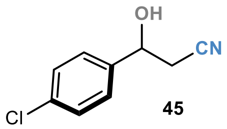

45

Prepared according to ***General Procedure E*** – using chloroxime **1b** (47.5 mg, 0.20 mmol, 1 equiv.) as the nitrile oxide precursor, 4-chlorostyrene (28.8 µL, 0.24 mmol, 1.2 equiv.) as the olefin substrate, and Et_3N as the sacrificial reductant in the electrochemical step. The crude mixture was purified by flash column chromatography (*isocratic in* 60% Et_2O in hexane) to provide product **45** (27 mg, 75% yield) as a pale yellow oil. The spectroscopic properties of this compound were consistent with the data available in the literature.[28]

^{1}H NMR (500 MHz, $CDCl_3$) δ 7.41 – 7.32 (m, 4H), 5.04 (td, *J* = 6.1, 1.8 Hz, 1H), 2.75 (dd, *J* = 6.1, 1.4 Hz, 2H), 2.55 (br, 1H). **^{13}C NMR** (126 MHz, $CDCl_3$) δ 139.4, 134.7, 129.1, 127.0, 116.9, 69.5, 28.0.

3-hydroxy-3-(4-(trifluoromethyl)phenyl)propanenitrile (46)

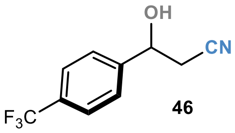

46

Prepared according to ***General Procedure E*** – using chloroxime **1b** (47.5 mg, 0.20 mmol, 1 equiv.) as the nitrile oxide precursor, 4-(trifluoromethyl)styrene (35.5 µL, 0.24 mmol, 1.2 equiv.) as the olefin substrate, and Et_3N as the sacrificial reductant in the electrochemical step. In this case, the crude 1,3-DC mixture was filtered through a short pad of silica gel (*flushing first with 15% Et_2O in hexane to remove the remainder alkene and, then, with EtOAc to elute the cycloadduct intermediate*) prior submission to the electrolysis. Purification of the crude material by flash column chromatography (*isocratic in* 50% Et_2O in hexane) provided product **46** (40 mg, 92% yield) as a pale yellow oil. The spectroscopic properties of this compound were consistent with the data available in the literature.[28]

^{1}H NMR (400 MHz, $CDCl_3$) δ 7.70 – 7.50 (m, 4H), 5.13 (t, *J* = 6.2 Hz, 1H), 2.86 (s, 1H), 2.79 (d, *J* = 6.2 Hz, 2H). **^{13}C NMR** (101 MHz, $CDCl_3$) δ 142.1, 131.4 (q, $^2J_{C-F}$ = 32.5 Hz), 125.7 (q, $^4J_{C-F}$ = 3.8 Hz), 123.9 (q, $^1J_{C-F}$ = 271.5 Hz), 122.6 (q, $^3J_{C-F}$ = 3.9 Hz), 116.9, 69.4, 28.1. **^{19}F NMR** (376 MHz, $CDCl_3$) δ –62.7.

3-hydroxy-3-(4-methoxyphenyl)propanenitrile (47)

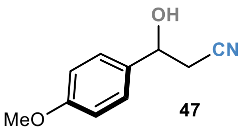

Prepared according to ***General Procedure E*** – using chloroxime **1b** (47.5 mg, 0.20 mmol, 1 equiv.) as the nitrile oxide precursor, 4-methoxystyrene (32.1 µL, 0.24 mmol, 1.2 equiv.) as the olefin substrate, and Et_3N as the sacrificial reductant in the electrochemical step. The crude mixture was purified by flash column chromatography (*isocratic in* 60% Et_2O in hexane) to provide product **47** (32 mg, 90% yield) as a yellow oil. The spectroscopic properties of this compound were consistent with the data available in the literature.[28]

^{1}H NMR (500 MHz, $CDCl_3$) δ 7.35 – 7.29 (m, 2H), 6.95 – 6.88 (m, 2H), 4.99 (td, *J* = 6.3, 3.0 Hz, 1H), 3.81 (s, 3H), 2.79 – 2.69 (m, 2H), 2.47 (br s, 1H). **^{13}C NMR** (126 MHz, $CDCl_3$) δ 158.0, 131.3, 125.0, 115.5, 112.4, 67.9, 53.4, 26.0.

3-hydroxy-3-phenylpropanenitrile (48)

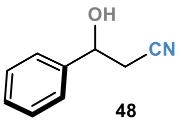

Prepared according to ***General Procedure E*** – using chloroxime **1b** (47.5 mg, 0.20 mmol, 1 equiv.) as the nitrile oxide precursor, styrene (27.6 µL, 0.24 mmol, 1.2 equiv.) as the olefin substrate, and Et_3N as the sacrificial reductant in the electrochemical step. The crude mixture was purified by flash column chromatography (*isocratic in* 60% Et_2O in hexane) to provide product **48** (26 mg, 88% yield) as a pale yellow oil. The spectroscopic properties of this compound were consistent with the data available in the literature.[28]

^{1}H NMR (400 MHz, $CDCl_3$) δ 7.43 – 7.32 (m, 5H), 5.03 (app t, *J* = 6.3 Hz, 1H), 2.79 – 2.70 (m, 2H), 2.63 (s, 1H). **^{13}C NMR** (101 MHz, $CDCl_3$) δ 141.0, 129.0, 128.9, 125.6, 117.3, 70.1, 28.0.

3-(2,6-dichlorophenyl)-3-hydroxypropanenitrile (49)

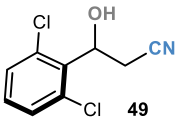

Prepared according to ***General Procedure E*** – using chloroxime **1b** (47.5 mg, 0.20 mmol, 1 equiv.) as the nitrile oxide precursor, 2,6-dichlorostyrene (41.5 mg, 0.24 mmol, 1.2 equiv.) as the olefin substrate, and Et_3N as the sacrificial reductant in the electrochemical step. The crude mixture was purified by flash column chromatography (*isocratic in* 50% Et_2O in hexane) to provide product **49** (41 mg, 94% yield) as a colorless oil. The spectroscopic properties of this compound were consistent with the data available in the literature.[29]

^{1}H NMR (500 MHz, $CDCl_3$) δ 7.43 – 7.35 (m, 2H), 7.28 – 7.20 (m, 1H), 5.83 (dd, *J* = 8.6, 6.3 Hz, 1H), 3.32 (br s, 1H), 3.21 (dd, *J* = 16.8, 8.6 Hz, 1H), 2.98 (dd, *J* = 16.8, 6.3 Hz, 1H). **^{13}C NMR** (126 MHz, $CDCl_3$) δ 134.4, 134.1, 130.4, 129.8, 116.6, 67.7, 24.0.

3-hydroxy-3,3-diphenylpropanenitrile (50)

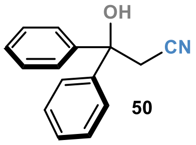
50

Prepared according to ***General Procedure E*** – using chloroxime **1b** (47.5 mg, 0.20 mmol, 1 equiv.) as the nitrile oxide precursor, 1,1-diphenylethylene (42.4 µL, 0.24 mmol, 1.2 equiv.) as the olefin substrate, and Et_3N as the sacrificial reductant in the electrochemical step. In this case, the crude 1,3-DC mixture was filtered through a short pad of silica gel (*flushing first with 15% Et_2O in hexane to remove the remainder alkene and, then, with EtOAc to elute the cycloadduct intermediate*) prior submission to the electrolysis. Purification of the crude material by flash column chromatography (*isocratic in* 50% Et_2O in hexane) provided product **50** (22 mg, 49% yield) as a colorless solid. The spectroscopic properties of this compound were consistent with the data available in the literature.[30]

^{1}H NMR (400 MHz, $CDCl_3$) δ 7.43 – 7.35 (m, 8H), 7.34 – 7.29 (m, 2H), 3.28 (s, 2H), 2.77 (br s, 1H). **^{13}C NMR** (101 MHz, $CDCl_3$) δ 143.9, 128.7, 128.2, 125.8, 117.1, 76.6, 32.7.

3-hydroxy-3,4-dimethylpentanenitrile (51)

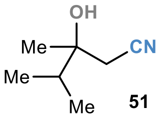
51

Prepared according to ***General Procedure D*** – using α-diazo-sulfone **1a** (40.0 mg, 0.20 mmol, 1 equiv.) as the nitrile oxide precursor, 2,3-dimethyl-1-butene (74.3 µL, 0.60 mmol, 3 equiv.) as the olefin substrate, and NEt_3 as the sacrificial reductant in the electrochemical step. The crude mixture was purified by flash column chromatography (*isocratic in* 70% Et_2O in hexane) to provide product **51** (11.0 mg, 43% yield) as a colorless oil. The title compound was previously unknown.

^{1}H NMR (500 MHz, $CDCl_3$) δ 2.56 (d, J = 16.6 Hz, 1H), 2.50 (d, J = 16.6 Hz, 1H), 1.89 (hept, J = 6.9 Hz, 1H), 1.73 (s, 1H), 1.30 (s, 3H), 0.98 (d, J = 6.9 Hz, 3H), 0.95 (d, J = 6.9 Hz, 3H). **^{13}C NMR** (126 MHz, $CDCl_3$) δ 117.9, 73.5, 37.0, 29.7, 23.4, 17.5, 17.0. **HRMS (ESI$^+$)** Mass calculated for $C_7H_{12}NONa$ $[M+Na]^+$: 149.0817, found: 149.0120.

2-(3-hydroxy-2-oxotetrahydrofuran-3-yl)acetonitrile (52)

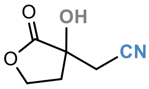
52

Prepared according to ***General Procedure E*** – using chloroxime **1b** (47.5 mg, 0.20 mmol, 1 equiv.) as the nitrile oxide precursor, α-methylene-γ-butyrolactone (21.0 µL, 0.24 mmol, 1.2 equiv.) as the olefin substrate, and Et_3N as the sacrificial reductant in the electrochemical step. In this case, the crude 1,3-DC mixture was filtered through a short pad of silica gel (*flushing first with 15% Et_2O in hexane to remove the remainder alkene and, then, with EtOAc to elute the cycloadduct intermediate*) prior submission to the electrolysis. Purification of the crude material by flash column chromatography (*isocratic in* 60% Et_2O in hexane) provided product **52** (16.2 mg, 57% yield) as a yellow oil. The title compound was previously unknown.

^{1}H NMR (400 MHz, $CDCl_3$) δ 4.55 (dt, J = 9.3, 7.2 Hz, 1H), 4.49 – 4.37 (m, 1H), 2.95 (d, J = 16.9 Hz, 1H), 2.81 (d, J = 16.9 Hz, 1H), 2.65 – 2.46 (m, 2H), 1.85 (s, 1H). **^{13}C NMR** (101 MHz, $CDCl_3$) δ 175.6, 115.3, 72.3, 65.8, 34.7, 26.4. **HRMS (ESI$^+$)** Mass calculated for $C_6H_7NO_3Na$ [M+Na]$^+$: 164.0318, found: 164.0316.

2-(2-*exo*-hydroxy-3-oxobicyclo[2.2.1]heptan-2-yl)acetonitrile (53)

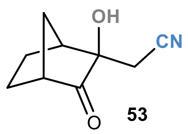

53

Prepared according to ***General Procedure E*** – using chloroxime **1b** (47.5 mg, 0.20 mmol, 1 equiv.) as the nitrile oxide precursor, 3-methylene-2-norbornanone (29.3 mg, 0.24 mmol, 1.2 equiv.) as the olefin substrate, and Et_3N as the sacrificial reductant in the electrochemical step. In this case, the crude 1,3-DC mixture was filtered through a short pad of silica gel (*flushing first with 15% Et_2O in hexane to remove the remainder alkene and, then, with EtOAc to elute the cycloadduct intermediate*) prior submission to the electrolysis. Purification of the crude material by flash column chromatography (*isocratic in* 60% Et_2O in hexane) provided product **53** (11 mg, 33% yield, *single diastereoisomer*) as a yellow oil. The spectroscopic properties of this compound were consistent with the data available in the literature.[31]

^{1}H NMR (400 MHz, $CDCl_3$) δ 2.78 – 2.71 (m, 1H), 2.72 (d, J = 17.0 Hz, 1H), 2.46 (d, J = 17.3 Hz, 1H), 2.34 (dt, J = 10.8, 2.0 Hz, 1H), 2.03 – 1.92 (m, 1H), 1.88 (dt, J = 13.1, 4.3 Hz, 1H), 1.68 – 1.47 (m, 5H). **^{13}C NMR** (101 MHz, $CDCl_3$) 213.2, 116.3, 76.1, 48.1, 44.6, 33.9, 24.2, 23.1, 22.7.

(2*R**,3*R**)-3,4-dihydroxy-2-(hydroxymethyl)butanenitrile (54)

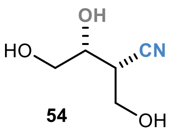

54

Prepared according to ***General Procedure E*** – using chloroxime **1b** (95 mg, 0.40 mmol, 1 equiv.) as the nitrile oxide precursor, *cis*-2-butene-1,4-diol (39.4 μL, 0.48 mmol, 1.2 equiv.) as the olefin substrate, and Et_3N as the sacrificial reductant in the electrochemical step. In this case, the crude 1,3-DC mixture was filtered through a short pad of silica gel (*flushing with EtOAc*) prior submission to the electrolysis. Purification of the crude material by flash column chromatography (*isocratic in* 30% acetone in Et_2O) provided product **54** (17 mg, 33% yield, *single diastereoisomer*) as a yellow oil. The title compound was previously unknown.

^{1}H NMR (400 MHz, CD_3OD) δ 3.90 – 3.83 (m, 3H), 3.73 (dd, J = 11.6, 4.0 Hz, 1H), 3.65 (dd, J = 11.5, 5.4 Hz, 1H), 3.34 – 3.28 (m, 3H), 2.97 (ddd, J = 7.3, 5.8, 5.0 Hz, 1H). **^{13}C NMR** (101 MHz, CD_3OD) δ 119.3, 69.1, 63.7, 58.6, 38.1.

(2*S**,3*R**)-3,4-dihydroxy-2-(hydroxymethyl)butanenitrile (55)

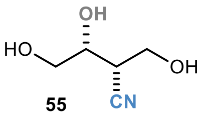

55

Prepared according to ***General Procedure E*** – using chloroxime **1b** (95 mg, 0.40 mmol, 1 equiv.) as the nitrile oxide precursor, *trans*-2-butene-1,4-diol (43 mg, 0.48 mmol, 1.2 equiv.) as the olefin substrate, and Et_3N as the

sacrificial reductant in the electrochemical step. In this case, the crude 1,3-DC mixture was filtered through a short pad of silica gel (*flushing with EtOAc*) prior submission to the electrolysis. Purification of the crude material by flash column chromatography (*isocratic in* 30% acetone in Et_2O) provided product **55** (22 mg, 42% yield, *single diastereoisomer*) as a yellow oil. The title compound was previously unknown.

^{1}H NMR (400 MHz, CD_3OD) δ 4.59 (br s, 1H), 3.90 – 3.75 (m, 3H), 3.65 (dd, *J* = 11.2, 5.7 Hz, 1H), 3.57 (dd, *J* = 11.2, 6.6 Hz, 1H), 3.34 – 3.28 (m, 2H), 3.06 (td, *J* = 6.8, 3.5 Hz, 1H). **^{13}C NMR** (101 MHz, CD_3OD) δ 118.6, 68.2, 63.5, 59.6, 38.4. **HRMS (ESI$^+$)** Mass calculated for $C_5H_9O_3NNa$ $[M+Na]^+$: 154.0475, found: 154.0471.

3-hydroxy-2-(hydroxymethyl)-3-methylbutanenitrile (56)

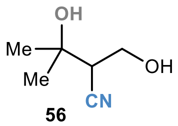
56

Prepared according to ***General Procedure E*** – using chloroxime **1b** (47.5 mg, 0.20 mmol, 1 equiv.) as the nitrile oxide precursor, 3-methyl-2-buten-1-ol (24.4 µL, 0.24 mmol, 1.2 equiv.) as the olefin substrate, and Et_3N as the sacrificial reductant in the electrochemical step. In this case, the crude 1,3-DC mixture was filtered through a short pad of silica gel (*flushing with EtOAc*) prior submission to the electrolysis. Purification of the crude material by flash column chromatography (*isocratic in* 100% Et_2O) provided product **56** (14 mg, 54% yield) as a colorless oil. The title compound was previously unknown.

^{1}H NMR (400 MHz, $CDCl_3$) δ 4.04 (d, *J* = 5.5 Hz, 2H), 3.07 (br. s, 1H), 2.84 (br. s, 1H), 2.81 (t, *J* = 5.4 Hz, 1H), 1.45 (s, 1H), 1.43 (s, 1H). **^{13}C NMR** (101 MHz, $CDCl_3$) δ 119.2, 71.9, 60.6, 45.3, 28.4, 27.6. **HRMS (ESI$^+$)** Mass calculated for $C_6H_{11}O_2NNa$ $[M+Na]^+$: 152.0682, found: 152.0677.

(2*R**,3*S**)-3-hydroxy-2-(2-hydroxyethyl)pentanenitrile + regioisomer (57)

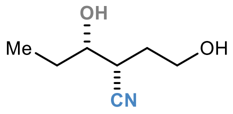
regioisomer 1

57 +

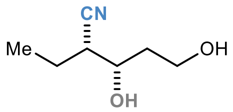
regioisomer 2

Prepared according to ***General Procedure E*** – using chloroxime **1b** (95 mg, 0.40 mmol, 1 equiv.) as the nitrile oxide precursor, *trans*-3-hexen-1-ol (58.8 µL, 0.48 mmol, 1.2 equiv.) as the olefin substrate, and Et_3N as the sacrificial reductant in the electrochemical step. In this case, the crude 1,3-DC mixture was filtered through a short pad of silica gel (*flushing with EtOAc*) prior submission to the electrolysis. Purification of the crude material by flash column chromatography (*isocratic in* 100% Et_2O) provided **57-regioisomer 1** (22 mg, 78% yield) as an amber oil. Continued elution delivered **57-regioisomer 2** (23 mg, 79% yield) as an amber oil. Both regioisomers of the title compound were previously unknown. The regioisomeric ratio was determined by ^{1}H NMR analysis of the crude reaction mixture.

Characterization Data for **57-regioisomer 1** – **^{1}H NMR** (400 MHz, $CDCl_3$) δ 3.92 (app q, *J* = 4.3 Hz, 1H), 3.85 (app tt, *J* = 7.2, 3.8 Hz, 1H), 3.68 – 3.59 (m, 1H), 2.97 (ddd, *J* = 7.8, 6.2, 3.0 Hz, 1H), 2.55

(d, J = 5.8 Hz, 1H), 2.10 – 1.92 (m, 3H), 1.81 – 1.63 (m, 2H), 1.03 (t, J = 7.4 Hz, 3H). **^{13}C NMR** (101 MHz, $CDCl_3$) δ 119.8, 72.7, 59.5, 36.0, 32.2, 28.7, 10.3. **HRMS (ESI$^+$)** Exact mass calculated for $C_7H_{13}O_2NNa$ [M+Na]$^+$: 166.0838, found: 166.0834.

Characterization Data for **57-regioisomer 2** – **^{1}H NMR** (400 MHz, $CDCl_3$) δ 4.05 – 3.93 (m, 2H), 3.87 (ddd, J = 11.0, 8.2, 3.6 Hz, 1H), 3.37 (d, J = 5.0 Hz, 1H), 2.56 (ddd, J = 9.7, 5.7, 4.0 Hz, 1H), 2.25 (d, J = 14.5 Hz, 1H), 1.93 (dddd, J = 13.9, 9.8, 8.2, 4.0 Hz, 1H), 1.82 – 1.71 (m, 3H), 1.12 (t, J = 7.4 Hz, 3H). **^{13}C NMR** (126 MHz, $CDCl_3$) δ 120.4, 70.3, 60.7, 41.5, 36.7, 22.3, 12.0. **HRMS (ESI$^+$)** Exact mass calculated for $C_7H_{13}O_2NNa$ [M+Na]$^+$: 166.0838, found: 166.0833.

(2*R,3*R**)-3-hydroxy-2-methyl-3-phenylpropanenitrile (58)**

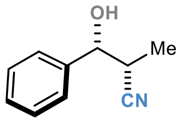

58

Prepared according to ***General Procedure E*** – using chloroxime **1b** (47.5 mg, 0.20 mmol, 1 equiv.) as the nitrile oxide precursor, *trans*-β-methylstyrene (31.1 μL, 0.24 mmol, 1.2 equiv.) as the olefin substrate, and Et_3N as the sacrificial reductant in the electrochemical step. In this case, the crude 1,3-DC mixture was filtered through a short pad of silica gel (*flushing first with 15% Et_2O in hexane to remove the remainder alkene and, then, with EtOAc to elute the cycloadduct intermediate*) prior submission to the electrolysis. Purification of the crude material by flash column chromatography (*isocratic in* 50% Et_2O in hexane) provided product **58** (21.1 mg, 65% yield, *single diastereoisomer*) as a colorless oil. The spectroscopic properties of this compound were consistent with the data available in the literature.[28]

^{1}H NMR (400 MHz, $CDCl_3$) δ 7.43 – 7.32 (m, 5H), 4.72 (dd, J = 6.6, 3.6 Hz, 1H), 2.92 (app p, J = 7.1 Hz, 1H), 2.47 (d, J = 3.6 Hz, 1H), 1.25 (d, J = 7.2 Hz, 3H). **^{13}C NMR** (101 MHz, $CDCl_3$) δ 140.1, 129.0, 128.9, 126.4, 121.1, 75.3, 34.7, 14.9.

(1*R,2*S**)-1-hydroxy-2,3-dihydro-1*H*-indene-2-carbonitrile (59)**

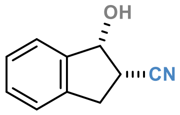

59

Prepared according to ***General Procedure E*** – using chloroxime **1b** (47.5 mg, 0.20 mmol, 1 equiv.) as the nitrile oxide precursor, indene (28.0 μL, 0.24 mmol, 1.2 equiv.) as the olefin substrate, and Et_3N as the sacrificial reductant in the electrochemical step. In this case, the crude 1,3-DC mixture was filtered through a short pad of silica gel (*flushing first with 15% Et_2O in hexane to remove the remainder alkene and, then, with EtOAc to elute the cycloadduct intermediate*) prior submission to the electrolysis. Purification of the crude material by flash column chromatography (*isocratic in* 70% Et_2O in hexane) provided product **59** (22.4 mg, 70% yield, *single diastereoisomer*) as a yellow oil. The spectroscopic properties of this compound were consistent with the data available in the literature.[28]

^{1}H NMR (400 MHz, $CDCl_3$) δ 7.50 – 7.46 (m, 1H), 7.40 – 7.29 (m, 3H), 5.35 (d, *J* = 5.4 Hz, 1H), 3.53 – 3.41 (m, 2H), 3.34 – 3.23 (m, 1H), 2.45 (s, 1H). **^{13}C NMR** (101 MHz, $CDCl_3$) δ 141.2, 139.7, 129.7, 127.9, 125.1 (*2 signals*), 119.4, 75.2, 37.0, 34.4.

(1*R,3*S**,4*S**)-3-((*tert*-butyldimethylsilyl)oxy)-4-cyano-*N*-methoxy-*N*-methylcyclopentane-1-carboxamide (60)**

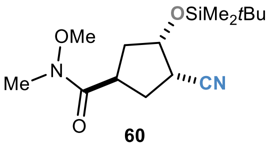

60

Prepared according to ***General Procedure E*** – using chloroxime **1b** (47.5 mg, 0.20 mmol, 1 equiv.) as the nitrile oxide precursor, *N*-methoxy-*N*-methylcyclopent-3-ene-1-carboxamide (37.3 mg, 0.24 mmol, 1.2 equiv.) as the olefin substrate, and Et_3N as the sacrificial reductant in the electrochemical step. Due to its high polarity, the hydroxy nitrile product was converted into the corresponding silyl ether, prior purification. This was achieved by concentrating the crude electrolysis mixture, adding a solution of *tert*-butyldimethylsilyl chloride (90.4 mg, 0.60 mmol, 3 equiv.) and imidazole (54.5 mg, 0.80 mmol, 4 equiv.) in DMF (0.6 mL), stirring the resulting mixture for 2 hours and concentrating again in vacuo. Purification of the crude material by flash column chromatography (*isocratic in* 100% Et_2O) provided product **60** (27 mg, 43% yield, *single diastereoisomer*) as a colorless oil. The title compound was previously unknown. The relative stereochemistry of compound **60** has been inferred by ^{1}H NOESY NMR analysis.

^{1}H NMR (500 MHz, $CDCl_{3)}$ δ 4.48 (app q, *J* = 3.8 Hz, 1H), 3.70 (s, 3H), 3.61 – 3.49 (m, 1H), 3.18 (s, 3H), 2.94 (td, *J* = 8.8, 4.2 Hz, 1H), 2.34 – 2.26 (m, 2H), 2.04 – 1.94 (m, 2H), 0.92 (s, 9H), 0.15 (s, 3H), 0.11 (s, 3H). **^{13}C NMR** (126 MHz, $CDCl_3$) δ 175.9, 119.8, 74.1, 61.5, 38.7, 37.0, 36.6, 32.4, 31.5, 25.7, 18.1, –4.8 (2 signals). **HRMS (APCI$^+$)** Mass calculated for $C_{15}H_{29}O_3N_2Si$ $[M+H]^+$: 313.1929, found: 313.1934.

(1*S,6*S**)-6-hydroxycyclohex-3-ene-1-carbonitrile (61)**

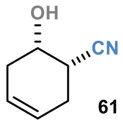

61

Prepared according to ***General Procedure E*** – using chloroxime **1b** (47.5 mg, 0.20 mmol, 1 equiv.) as the nitrile oxide precursor, 1,4-cyclohexadiene (22.7 μL, 0.24 mmol, 1.2 equiv.) as the olefin substrate, and 1,4-cyclohexadiene as the sacrificial reductant in the electrochemical step. In this case, the crude 1,3-DC mixture was filtered through a short pad of silica gel (*flushing first with 15% Et_2O in hexane to remove the remainder alkene and, then, with EtOAc to elute the cycloadduct intermediate*) prior submission to the electrolysis. Purification of the crude material by flash column chromatography (*isocratic in* 80% Et_2O in hexane) provided product **61** (6 mg, 26% yield, *single diastereoisomer*) as a colorless oil. The title compound was previously unknown.

^{1}H NMR (400 MHz, $CDCl_3$) δ 5.74 – 5.60 (m, 2H), 4.12 (ddd, *J* = 7.7, 5.0, 3.1 Hz, 1H), 3.04 (tdd, *J* = 5.7, 3.2, 1.0 Hz, 1H), 2.60 – 2.37 (m, 3H), 2.37 – 2.26 (m, 1H), 2.19 (s, 1H). **^{13}C NMR** (101 MHz, $CDCl_3$)

δ 124.4, 123.0, 120.5, 65.9, 32.7, 31.7, 26.6. **HRMS (ESI⁻)** Mass calculated for C_7H_8ON $[M–H]^-$: 122.0611, found: 122.0601.

2-(4-bromophenyl)-6-hydroxy-1,3-dioxooctahydro-1*H*-isoindole-5-carbonitrile (62)

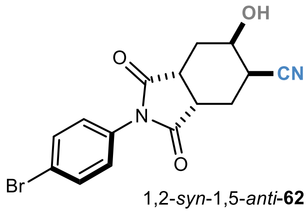

1,2-*syn*-1,5-*anti*-**62**

+

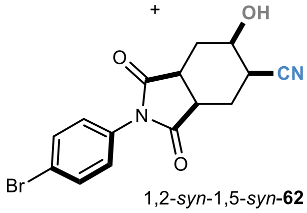

1,2-*syn*-1,5-*syn*-**62**

Prepared according to ***General Procedure D*** – using α-diazo-sulfone **1a** (80 mg, 0.40 mmol, 2 equiv.) as the nitrile oxide precursor, *cis*-1,2,3,6-*N*-(4-bromophenyl)-tetrahydrophthalimide (91 mg, 0.20 mmol, 1 equiv.) as the olefin substrate, and NEt_3 as the sacrificial reductant in the electrochemical step. In this case, the crude 1,3-DC mixture was filtered through a short pad of silica gel (*flushing first with 15% Et_2O in hexane to remove the remainder alkene and, then, with EtOAc to elute the cycloadduct intermediate*) prior submission to the electrolysis. Purification of the crude material by flash column chromatography (*gradient from* 50% Et_2O in hexane *to* 100% Et_2O) provided diastereoisomer **1,2-*syn*-1,5-*anti*-62** (12 mg, 21% yield) as a colorless solid. Continued elution delivered diastereoisomer **1,2-*syn*-1,5-*syn*-62** (13 mg, 23% yield) as a colorless solid. Both diastereoisomers of the title compound were previously unknown. The diastereoisomeric ratio was determined by ^{1}H NMR analysis of the crude reaction mixture. The relative stereochemistry of both diastereoisomers has been inferred by X-ray crystallographic analysis on isomer **1,2-*syn*-1,5-*anti*-62** (*see Section S8.2*).

Characterization Data for **1,2-*syn*-1,5-*anti*-62** – **^{1}H NMR** (400 MHz, $CDCl_3$) δ 7.66 – 7.56 (m, 2H), 7.22 – 7.13 (m, 2H), 4.10 (dt, *J* = 7.0, 3.6 Hz, 1H), 3.34 (app q, *J* = 7.5 Hz, 1H), 3.24 (app q, *J* = 6.9 Hz, 1H), 2.90 – 2.82 (m, 1H), 2.69 (d, *J* = 4.3 Hz, 1H), 2.48 (ddd, *J* = 14.1, 8.8, 6.9 Hz, 1H), 2.30 (dd, *J* = 14.3, 7.2 Hz, 1H), 2.21 (ddd, *J* = 14.6, 6.3, 4.6 Hz, 1H), 1.99 (ddd, *J* = 14.3, 7.6, 3.3 Hz, 1H). **^{13}C NMR** (101 MHz, $CDCl_3$) δ 176.9, 176.0, 132.4, 130.5, 127.5, 122.6, 119.0, 64.7, 37.7, 37.4, 32.4, 29.8, 22.7. **HRMS (ESI⁻)** Exact mass calculated for $C_{15}H_{12}O_3N_2Br$ $[M-H^+]$: 347.0037, found: 347.0031.

Characterization Data for **1,2-*syn*-1,5-*syn*-62** – **^{1}H NMR** (400 MHz, $CDCl_3$) δ 7.64 – 7.55 (m, 2H), 7.23 – 7.13 (m, 2H), 4.14 – 4.04 (m, 1H), 3.34 (app q, *J* = 7.5 Hz, 1H), 3.24 (app q, *J* = 6.9 Hz, 1H), 2.86 (ddd, *J* = 8.8, 4.3, 2.9 Hz, 1H), 2.70 – 2.61 (m, 1H), 2.48 (ddd, *J* = 14.4, 8.9, 6.9 Hz, 1H), 2.29 (app dt, *J* = 14.4, 7.2 Hz, 1H), 2.21 (ddd, *J* = 14.4, 6.3, 4.5 Hz, 1H), 1.99 (ddd, *J* = 14.6, 7.7, 4.6 Hz, 1H). **^{13}C NMR**(101 MHz, $CDCl_3$) δ 175.0, 174.1, 130.5, 128.6, 125.6, 120.6, 117.1, 62.8, 35.8, 35.5, 30.4, 27.9, 20.8. **HRMS** (ESI⁻) Exact mass calculated for $C_{15}H_{12}O_3N_2Br$ $[M–H]^-$: 347.0037, found: 347.0032.

2-hydroxytetrahydro-2*H*-pyran-3-carbonitrile (63)

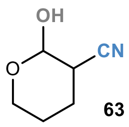

63

Prepared according to ***General Procedure E*** – using chloroxime **1b** (47.5 mg, 0.20 mmol, 1 equiv.) as the nitrile oxide precursor, 3,4-dihydro-2*H*-pyran (21.9 µL, 0.24 mmol, 1.2 equiv.) as the olefin substrate, and Et_3N as the sacrificial

reductant in the electrochemical step. In this case, the crude 1,3-DC mixture was filtered through a short pad of silica gel (*flushing first with 15% Et_2O in hexane to remove the remainder alkene and, then, with EtOAc to elute the cycloadduct intermediate*) prior submission to the electrolysis. Purification of the crude material by flash column chromatography (*isocratic in* 70% EtOAc in hexane, *SiO_2 neutralized with 2% Et_3N prior loading*) provided product **63** (16 mg, 67% yield, *isolated as a 1:1 mixture of diastereoisomers*) as a colorless oil. The title compound was previously unknown. The diastereoisomeric ratio was determined by ^{1}H NMR analysis of the crude reaction mixture.

^{1}H NMR (400 MHz, $CDCl_3$) δ 5.09 (d, *J* = 2.7 Hz, 1H), 5.01 (d, *J* = 5.5 Hz, 1H), 4.12 – 4.01 (m, 2H), 3.66 – 3.54 (m, 2H), 3.19 (s, 2H), 2.92 (ddd, *J* = 8.4, 4.3, 2.7 Hz, 1H), 2.66 (ddd, *J* = 8.6, 5.6, 4.4 Hz, 1H), 2.31 – 2.19 (m, 2H), 1.96 – 1.83 (m, 2H), 1.83 – 1.74 (m, 2H), 1.65 – 1.56 (m, 2H). **^{13}C NMR** (101 MHz, $CDCl_3$) δ 119.5, 119.1, 93.3, 91.2, 63.8, 62.5, 33.8, 33.7, 24.3, 23.3, 22.9, 22.8.

(3*R,4*R**)-4-hydroxytetrahydrofuran-3-carbonitrile (64)**

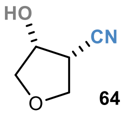

64

Prepared according to ***General Procedure E*** – using chloroxime **1b** (47.5 mg, 0.20 mmol, 1 equiv.) as the nitrile oxide precursor, 2,5-dihydrofuran (18.1 μL, 0.24 mmol, 1.2 equiv.) as the olefin substrate, and Et_3N as the sacrificial reductant in the electrochemical step. In this case, the crude 1,3-DC mixture was filtered through a short pad of silica gel (*flushing first with 15% Et_2O in hexane to remove the remainder alkene and, then, with EtOAc to elute the cycloadduct intermediate*) prior submission to the electrolysis. Purification of the crude material by flash column chromatography (*isocratic in* Et_2O) provided product **64** (11.2 mg, 49% yield, *single diastereoisomer*) as an amber oil. The title compound was previously unknown.

^{1}H NMR (400 MHz, $CDCl_3$) δ 4.68 – 4.59 (m, 1H), 4.16 (t, *J* = 8.3 Hz, 1H), 4.05 – 3.98 (m, 2H), 3.84 (dd, *J* = 10.1, 2.3 Hz, 1H), 3.15 – 3.08 (m, 1H), 2.93 – 2.87 (m, 1H). **^{13}C NMR** (101 MHz, $CDCl_3$) δ 116.9, 75.2, 71.5, 68.6, 37.2. **HRMS (ESI$^+$)** Exact mass calculated for $C_5H_7O_2NNa$ $[M+Na]^+$: 136.0374, found: 136.0396.

dimethyl (1*S,2*R**,3*S**,4*S**,5*R**,6*R**)-5-((*tert*-butyldimethylsilyl)oxy)-6-cyano-7-oxabicyclo [2.2.1]heptane-2,3-dicarboxylate (65)**

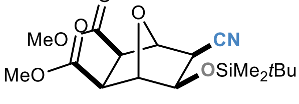

65

Prepared according to ***General Procedure D*** – using α-diazo-sulfone **1a** (40.0 mg, 0.20 mmol, 1 equiv.) as the nitrile oxide precursor, methyl *exo*-3,6-epoxy-1,2,3,6-tetrahydrophthalate (127 mg, 0.60 mmol, 3 equiv.) as the olefin substrate, and Et_3N as the sacrificial reductant in the electrochemical step. Due to its high polarity, the hydroxy nitrile product was converted into the corresponding silyl ether, prior purification. This was achieved by concentrating the crude electrolysis mixture, adding a solution of *tert*-butyldimethylsilyl chloride (90.4 mg, 0.60 mmol, 3 equiv.) and imidazole (54.5 mg, 0.80

mmol, 4 equiv.) in DMF (0.6 mL), stirring the resulting mixture for 2 hours and concentrating again in vacuo. Purification of the crude material by flash column chromatography (*isocratic in* 100% Et_2O) provided product **65** (50 mg, 68% yield, *single diastereoisomer*) as a colorless solid. The title compound was previously unknown. The relative stereochemistry of compound **65** has been inferred by ^1H NOESY NMR analysis.

^{1}H NMR (500 MHz, $CDCl_3$) δ 5.13 (d, J = 1.4 Hz, 1H), 4.71 (d, J = 1.4 Hz, 1H), 4.13 (d, J = 6.5 Hz, 1H), 3.70 (s, 3H), 3.70 (s, 3H), 2.98 (d, J = 6.5 Hz, 1H), 2.91 (d, J = 9.5 Hz, 1H), 2.83 (d, J = 9.4 Hz, 1H), 0.94 (s, 9H), 0.15 (s, 3H), 0.14 (s, 3H). **^{13}C NMR** (126 MHz, $CDCl_3$) δ 170.0, 169.7, 116.1, 84.5, 80.5, 74.5, 52.6 (2 signals), 50.5, 46.7, 43.1, 25.7, 18.2, –4.8 (2 signals). **HRMS (ESI$^+$)** Mass calculated for $C_{17}H_{27}NO_6Si$ $[M+Na]^+$: 369.1608, found: 369.1612.

(10*S**,11*R**)-11-hydroxy-10,11-dihydro-5*H*-dibenzo[*b,f*]azepine-10-carbonitrile (66)

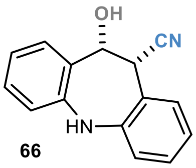

66

Prepared according to ***General Procedure E*** – using chloroxime **1b** (47.5 mg, 0.20 mmol, 1 equiv.) as the nitrile oxide precursor, 5*H*-dibenz[*b,f*]azepine (46.4 mg, 0.24 mmol, 1.2 equiv.) as the olefin substrate, and Et_3N as the sacrificial reductant in the electrochemical step. *N.B. 5H-dibenz[b,f]azepine is poorly soluble in EtOAc, thus the 1,3-DC mixture has to be sonicated, prior heating, to ensure complete dissolution of the alkene substrate*. In this case, the crude 1,3-DC mixture was filtered through a short pad of silica gel (*flushing first with 15% Et_2O in hexane to remove the remainder alkene and, then, with EtOAc to elute the cycloadduct intermediate*) prior submission to the electrolysis. Purification of the crude material by flash column chromatography (*isocratic in* 50% Et_2O in hexane, *SiO_2 neutralized with 2% Et_3N prior loading*) provided product **66** (25 mg, 53% yield, *single diastereoisomer*) as a yellow solid. The title compound was previously unknown.

^{1}H NMR (400 MHz, $CDCl_3$) δ 7.56 (d, J = 7.7 Hz, 1H), 7.42 (dd, J = 7.8, 1.6 Hz, 1H), 7.30 – 7.20 (m, 2H), 7.07 (app td, J = 7.5, 1.2 Hz, 1H), 7.00 – 6.90 (m, 2H), 6.84 (dd, J = 8.1, 1.2 Hz, 1H), 6.08 (s, 1H), 5.37 (d, J = 7.3 Hz, 1H), 4.51 (d, J = 1.5 Hz, 1H), 2.12 (d, J = 8.3 Hz, 1H). **^{13}C NMR** (101 MHz, $CDCl_3$) δ 142.0, 140.9, 131.3, 130.5, 129.6, 129.4, 125.3, 122.4, 120.6, 119.5, 119.2, 118.9, 118.7, 72.4, 42.1. **HRMS (ESI$^+$)** Mass calculated for $C_{15}H_{12}ON_2Na$ $[M+Na]^+$: 259.0842, found: 259.0841.

(1*S*,2*S*,3*S*,5*S*)-2-hydroxy-2,6,6-trimethylbicyclo[3.1.1]heptane-3-carbonitrile (67)

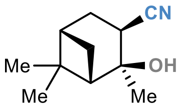

67

Prepared according to ***General Procedure E*** – using chloroxime **1b** (47.5 mg, 0.20 mmol, 1 equiv.) as the nitrile oxide precursor, (+)-α-pinene (38.1 μL, 0.24 mmol, 1.2 equiv.) as the olefin substrate, and Et_3N as the sacrificial reductant in the electrochemical step. In this case, the crude 1,3-DC mixture was filtered through a short pad of silica gel (*flushing first with 15% Et_2O in hexane to remove the remainder alkene and, then, with EtOAc to elute the cycloadduct intermediate*) prior submission to the electrolysis. Purification of the crude material by flash

column chromatography (*isocratic in* 20% EtOAc in hexane) provided product **67** (27 mg, 76% yield, *single enantiomer*) as a yellow oil. The title compound was previously unknown. The stereochemistry of compound **67** has been inferred by ^{1}H NOESY NMR analysis.

^{1}H NMR (400 MHz, $CDCl_3$) δ 3.16 (dd, *J* = 10.5, 7.5 Hz, 1H), 2.39 – 2.26 (m, 2H), 2.20 (ddd, *J* = 13.5, 7.5, 2.3 Hz, 1H), 2.10 (s, 1H), 2.07 – 2.00 (m, 2H), 1.54 (d, *J* = 10.7 Hz, 1H), 1.42 (s, 3H), 1.30 (s, 3H), 0.96 (s, 3H). **^{13}C NMR** (101 MHz, $CDCl_3$) δ 121.0, 73.0, 53.5, 39.8, 38.4, 33.5, 31.5, 30.6, 27.9, 27.8, 23.2. **HRMS (ESI$^+$)** Exact mass calculated for $C_{11}H_{17}ONNa$ $[M+Na]^+$: 202.1202, found: 216.0995.

3-hydroxy-3-((*S*)-4-methyl-5-oxocyclohex-3-en-1-yl)butanenitrile (68)

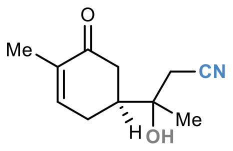

68

Prepared according to ***General Procedure E*** – using chloroxime **1b** (47.5 mg, 0.20 mmol, 1 equiv.) as the nitrile oxide precursor, (+)-carvone (37.6 µL, 0.24 mmol, 1.2 equiv.) as the olefin substrate, and Et_3N as the sacrificial reductant in the electrochemical step. In this case, the crude 1,3-DC mixture was filtered through a short pad of silica gel (*flushing first with 15% Et_2O in hexane to remove the remainder alkene and, then, with EtOAc to elute the cycloadduct intermediate*) prior submission to the electrolysis. Purification of the crude material by flash column chromatography (*isocratic in* Et_2O) provided product **68** (10 mg, 27% yield, *isolated as a 1:1 mixture of diastereoisomers*) as a yellow oil. The title compound was previously unknown. The diastereoisomeric ratio was assigned by ^{13}C NMR analysis of the purified product.

^{1}H NMR (400 MHz, $CDCl_3$) δ 6.85 – 6.69 (m, 1H), 2.68 – 2.52 (m, 3H), 2.47 – 2.24 (m, 4H), 1.97 – 1.86 (m, 1H), 1.79 (s, 3H), 1.41 (s, 3H). **^{13}C NMR** (101 MHz, $CDCl_3$) δ 199.4, 199.2, 144.7, 144.2, 135.6, 135.4, 117.4 (2 signals), 71.8, 71.7, 44.0, 43.9, 39.1, 38.6, 29.8 (2 signals), 27.1, 26.5, 24.5, 24.2, 15.6 (2 signals). **HRMS (ESI$^+$)** Exact mass calculated for $C_{11}H_{15}O_2NNa$ $[M+Na]^+$: 216.0995, found: 216.0988.

(3*R*,3a*R*,5*R*,6*R*,7*R*,8a*S*)-6-hydroxy-3,6,8,8-tetramethyloctahydro-1*H*-3a,7-methanoazulene-5-carbonitrile (69)

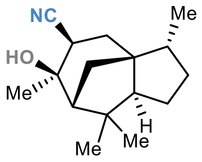

69

Prepared according to ***General Procedure E*** – using chloroxime **1b** (47.5 mg, 0.20 mmol, 1 equiv.) as the nitrile oxide precursor, (–)-α-cedrene (52.6 µL, 0.24 mmol, 1.2 equiv.) as the olefin substrate, and Et_3N as the sacrificial reductant in the electrochemical step. In this case, the crude 1,3-DC mixture was filtered through a short pad of silica gel (*flushing first with 15% Et_2O in hexane to remove the remainder alkene and, then, with EtOAc to elute the cycloadduct intermediate*) prior submission to the electrolysis. Purification of the crude material by flash column chromatography (*isocratic in* 80% Et_2O in hexane) provided product **69** (11 mg, 21% yield, *single enantiomer*) as a colorless solid. The title compound was

previously unknown. The absolute stereochemistry of compound **69** has been inferred by X-ray crystallographic analysis (*see Section S8.3*).

^{1}H NMR (400 MHz, $CDCl_3$) δ 2.79 (dd, *J* = 12.0, 6.1 Hz, 1H), 2.07 – 2.01 (m, 1H), 1.96 – 1.89 (m, 1H), 1.86 (d, *J* = 12.2 Hz, 1H), 1.80 – 1.70 (m, 3H), 1.67 – 1.58 (m, 3H), 1.52 (s, 3H), 1.47 – 1.37 (m, 1H), 1.36 – 1.24 (m, 2H), 1.13 (s, 3H), 1.03 (s, 3H), 0.88 (d, *J* = 7.1 Hz, 3H). **^{13}C NMR** (101 MHz, $CDCl_3$) δ 120.9, 71.9, 60.6, 56.7, 53.0, 41.6, 41.5, 38.5, 36.9, 36.8, 35.1, 29.0, 28.8, 28.1, 25.4, 15.3. **HRMS (ESI)** Mass calculated for $C_{16}H_{25}ON$ $[M+H]^+$: 247.1936, found: 247.1931.

2-((1*R*,4*R*,6*S*,9*R*,10*S*)-9-hydroxy-4,12,12-trimethyl-5-oxatricyclo[8.2.0.04,6]dodecan-9-yl)-acetonitrile (70)

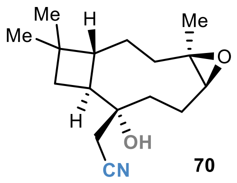

70

Prepared according to ***General Procedure E*** – using chloroxime **1b** (47.5 mg, 0.20 mmol, 1 equiv.) as the nitrile oxide precursor, (–)-caryophillene oxide (52.9 mg, 0.24 mmol, 1.2 equiv.) as the olefin substrate, and 1,4-cyclohexadiene as the sacrificial reductant in the electrochemical step. In this case, the crude 1,3-DC mixture was filtered through a short pad of silica gel (*flushing first with 15% Et_2O in hexane to remove the remainder alkene and, then, with EtOAc to elute the cycloadduct intermediate*) prior submission to the electrolysis. Purification of the crude material by flash column chromatography (*isocratic in* 80% Et_2O in hexane) provided product **70** (35 mg, 47% yield, *single enantiomer*) as a colorless solid. The title compound was previously unknown. The stereochemistry of compound **70** has been inferred by ^{1}H NOESY NMR analysis.

^{1}H NMR (400 MHz, $CDCl_3$) δ 3.59 – 3.50 (m, 1H), 2.35 (d, *J* = 16.3 Hz, 1H), 2.30 (d, *J* = 16.3 Hz, 1H), 2.19 – 2.10 (m, 1H), 2.05 (td, *J* = 11.4, 7.3 Hz, 1H), 1.95 – 1.88 (m, 2H), 1.86 – 1.77 (m, 3H), 1.74 – 1.62 (m, 2H), 1.60 – 1.53 (m, 2H), 1.50 – 1.40 (m, 1H), 1.31 – 1.24 (m, 1H), 1.24 (s, 3H), 1.04 (s, 3H), 1.03 (s, 3H). **^{13}C NMR** (101 MHz, $CDCl_3$) δ 117.8, 78.9, 73.3, 70.1, 48.0, 47.7, 38.7, 36.6, 35.3, 31.7, 30.4, 27.9, 25.4, 23.5, 22.5, 20.5. **HRMS (ESI)** Mass calculated for $C_{16}H_{24}O_2N$ $[M+H]^+$: 262.1810, found: 262.1813.

(1*S*,3*R*,5*S*,6*R*,7*R*,8*S*,8a*R*)-6-cyano-5-hydroxy-8-(2-((2*R*,4*R*)-4-hydroxy-6-oxotetrahydro-2*H*-pyran-2-yl)ethyl)-3,7-dimethyl-1,2,3,5,6,7,8,8a-octahydronaphthalen-1-yl 2,2-dimeth-ylbutanoate (71)

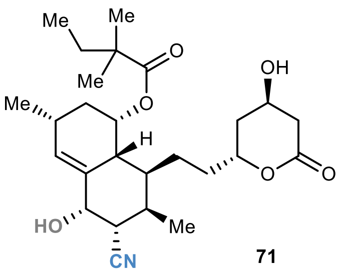

71

Prepared according to ***General Procedure E*** – using chloroxime **1b** (47.5 mg, 0.20 mmol, 1 equiv.) as the nitrile oxide precursor, simvastatin (100 mg, 0.24 mmol, 1.2 equiv.) as the olefin substrate, and 1,4-cyclohexadiene as the sacrificial reductant in the electrochemical step. In this case, the crude 1,3-DC mixture was filtered through a short pad of silica gel (*flushing first with 50% Et_2O in hexane to remove the remainder alkene and, then, with*

EtOAc to elute the cycloadduct intermediate) prior submission to the electrolysis. Purification of the crude material by flash column chromatography (*isocratic in* 10% MeOH in Et_2O) provided product **71** (19 mg, 22% yield, *single enantiomer*) as a colorless solid. The title compound was previously unknown. The stereochemistry of compound **71** has been inferred by ^{1}H NOESY NMR analysis.

^{1}H NMR (400 MHz, $CDCl_3$) δ 5.97 (dt, *J* = 4.6, 2.1 Hz, 1H), 5.37 (td, *J* = 4.4, 2.6 Hz, 1H), 4.60 (ddt, *J* = 11.2, 7.6, 4.0 Hz, 1H), 4.45 – 4.30 (m, 2H), 3.17 (dd, *J* = 5.3, 2.9 Hz, 1H), 2.73 (dd, *J* = 17.6, 5.0 Hz, 1H), 2.63 (ddd, *J* = 17.7, 3.7, 1.6 Hz, 1H), 2.49 – 2.35 (m, 2H), 2.25 – 2.17 (m, 2H), 2.11 (d, *J* = 9.2 Hz, 1H), 2.07 – 1.90 (m, 3H), 1.87 (dd, *J* = 7.6, 2.6 Hz, 1H), 1.80 – 1.68 (m, 2H), 1.67 – 1.52 (m, 3H), 1.50 – 1.38 (m, 1H), 1.38 – 1.27 (m, 1H), 1.19 (s, 3H), 1.17 (s, 3H), 1.16 (d, *J* = 7.2 Hz, 3H), 1.05 (d, *J* = 7.3 Hz, 3H), 0.84 (t, *J* = 7.5 Hz, 3H). **^{13}C NMR** (101 MHz, $CDCl_3$) δ 177.9, 170.2, 132.2, 127.4, 119.1, 76.1, 68.3, 66.1, 62.6, 43.3, 43.1, 39.1, 38.6, 36.4, 36.2, 33.2, 33.0, 32.2, 31.9, 27.6, 25.1, 24.8, 24.8, 22.4, 12.2, 9.4. **HRMS (ESI$^+$)** Mass calculated for $C_{26}H_{39}O_6NNa$ $[M+H]^+$: 484.2670, found: 484.2665.

S.7 – Synthetic Applications

S.7.1 – Scale-Up of the Telescoped Process Between 1b and Styrene to Give 48

S.7.1.1 – *Adjustment of Current Intensity:* In order to maintain constant the current density (*J*) within the electrochemical cell, the current intensity (*i*) for the electrochemical step run on a **3.2 mmol scale** was adjusted, according to the following calculations:

■ *Determination of the **J (A•m^{-2})** value for the electrolysis in **General Procedure E** (0.2 mmol, i = –15 mA, Q = 6.0 F/mol):*

▸ Dimensions of IKA microelectrodes: **3 x 56 x 1 mm** (W, H, D)

▸ Submerged electrodes height: **29 mm**

▸ Total surface area of submerged electrodes [A (m^2)]:

[(29 mm x 3 mm) x 2] + [(29 mm x 1 mm) x 2] + (3 mm x 1 mm) = 235 mm^2 = **2.35 x 10^{-4} m^2**

▸ ***J* (A•m^{-2}) =** *I* (A) / A (m^2) = 0.015 A / 2.35 x 10^{-4} m^2 = **63.8 A•m^{-2}**

■ *Determination of the **i (mA)** value for the electrolysis at **3.2 mmol scale** (Q = 6.0 F/mol):*

▸ Dimensions of IKA electrodes: **8 x 52.5 x 2 mm** (W, H, D)

▸ Submerged electrodes height: **37 mm**

▸ Total surface area of submerged electrodes [A (m^2)]:

[(37 mm x 8 mm) x 2] + [(37 mm x 2 mm) x 2] + (8 mm x 2 mm) = 756 mm^2 = **7.56 x 10^{-4} m^2**

▸ ***I* (A) =** *J* (A•m^{-2}) x A (m^2) = 63.8 A•m^{-2} x 7.56 x 10^{-4} m^2 = 0.048 A → ***i* = –48 mA**

S.7.1.2 – *Synthesis of Product **48** on a 3.2 mmol Scale:*

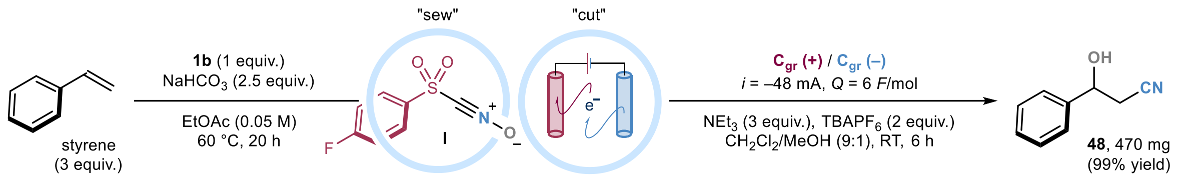

To a vigorously stirring solution of $NaHCO_3$ (672 mg, 8.00 mmol) and styrene (1.22 mL, 9.60 mmol) in EtOAc (64 mL), at room temperature, was added chloroxime **1b** (760 mg, 3.20 mmol) in one portion. The resulting mixture was then heated to 60 °C, and stirred for 20 hours. After this time, the reaction mixture was allowed to cool down to room temperature and then concentrated under reduced pressure. The crude material was dissolved in a 9:1 mixture of CH_2Cl_2 and MeOH (16 mL), and transferred into a 20 mL IKA ElectraSyn® vial – fitted with carbon graphite electrodes [*dimensions (W, H, D)* = 8 x 52.5 x 2 mm, both working and counter electrode] – containing $TBAPF_6$ (2.48 g, 6.40 mmol) and Et_3N (1.34 mL, 9.6 mmol). The vial was sealed and mounted on an IKA ElectraSyn® station. The electrolysis was conducted under constant current regime (–48 mA), with a total charge (*Q*) of 6 F/mol. Completed the electrolysis, the crude mixture was concentrated under reduced pressure, and the residue was purified by flash column chromatography (*isocratic in* 80% Et_2O in pentane) to afford hydroxy nitrile **48** (470 mg, 99% yield) as a pale yellow oil. The spectroscopic properties of the obtained product matched those reported in *Section S.6.2*.

Reaction set-up for 1,3-DC *sew* step (*left*) and electrochemical *cut* step (*right*).

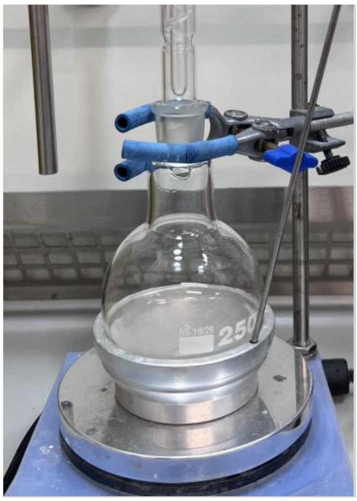

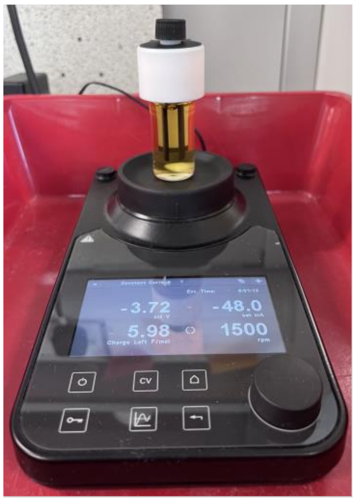

S.7.2 – Synthetic Route to Fluoxetine

S.7.2.1 – *Synthesis of **Seproxetine** (**S-7**)*

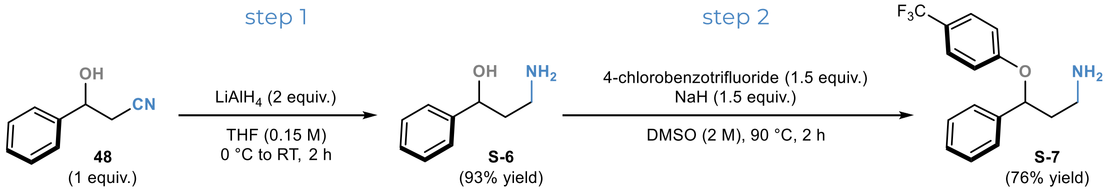

■ **Step 1** – To a suspension of $LiAlH_4$ (53.9 mg, 1.35 mmol) in THF (6 mL), at 0 °C, was added hydroxy nitrile **48** (132 mg, 0.90 mmol), in one portion. The resulting mixture was allowed to warm up to room temperature, and stirred for 2 hours. After this time, the reaction was quenched by cautious addition of water (5 mL) and extracted with EtOAc (3 x 10 mL). The combined organic layers were washed with brine, dried over $MgSO_4$ and filtered. Removal of the solvent under reduced pressure delivered γ-amino alcohol **S-6** (127 mg, 93% yield) as a colorless solid. This material was used in the next step of the synthesis without any further purification procedure. The spectroscopic properties of this compound were consistent with the data available in the literature.[32]

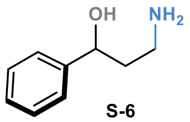

S-6

^{1}H NMR (400 MHz, $CDCl_3$) δ 7.39 – 7.29 (m, 4H), 7.28 – 7.20 (m, 1H), 4.89 (dt, J = 7.4, 3.2 Hz, 1H), 3.19 – 2.77 (m, 5H), 1.90 – 1.67 (m, 2H). **^{13}C NMR** (101 MHz, $CDCl_3$) δ 145.2, 128.3, 127.0, 125.7, 75.0, 40.3, 40.1.

■ **Step 2** – To a solution of γ-amino alcohol **S-6** (147 mg, 0.97 mmol) in DMSO (0.3 mL) was added NaH (*60% dispersion in mineral oil*, 58.2 mg, 1.45 mmol). The resulting mixture was heated to 100 °C, and stirred for 30 minutes. After this time, a solution of 4-chlorobenzotrifluoride (263 mg, 1.45 mmol) in DMSO (0.2 mL) was added to the mixture, and the reaction was stirred at 100 °C for 20 hours. The reaction was quenched by addition of water (2 mL), and extracted with CH_2Cl_2 (3 x 5 mL). The combined organic fractions were washed with water, dried over $MgSO_4$, filtered, and the solvent removed in vacuo. Purification of the crude material by flash column chromatography (*isocratic in* 5% MeOH in CH_2Cl_2) delivered seproxetine **S-7** (218 mg, 76% yield) as pale yellow oil. The spectroscopic properties of this compound were consistent with the data available in the literature.[33]

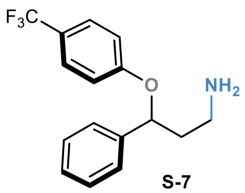

S-7

^{1}H NMR (500 MHz, $CDCl_3$) (400 MHz, $CDCl_3$) δ 7.45 – 7.39 (m, 2H), 7.36 – 7.30 (m, 4H), 7.29 – 7.25 (m, 1H) , 6.94 – 6.86 (m, 2H), 5.32 (dd, J = 8.4, 4.6 Hz, 1H), 2.90 (t, J = 6.8 Hz, 2H), 2.18 (ddt, J = 14.8, 8.4, 6.4 Hz, 1H), 2.04 – 1.95 (m, 1H), 1.92 (d, J = 5.7 Hz, 2H). **^{13}C NMR** (126 MHz, $CDCl_3$) δ (101 MHz, $CDCl_3$) δ 160.5, 141.0, 128.8, 127.9, 126.8 (q, $^3J_{C–F}$ = 3.8 Hz), 125.8, 124.5 (q, $^1J_{C–F}$ = 270.7 Hz), 123.0 (q, $^2J_{C–F}$ = 32.7 Hz), 115.8, 78.3, 42.0, 38.5. **^{19}F NMR** (376 MHz, $CDCl_3$) δ –61.5.

S.7.2.2 – *Synthesis of **Fluoxetine** (**S-8**)*

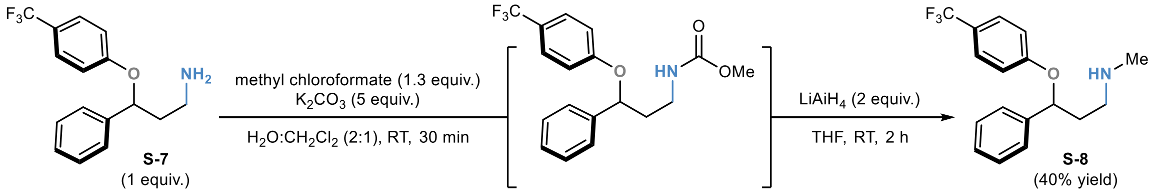

To a mixture of seproxetine **S-7** (219 mg, 0.74 mmol) and methyl chloroformate (74.5 μL, 0.96 mmol) in CH_2Cl_2 (4 mL) was added a solution of K_2CO_3 (511 mg, 3.70 mmol) in water (8 mL). The resulting mixture was stirred for 30 minutes at room temperature, then diluted

with water (5 mL) and, ultimately, extracted with CH_2Cl_2 (3 x 10 mL). The combined organic extracts were dried over Na_2SO_4, filtered, and the solvent removed in vacuo to afford an amber oil. The crude material was re-dissolved in THF (4 mL), and this solution was added to a suspension of $LiAlH_4$ (56.2 mg, 1.48 mmol) in THF (6 mL), at 0 °C. Completed the addition, the reaction was allowed to warm up to room temperature, and stirred for 2 hours. The reaction was quenched by cautious addition of water (10 mL) and extracted with EtOAc (3 x 15 mL). The combined organic layers were washed with brine, dried over $MgSO_4$, filtered and the solvent removed in vacuo. Purification of the crude material by flash column chromatography (*isocratic in* 5% MeOH in CH_2Cl_2) delivered fluoxetine **S-8** (92.0 mg, 40% yield) as a colorless oil. The spectroscopic properties of this compound were consistent with the data available in the literature.[34]

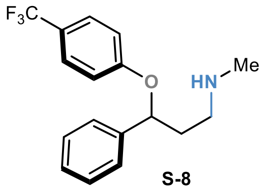

S-8

^{1}H NMR (400 MHz, $CDCl_3$) δ 7.45 (d, J = 8.7 Hz, 2H), 7.39 – 7.33 (m, 4H), 7.32 – 7.26 (m, 1H), 6.93 (d, J = 8.7 Hz, 2H), 5.36 – 5.27 (m, 1H), 2.49 – 2.42 (m, 2H), 2.26 (s, 3H), 2.25 – 2.15 (m, 1H), 2.05 – 1.95 (m, 1H). **^{13}C NMR** (126 MHz, $CDCl_3$) δ 160.7, 141.2, 128.8, 127.8, 126.7 (q, $^3J_{C-F}$ = 3.7 Hz), 125.9, C_qF_3 not detected, 122.7 (q, $^2J_{C-F}$ = 33.5 Hz), 115.8, 78.5, 55.8, 45.5, 36.8. **^{19}F NMR** (376 MHz, $CDCl_3$) δ –61.6.

S.7.3 – Synthetic Manipulations of 67

S.7.3.1 – *Synthesis of* ***3-(3-acetyl-2,2-dimethylcyclobutyl)propanoic acid*** *(**73**)*

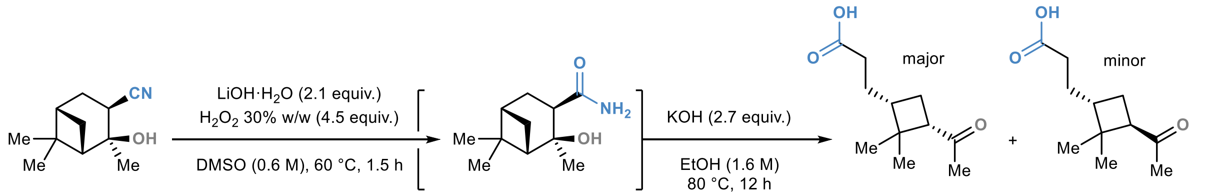

67 (1 equiv.) **72** not isolated **73** (90% yield), 2.8:1 d.r.

A three-necked flask, equipped with reflux condenser, was charged with 1,2-hydroxy nitrile **67** (150 mg, 837 μmol), LiOH monohydrate (73.7 mg, 1.76 mmol) and DMSO (1.5 mL), and the resulting solution was heated to 60 °C. Once reached this temperature, an aqueous solution of H_2O_2 (*30% w/w*, 256 μL, 2.51 mmol) was slowly added over 1 hour to the reaction vessel. Completed the addition, the mixture was stirred at the same temperature for further 30 minutes, and then allowed to cool down to room temperature. The crude mixture was quenched by addition of a 1M HCl aq. solution (1 mL) and extracted with Et_2O (3 x 5 mL). The combined organic layers were dried over Na_2SO_4, filtered and the solvent removed under reduced pressure, to give crude **72** as a yellow glue. This was dissolved in EtOH (1 mL) and treated with a 8M aq. solution of KOH (300 μL). The resulting mixture was heated to 80 °C, stirred for 12 hours, and then allowed to cool down to room temperature. The crude mixture was partitioned into water (5 mL) and Et_2O (5 mL), and extracted with Et_2O (3 x 5 mL). The aqueous layers were acidified with 1M HCl, till pH ~1, and then counter-extracted with Et_2O

(3 x 10 mL). The combined organic layers were dried over Na_2SO_4, filtered and the solvent removed under reduced pressure. Purification of the crude material by flash column chromatography (*gradient from* 50% EtOAc in hexane *to* 70% EtOAc in hexane) delivered carboxylic acid **73** (149.8 mg, 90% yield, *2.8:1 mixture of diastereoisomers*) as an off-white solid. The title compound was previously unknown. The stereochemistry of both diastereoisomers of **73** has been inferred by ^{1}H NOESY NMR analysis.

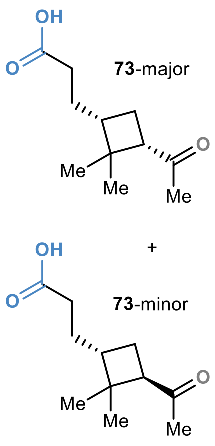

^{1}H NMR (500 MHz, $CDCl_3$) δ 2.93 (dd, *J* = 8.4, 6.3 Hz, 1H x **73-minor**), 2.83 – 2.77 (m, 1H x **73-major**), 2.41 (ddd, *J* = 11.7, 8.8, 6.3 Hz, 1H x **73-minor**), 2.35 – 2.19 (m, 2H x **73-major** & 2H x **73-minor**), 2.06 (s, 3H x **73-minor**), 2.04 (s, 3H x **73-major**), 1.96 – 1.77 (m, 3H x **73-major** & 2H x **73-minor**), 1.71 – 1.60 (m, 1H x **73-major** & 1H x **73-minor**), 1.54 (dtd, *J* = 13.6, 8.5, 6.4 Hz, 1H x **73-major**), 1.49 – 1.41 (m, 1H x **73-minor**), 1.30 (s, 3H x **73-major**), 1.22 (s, 3H x **73-minor**), 0.99 (s, 3H x **73-minor**), 0.87 (s, 3H x **73-major**). **^{13}C NMR** (126 MHz, $CDCl_3$) δ 209.3 (**73-minor**), 208.1 (**73-major**), 179.4 (**73-minor**), 179.4 (**73-major**), 54.1 (**73-major**), 53.7 (**73-minor**), 43.3 (**73-major**), 41.7 (**73-minor**), 41.4 (**73-major**), 41.0 (**73-minor**), 32.1 (**73-minor**), 32.0 (**73-major**), 31.0 (**73-minor**), 30.6 (**73-major**), 30.3 (**73-major**), 29.8 (**73-minor**), 26.0 (**73-minor**), 25.1 (**73-major**), 24.7 (**73-minor**), 22.9 (**73-major**), 22.5 (**73-minor**), 17.2 (**73-major**). **HRMS (ESI$^+$)** Mass calculated for $C_{11}H_{18}O_3Na$ $[M+Na]^+$: 221.1135, found: 221.1141.

S.8 – X-ray Crystallographic Data

All data collections, crystal structure determinations and refinements were performed by the X-ray crystallography service of the Department of Chemistry at The University of Manchester (EPSRC; EP/T011289/1). For these endeavors, we thank Dr George Whitehead and Dr Avantika Hasija.

Data collection: X-ray data was collected at a temperature of 100 K on a Rigaku FR-X DW rotating anode diffractometer using CuKα radiation, (λ = 1.54184 Å) with an AFC-11 RINC goniometer and a Rigaku Hypix 6000 HE photon counting detector. The diffractometer was equipped with an Oxford Cryosystems Cryostream 800 plus nitrogen flow gas system.

Crystal Structure Determinations and Refinements: X-ray data were processed and reduced using CrysAlisPro v41.[35] Absorption correction was performed using empirical methods (SCALE3 ABSPACK) based upon symmetry-equivalent reflections combined with measurements at different azimuthal angles. The crystal structures were solved using ShelXT and refined against all F^2 values using the ShelXL implemented through Olex 2.[36,37] All the non-hydrogen atoms were refined anisotropically. Hydrogen atoms were placed in a calculated position refined using idealized geometries (riding model) and assigned fixed isotropic displacement parameters.

Data Availability: Crystallographic data for compounds **1b**, **62** and **69** have been deposited in the Cambridge Crystallographic Data Centre, with deposition numbers CCDC 2384299 (**1b**), 2384621 (**62**), 2384300 (**69**) and are available free of charge at www.ccdc.cam.ac.uk/data_request/cif; or from the Cambridge Crystallographic Data Centre (12 Union Road, Cambridge CB21EZ, UK – fax: (+44)1223 336033, email: deposit@ccdc.cam.ac.uk).

S.8.1 – Single Crystal X-ray Diffraction Data for Compound 1b:

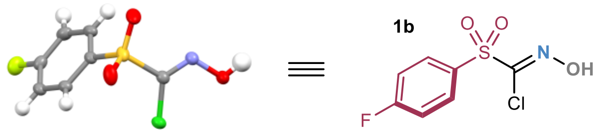

CCDC Deposition Number	2384299
Empirical Formula	$C_7H_5ClFNO_3S$
Temperature (K)	100
Crystal system	monoclinic
Space group	P 1 21/c 1
a (Å)	10.5995(3)
b (Å)	8.0433(2)
c (Å)	10.9146(3)
α (°)	90
β (°)	104.860(3)
γ (°)	90
Volume (Å^3)	899.40(4)
Z	4
F(000)	480.0
Radiation	Cu K\a
2Θ range for data collection (°)	4.3050 to 75.3930
Index ranges	–13 ≤ h ≤ 13, –9 ≤ k ≤ 9, –13 ≤ l ≤ 13
Goodness-of-fit on F2	1.091
Final R indexes [I>=2σ (I)]	R_1 = 0.0328 | wR_2 = 0.0918
Final R indexes [all data]	R_1 = 0.0338 | wR_2 = 0.0918

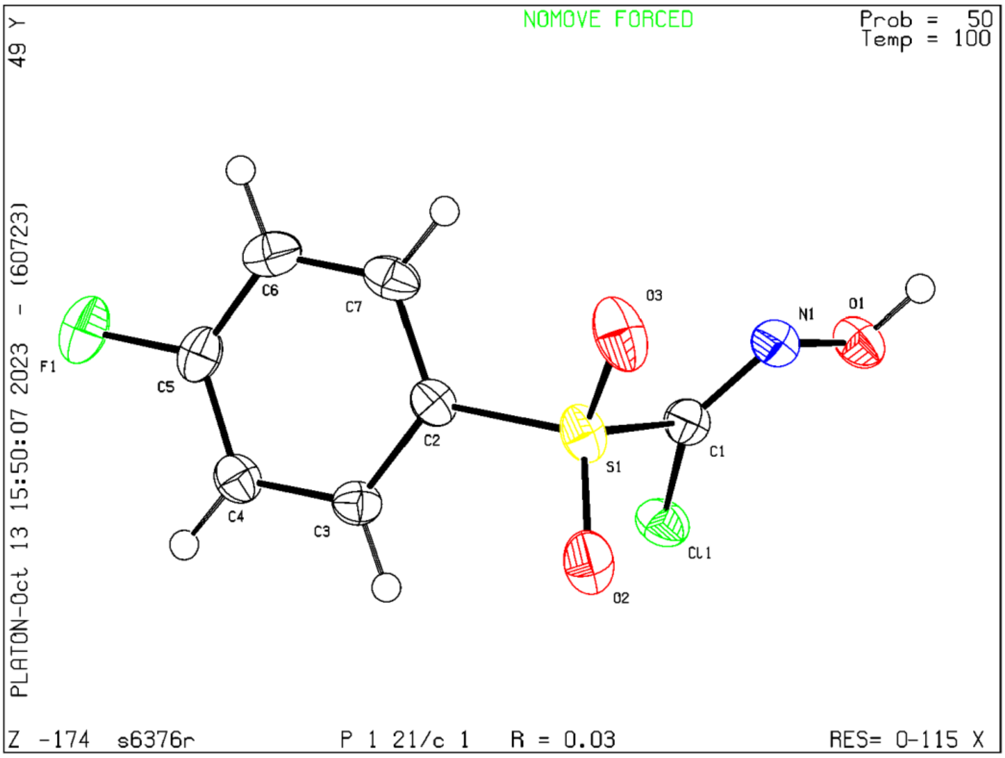

S.8.2 – Single Crystal X-ray Diffraction Data for Compound 62:

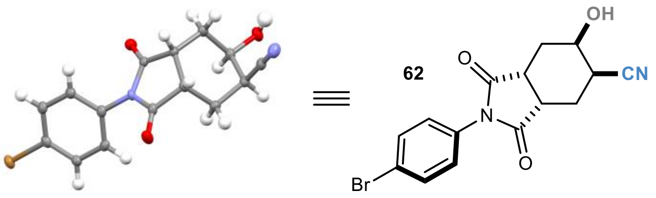

CCDC Deposition Number	2384621
Empirical Formula	$C_{15}H_{13}BrN_2O_3$
Temperature (K)	100
Crystal system	orthorhombic
Space group	P n a 21
a (Å)	6.79151(14)
b (Å)	16.3107(3)
c (Å)	12.2567(3)
α (°)	90
β (°)	90
γ (°)	90
Volume (Å^3)	1357.72(5)

Z	4
F(000)	704.0
Radiation	Cu K\a
2θ range for data collection (°)	2.7150 to 75.9720
Index ranges	–8 ≤ h ≤ 8, –19 ≤ k ≤ 19, –14 ≤ l ≤ 14
Goodness-of-fit on F2	1.101
Final R indexes [I>=2σ (I)]	R_1 = 0.0246 | wR_2 = 0.0699
Final R indexes [all data]	R_1 = 0.0248 | wR_2 = 0.0699

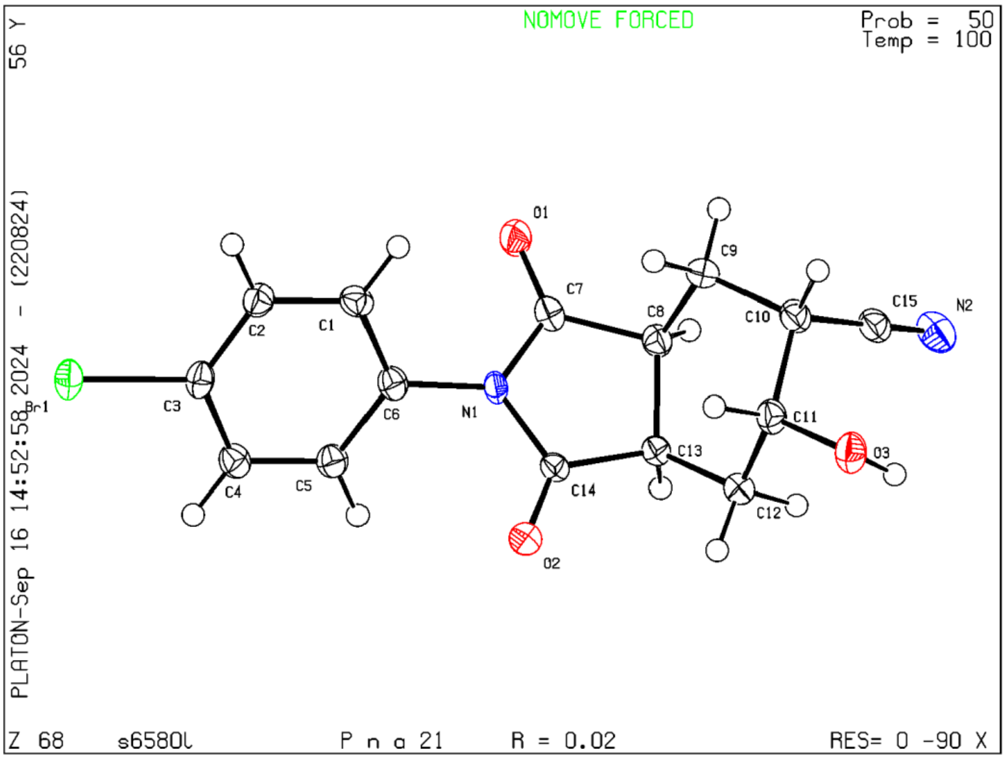

S.8.3 – Single Crystal X-ray Diffraction Data for Compound 69:

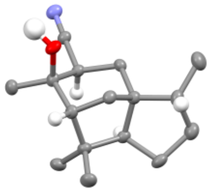

69

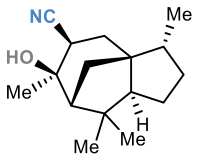

CCDC Deposition Number	2384300
Empirical Formula	$C_{16}H_{25}NO$
Temperature (K)	100
Crystal system	orthorhombic

Space group	P 21 21 21
a (Å)	7.0056(2)
b (Å)	8.1668(2)
c (Å)	25.4708(6)
α (°)	90
β (°)	90
γ (°)	90
Volume (Å^3)	1457.26(6)
Z	4
F(000)	544.0
Radiation	Cu K\a
2Θ range for data collection (°)	3.4680 to 80.0390
Index ranges	$-8 \le h \le 8, -10 \le k \le 10, -32 \le l \le 32$
Goodness-of-fit on F2	1.091
Final R indexes [I>=2σ (I)]	R_1 = 0.0530 | wR_2 = 0.1481
Final R indexes [all data]	R_1 = 0.0620 | wR_2 = 0.1481
Absolute structure parameter	-0.2(3)

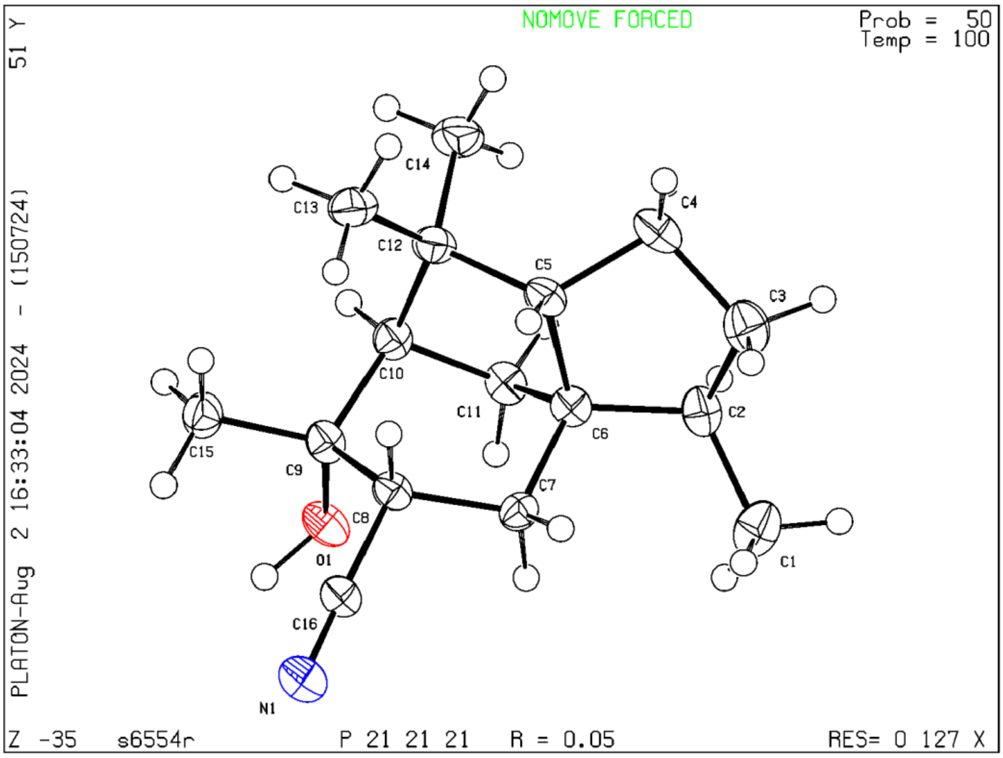

S.9 – References

1. Liu, S.; Jin, Z.; Teo, Y. C.; Xia, Y. *J. Am. Chem. Soc.* **2014**, *136*, 17434–17437.
2. Zimdars, P.; Böhlig, K.; Metz, P. *Eur. J. Org. Chem.* **2019**, 6163–6167.
3. Coe, J. W.; Bianco, K. E.; Boscoe, B. P.; Brooks, P. R.; Cox, E. D.; Vetelino. M. G. *J. Org. Chem.* **2003**, *68*, 9964–9970.
4. Tan, A.; Koc, B.; Sahin, E.; Kishali, N. H.; Kara, Y. *Synthesis* **2011**, *7*, 1079–1084.
5. Hanson, P.; Wren, S. A. C. *J. Chem. Soc. Perkin Trans. 1* **1990**, 2089–2097.
6. Dar'in, D.; Kantin, G.; Bakulina, O.; Krasavin, M. *Synthesis* **2020**, *52*, 2259–2266.
7. Yan, Y.; Ma, G.; Wei, W.; Zhao, J. *Aust. J. Chem.* **2016**, *69*, 239–242.
8. Davies, H. M. L.; Houser, J. H.; Thornley, C. *J. Org. Chem.* **1995**, *60*, 7529–7534.
9. Korneev, S.; Richter, C. *Synthesis* **1995**, *1995*, 1248–1250.
10. Föhlisch, B.; Herter, R.; Wolf, E.; Stezowski, J. J.; Eckle, E. *Chem. Ber.* **1982**, *115*, 355–380.
11. Mowner, M. P.; Blackmond, D. G. *J. Am. Chem. Soc.* **2015**, *137*, 2386−2391.
12. Chen, R.; Zhao, Y.; Fang, S.; Long, W.; Sun, H.; Wan, X. *Org. Lett.* **2017**, *19*, 5896−5899.
13. Guo, D.-C.; Zhang, C.; Li, F.; Zhang, F.; Yu, F.; He, Y.-P. *Synthesis* **2017**, *49*, 1356–1370.
14. Patel, S.; Paul, B.; Paul, H.; Shankhdhar, R.; Chatterjee, I. *Chem. Commun.* **2022** , *58*, 4857–4860.
15. Hioki, Y.; Constantini, M.; Griffin, J.; Harper, K. C.; Prado Merini, M.; Nissi, B.; Kawamata, Y.; Baran, P. S. *Science* **2023**, *380*, 81–87.
16. Barton, L. M.; Chen, L.; Blackmond, D. G.; Baran, P. S. *PNAS* **2021**, *118*, e2109408118.
17. Sassaman, M. B.; Prakash, G. K. S.; Olah, G. A. *J. Org. Chem.* **1990**, *55*, 2016–2018.
18. Lai, J.; Chang, L.; Yuan, G. *Org. Lett.* **2016**, *18*, 3194−3197.
19. Yamasaki, S.; Kanai, M.; Shibasaki, M. *J. Am. Chem. Soc.* **2001**, *123*, 1256–1257
20. Majerić Elenkov, M.; Hauer, B.; Janssen, D. B. *Adv. Synth. Catal.* **2006**, *348*, 579–585.
21. Coady, T. M.; Coffey, L. V.; O'Reilly, C.; Lennon, C. M. *Eur. J. Org. Chem.* **2015**, 1108–1116.
22. Naeimi, H.; Moradian, M. *Can. J. Chem.* **2006**, *84*, 1575–1579.
23. Boate, D. R.; Hunter, D. H. Org. Magn. Reason. **1984**, *22*, 167–170.
24. Huisgen, R.; Christl, M. *Angew. Chem. Int. Ed. Engl.* **1967**, *6*, 456–457; *Angew. Chem.* **1967**, *79*, 471–472.

25. Lukevics, E.; Dirnens, V.; Kemme, A.; Popelis, J. *J. Organomet. Chem.* **1996**, *521*, 235–244.
26. Talwar, D.; Wu, X.; Saidi, O.; Poyatos Salguero, N.; Xiao, J. *Chem. Eur. J.* **2014**, *20*, 12835–12842.
27. Chakraborty, S.; Patel, Y. J.; Krause, J. A.; Guan, H. *Angew. Chem. Int. Ed.* **2013**, *52*, 7523–7526.
28. Kiyokawa, K.; Ishizuka, M.; Minakata, S. *Angew. Chem. Int. Ed.* **2023**, *62*, e202218743.
29. Fan, L.; Ozerov, O. V. *Chem. Commun.* **2005**, 4450–4452.
30. Mi, M.; Liu, C.; Chen, H.; Qi, Y.; Liu, Y. *Tetrahedron Lett.* **2023**, *132*, 154819.
31. You, Z.; Lee, H. J. *Tetrahedron Lett.* **1996**, *37*, 1165–1168.
32. Li, B., Tochtrop, G. P. *J. Org. Chem.* **2022**, *87*, 3851−3855.
33. Cashman, J. R.; Voelker, T.; Johnson, R.; Janowsky, A. *Bioorg. Med. Chem.* **2009**, *17*, 337–343.
34. Zhang, M.; Liu, Z.; Zhao, W. *Angew. Chem. Int. Ed.* **2023**, *62*, e202215455.
35. Rigaku Oxford Diffraction, CrysAlisPro Software system, version 41, Rigaku Corporation, Oxford, UK (2017).
36. (a) Sheldrick, G. M. *Acta Cryst. A.* **2015**, *71*, 3–8; (b) Sheldrick, G. M. *Acta Cryst. C.* **2015**, *71*, 3–8.
37. Dolomanov, O. V.; Bourhis, L. J.; Gildea, R. J.; Howard, J. A. K.; Puschmann, H. *J. Appl. Cryst.* **2009**, *42*, 339–341.

S.10 – NMR Spectra (^{1}H, ^{13}C, ^{19}F and ^{31}P Traces)

^{1}H NMR (400 MHz, $CDCl_3$) for compound **6**:

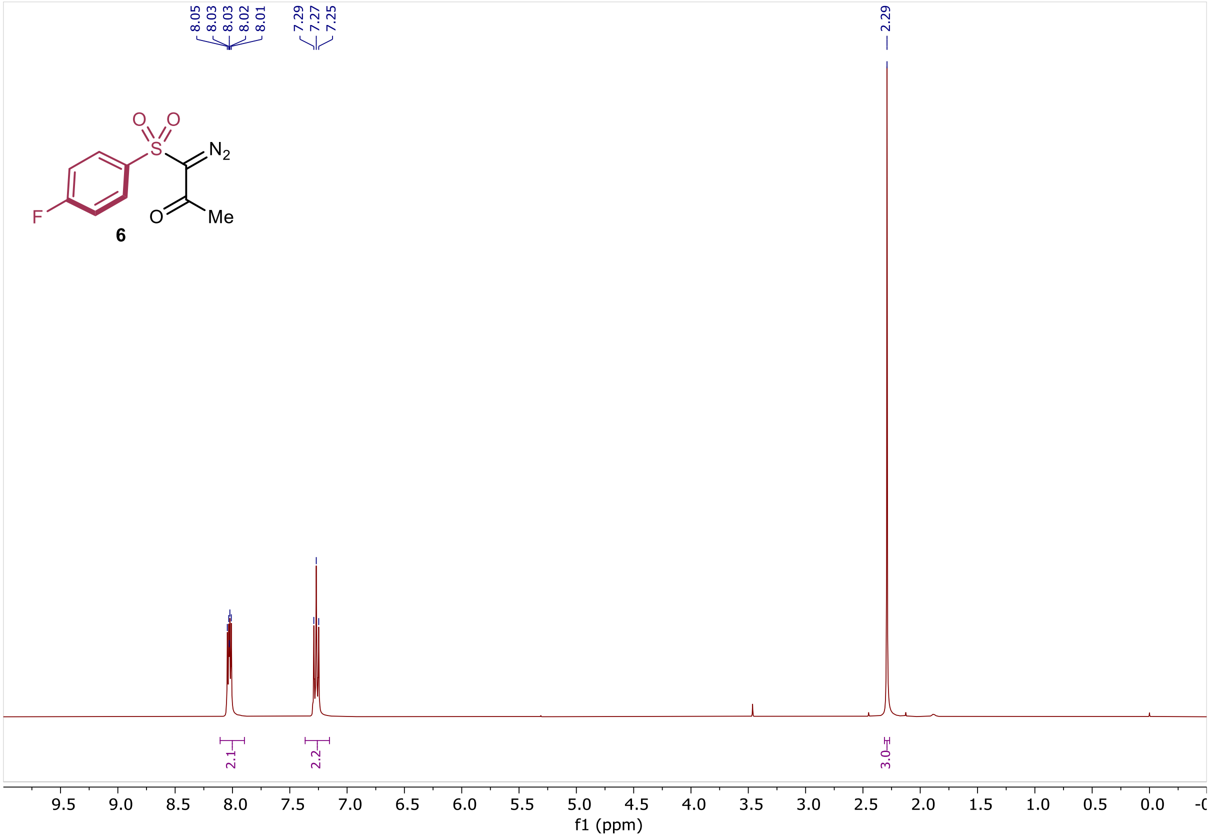

^{13}C NMR (101 MHz, $CDCl_3$) for compound **6**:

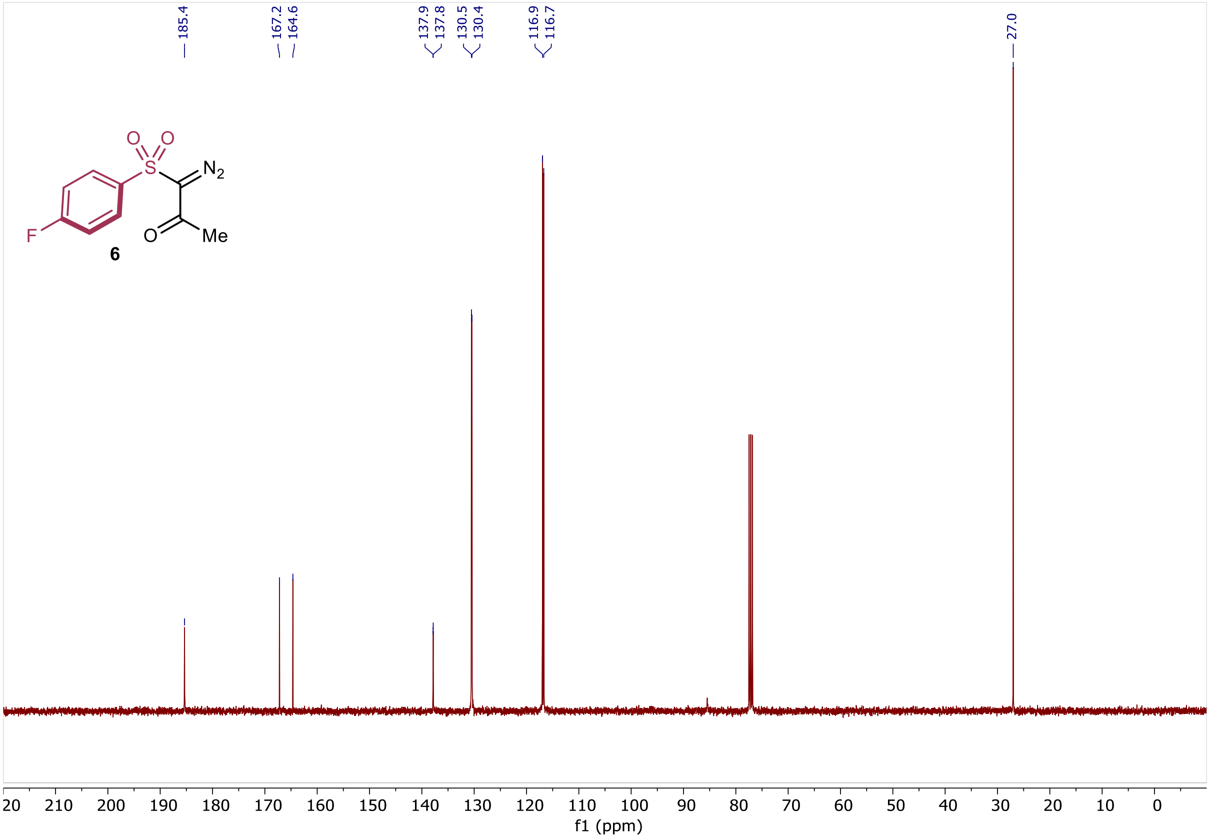

^{19}F NMR (376 MHz, $CDCl_3$) for compound **6**:

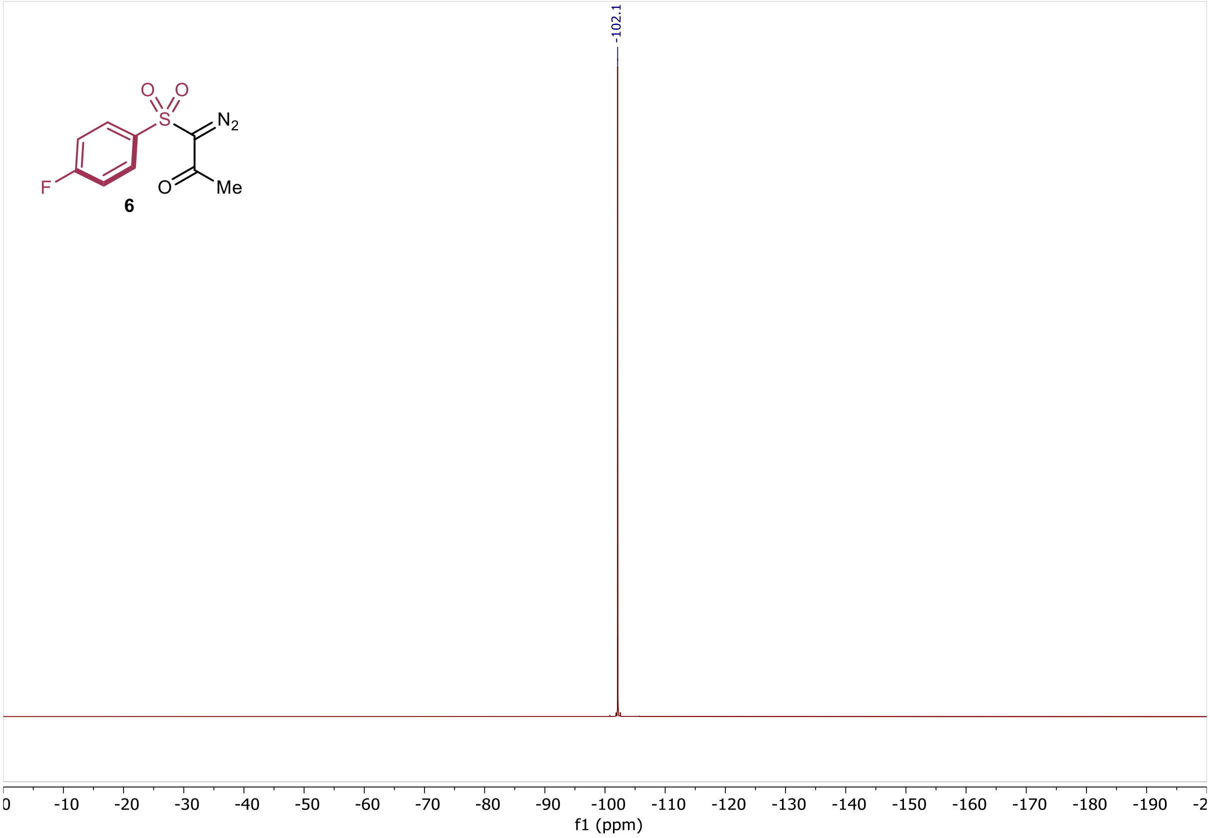

^{1}H NMR (400 MHz, $CDCl_3$) for compound **1a**:

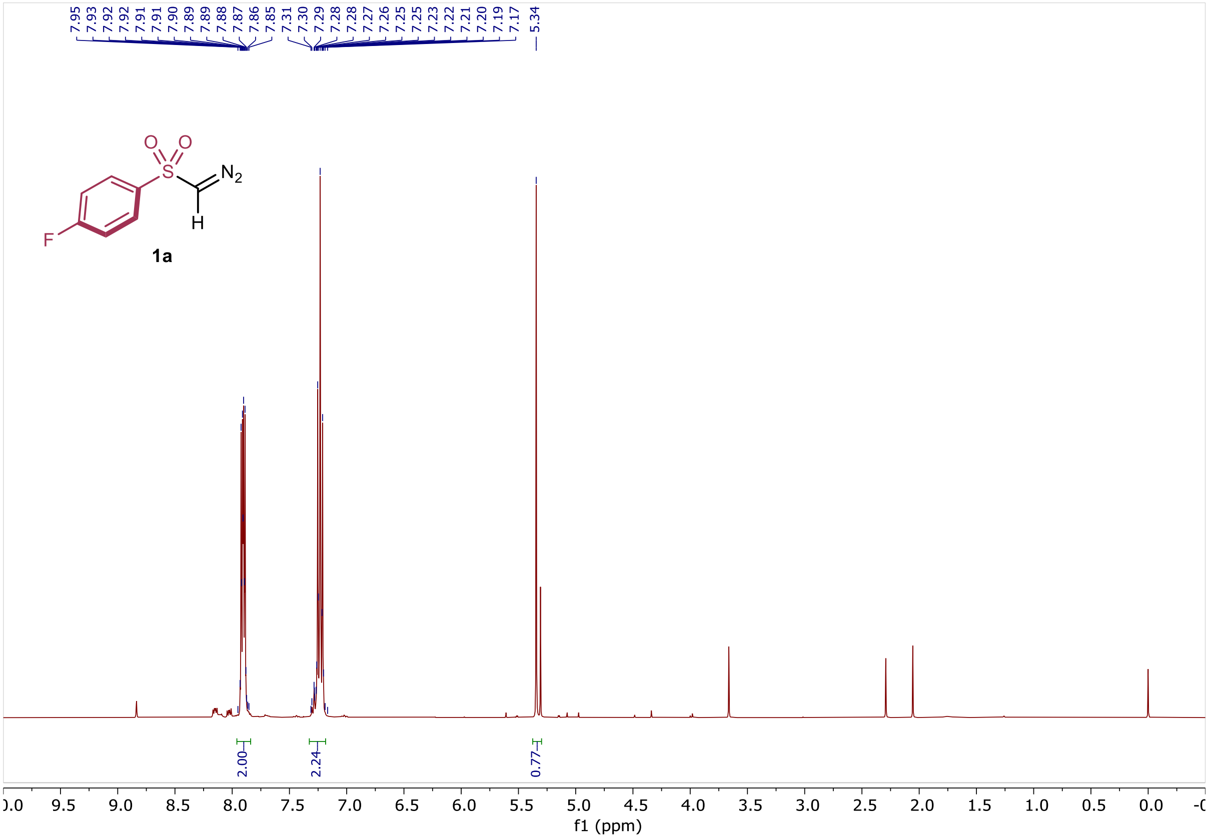

^{13}C NMR (101 MHz, $CDCl_3$) for compound **1a**:

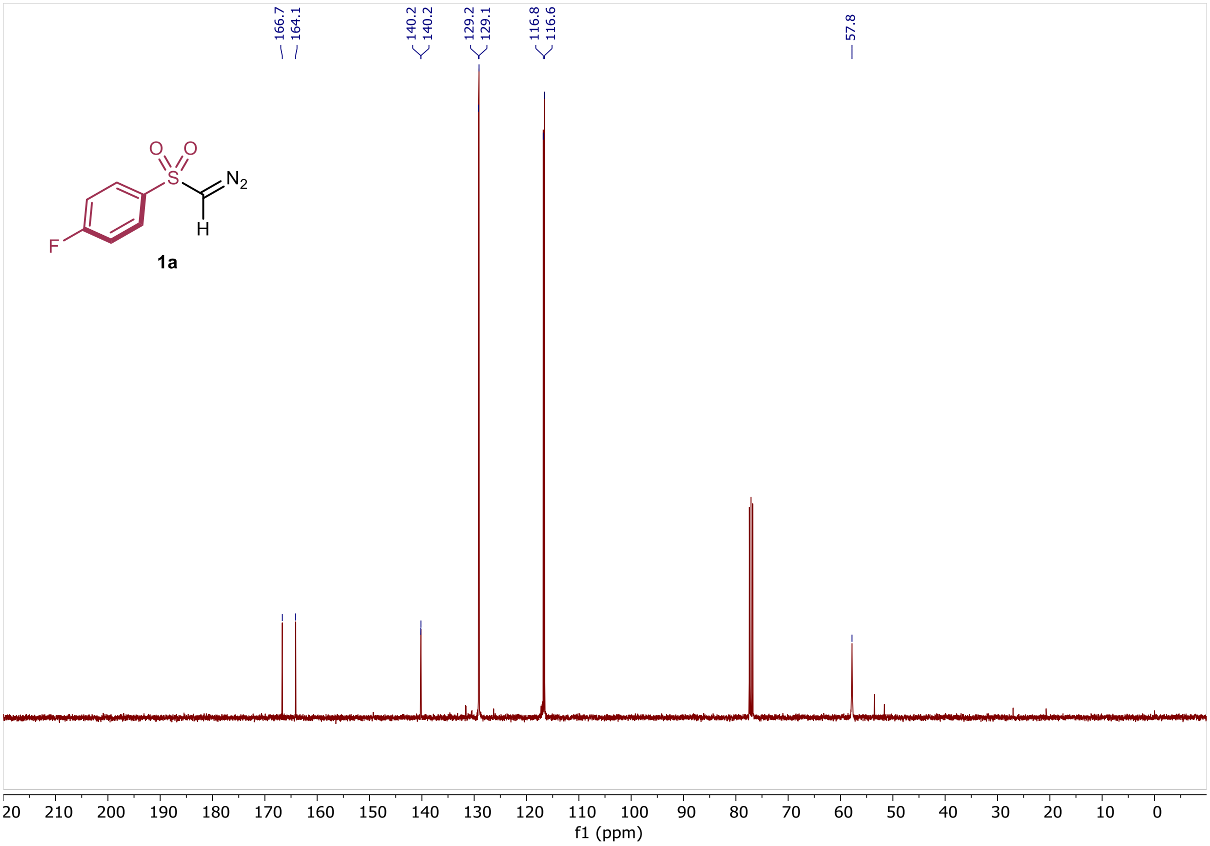

^{19}F NMR (376 MHz, $CDCl_3$) for compound **1a**:

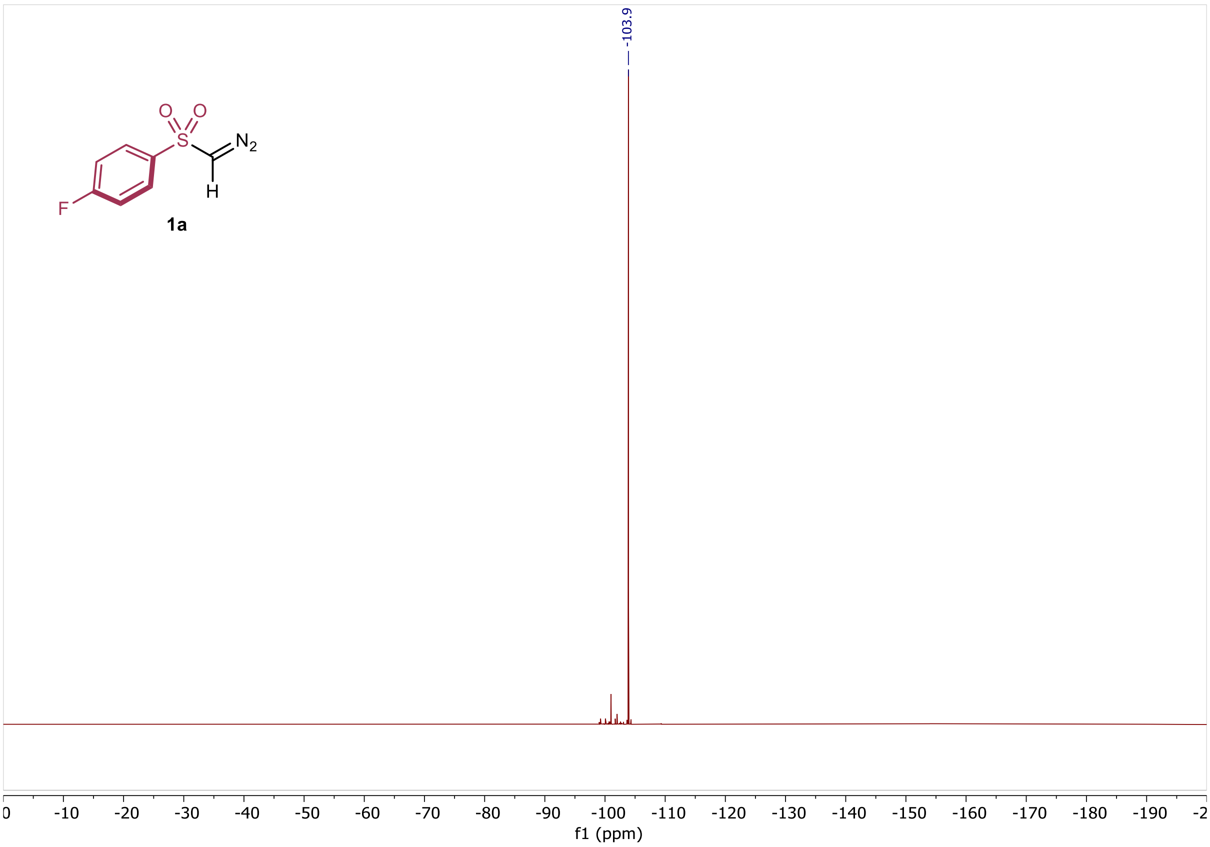

[1]**H NMR** (500 MHz, DMSO-d_6) for compound **1b**:

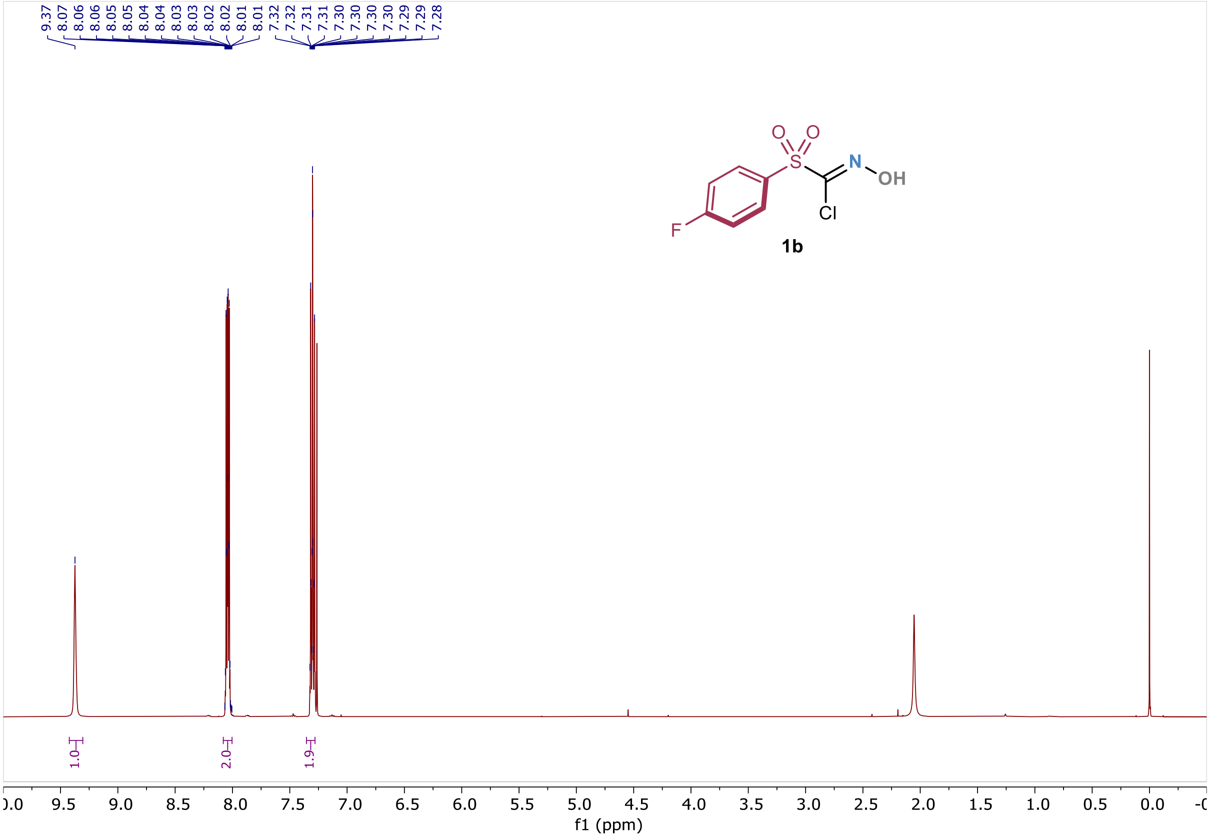

[13]**C NMR** (101 MHz, DMSO-d_6) for compound **1b**:

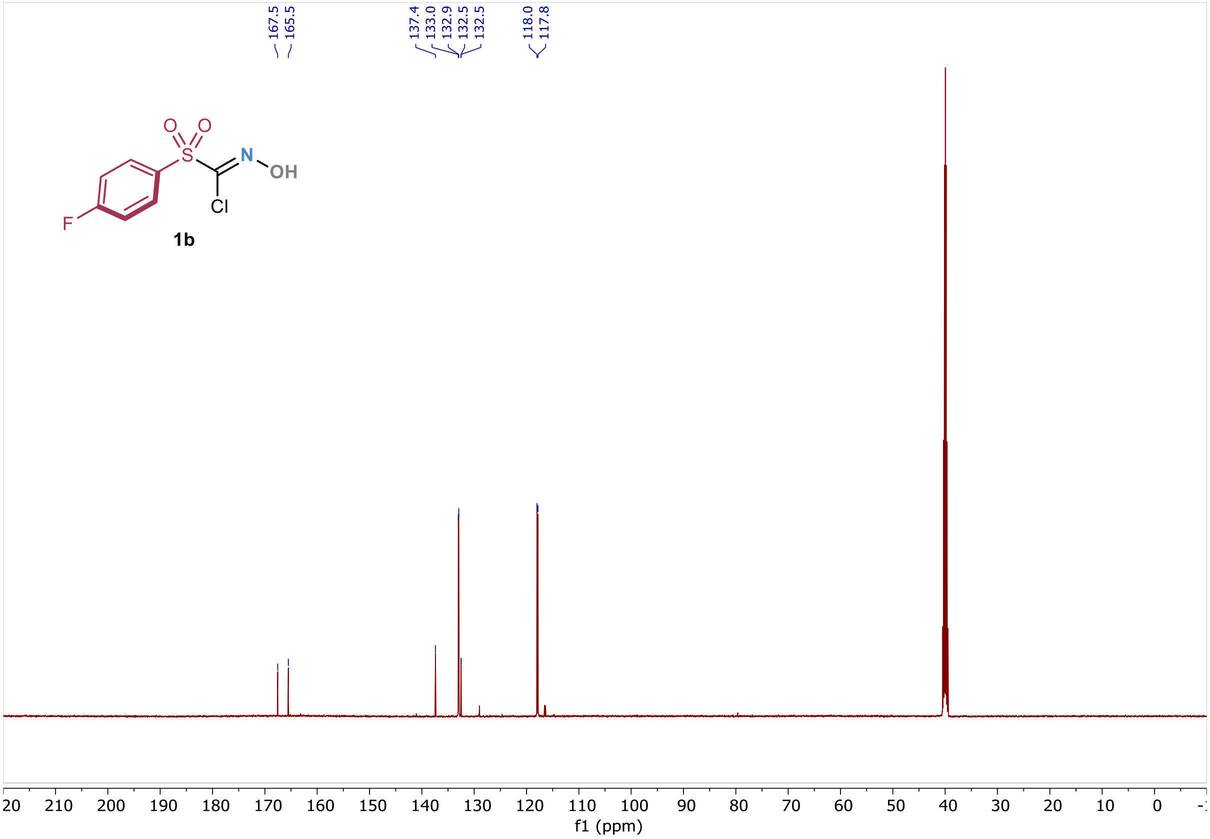

^{19}**F NMR** (376 MHz, DMSO-d_6) for compound **1b**:

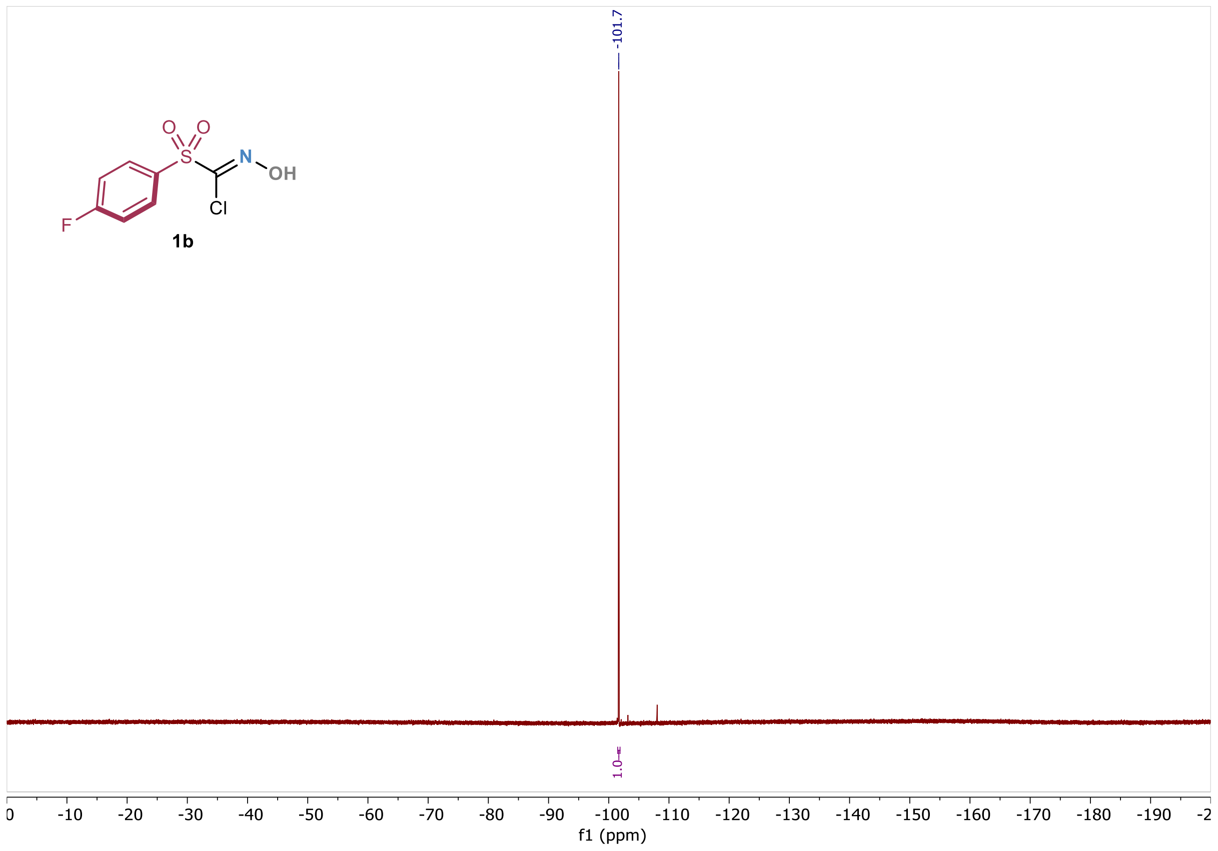

^{1}**H NMR** (500 MHz, $CDCl_3$) for compound **S-1**:

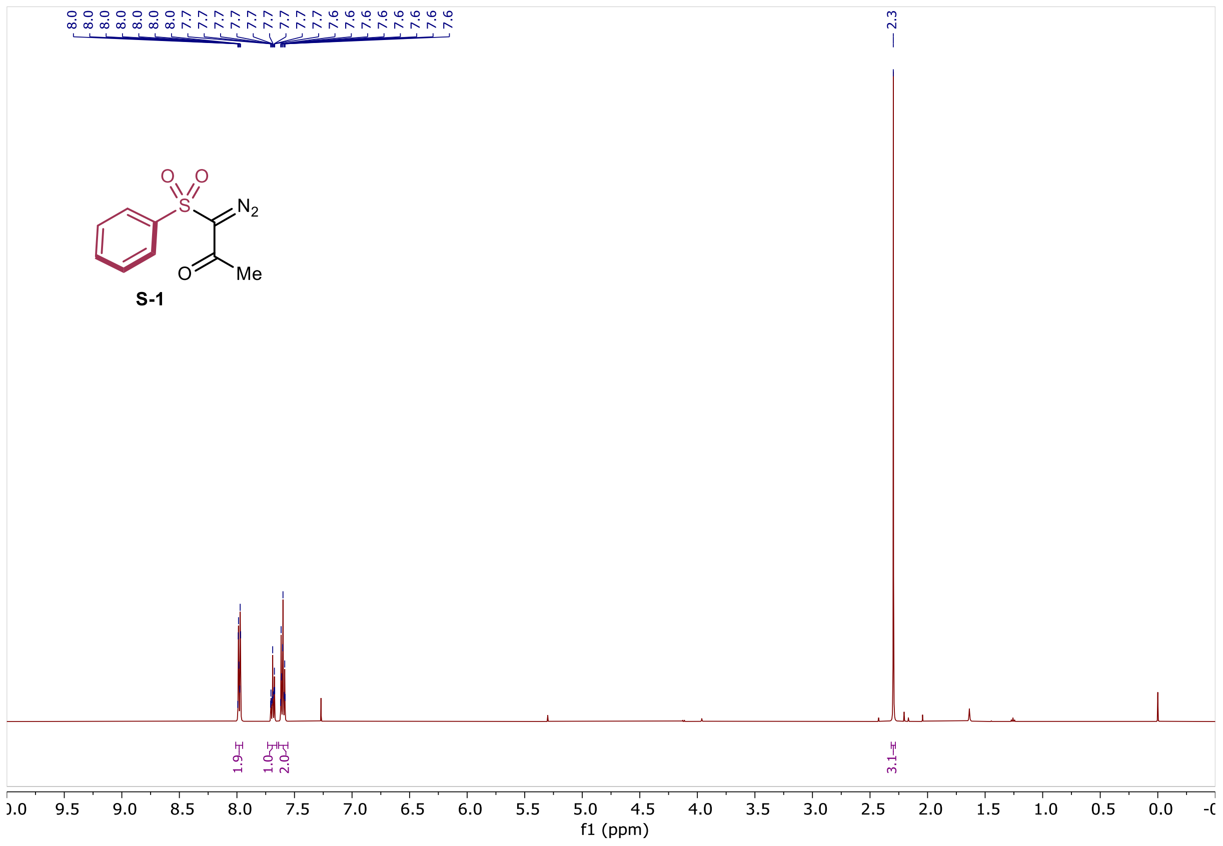

^{13}C NMR (126 MHz, $CDCl_3$) for compound **S-1**:

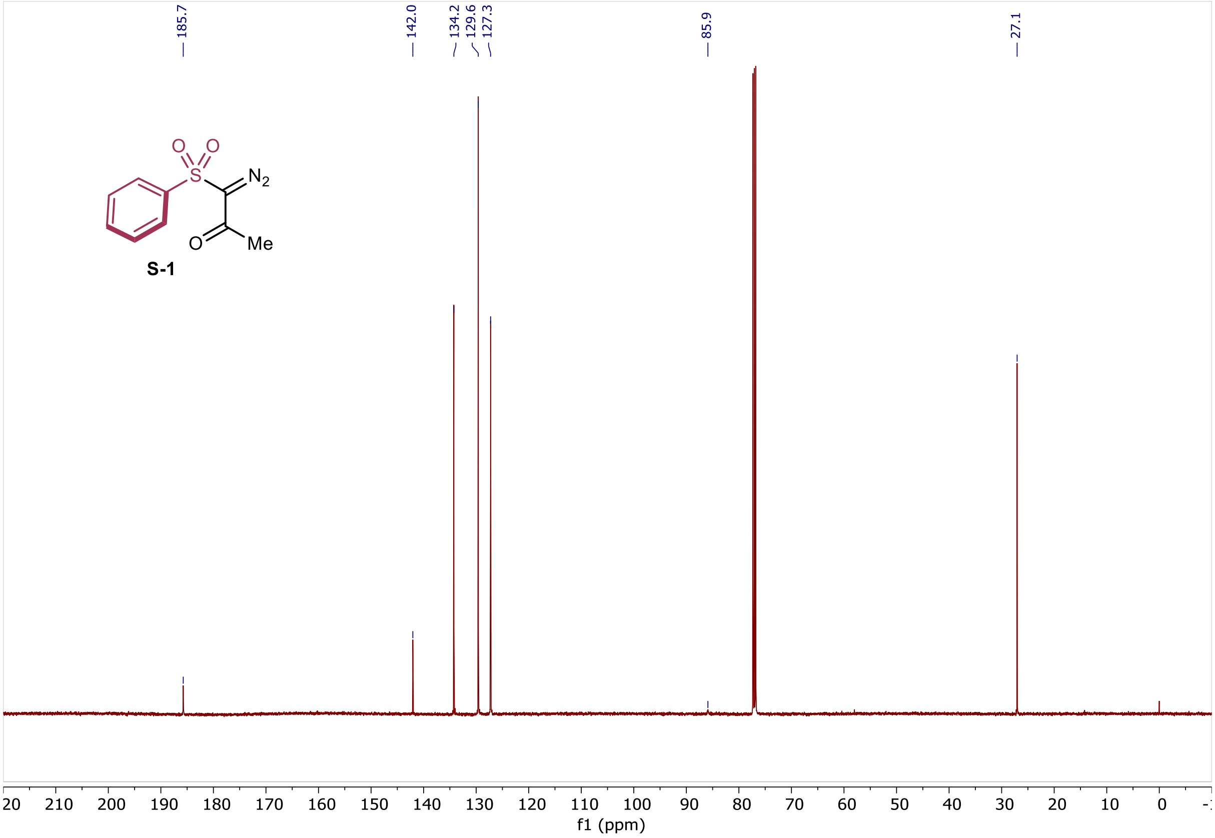

^{1}H NMR (400 MHz, $CDCl_3$) for compound **1c**:

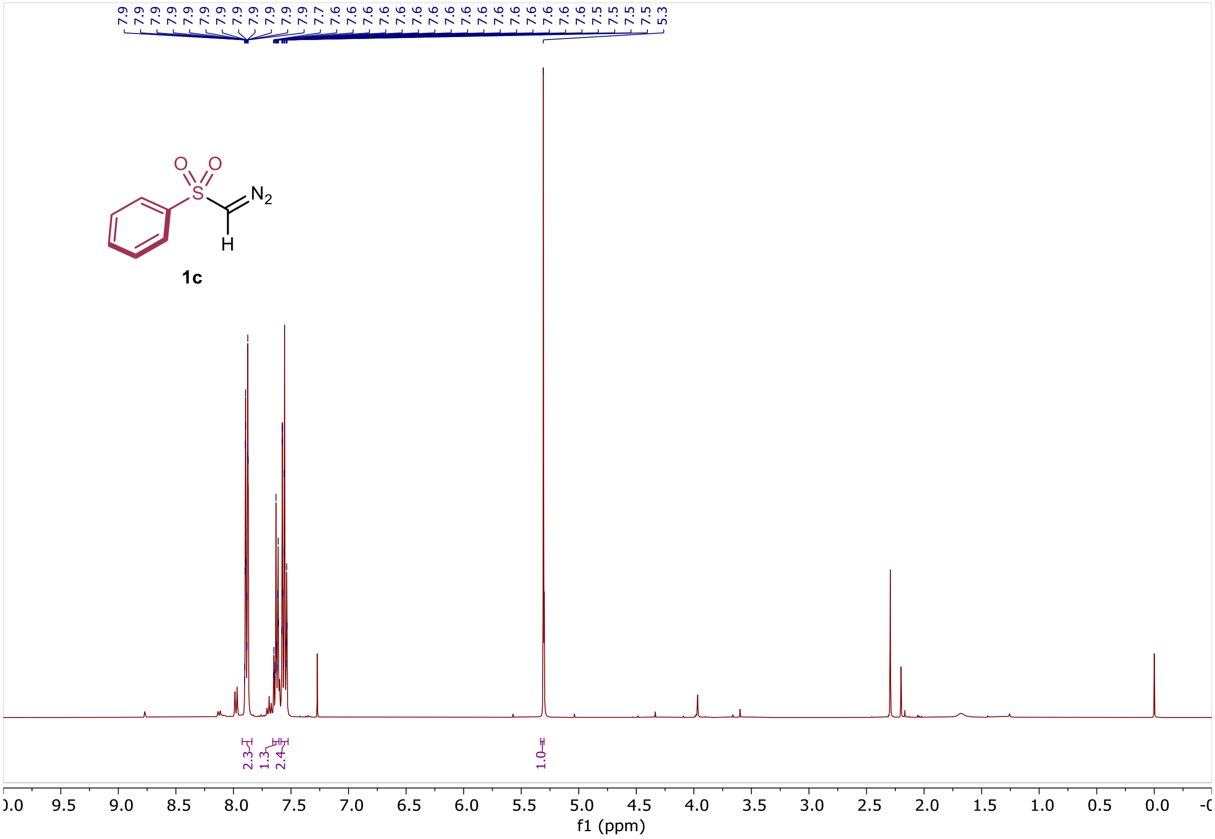

^{13}C NMR (101 MHz, $CDCl_3$) for compound **1c**:

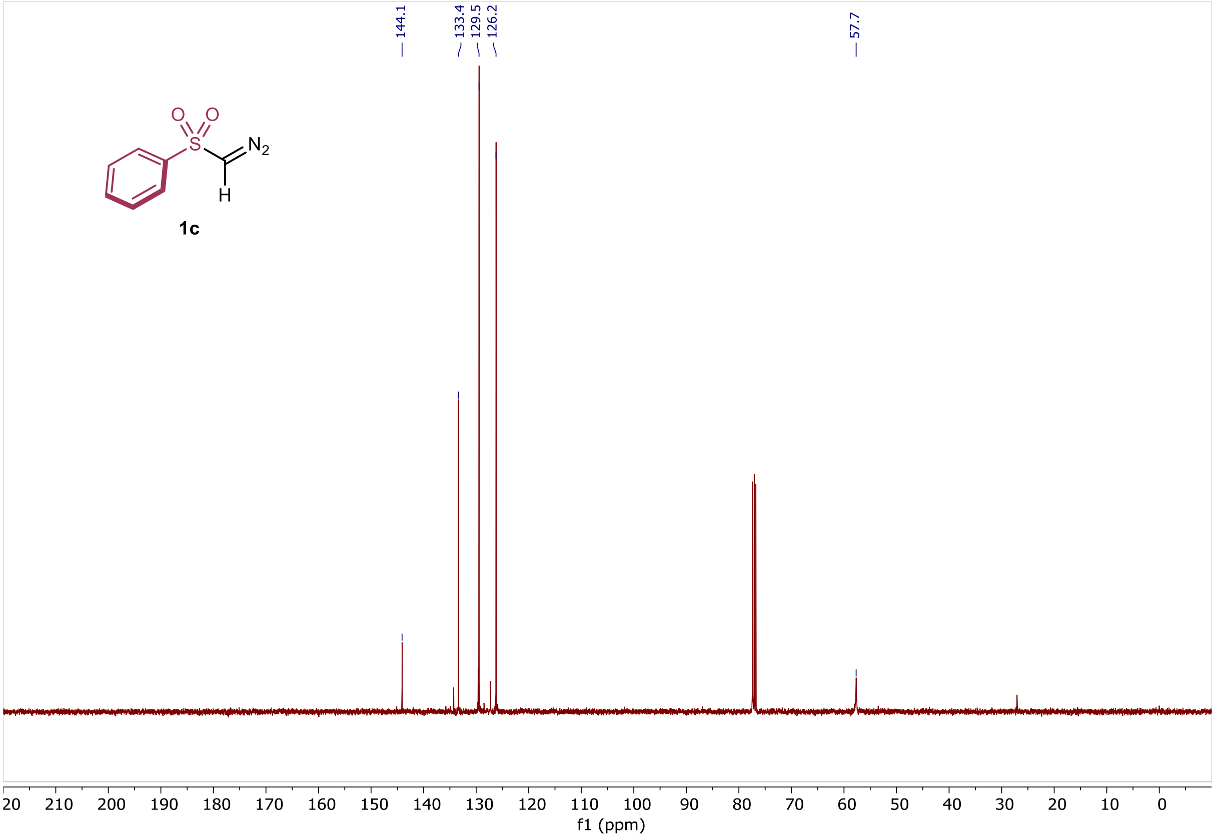

^{1}H NMR (400 MHz, $CDCl_3$) for compound **8**:

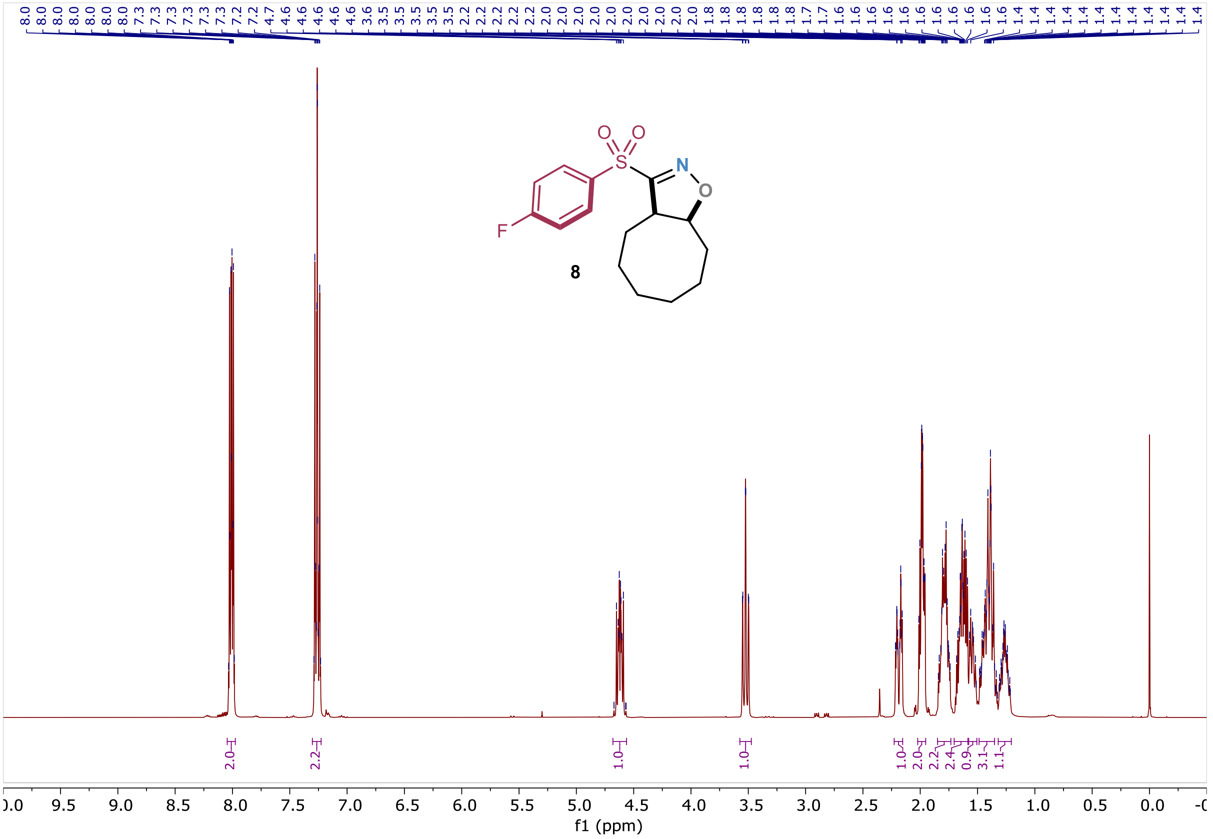

¹³C NMR (126 MHz, $CDCl_3$) for compound **8**:

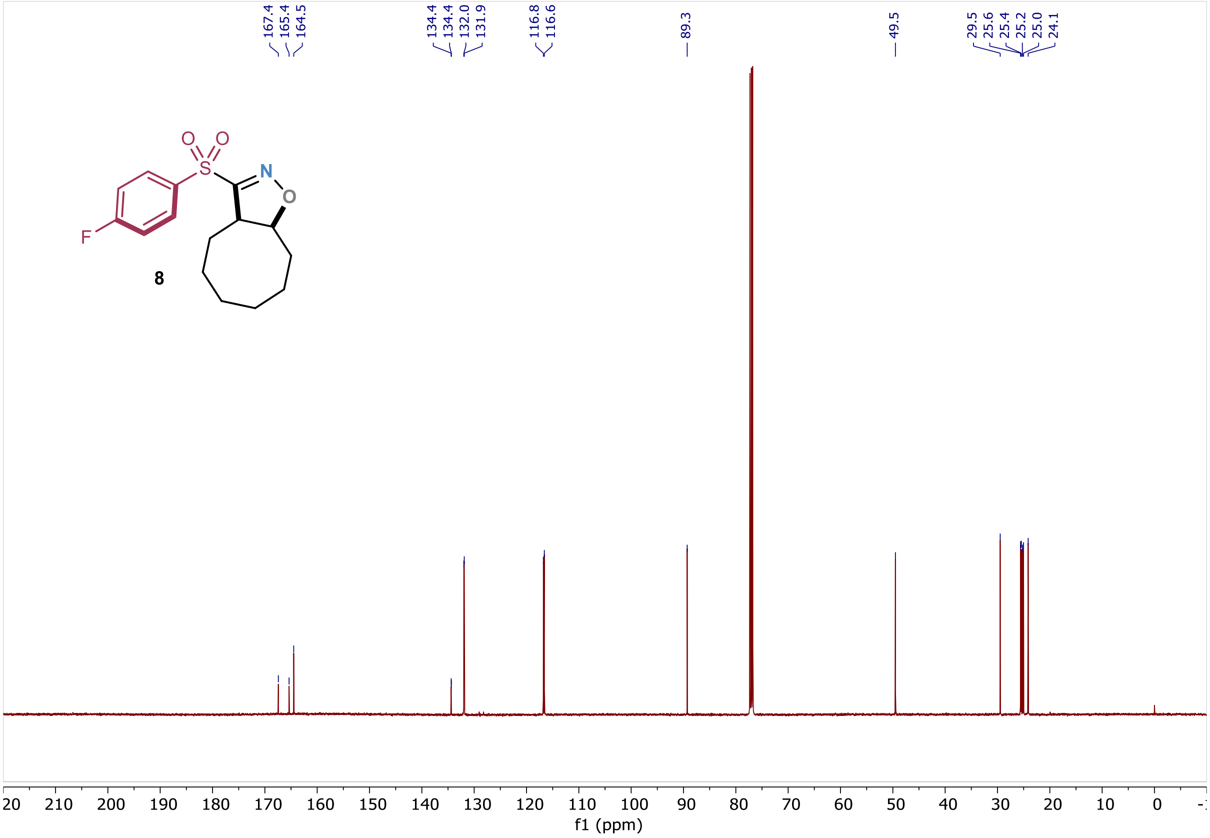

¹⁹F NMR (471 MHz, $CDCl_3$) for compound **8**:

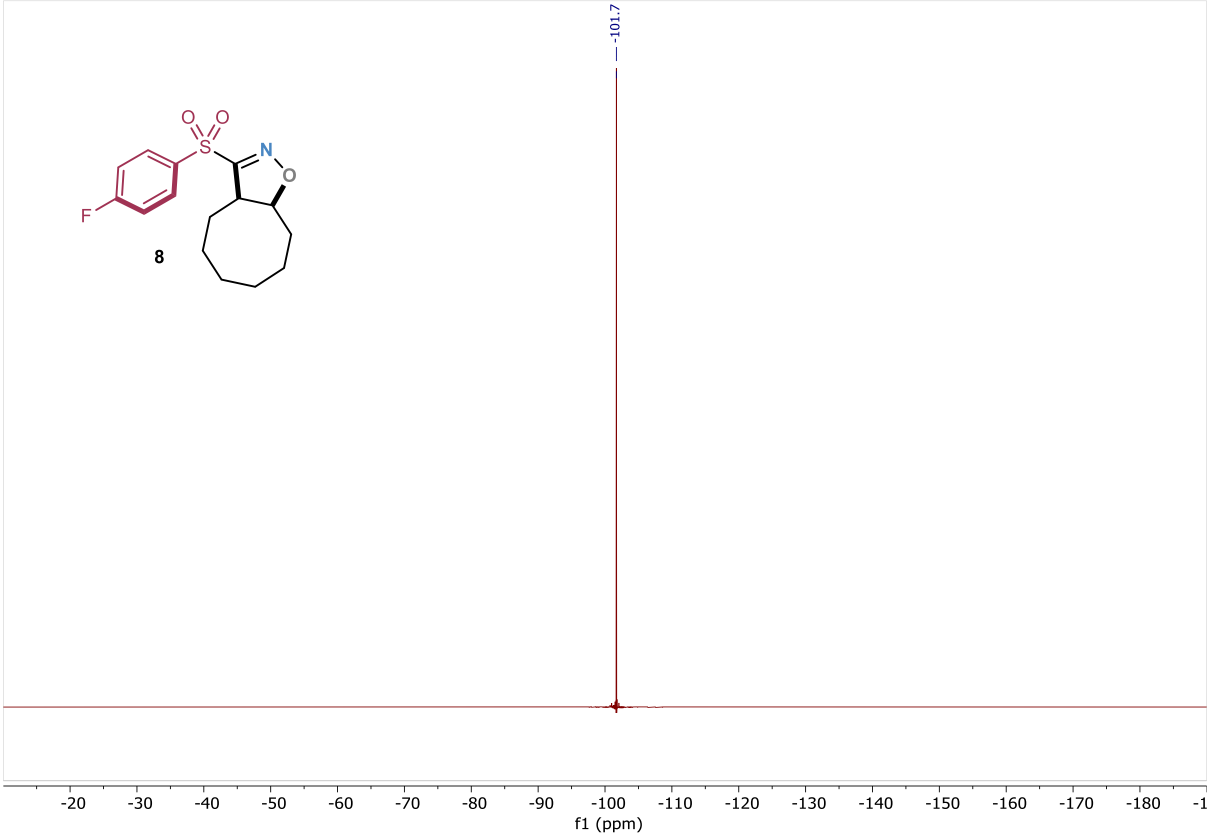

^{1}H NMR (400 MHz, $CDCl_3$) for compound **11**:

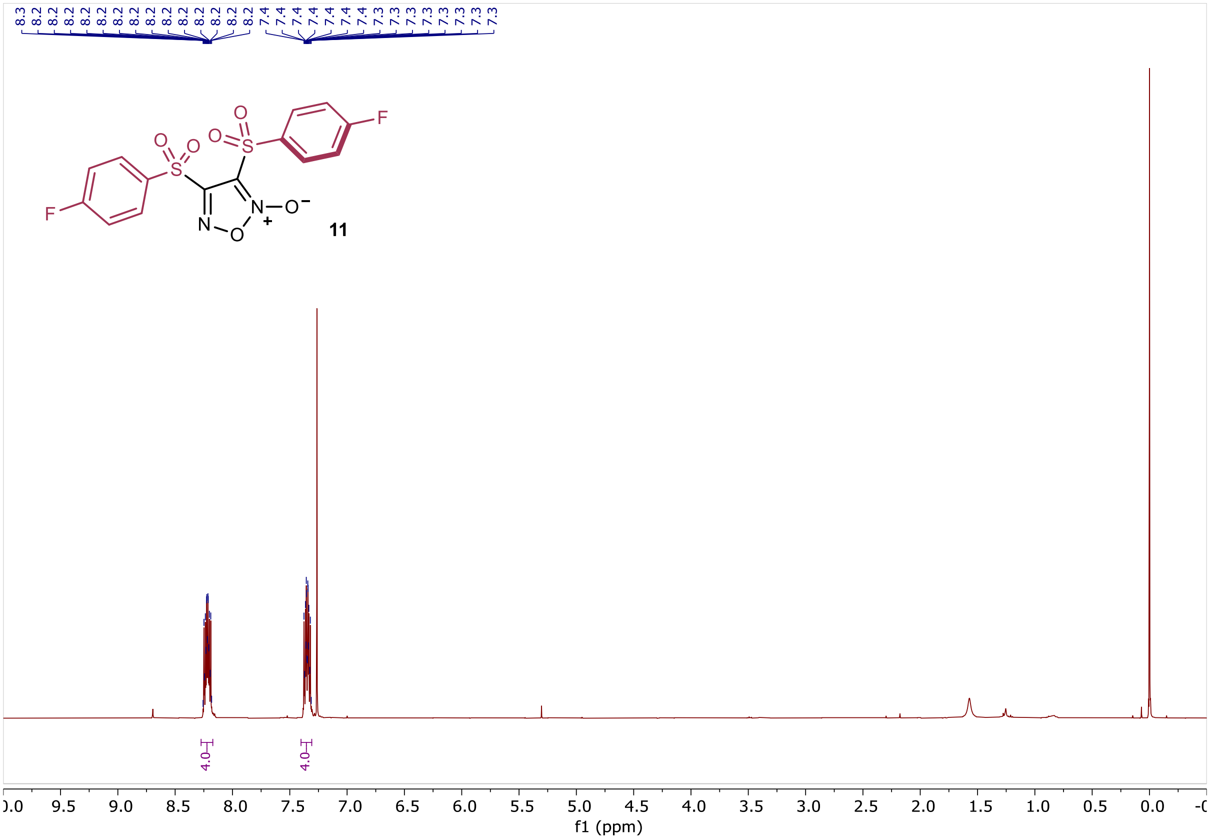

^{13}C NMR (126 MHz, $CDCl_3$) for compound **11**:

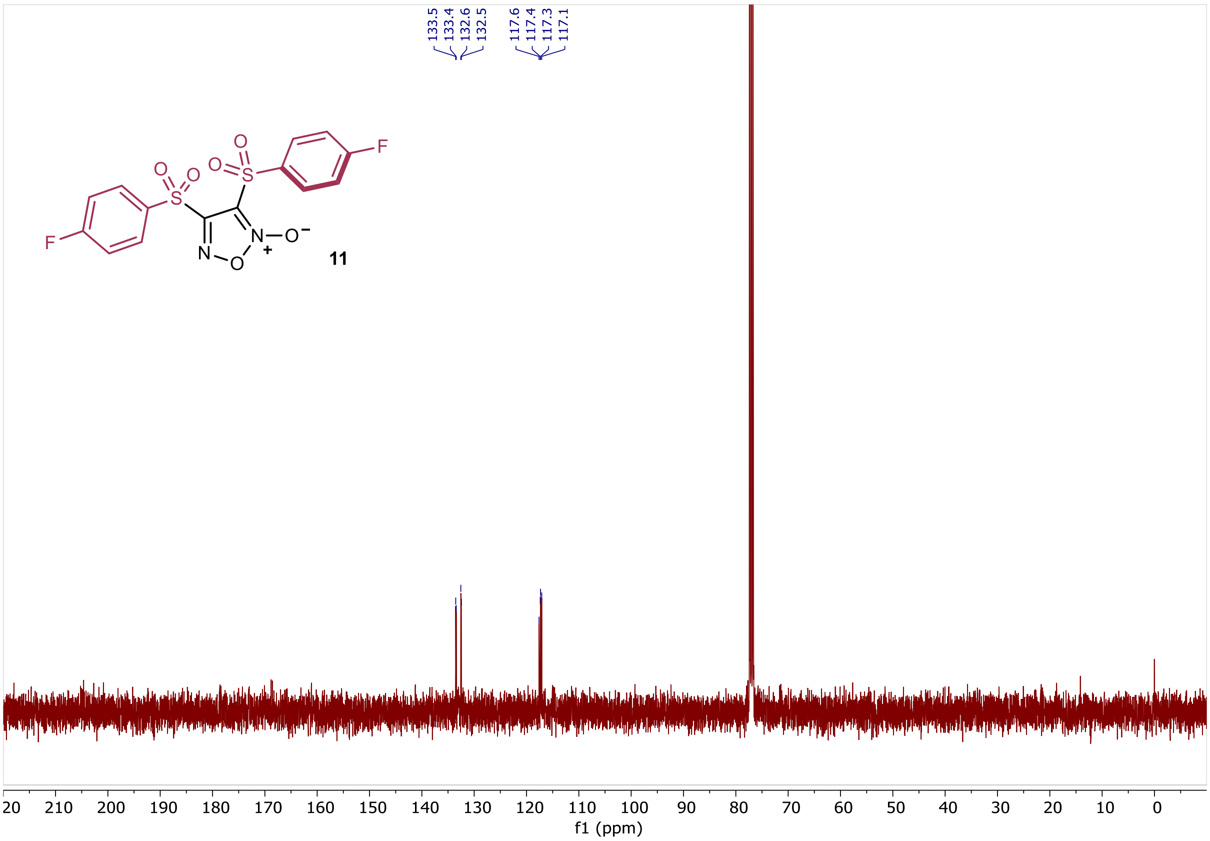

^{19}F NMR (376 MHz, $CDCl_3$) for compound **11**:

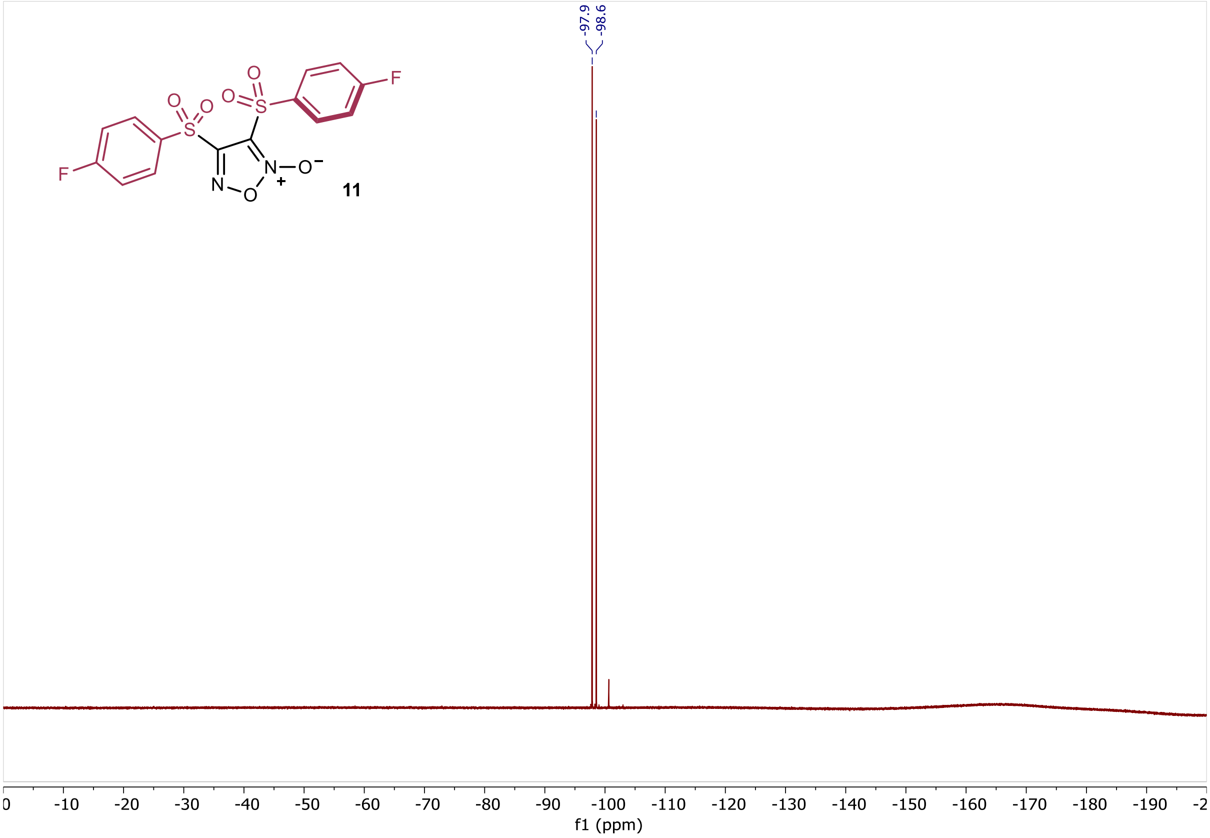

^{1}H NMR (400 MHz, $CDCl_3$) for compound **S-2**:

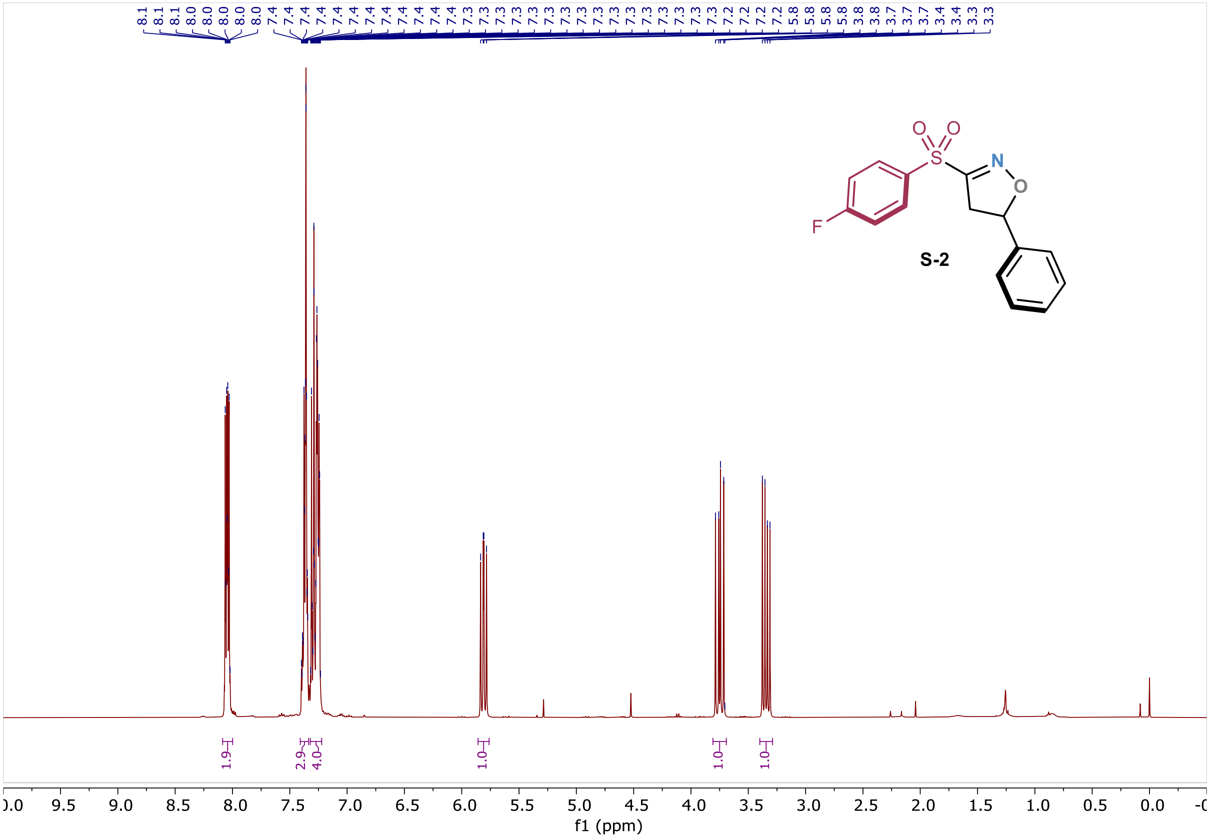

^{13}C NMR (101 MHz, $CDCl_3$) for compound **S-2**:

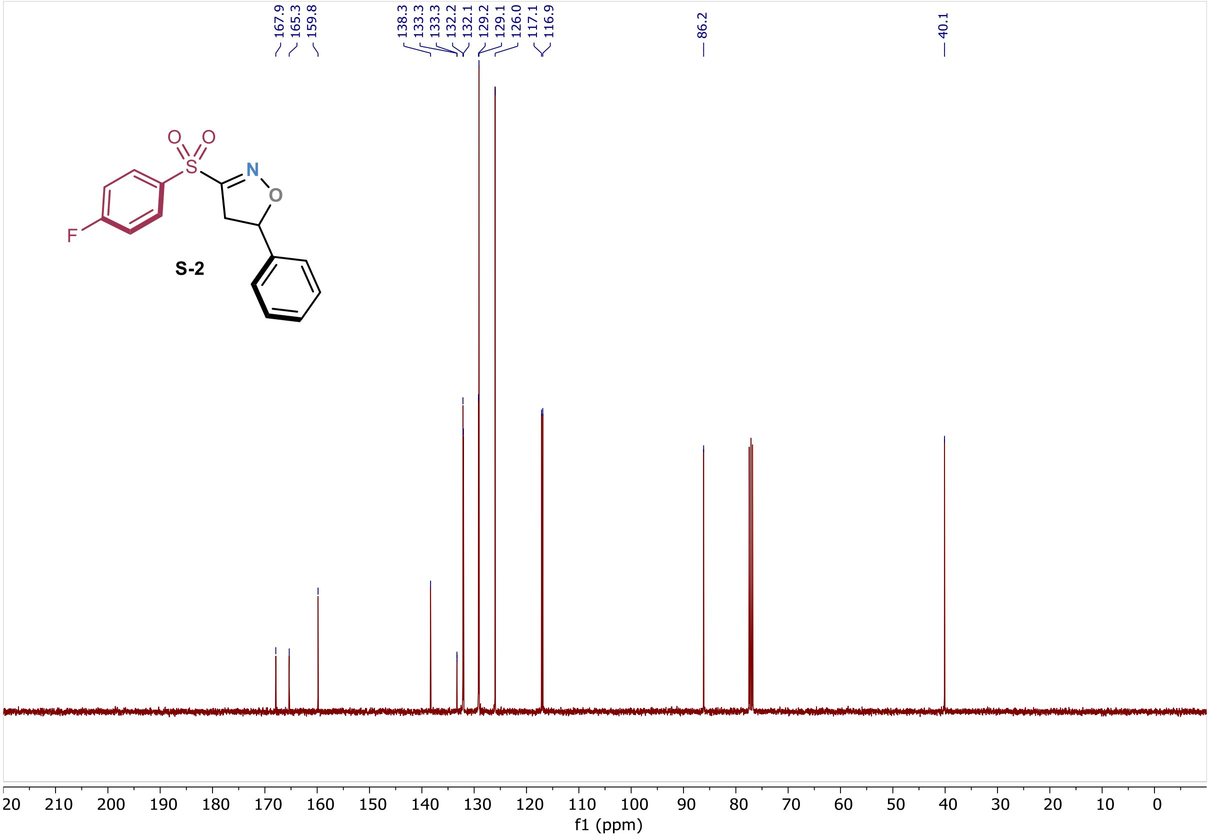

^{19}F NMR (376 MHz, $CDCl_3$) for compound **S-2**:

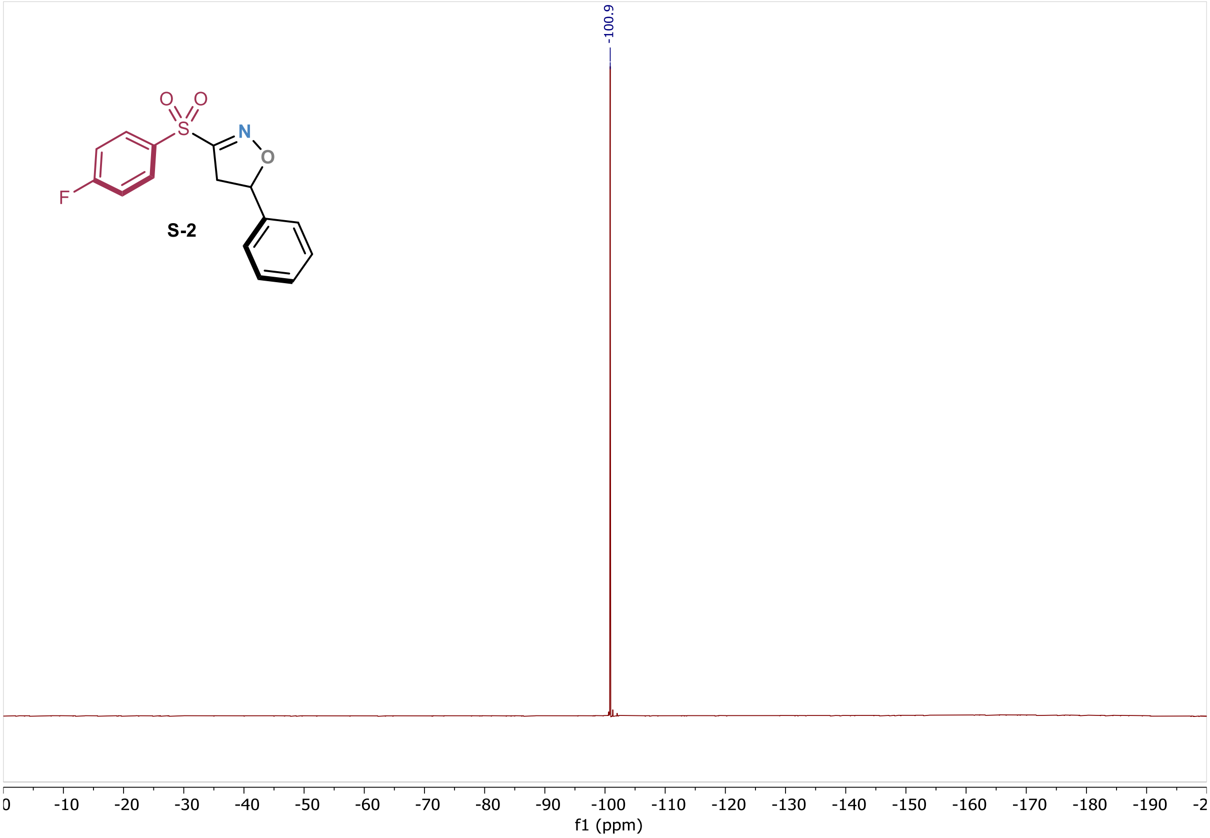

^{1}H NMR (400 MHz, $CDCl_3$) for compound **S-3**:

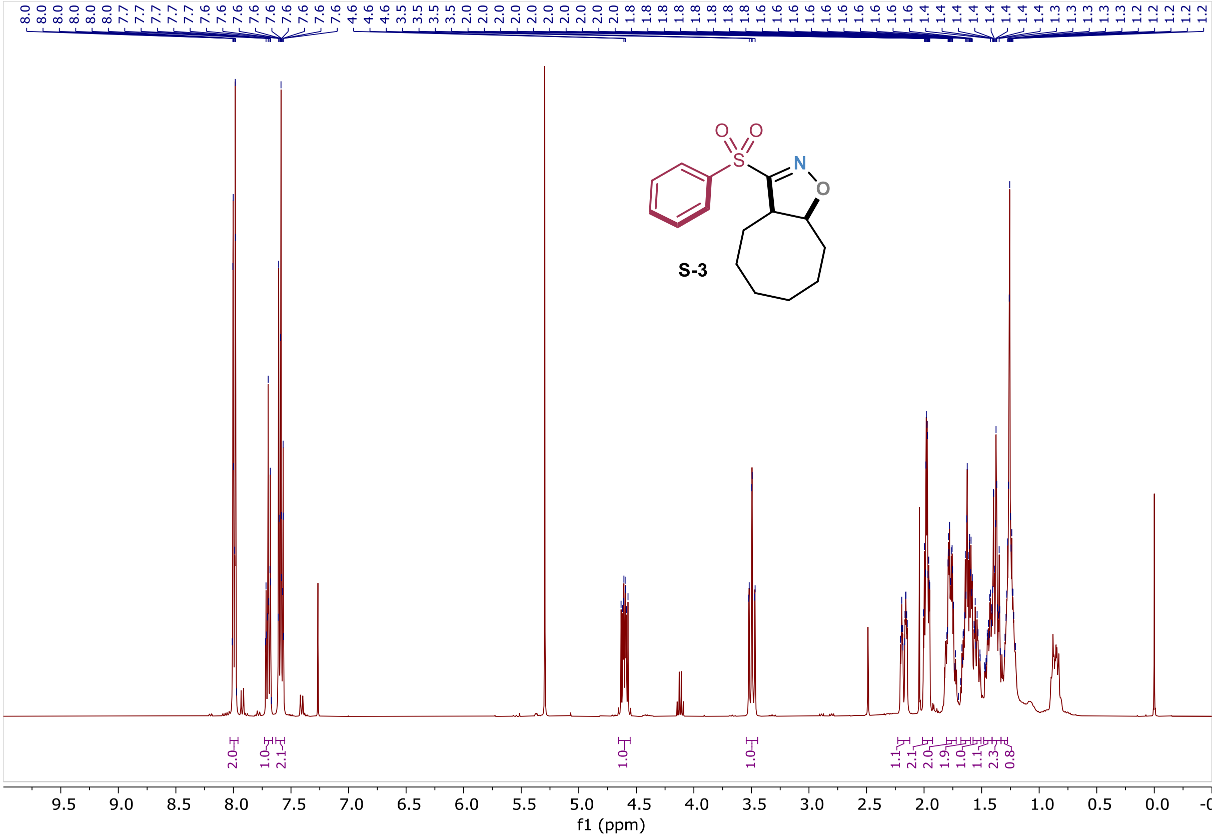

^{13}C NMR (101 MHz, $CDCl_3$) for compound **S-3**:

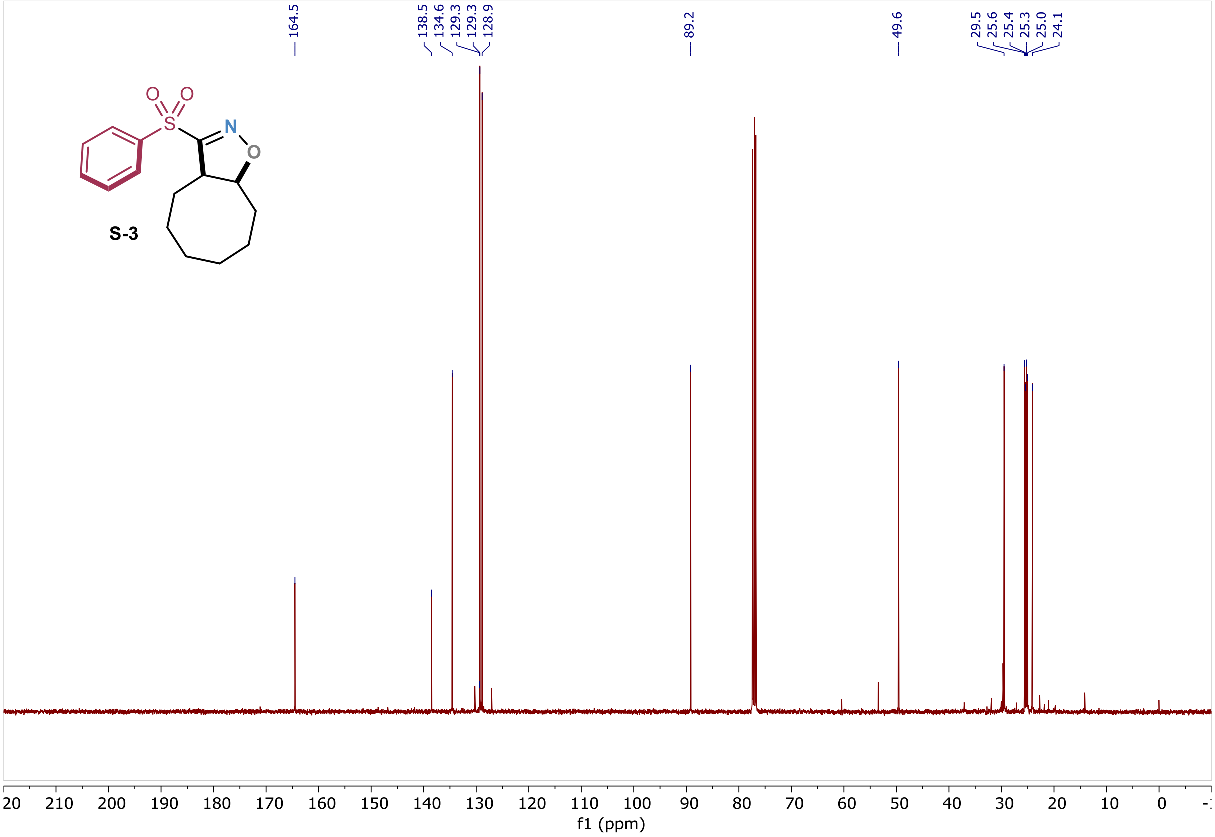

^{1}H NMR (500 MHz, $CDCl_3$) for compound **12**:

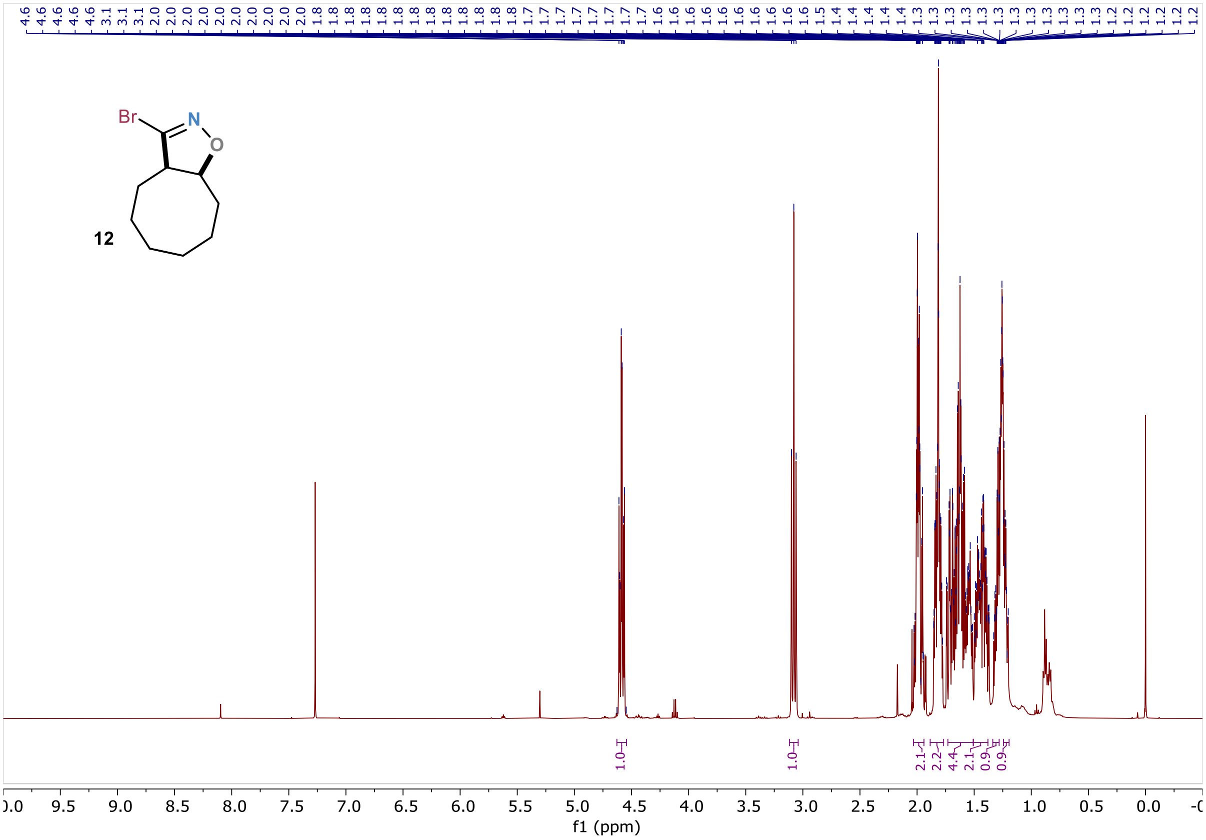

^{13}C NMR (126 MHz, $CDCl_3$) for compound **12**:

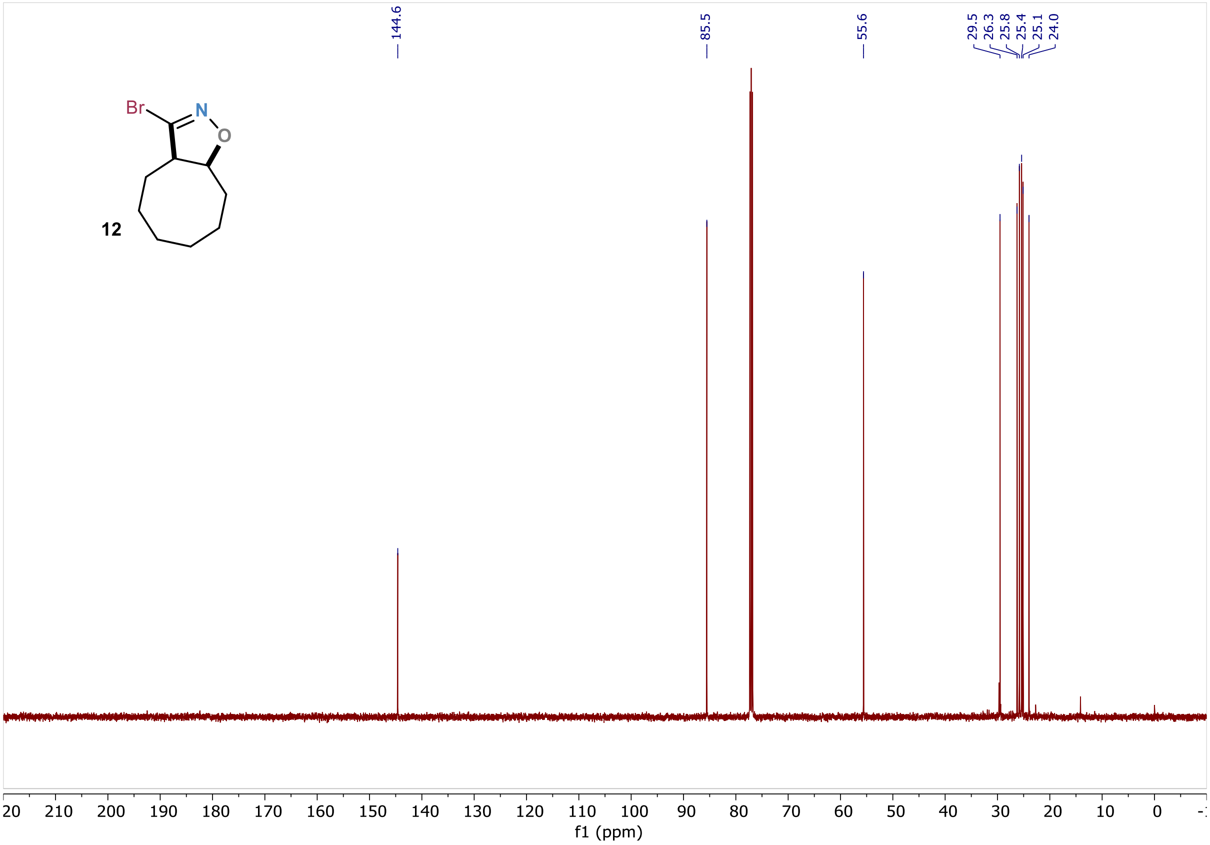

^{1}H NMR (400 MHz, $CDCl_3$) for compound **13**:

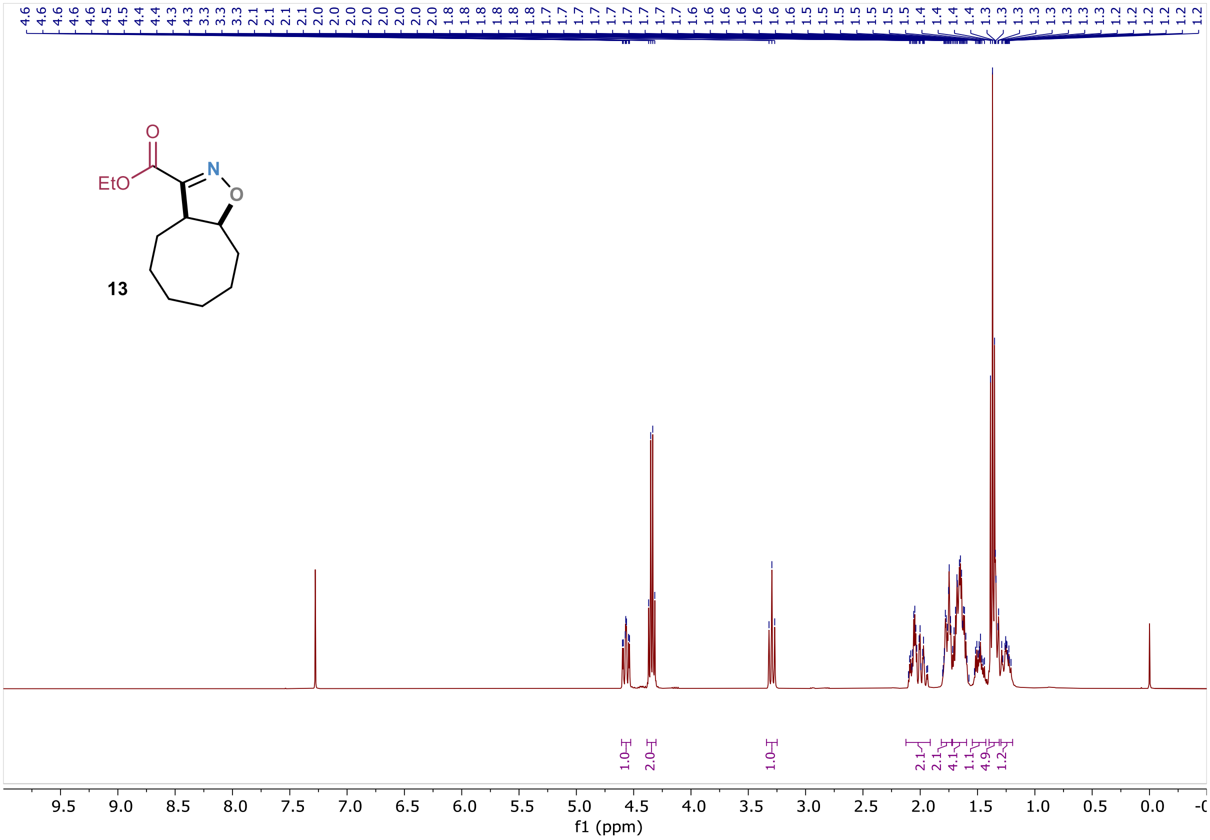

^{13}C NMR (101 MHz, $CDCl_3$) for compound **13**:

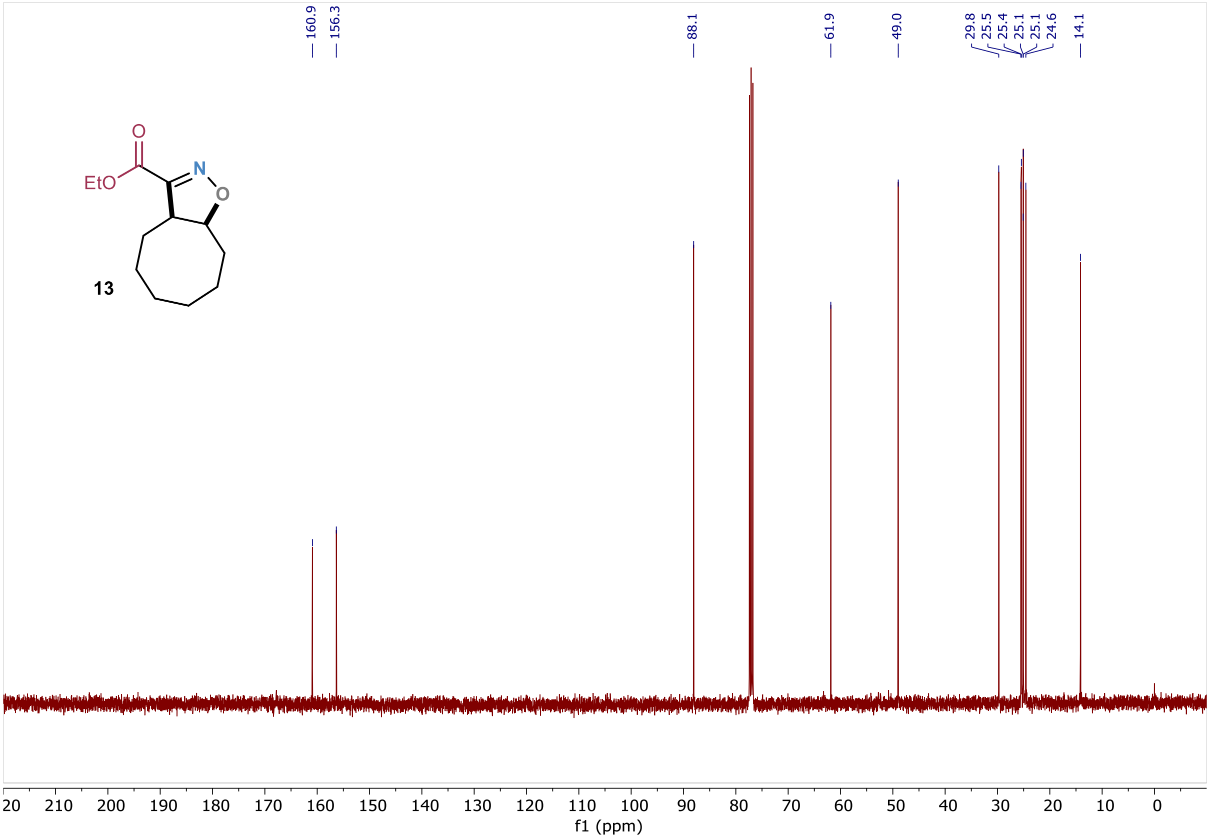

¹H NMR (400 MHz, $CDCl_3$) for compound **S-4**:

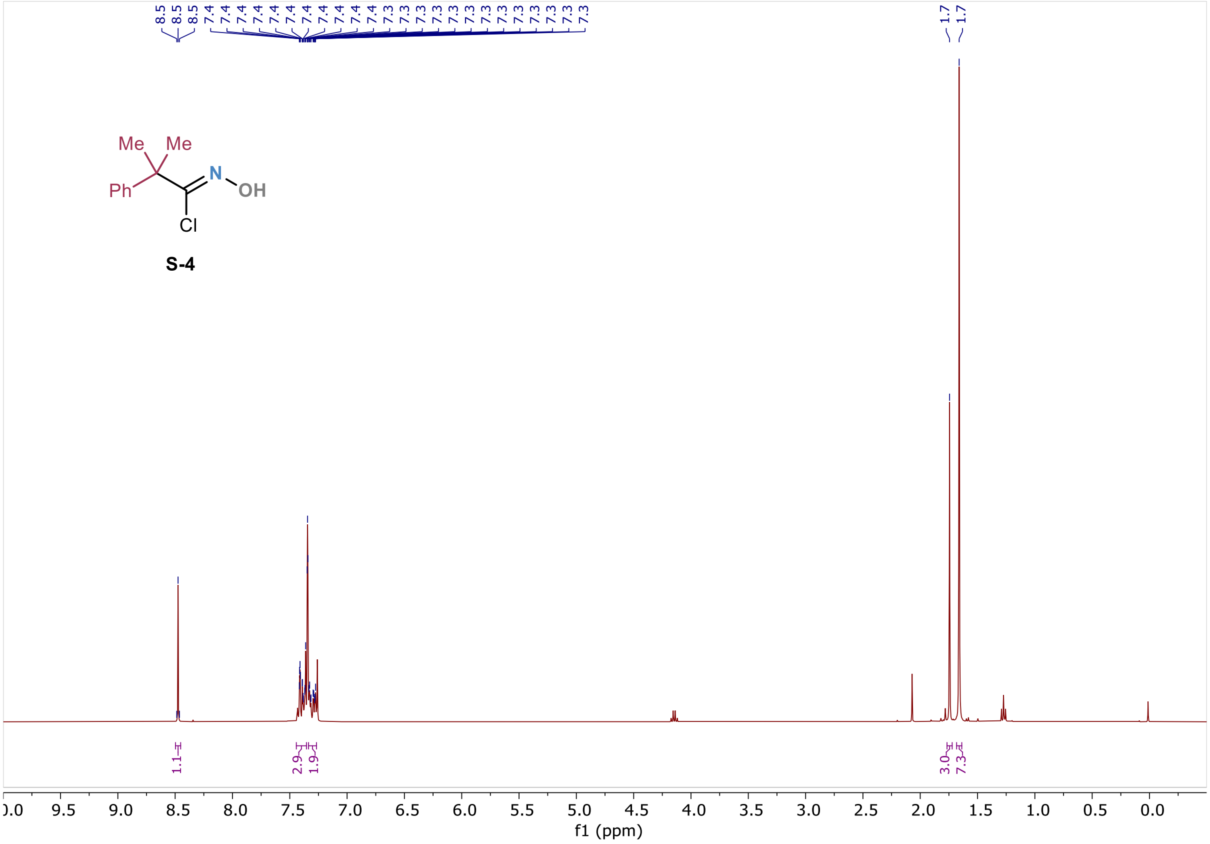

¹³C NMR (101 MHz, $CDCl_3$) for compound **S-4**:

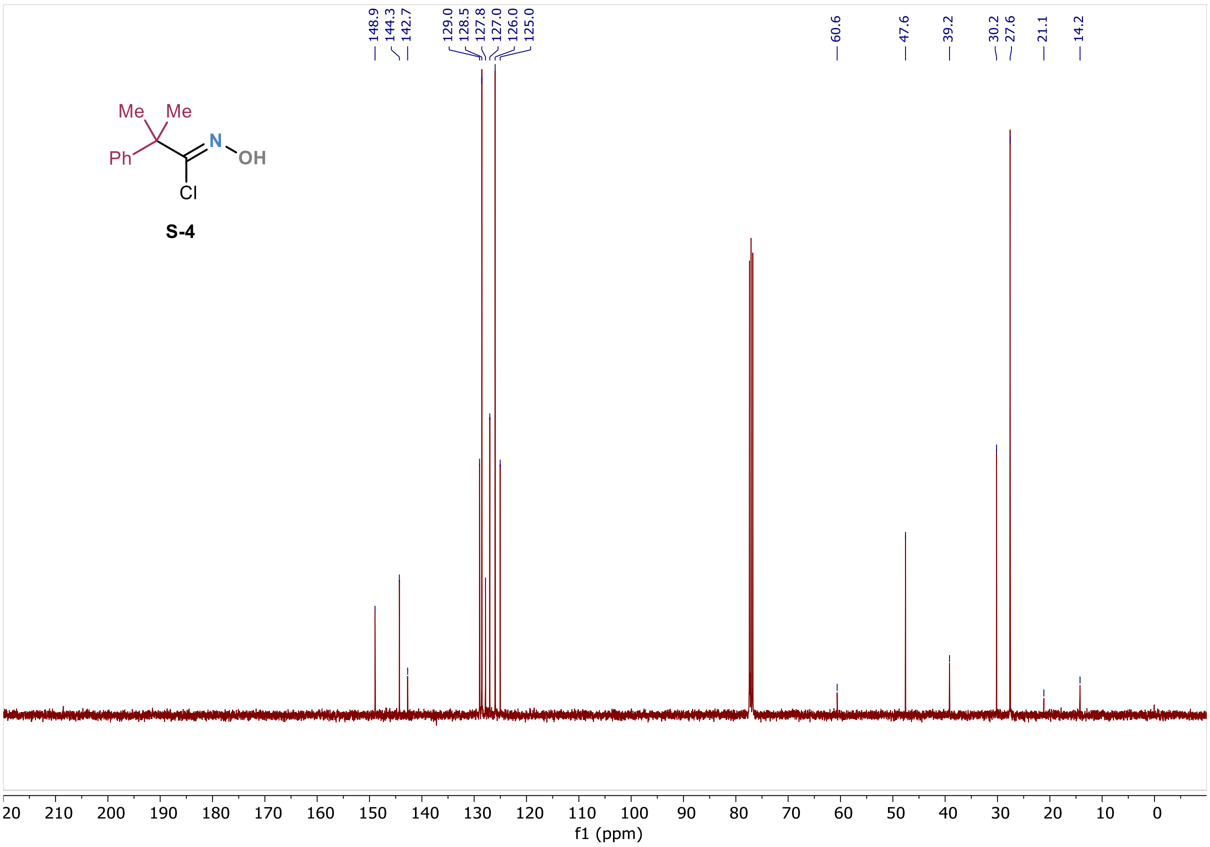

^{1}H NMR (400 MHz, $CDCl_3$) for compound **14**:

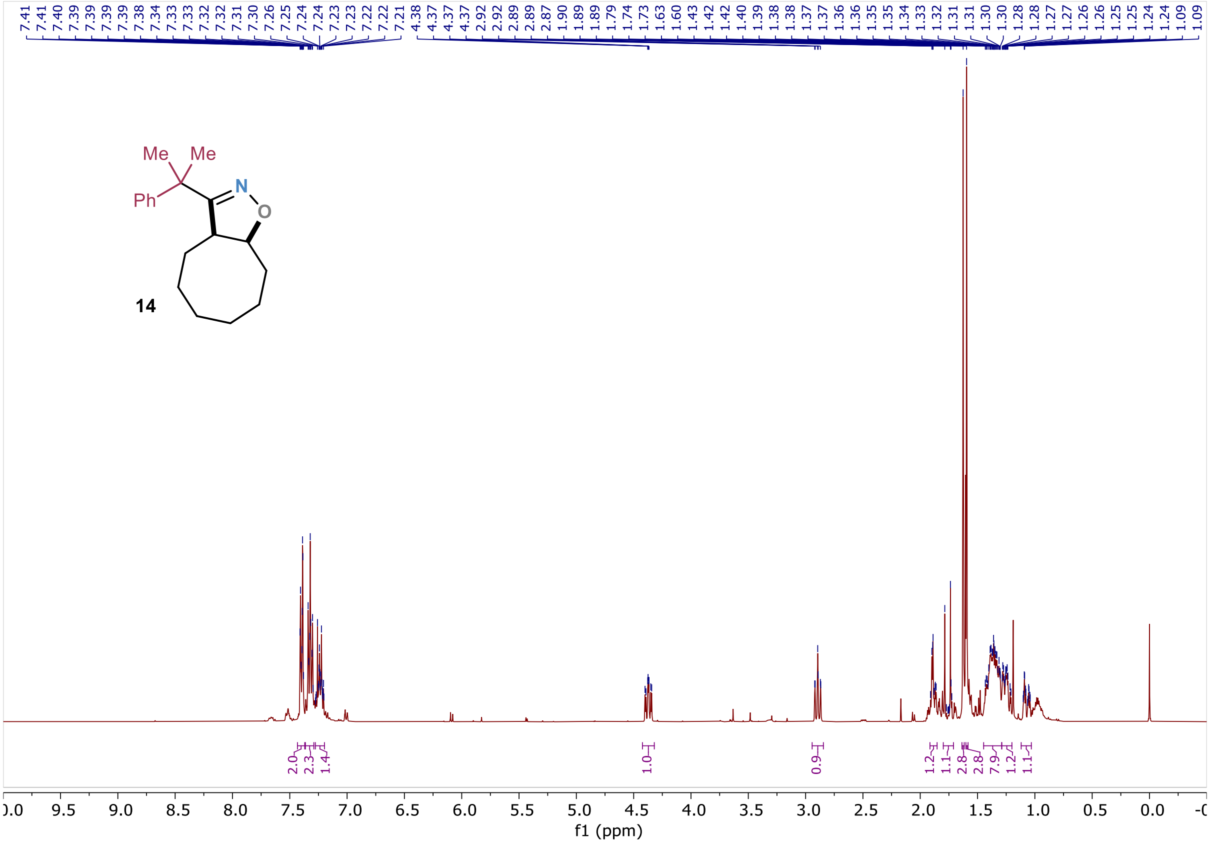

^{13}C NMR (101 MHz, $CDCl_3$) for compound **14**:

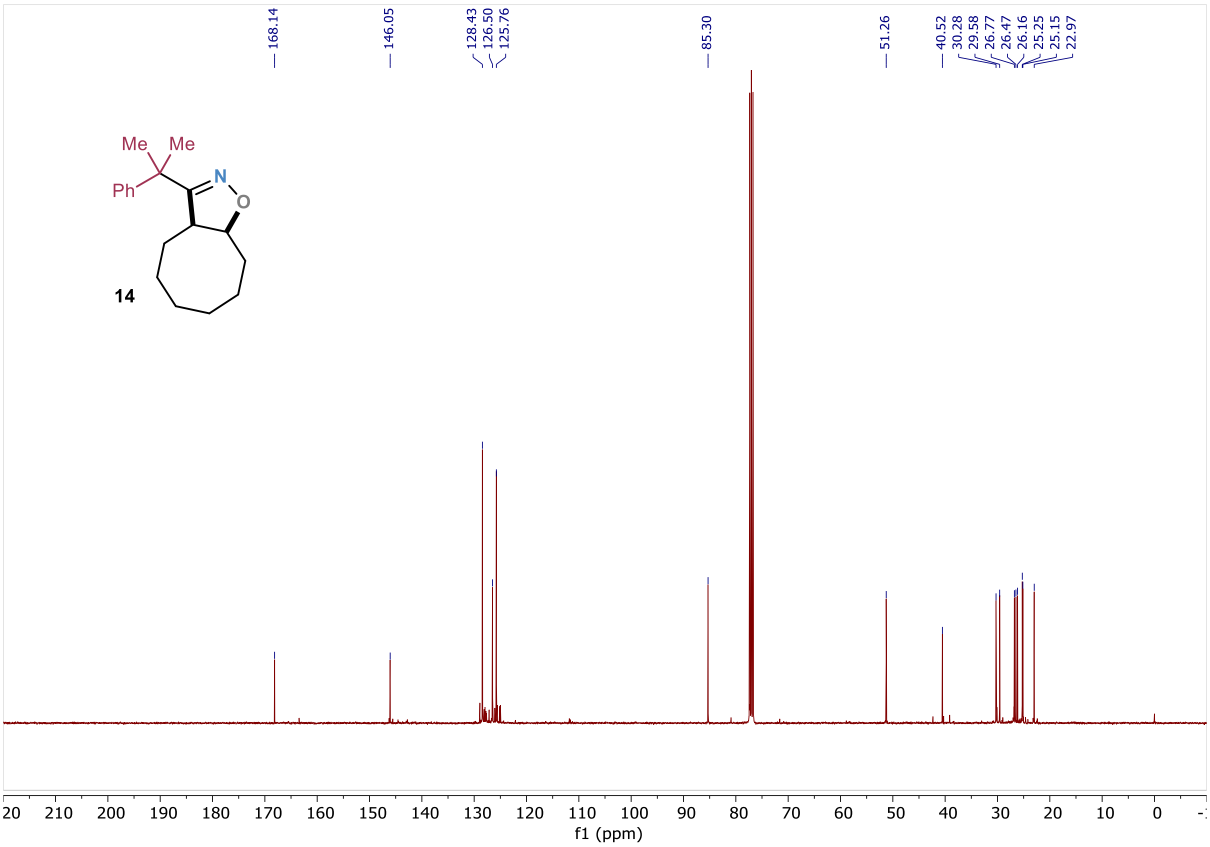

^{1}H NMR (400 MHz, $CDCl_3$) for compound **15**:

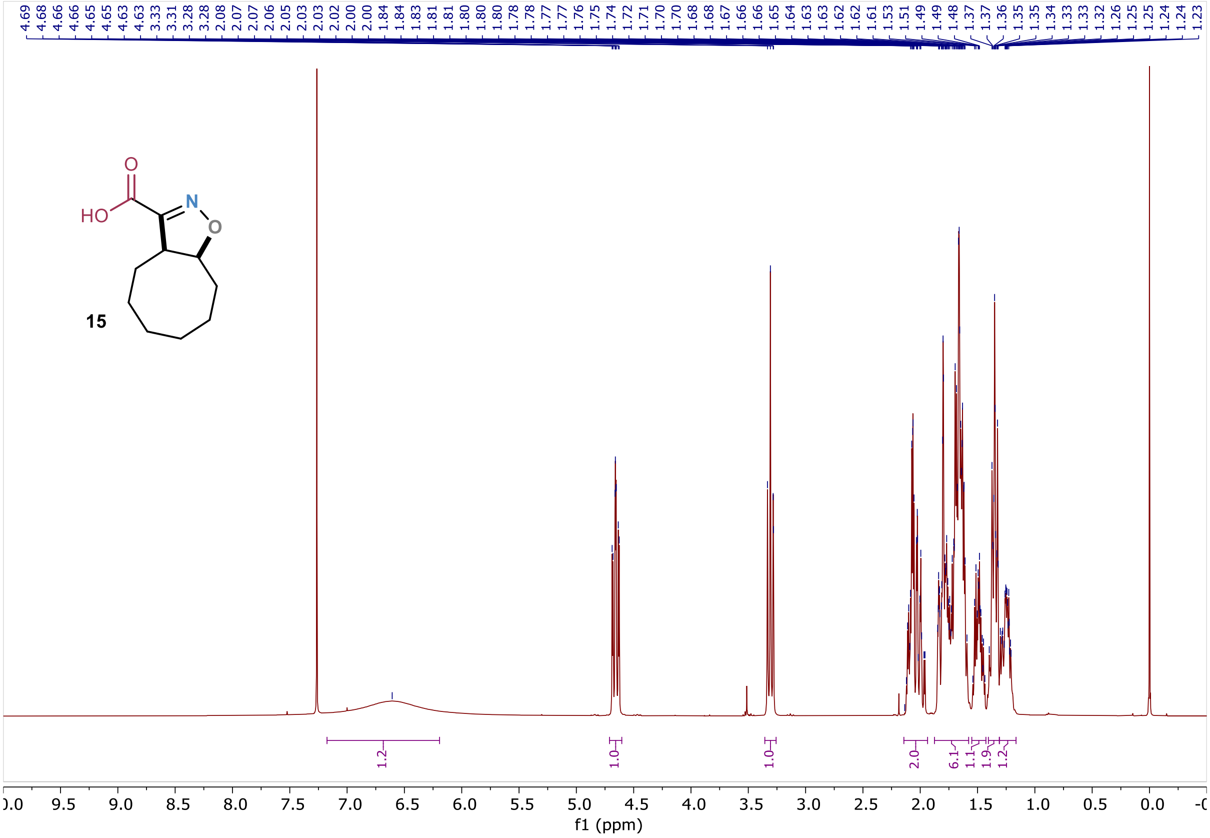

^{13}C NMR (126 MHz, DMSO-d_6) for compound **15**:

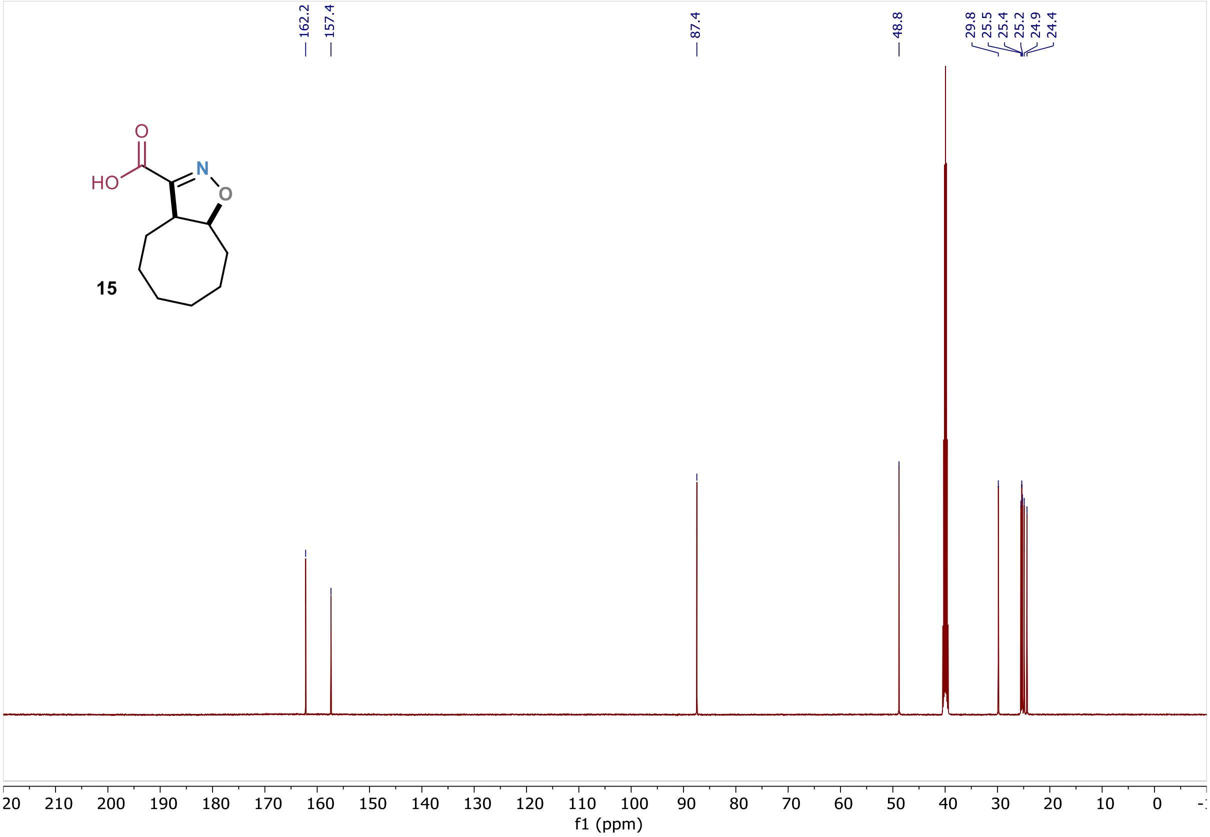

^{1}H NMR (400 MHz, $CDCl_3$) for compound **16**:

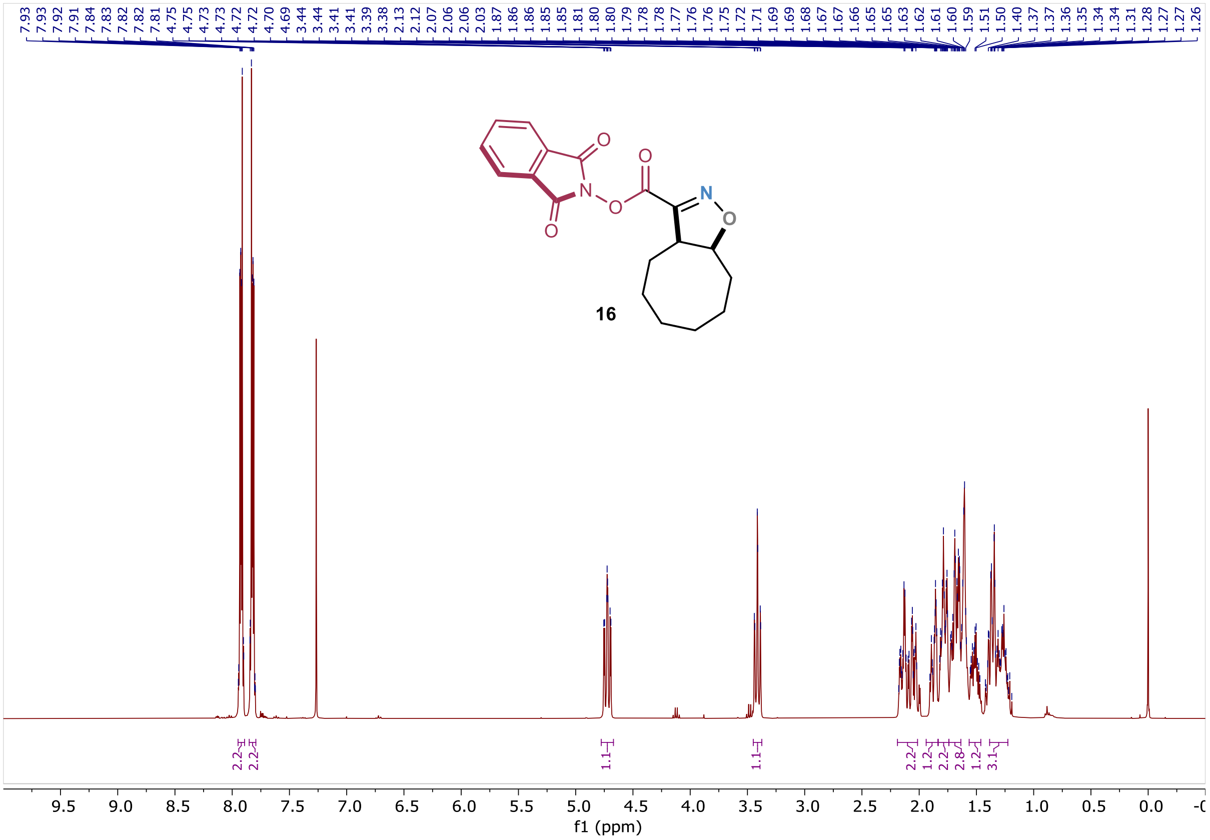

^{13}C NMR (126 MHz, DMSO-d_6) for compound **16**:

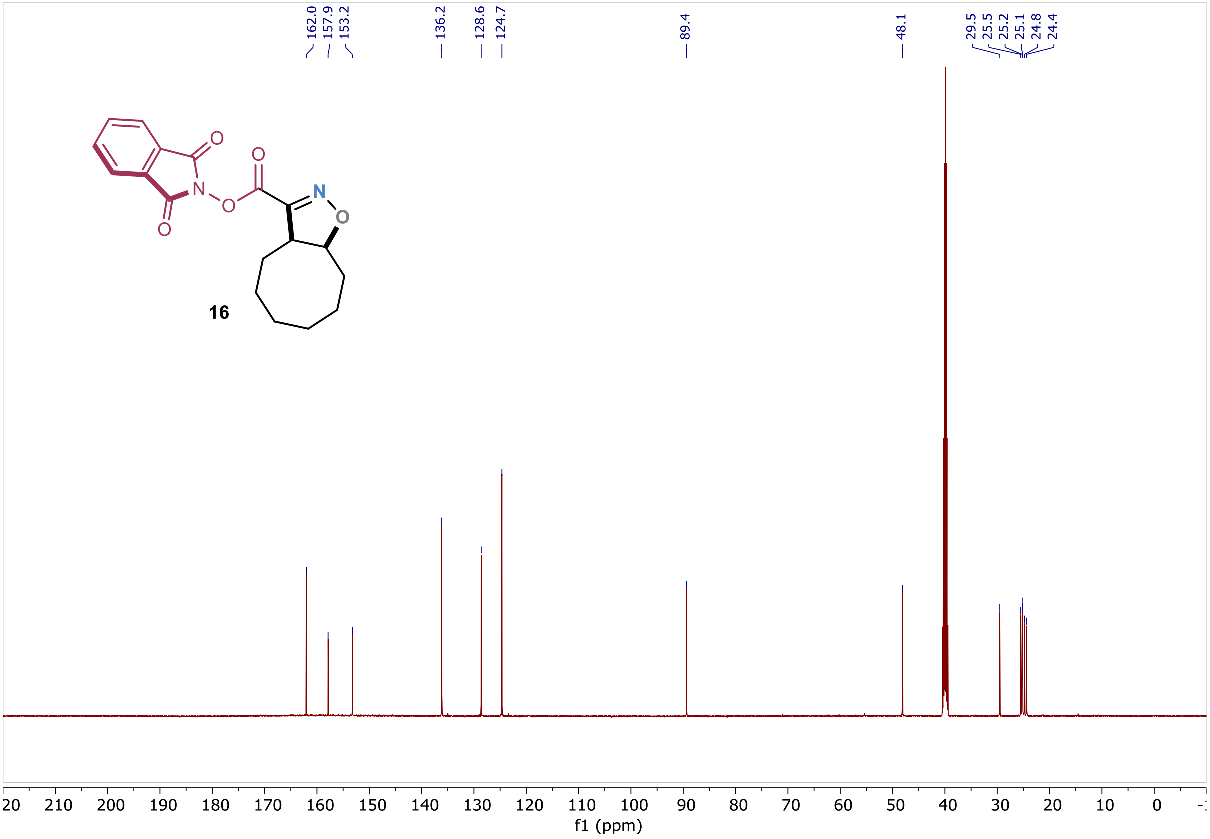

¹H NMR (500 MHz, $CDCl_3$) for compound **18**:

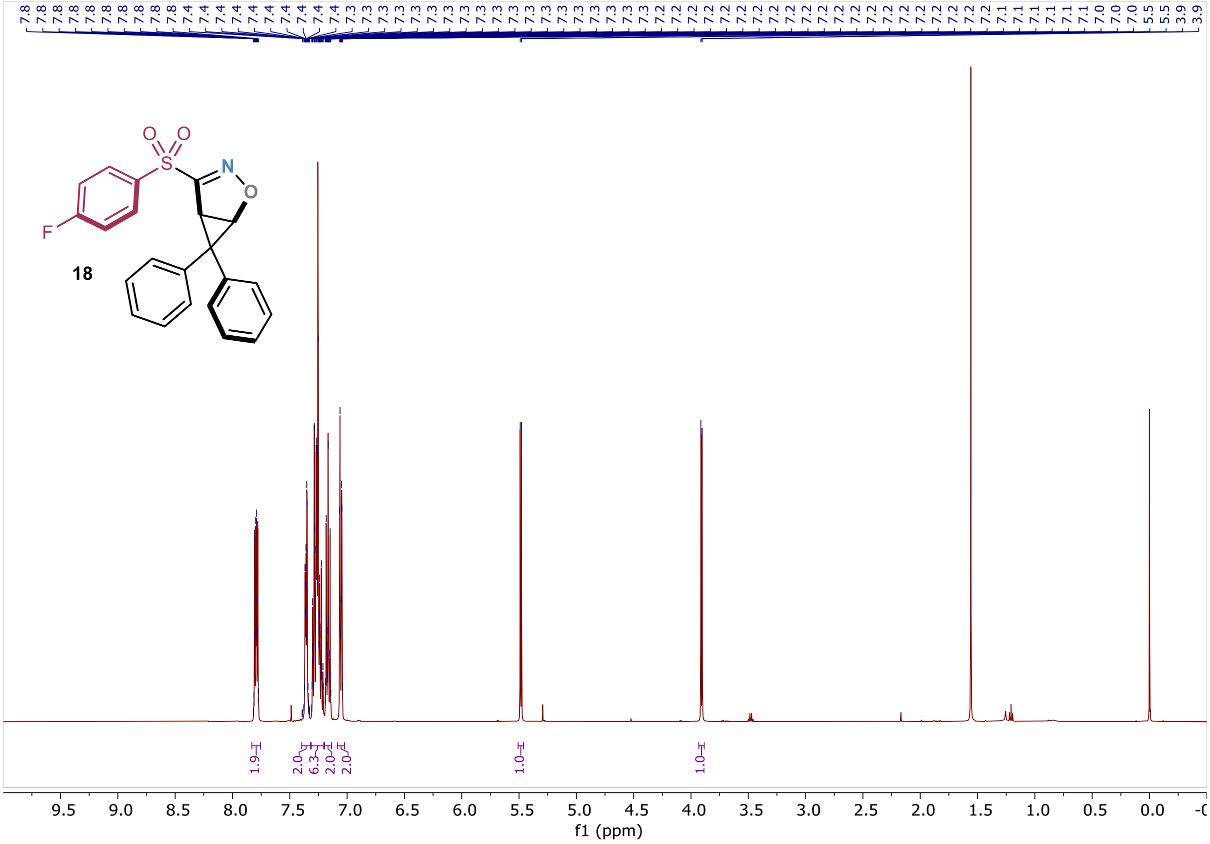

¹³C NMR (126 MHz, $CDCl_3$) for compound **18**:

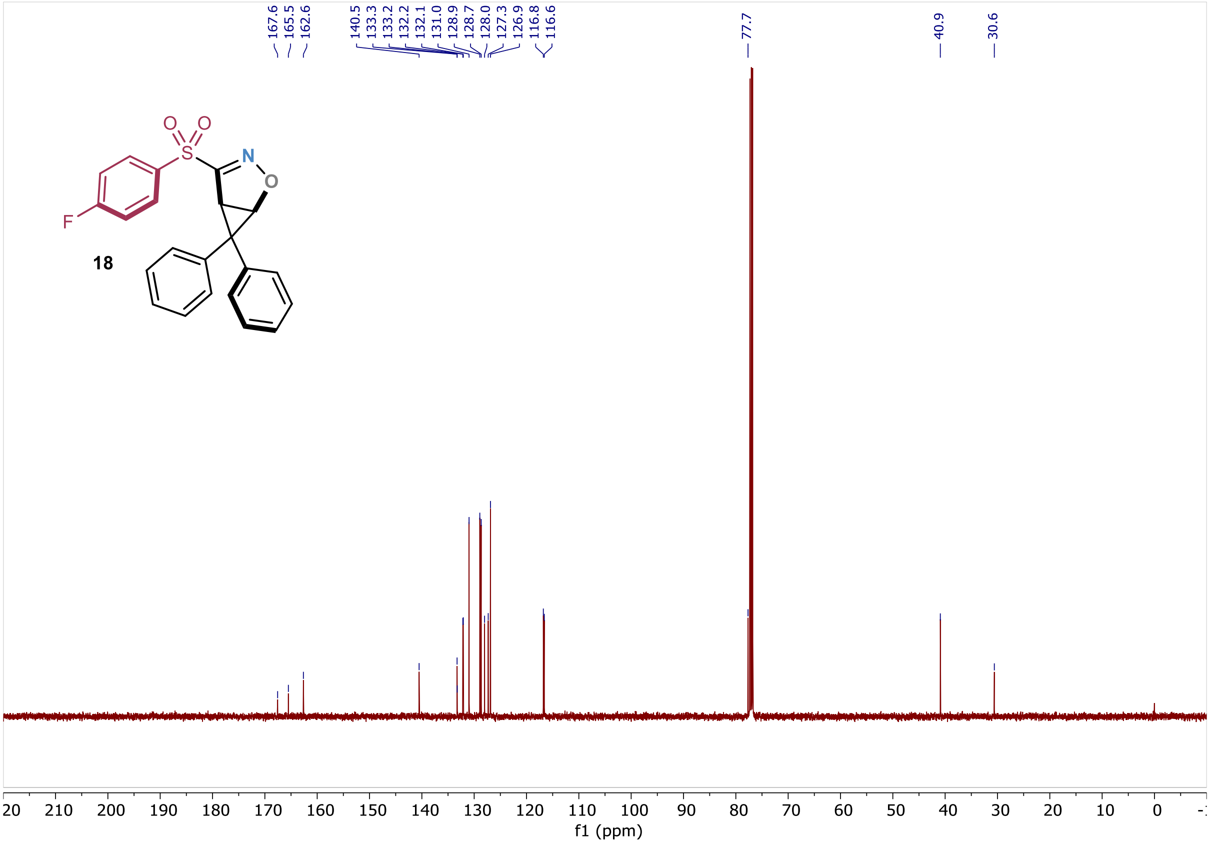

^{19}F NMR (471 MHz, $CDCl_3$) for compound **18**:

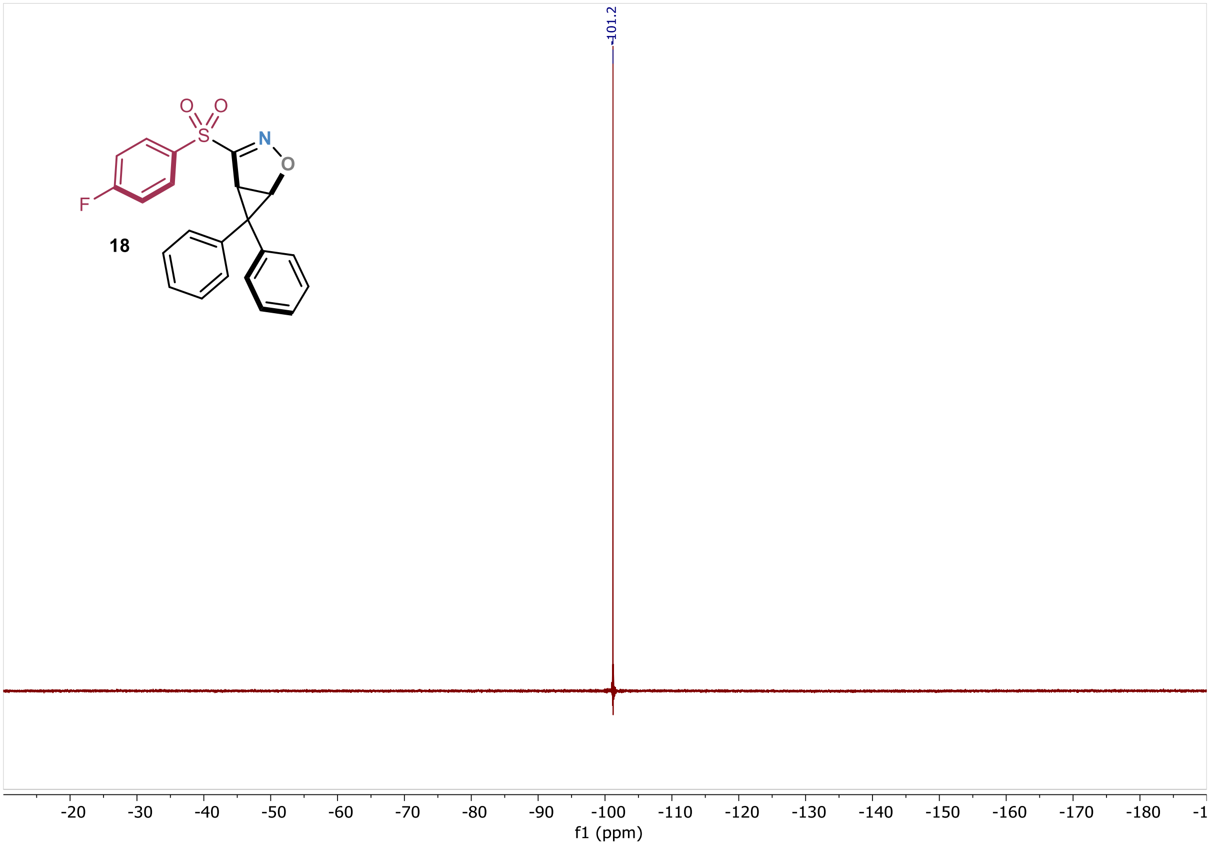

^{1}H NMR (500 MHz, $CDCl_3$) for compound **23**:

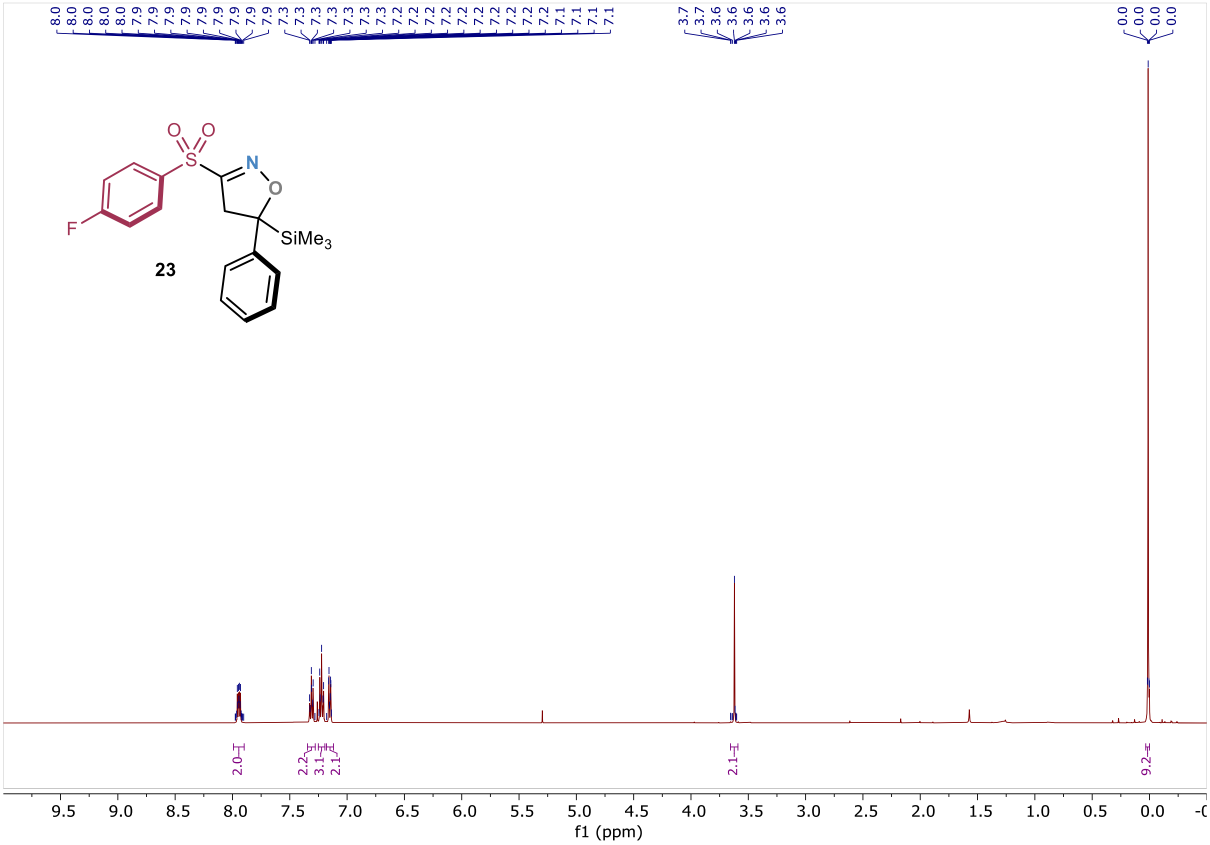

^{13}C NMR (126 MHz, $CDCl_3$) for compound **23**:

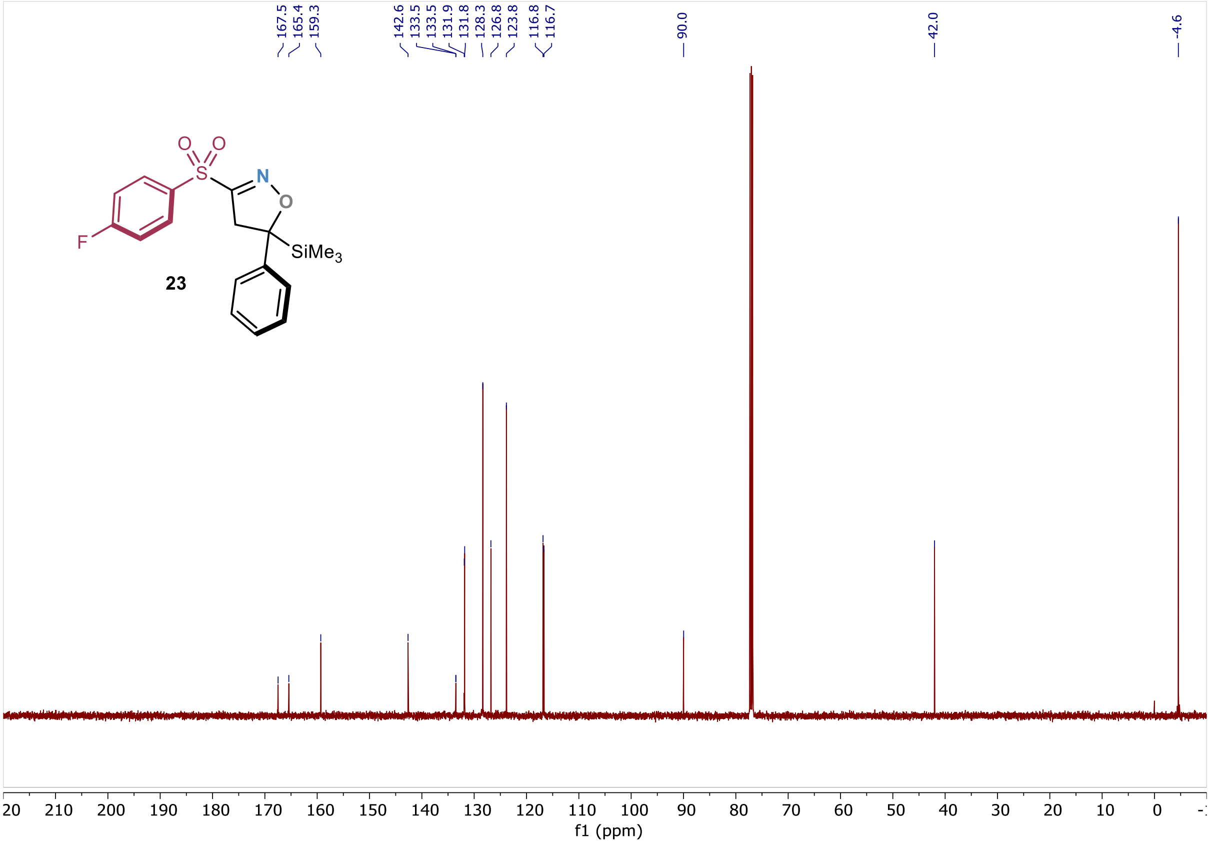

^{19}F NMR (471 MHz, $CDCl_3$) for compound **23**:

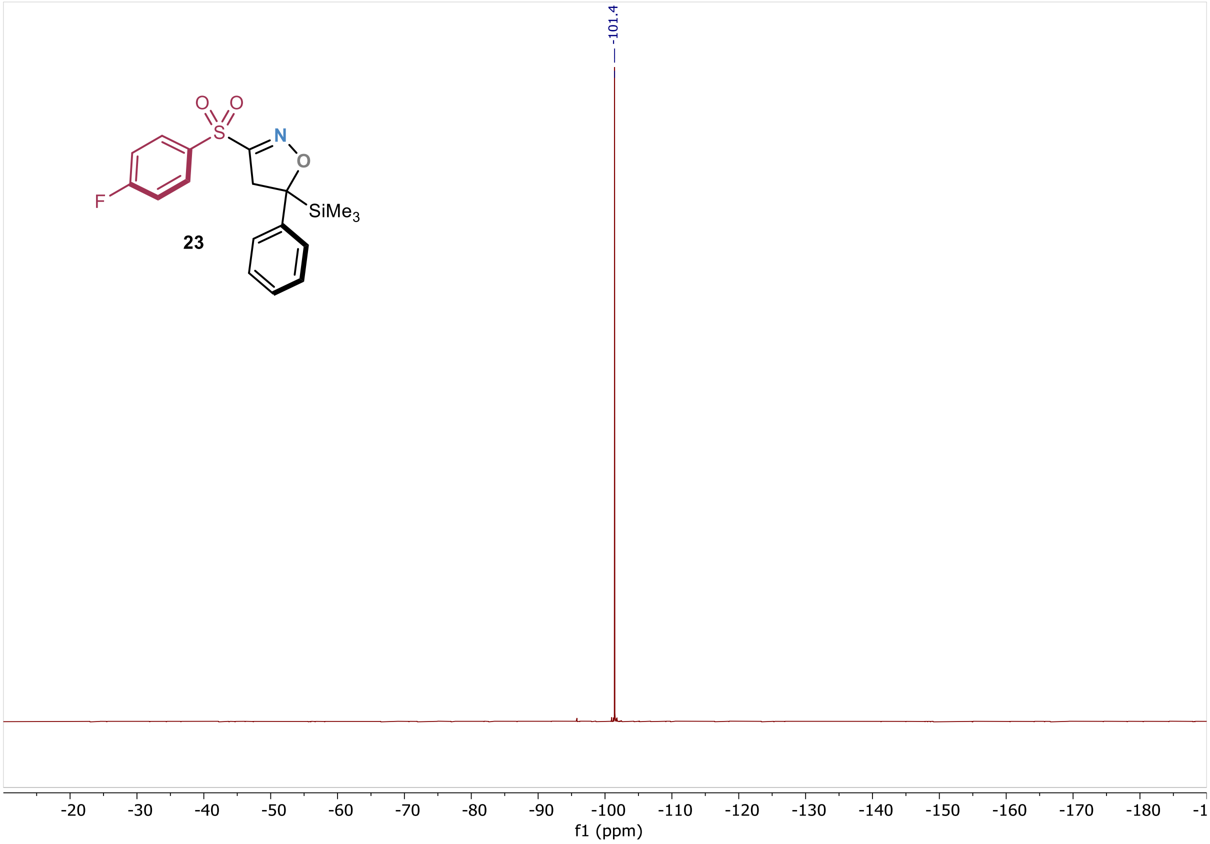

^{1}H NMR (500 MHz, $CDCl_3$) for compound **19**:

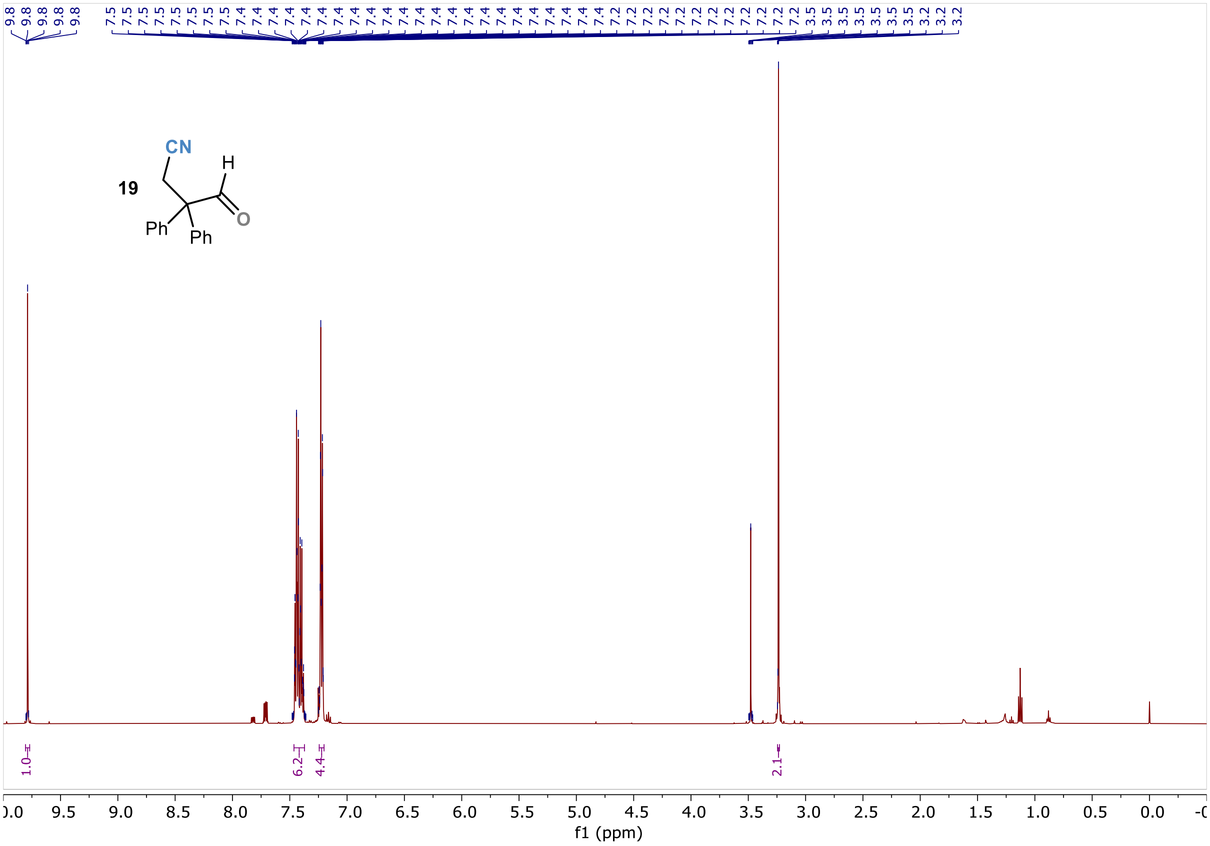

^{13}C NMR (126 MHz, $CDCl_3$) for compound **19**:

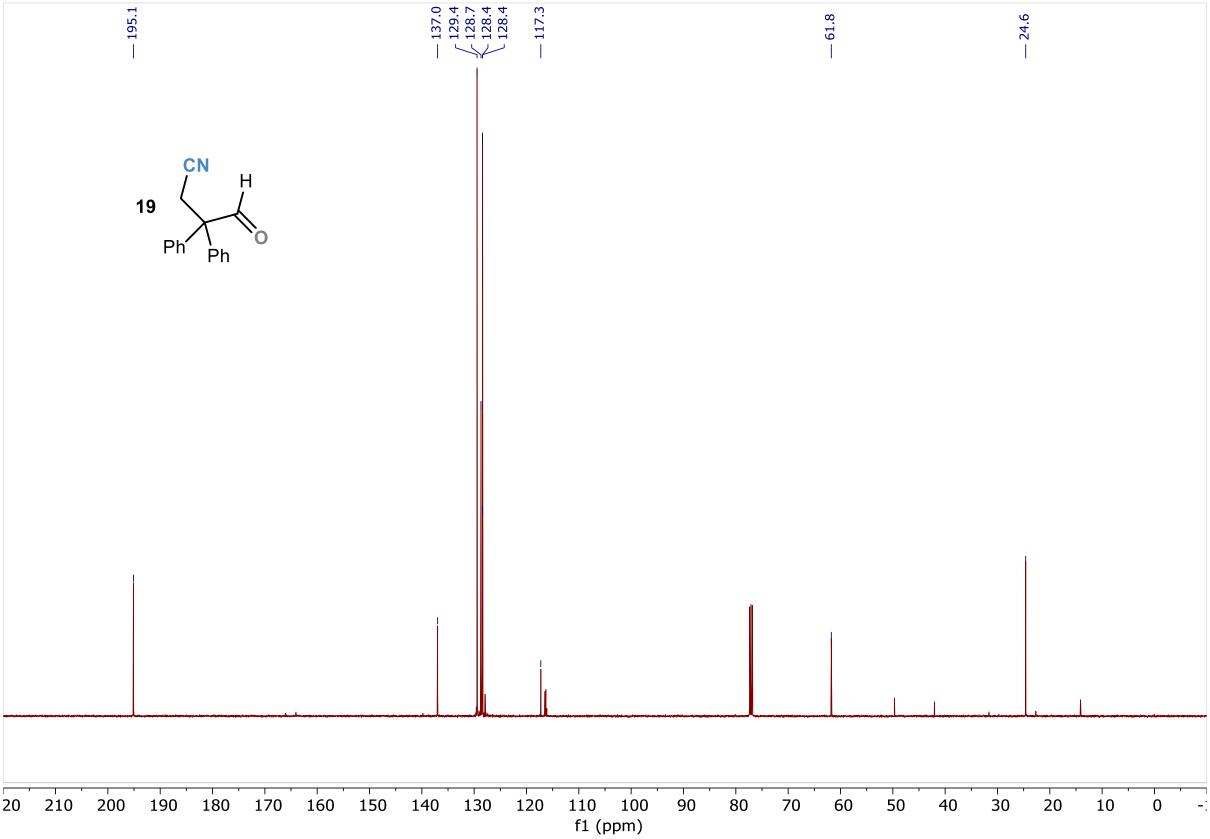

^{1}H NMR (500 MHz, $CDCl_3$) for compound **20**:

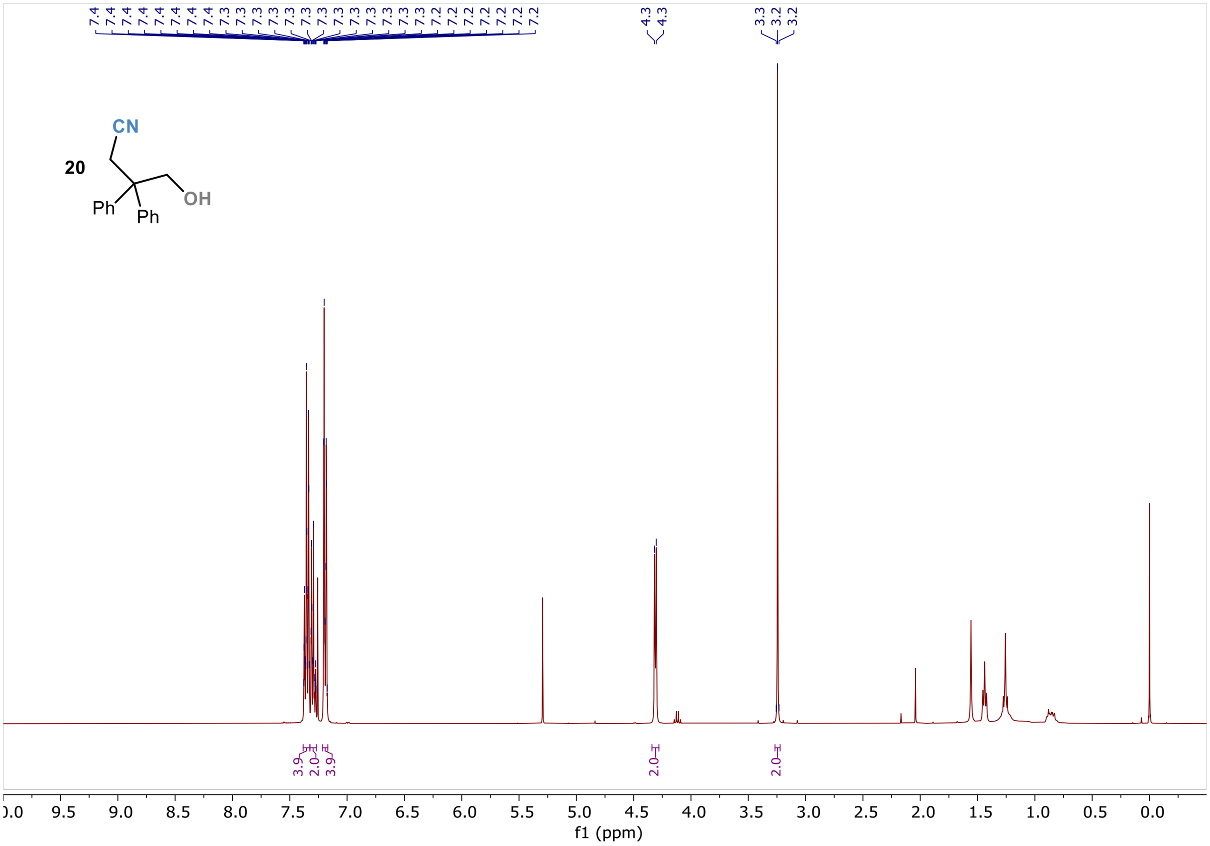

^{13}C NMR (126 MHz, $CDCl_3$) for compound **20**:

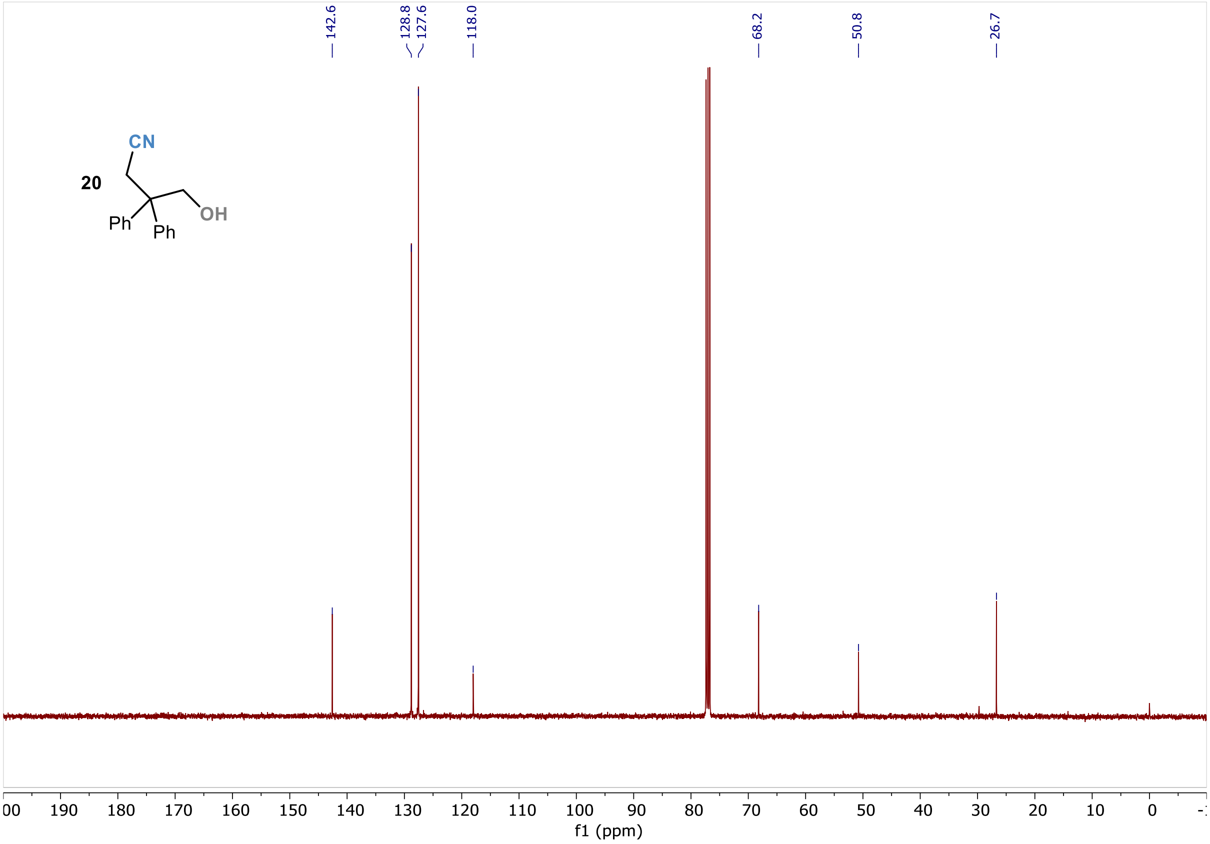

^{1}**H NMR** (400 MHz, $CDCl_3$) for compound **24**:

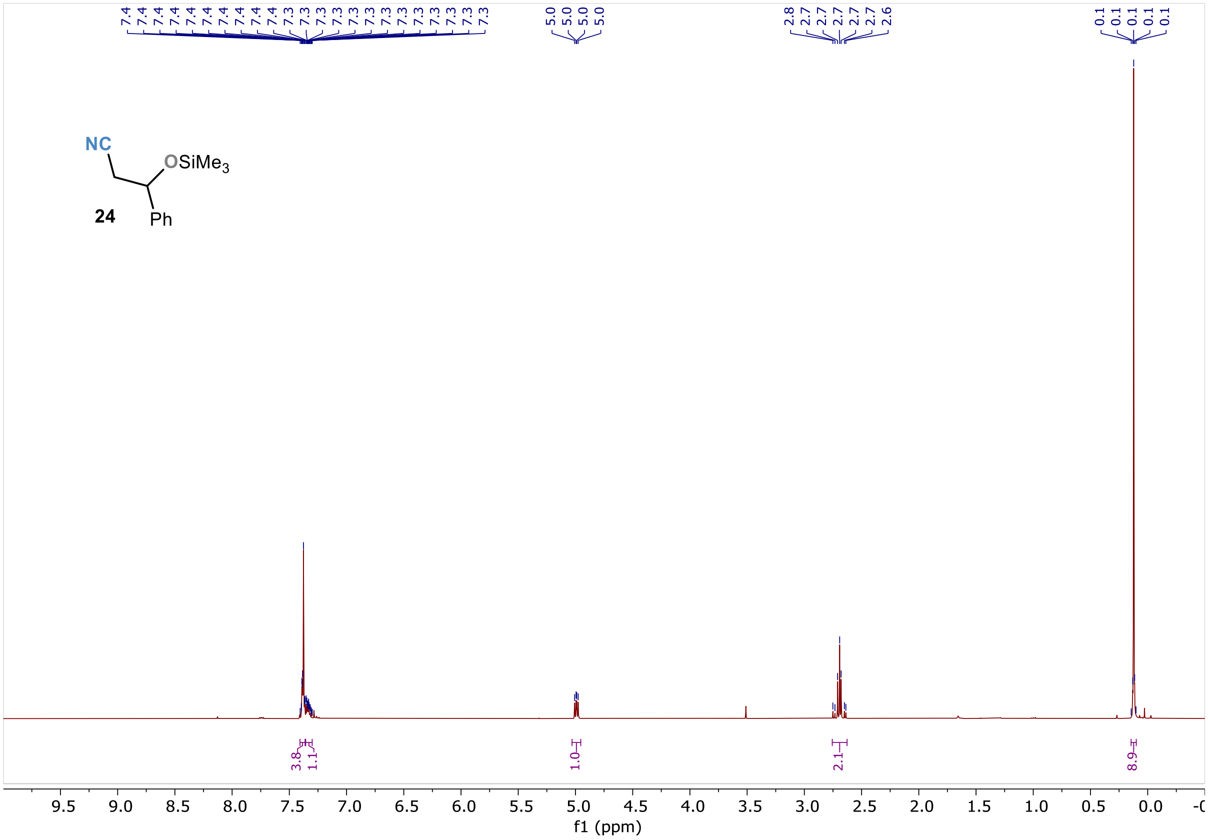

^{13}**C NMR** (101 MHz, $CDCl_3$) for compound **24**:

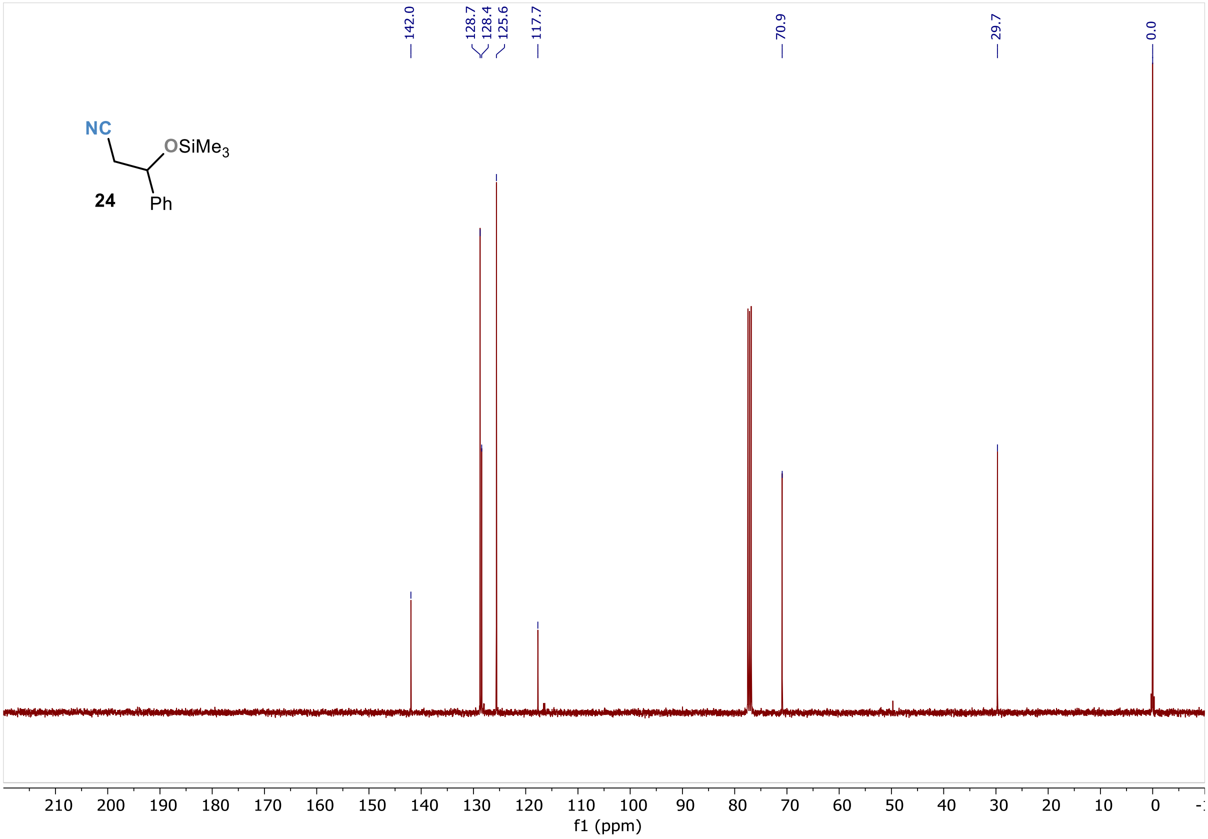

¹H NMR (500 MHz, $CDCl_3$) for compound **9**:

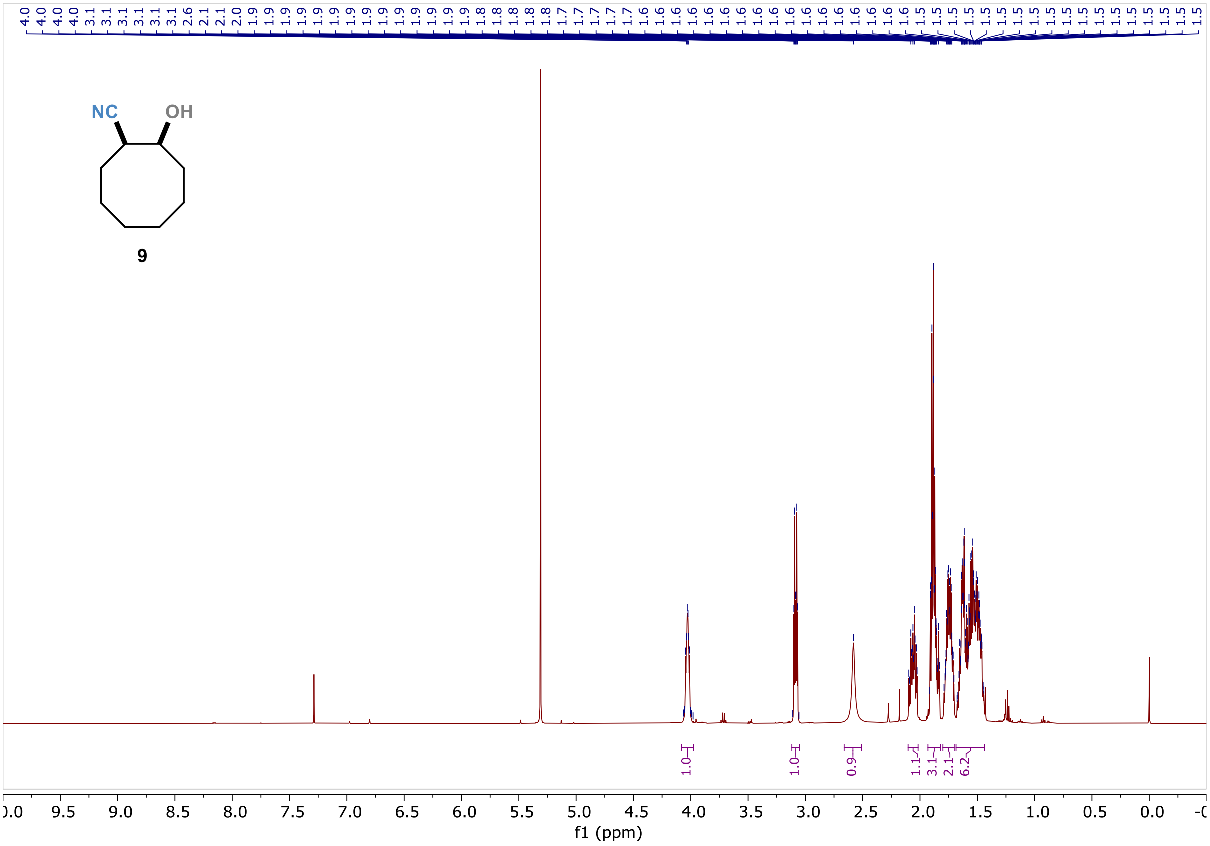

¹³C NMR (126 MHz, $CDCl_3$) for compound **9**:

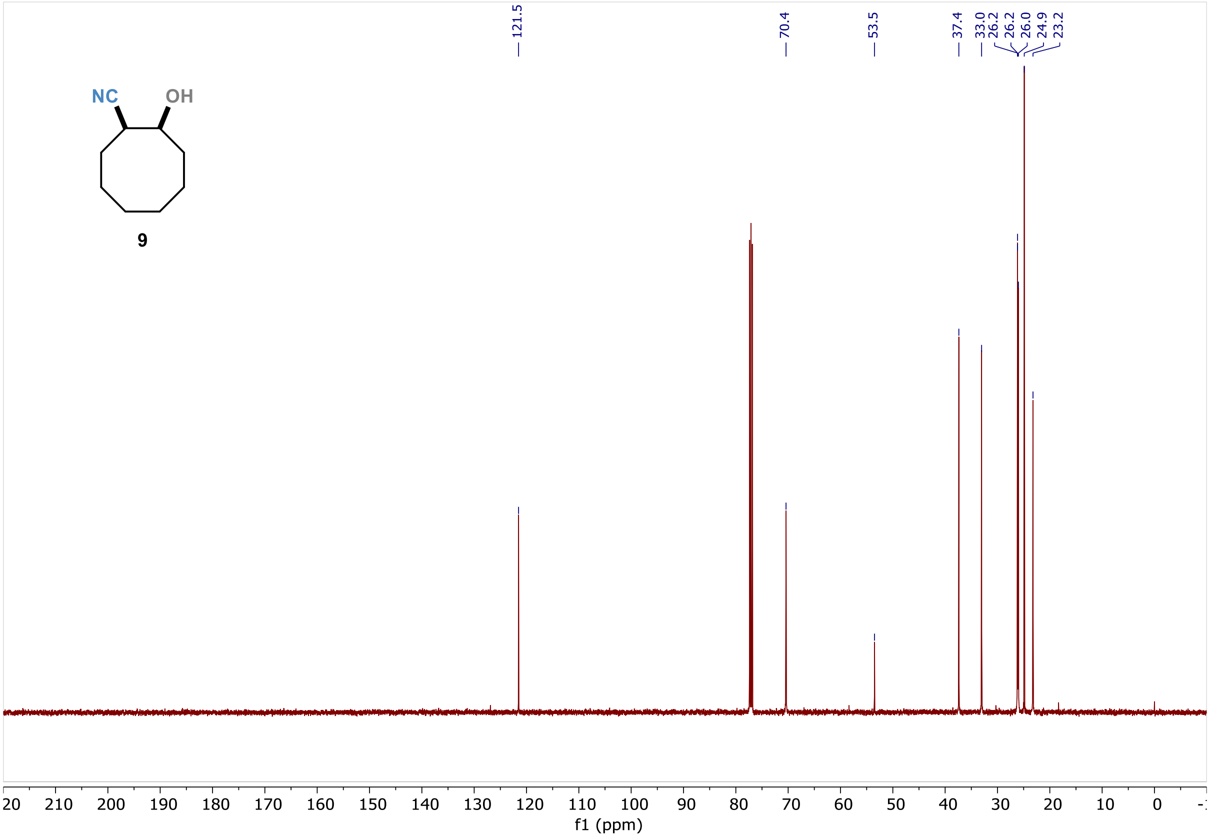

¹H NMR (500 MHz, $CDCl_3$) for compound **27**:

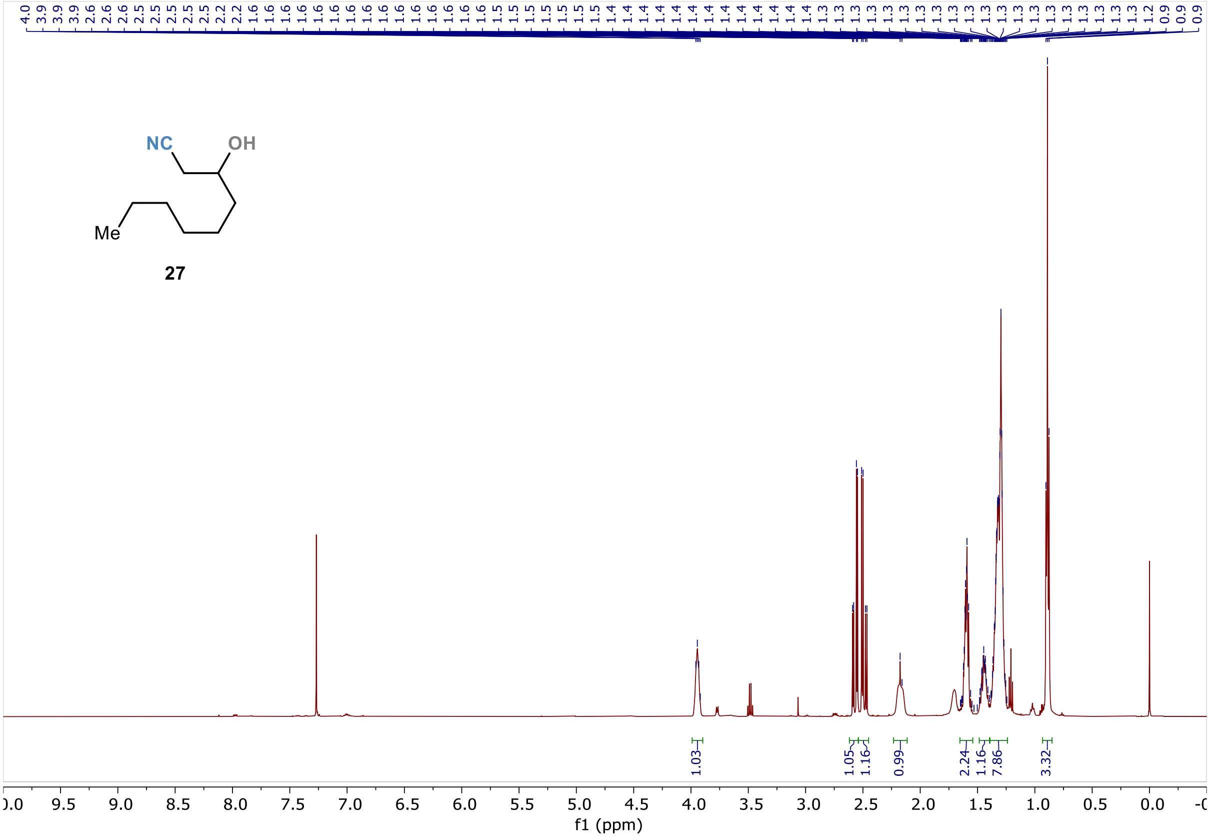

¹³C NMR (126 MHz, $CDCl_3$) for compound **27**:

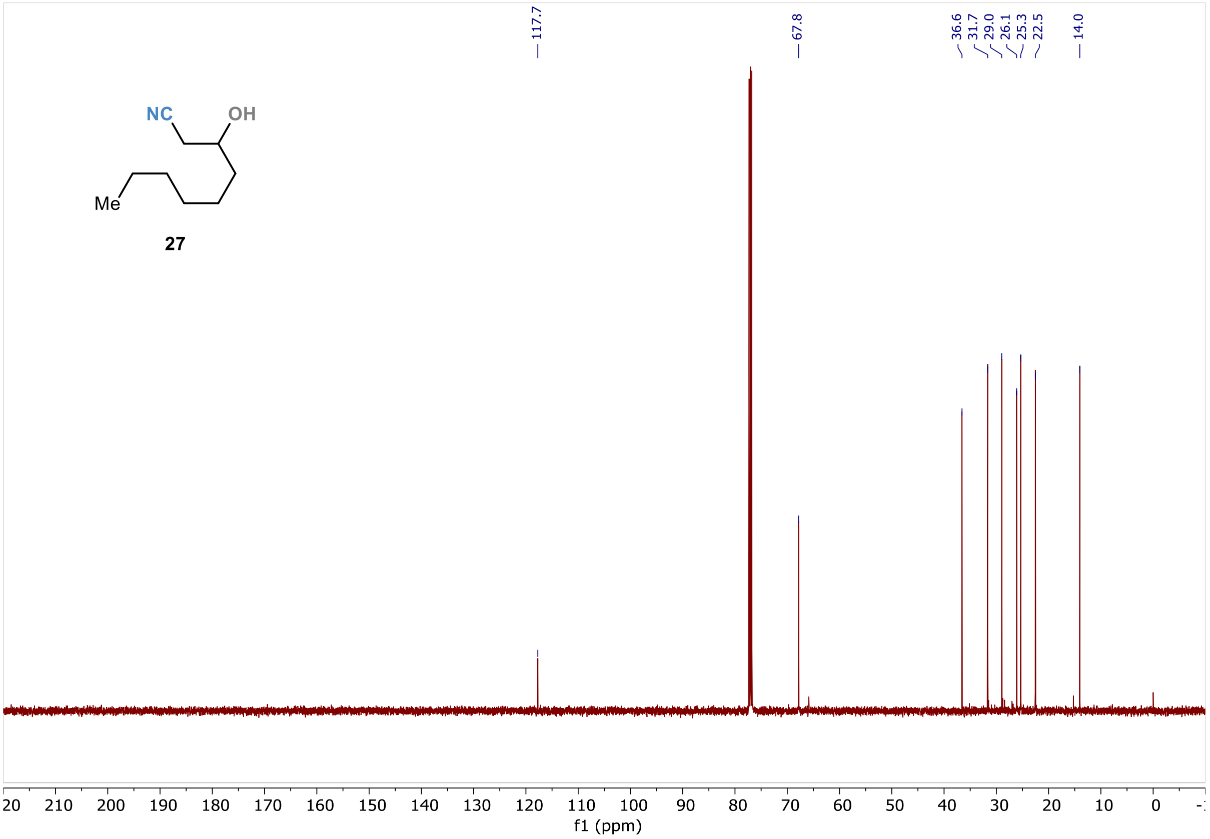

¹H NMR (400 MHz, $CDCl_3$) for compound **28**:

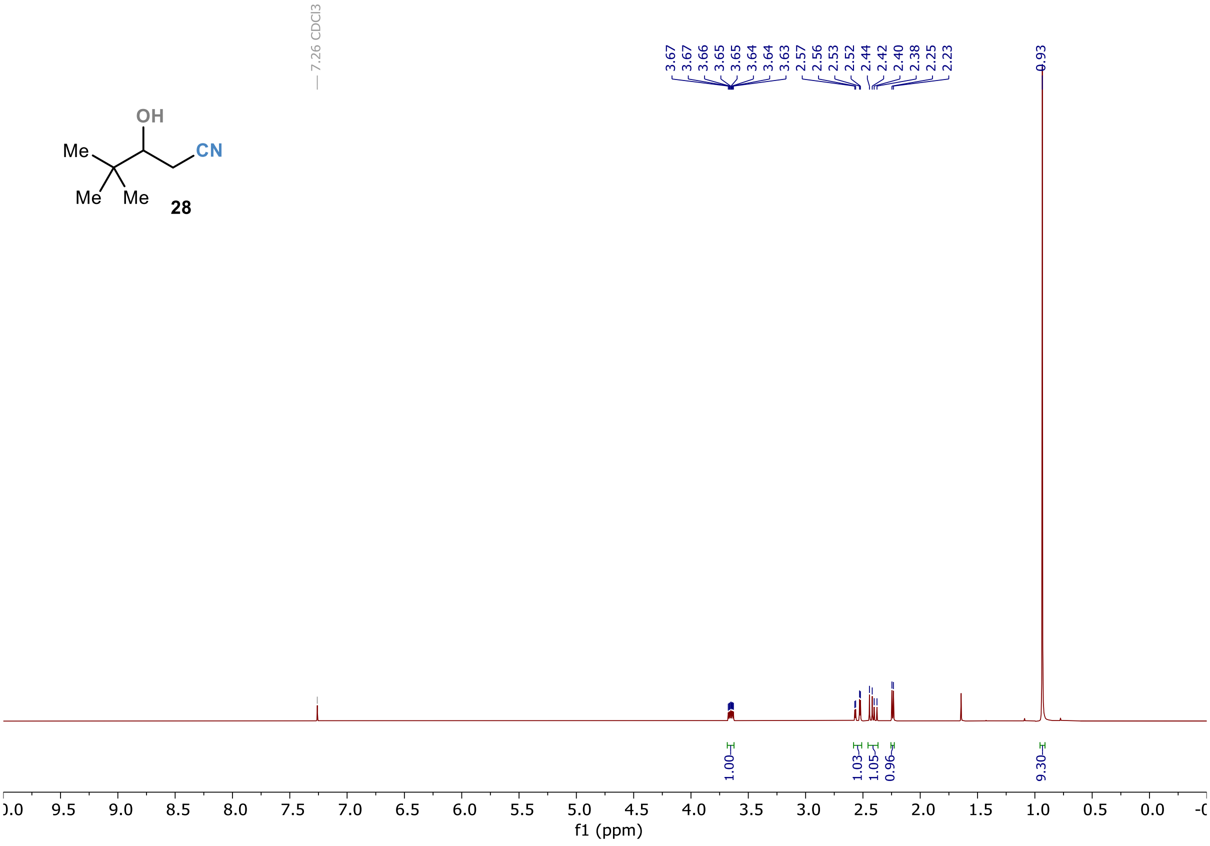

¹³C NMR (101 MHz, $CDCl_3$) for compound **28**:

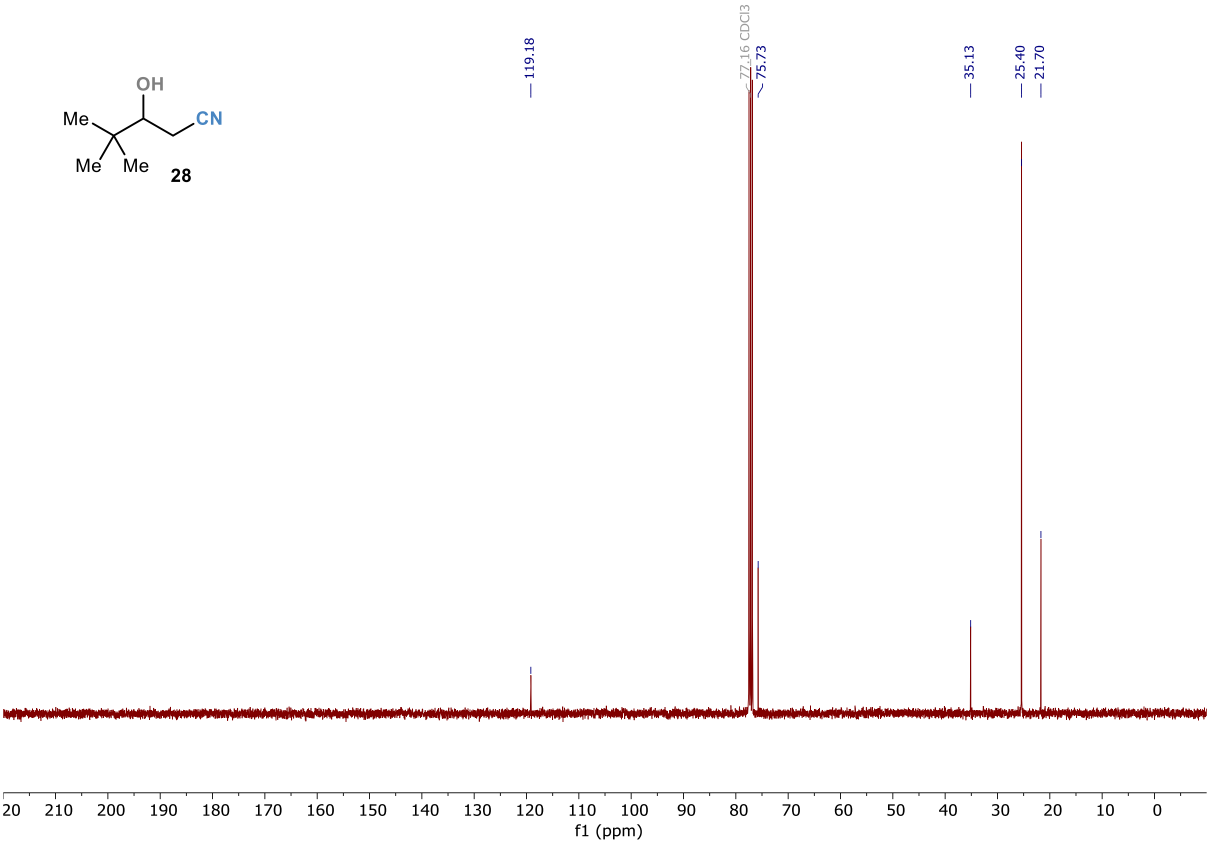

^{1}H NMR (500 MHz, $CDCl_3$) for compound **29**:

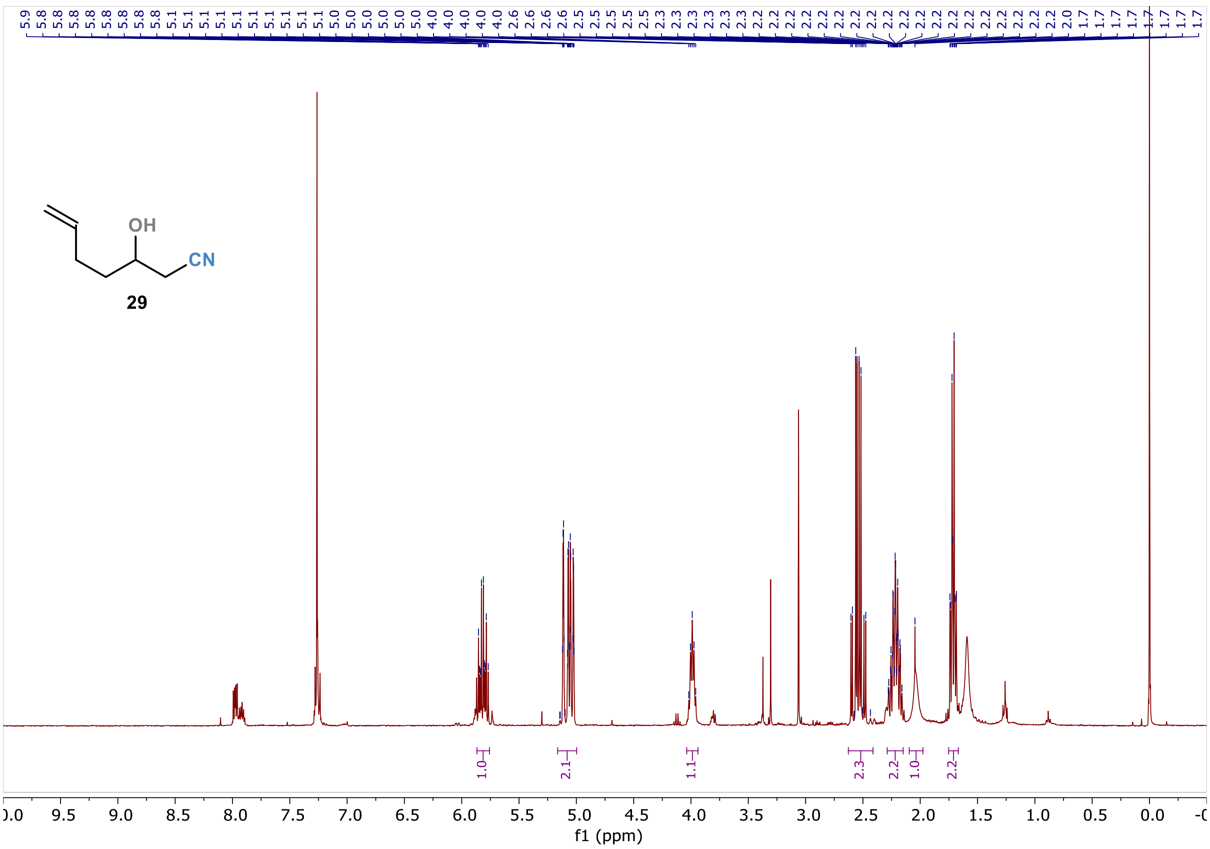

^{13}C NMR (126 MHz, $CDCl_3$) for compound **29**:

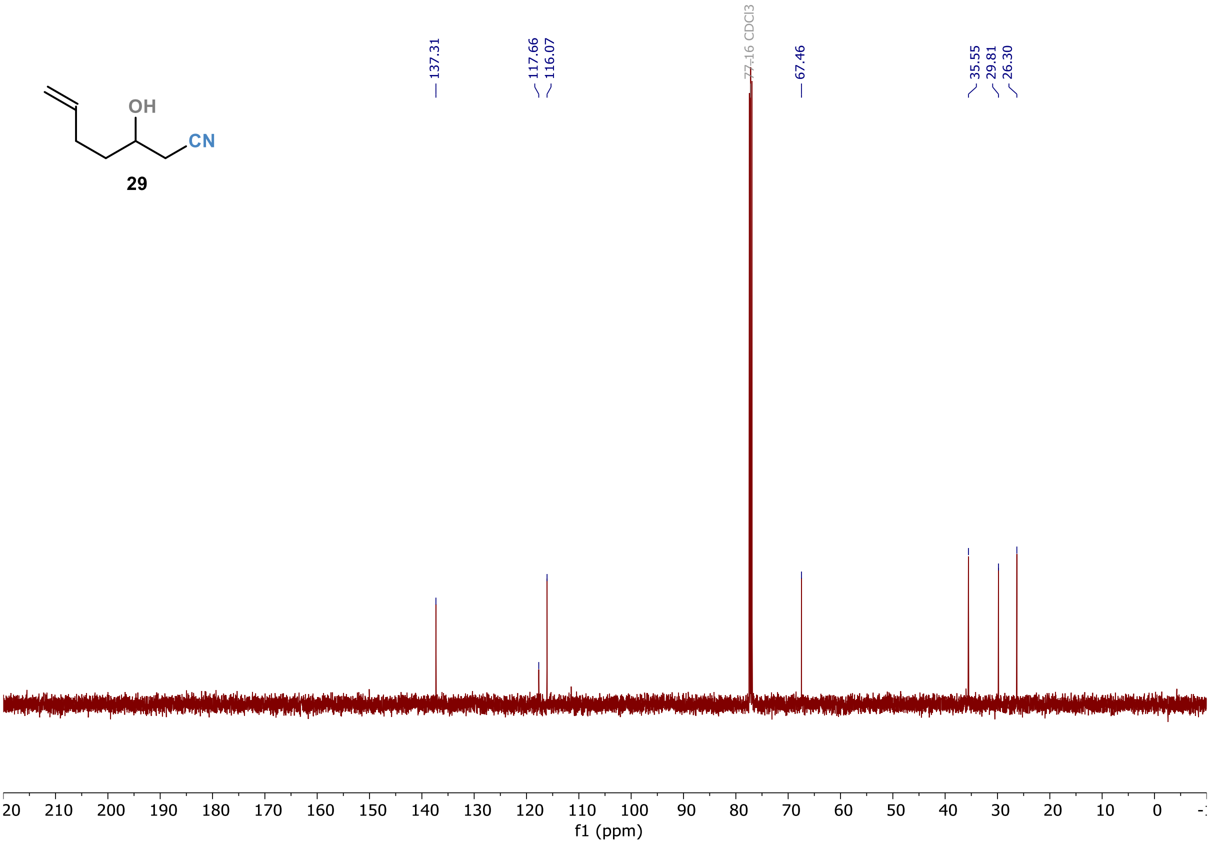

^{1}H NMR (500 MHz, $CDCl_3$) for compound **30**:

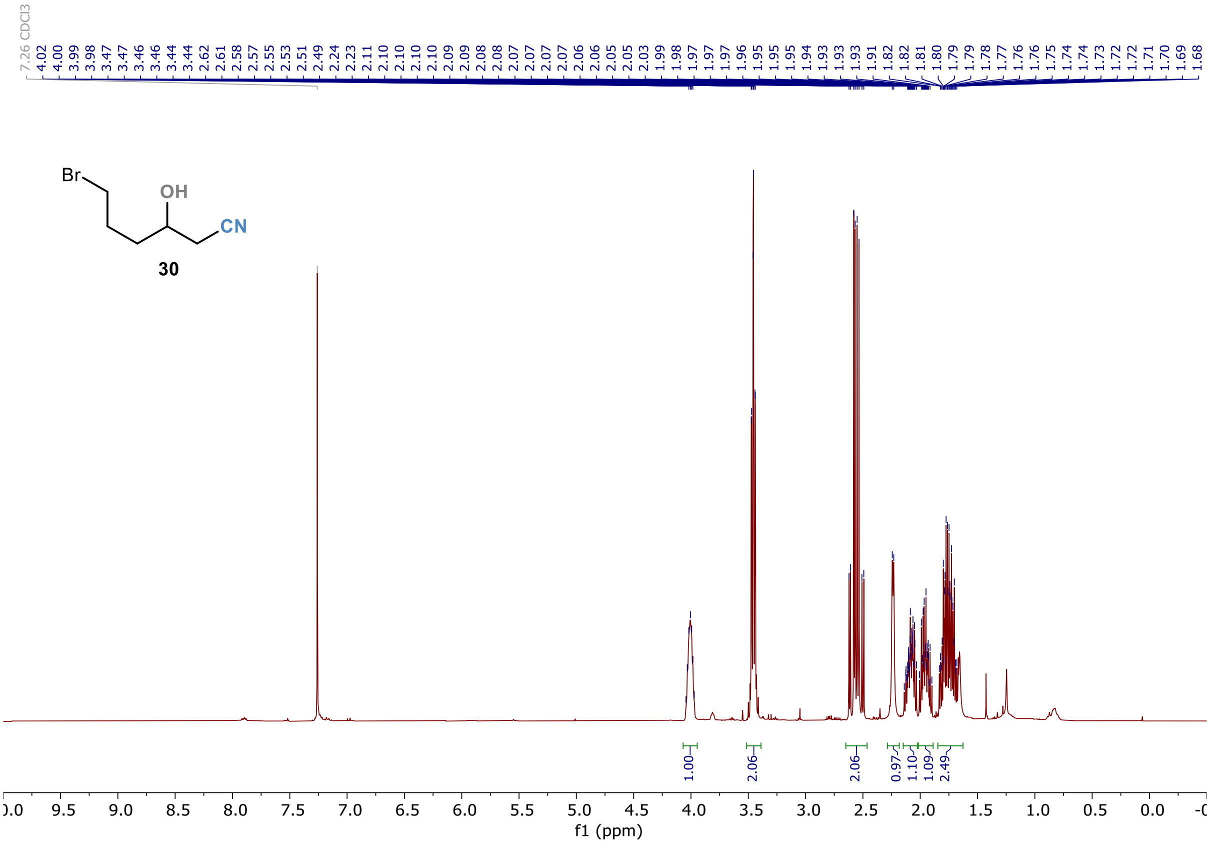

^{13}C NMR (126 MHz, $CDCl_3$) for compound **30**:

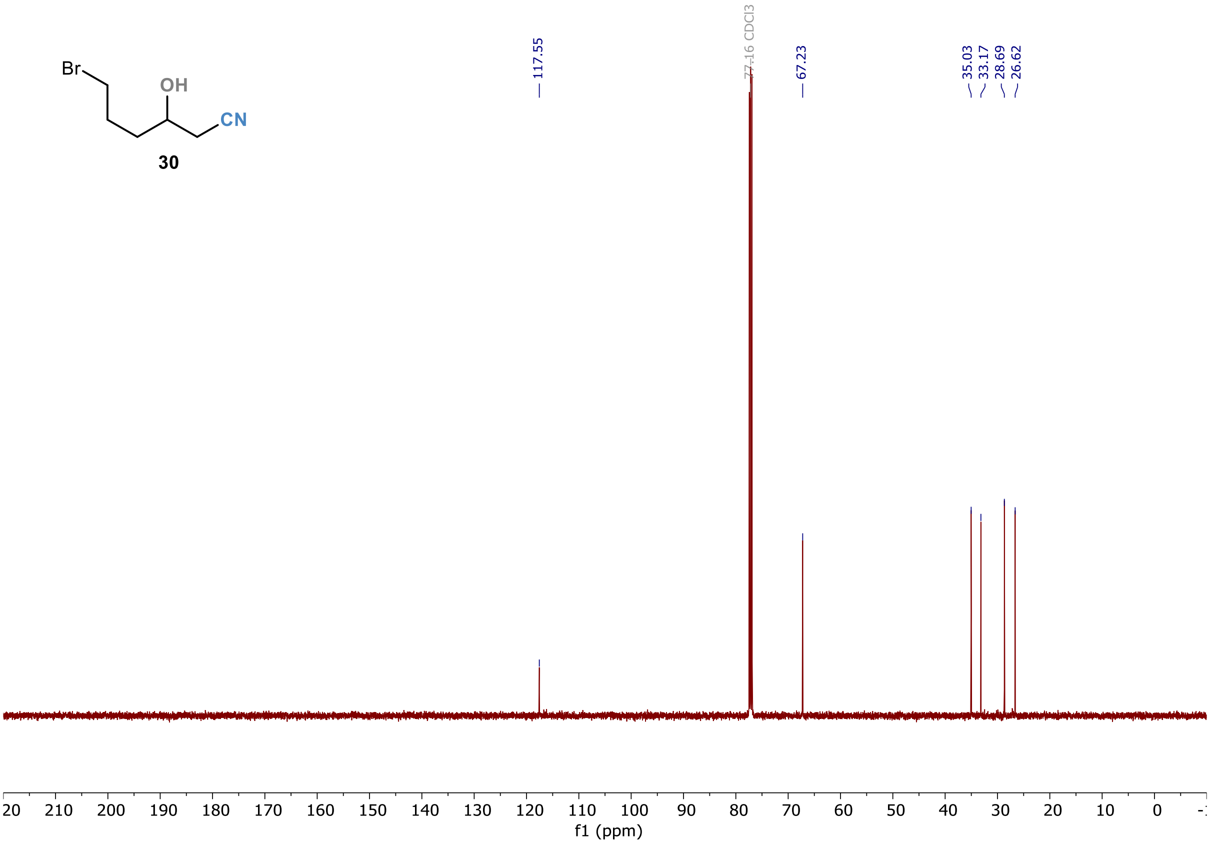

¹H NMR (500 MHz, $CDCl_3$) for compound **31-*major***:

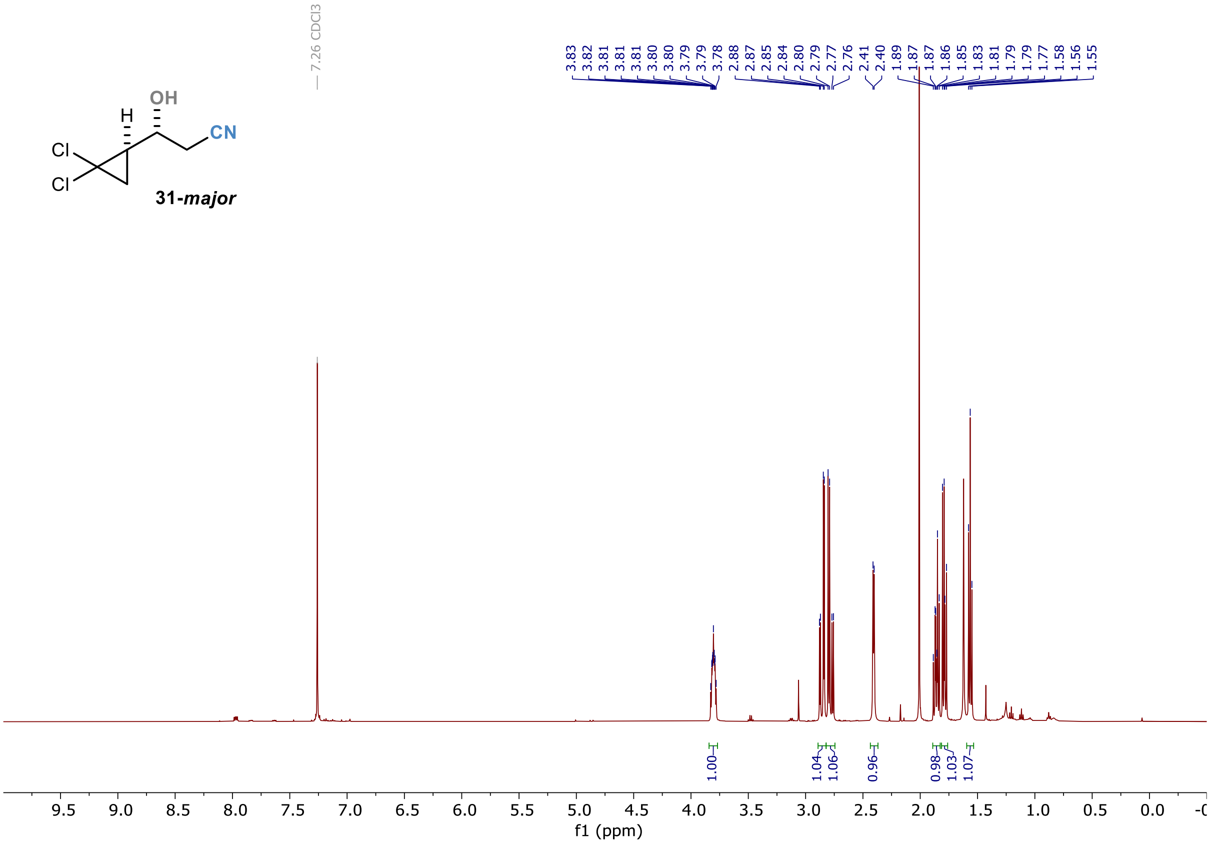

¹³C NMR (126 MHz, $CDCl_3$) for compound **31-*major***:

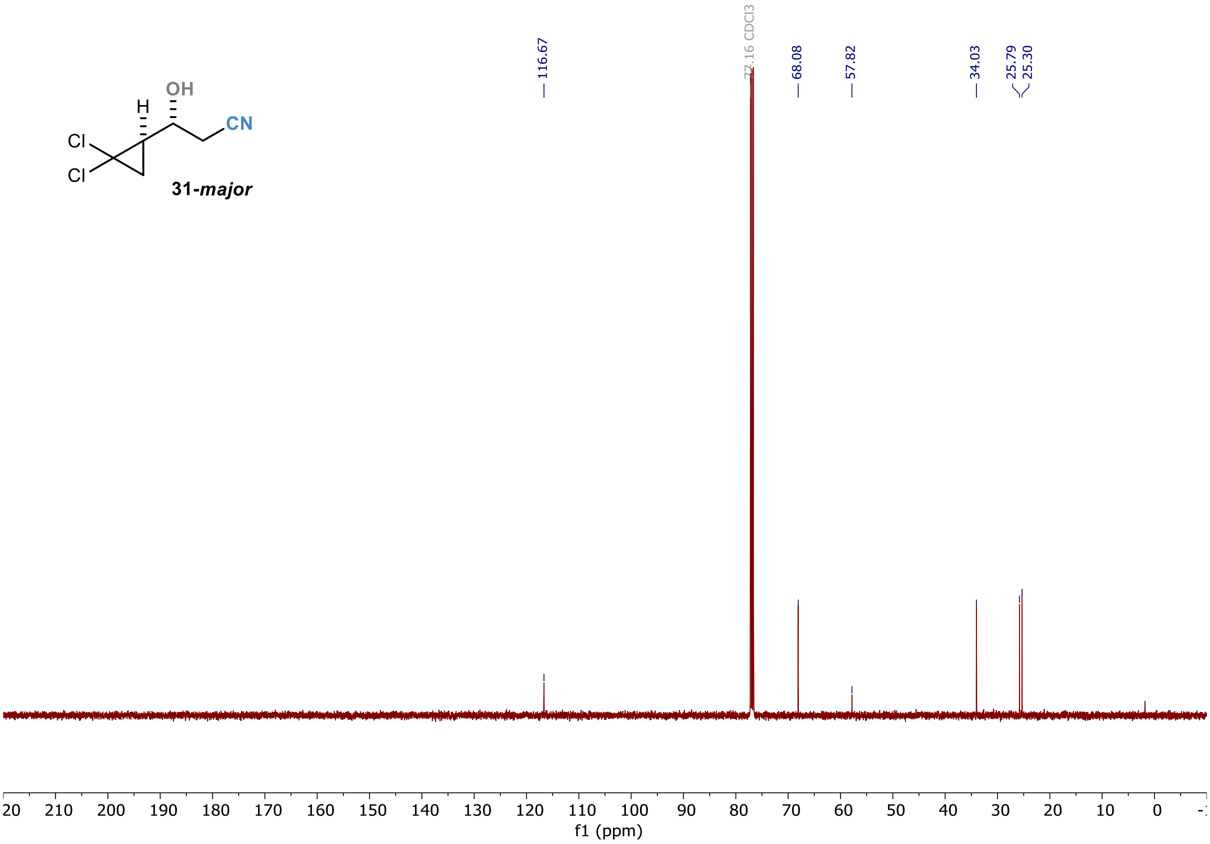

¹H NMR (400 MHz, $CDCl_3$) for compound **31-*minor***:

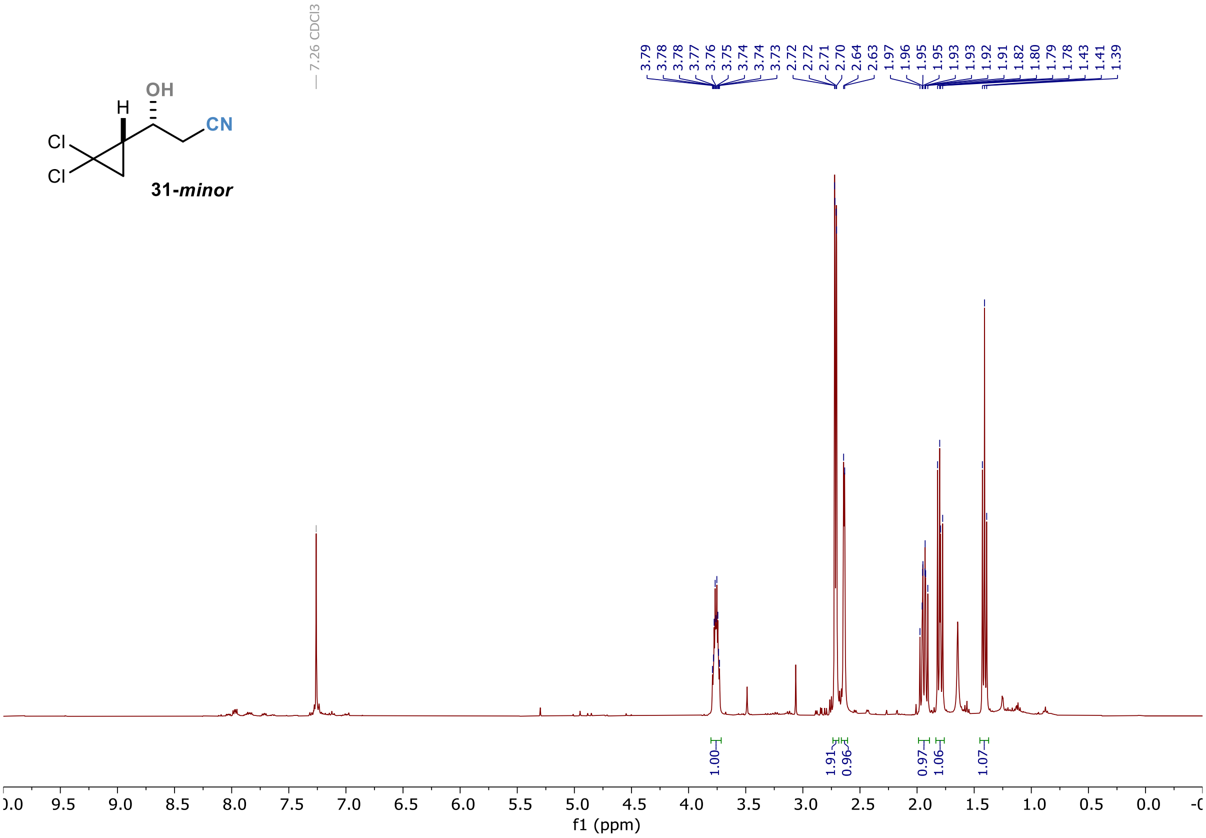

¹³C NMR (101 MHz, $CDCl_3$) for compound **31-*minor***:

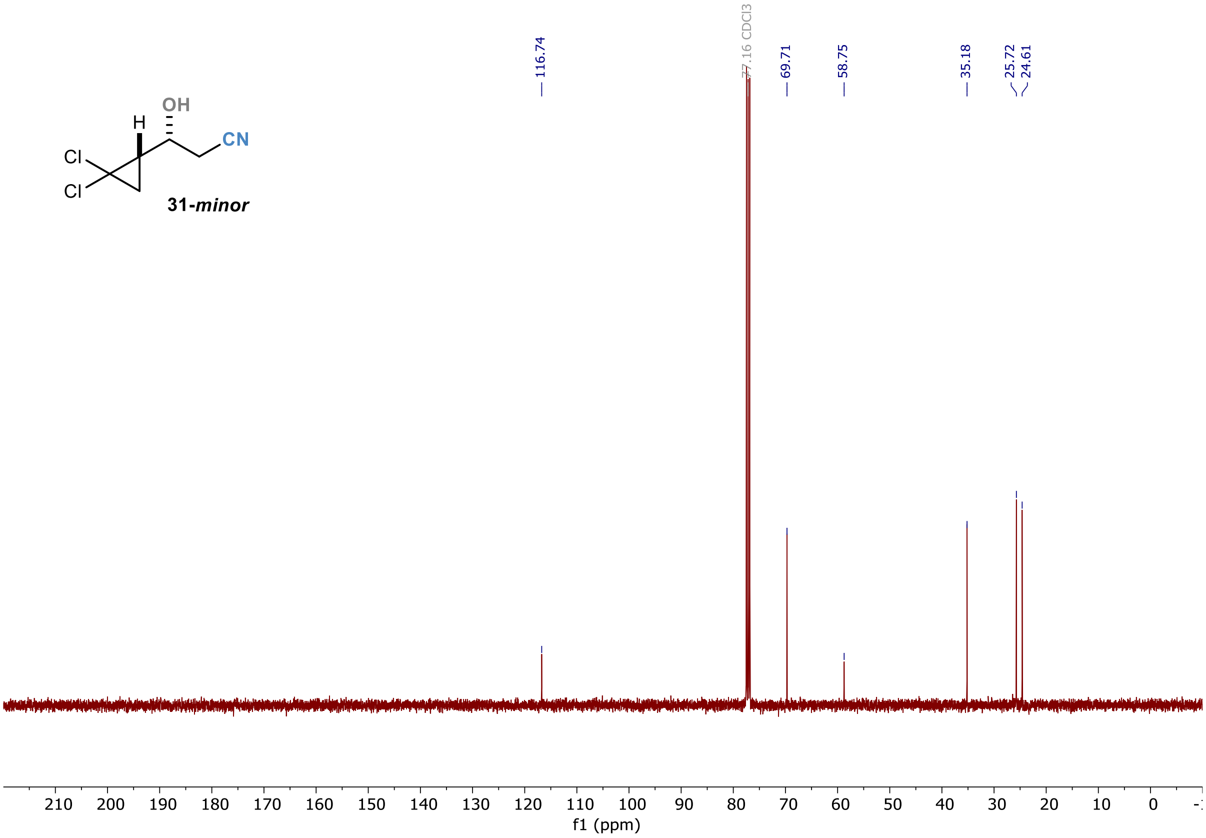

¹H NMR (400 MHz, $CDCl_3$) for compound **32**:

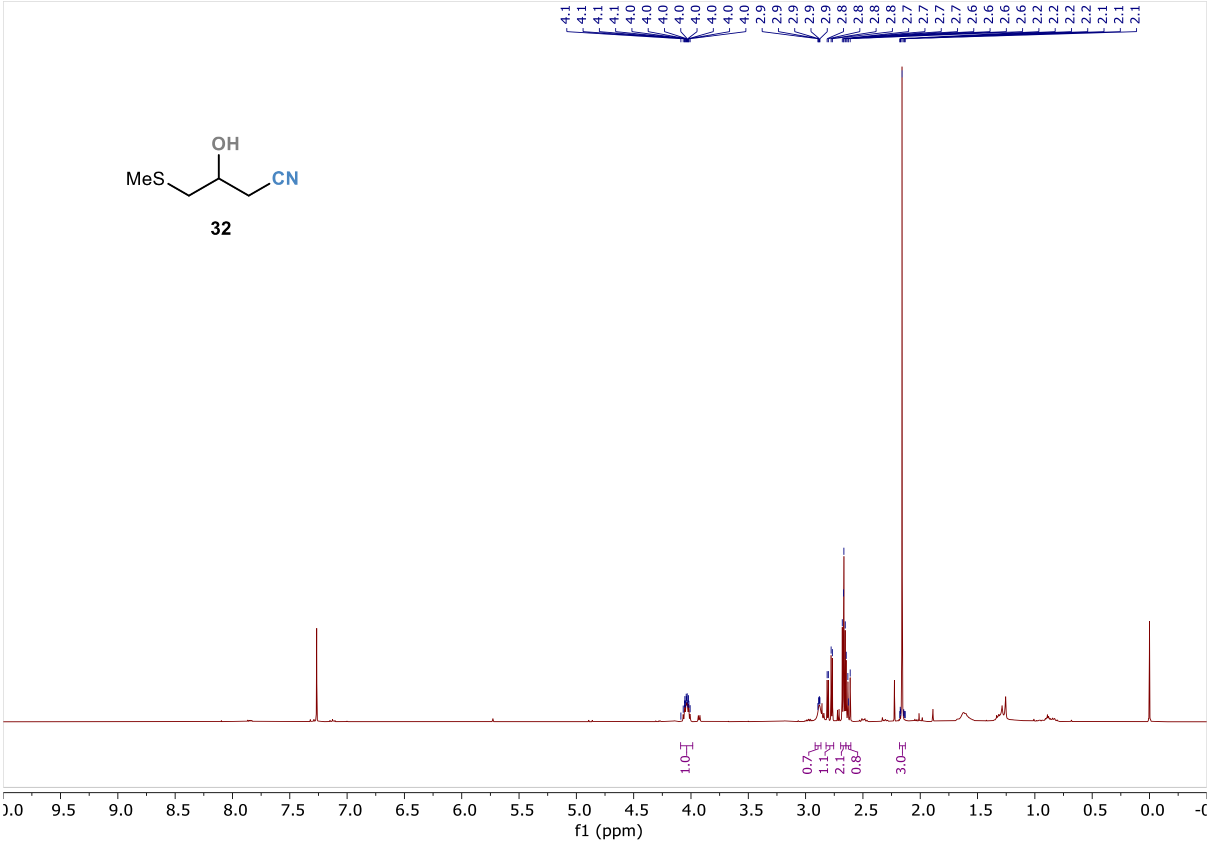

¹³C NMR (101 MHz, $CDCl_3$) for compound **32**:

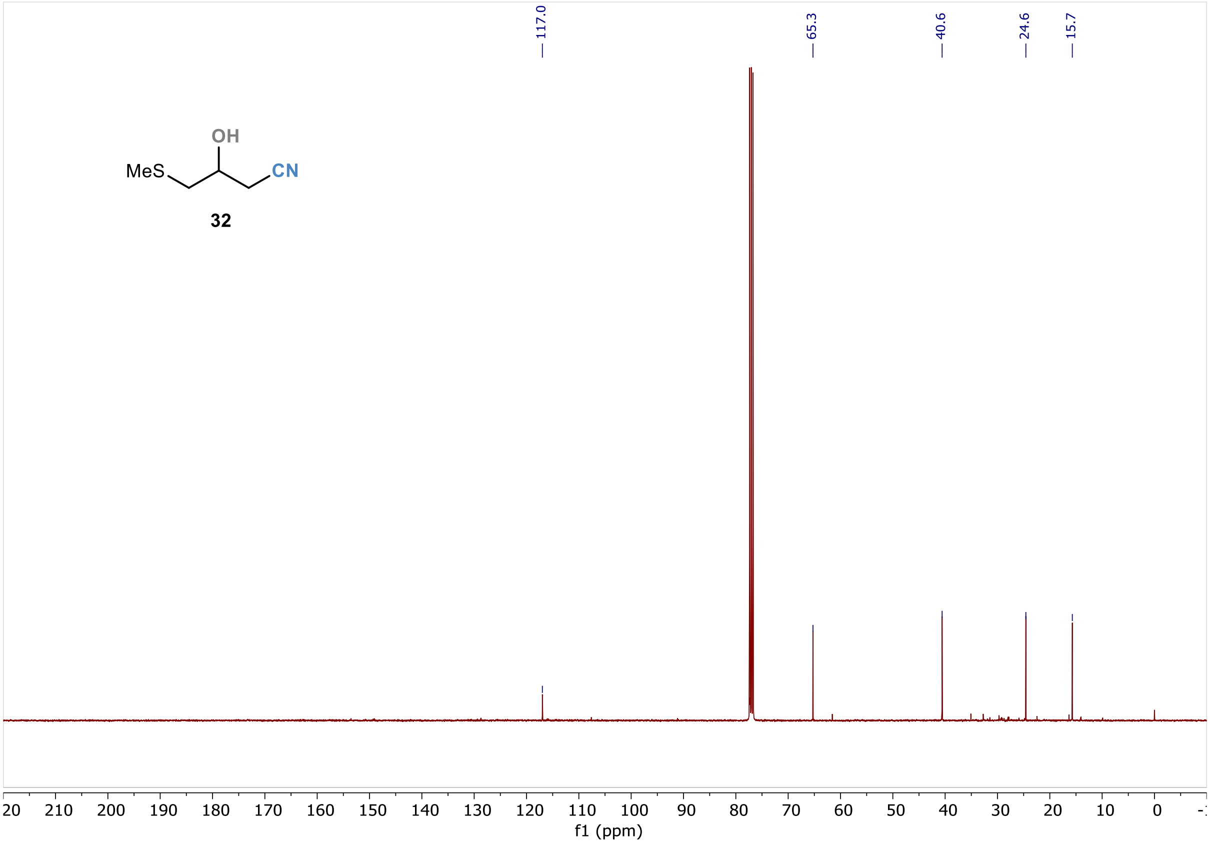

^{1}H NMR (400 MHz, $CDCl_3$) for compound **33**:

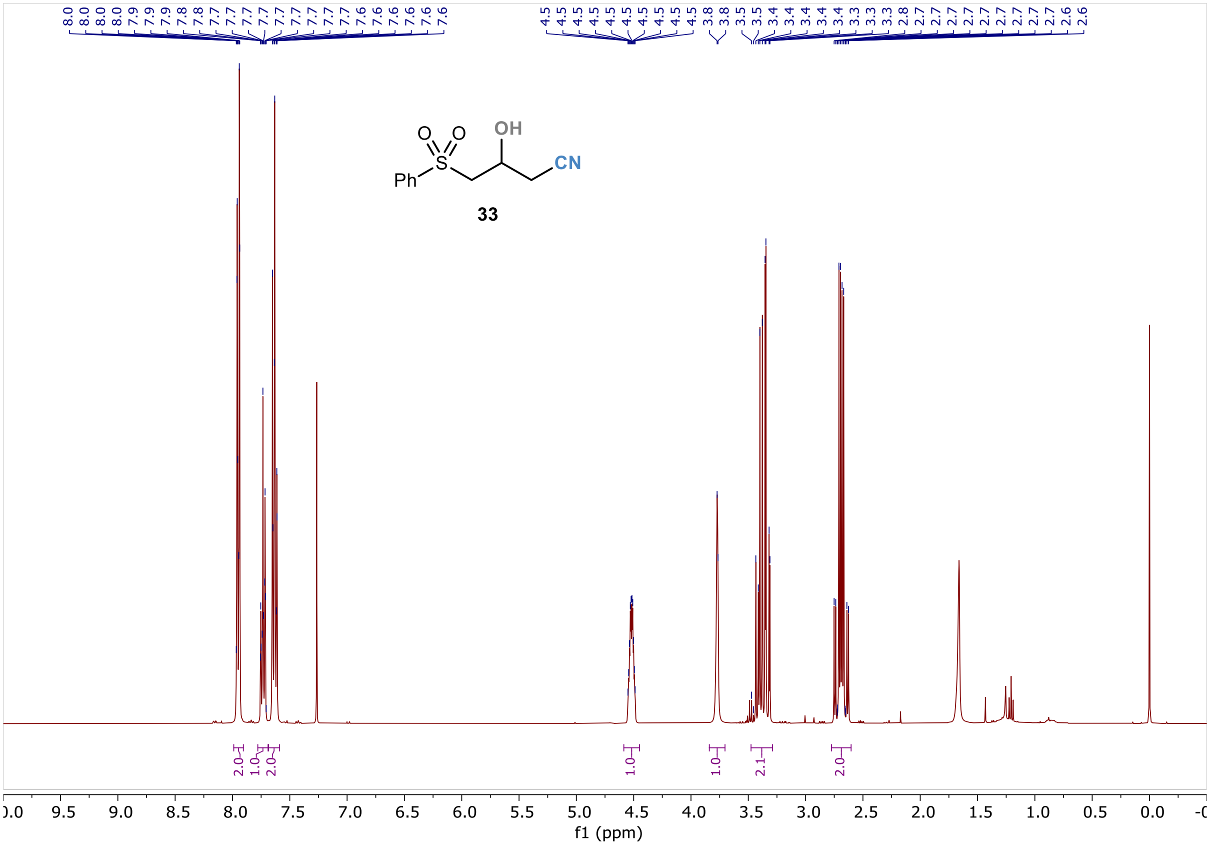

^{13}C NMR (101 MHz, $CDCl_3$) for compound **33**:

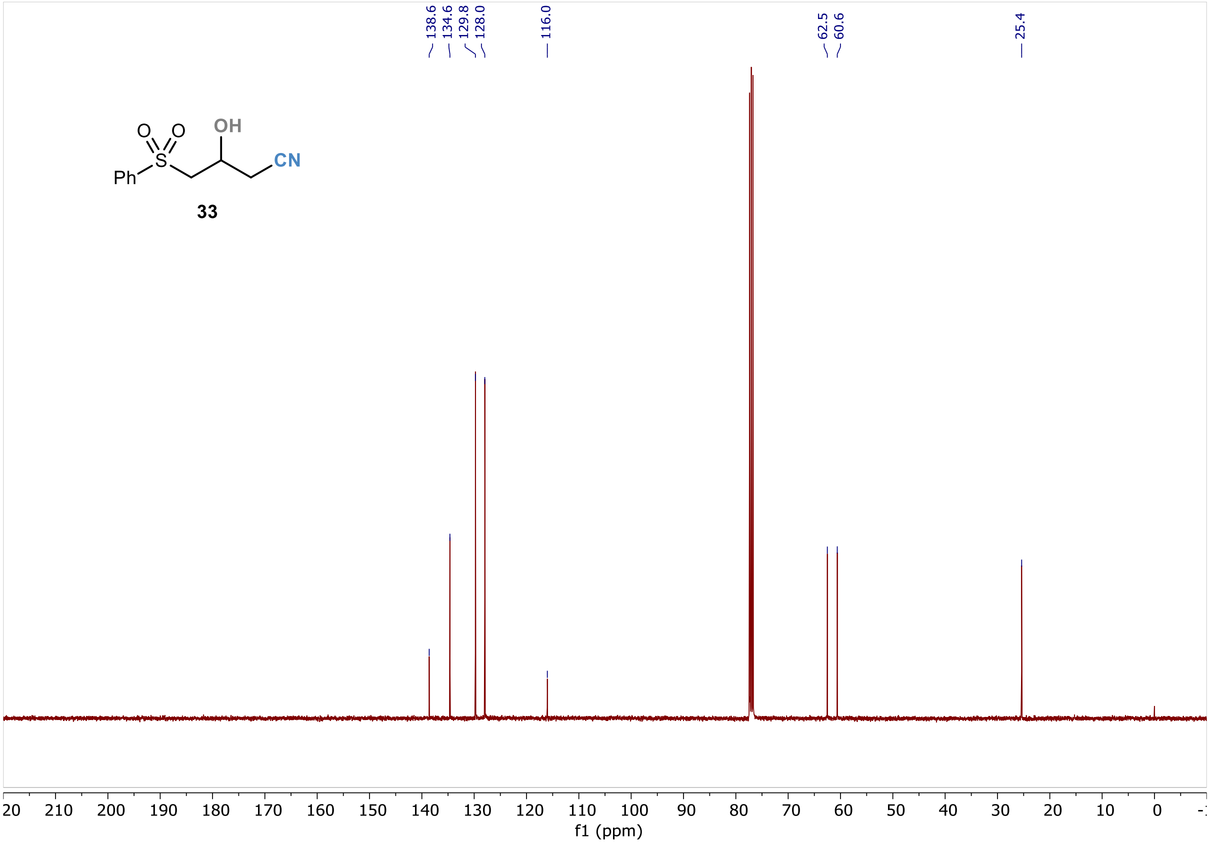

^{1}H NMR (400 MHz, $CDCl_3$) for compound **34**:

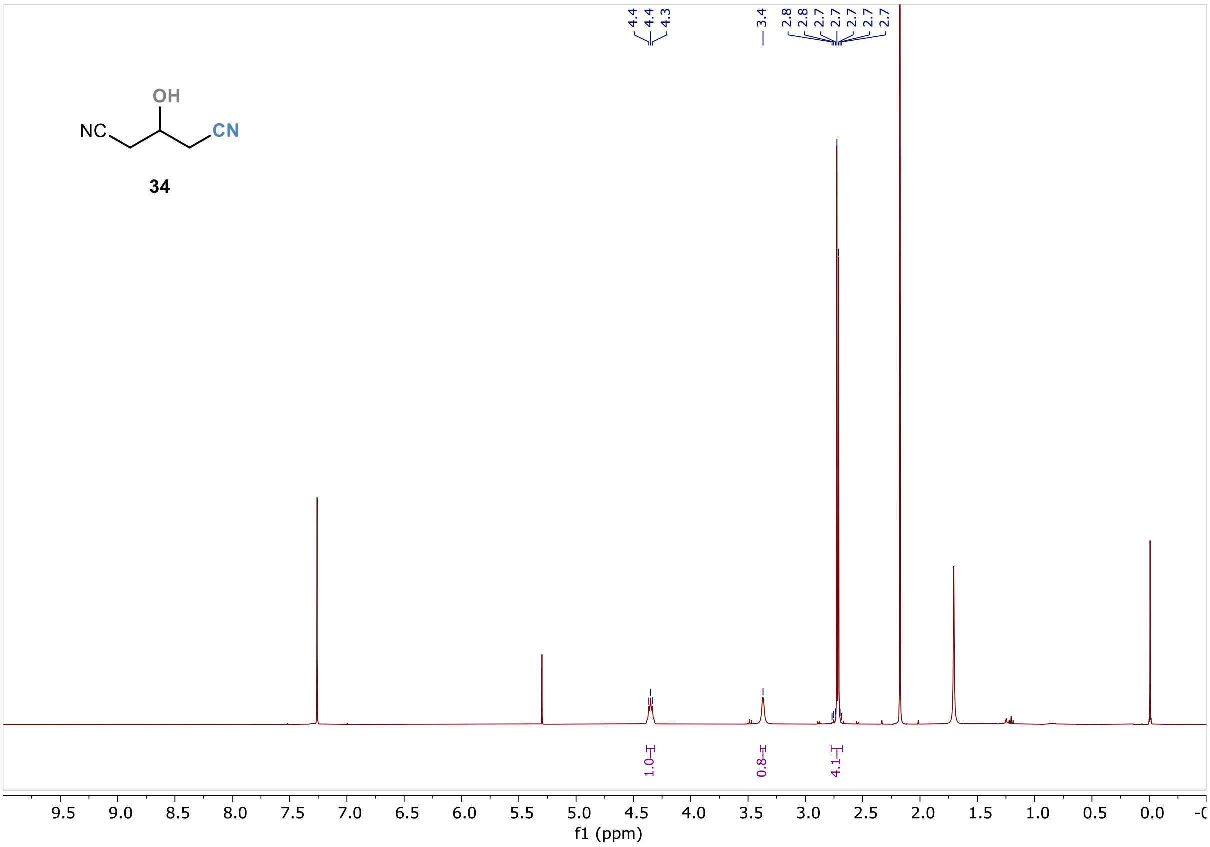

^{13}C NMR (101 MHz, $CDCl_3$) for compound **34**:

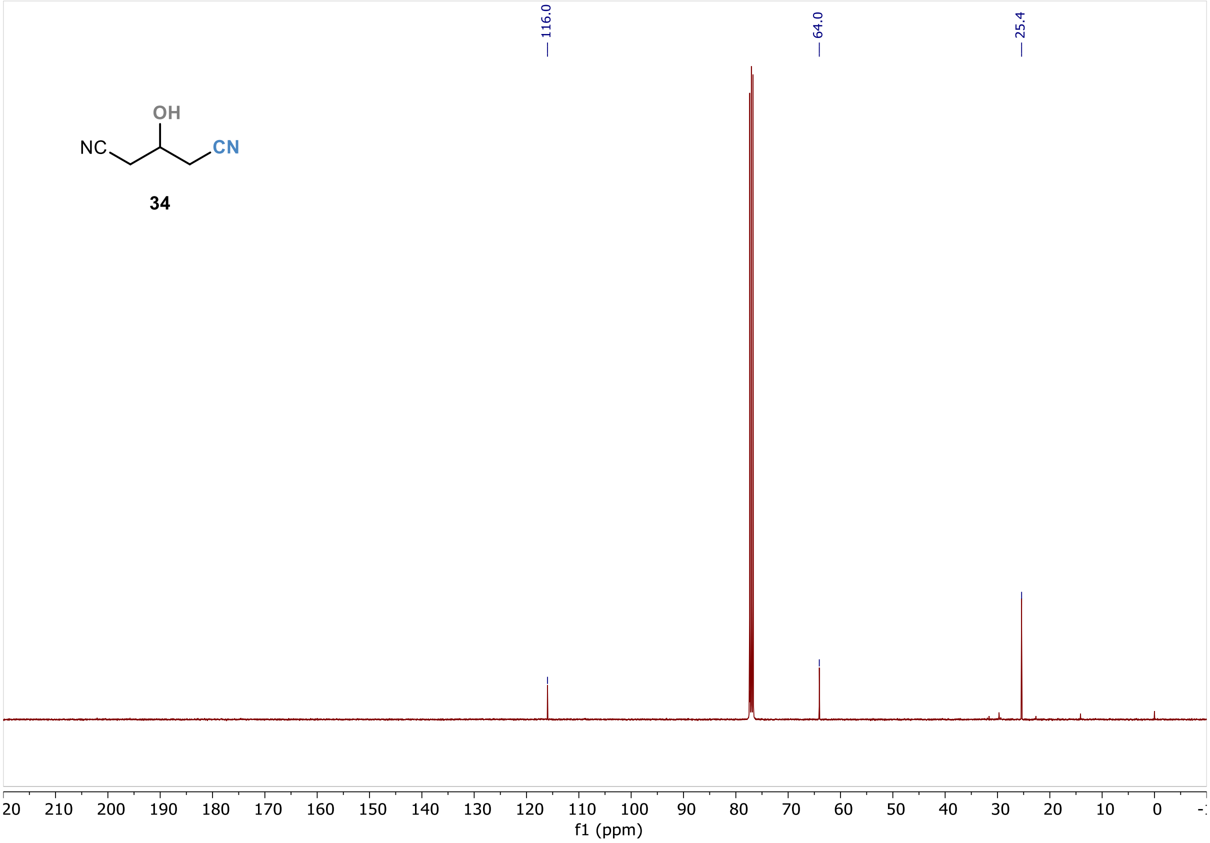

^{1}H NMR (400 MHz, $CDCl_3$) for compound **35**:

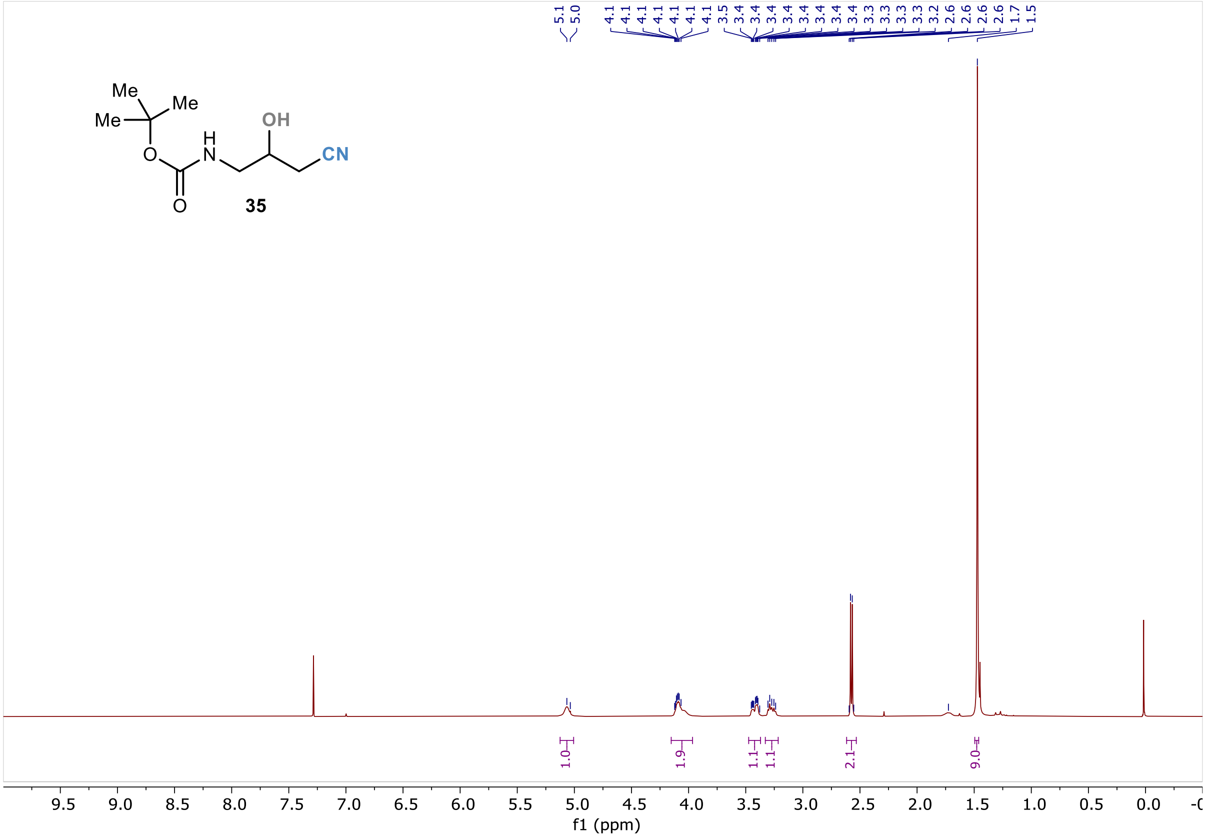

^{13}C NMR (101 MHz, $CDCl_3$) for compound **35**:

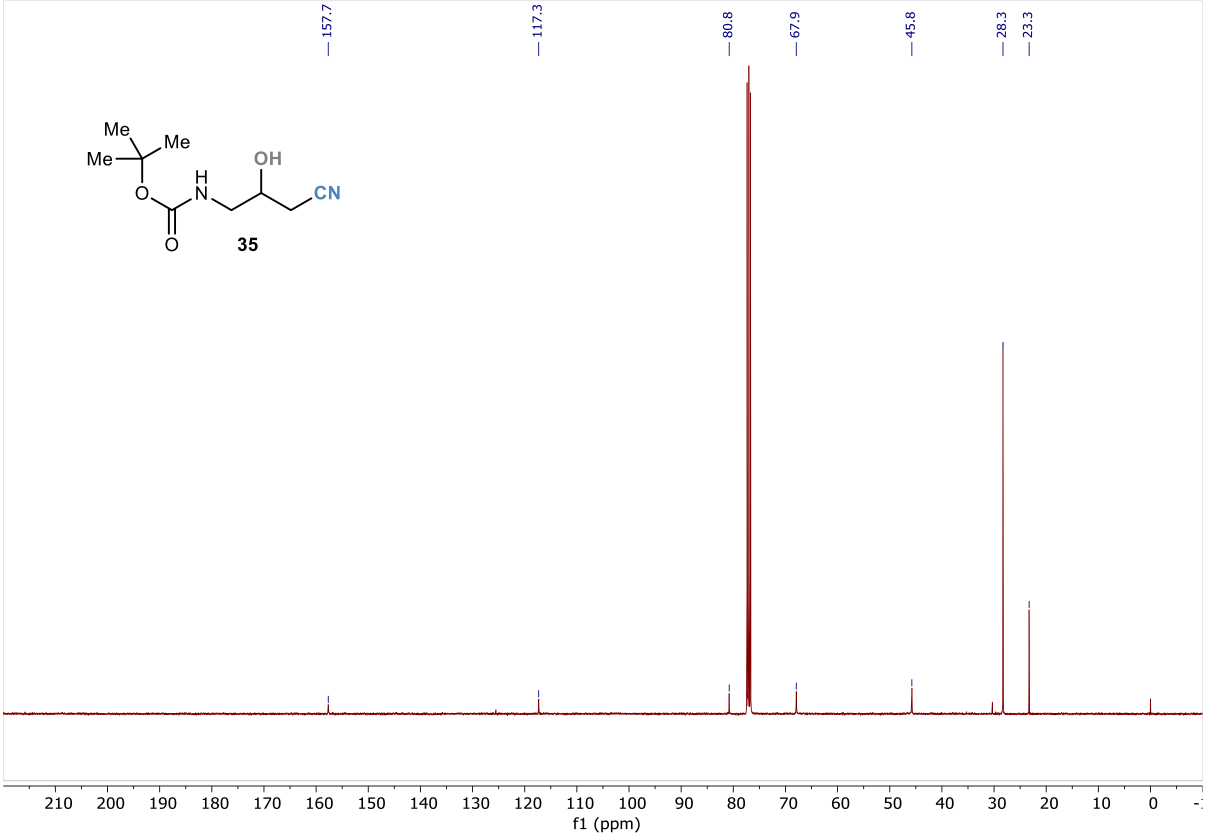

^{1}**H NMR** (400 MHz, $CDCl_3$) for compound **36**:

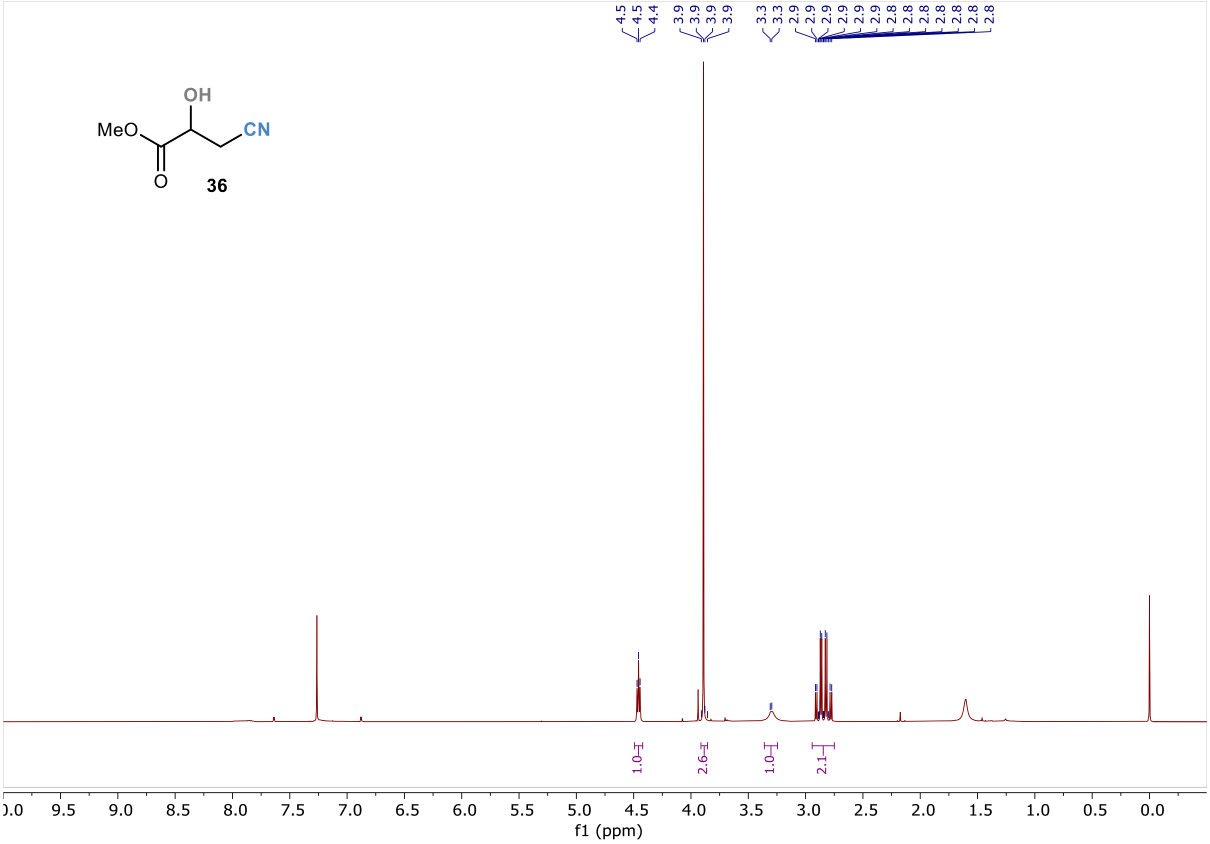

^{13}**C NMR** (101 MHz, $CDCl_3$) for compound **36**:

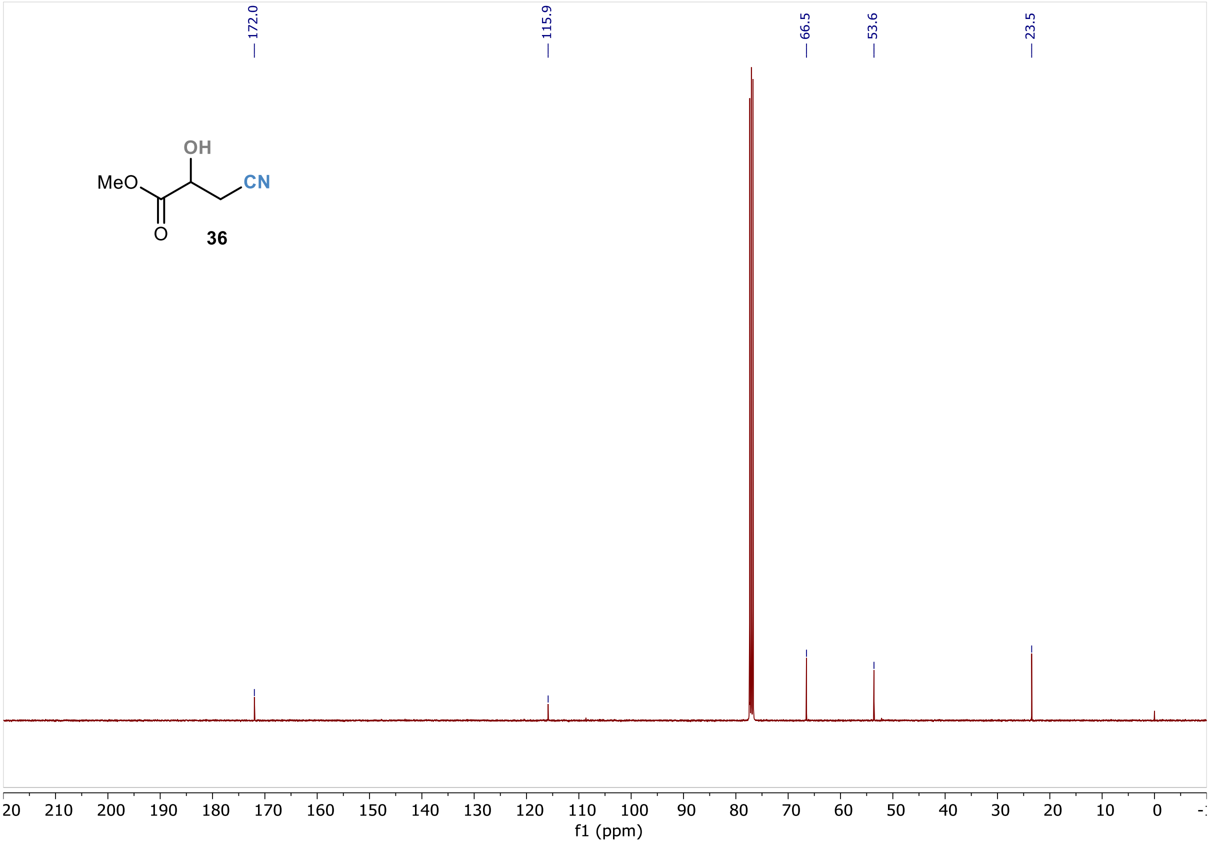

^{1}**H NMR** (400 MHz, $CDCl_3$) for compound **37**:

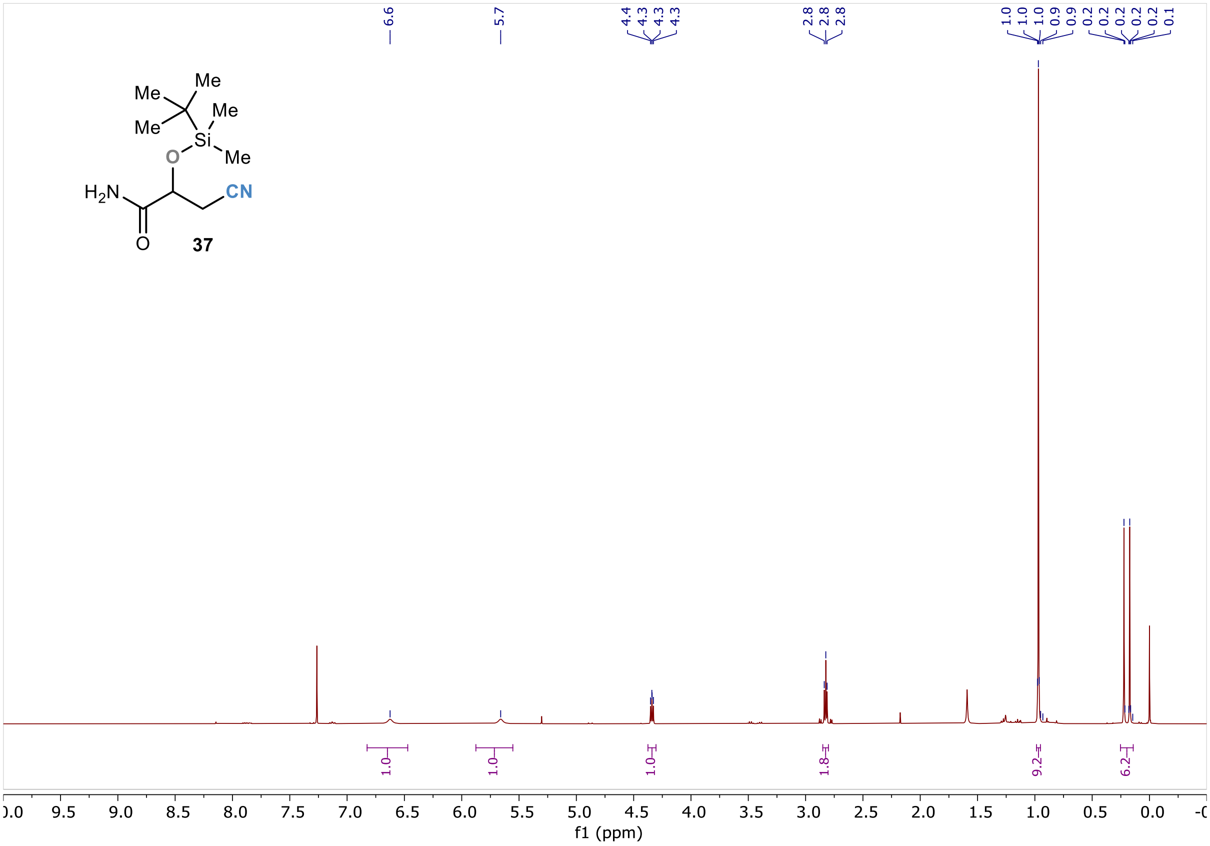

^{13}**C NMR** (101 MHz, $CDCl_3$) for compound **37**:

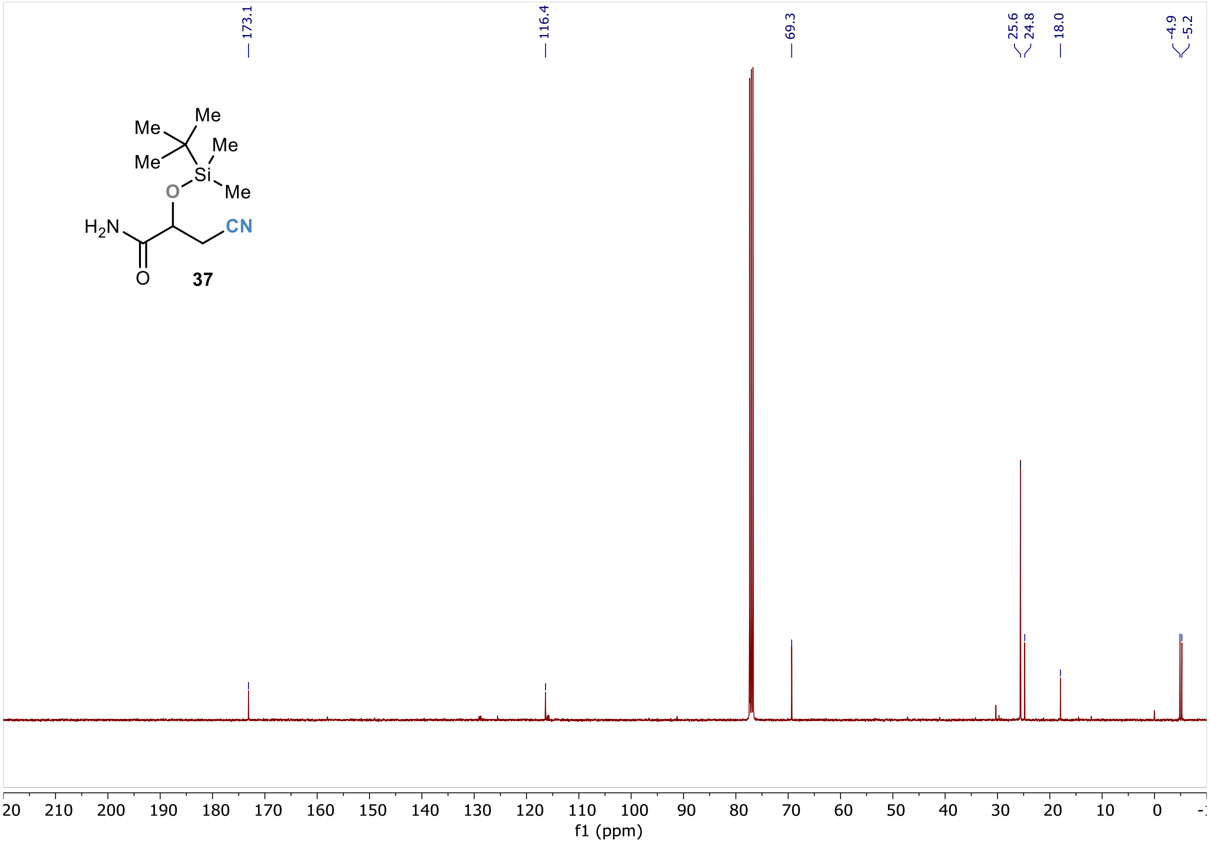

[1]**H NMR** (400 MHz, $CDCl_3$) for compound **38**:

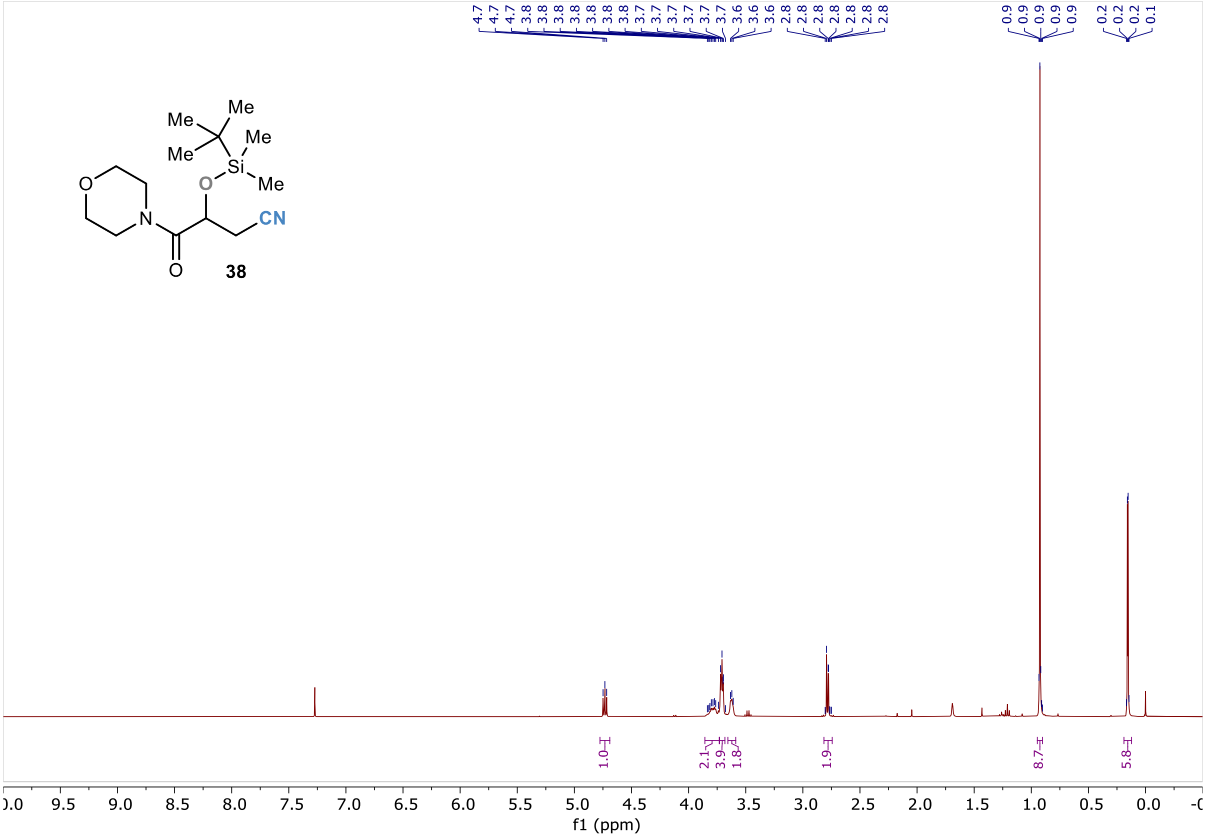

[13]**C NMR** (101 MHz, $CDCl_3$) for compound **38**:

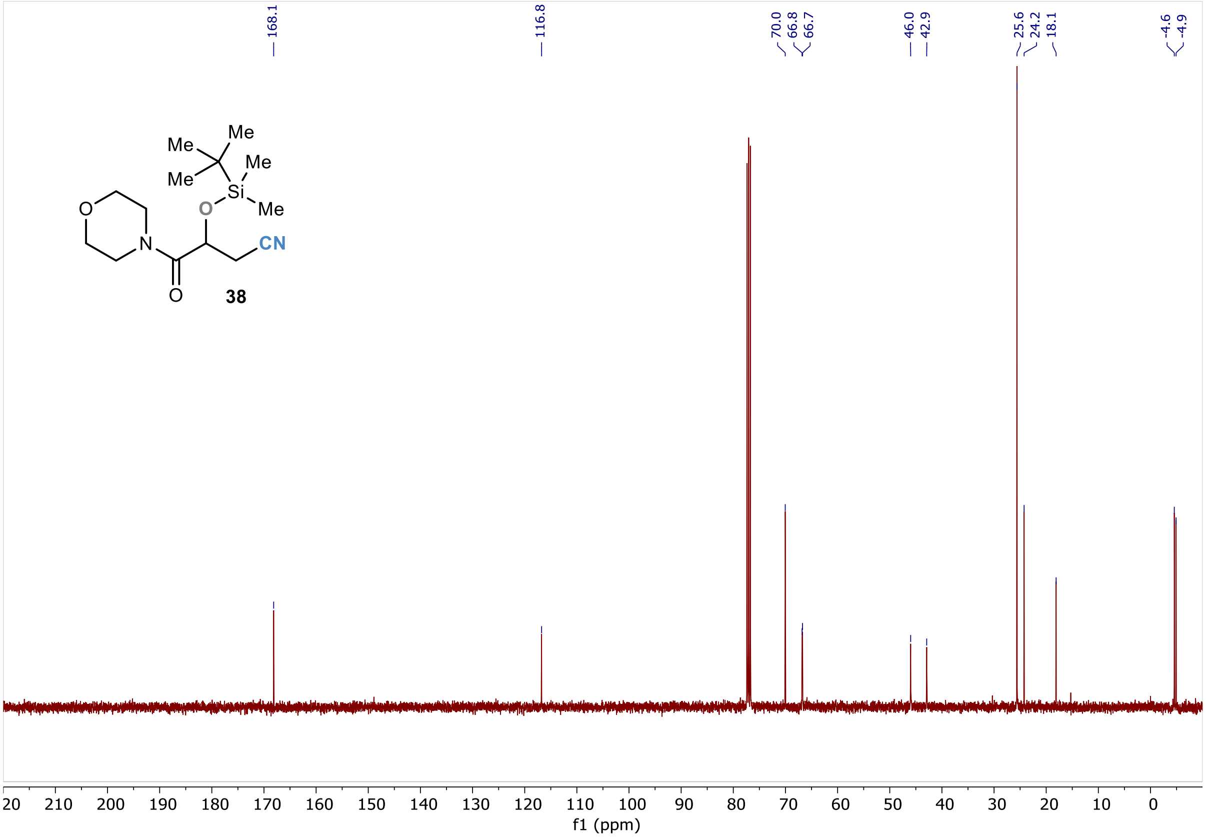

^{1}H NMR (400 MHz, $CDCl_3$) for compound **39**:

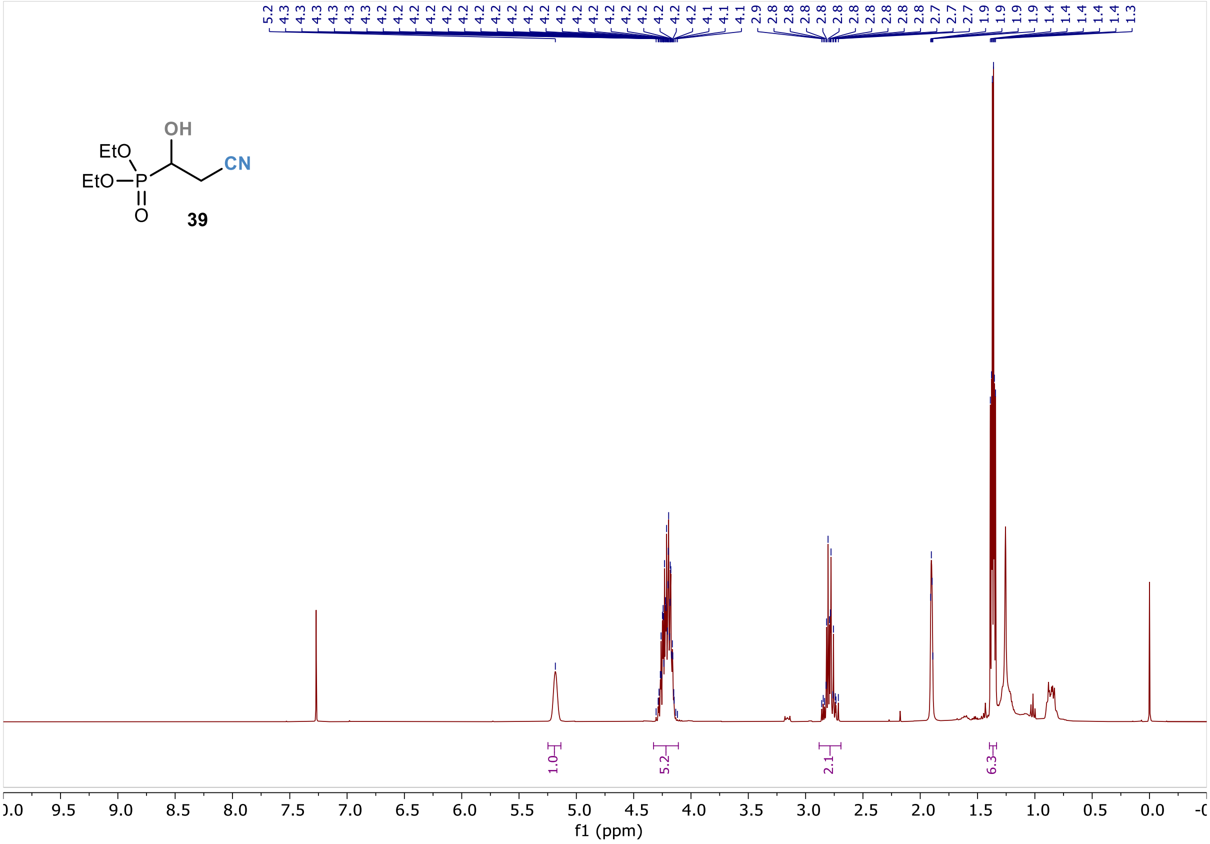

^{13}C NMR (101 MHz, $CDCl_3$) for compound **39**:

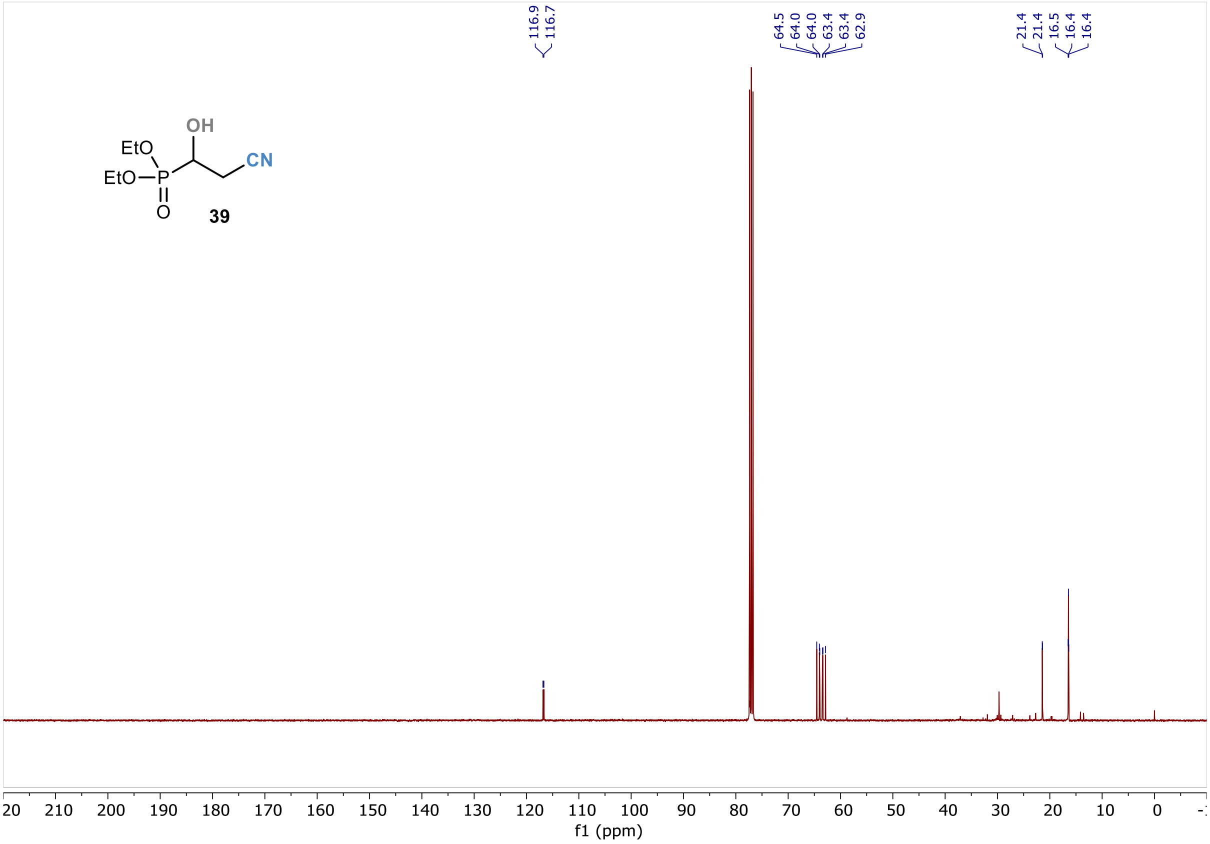

^{31}**P NMR** (162 MHz, $CDCl_3$) for compound **39**:

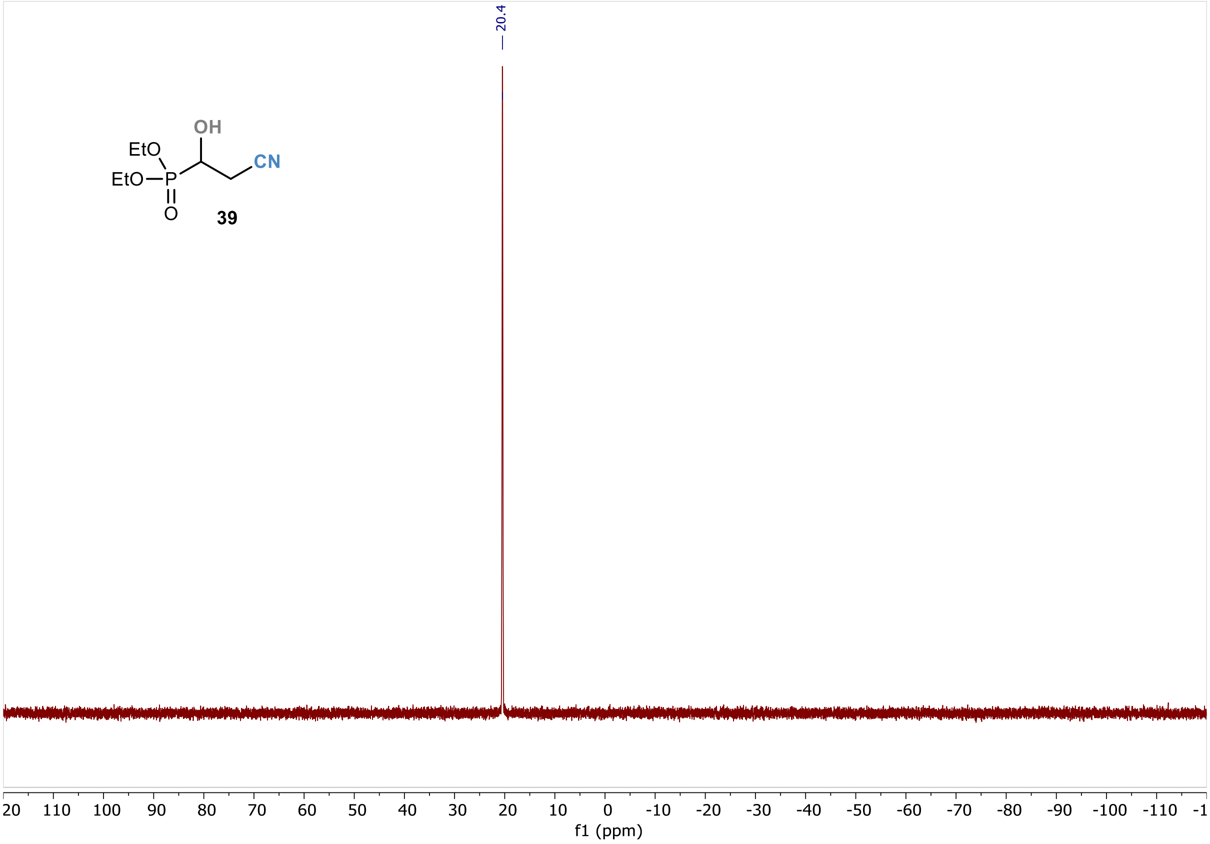

^{1}**H NMR** (400 MHz, $CDCl_3$) for compound **40**:

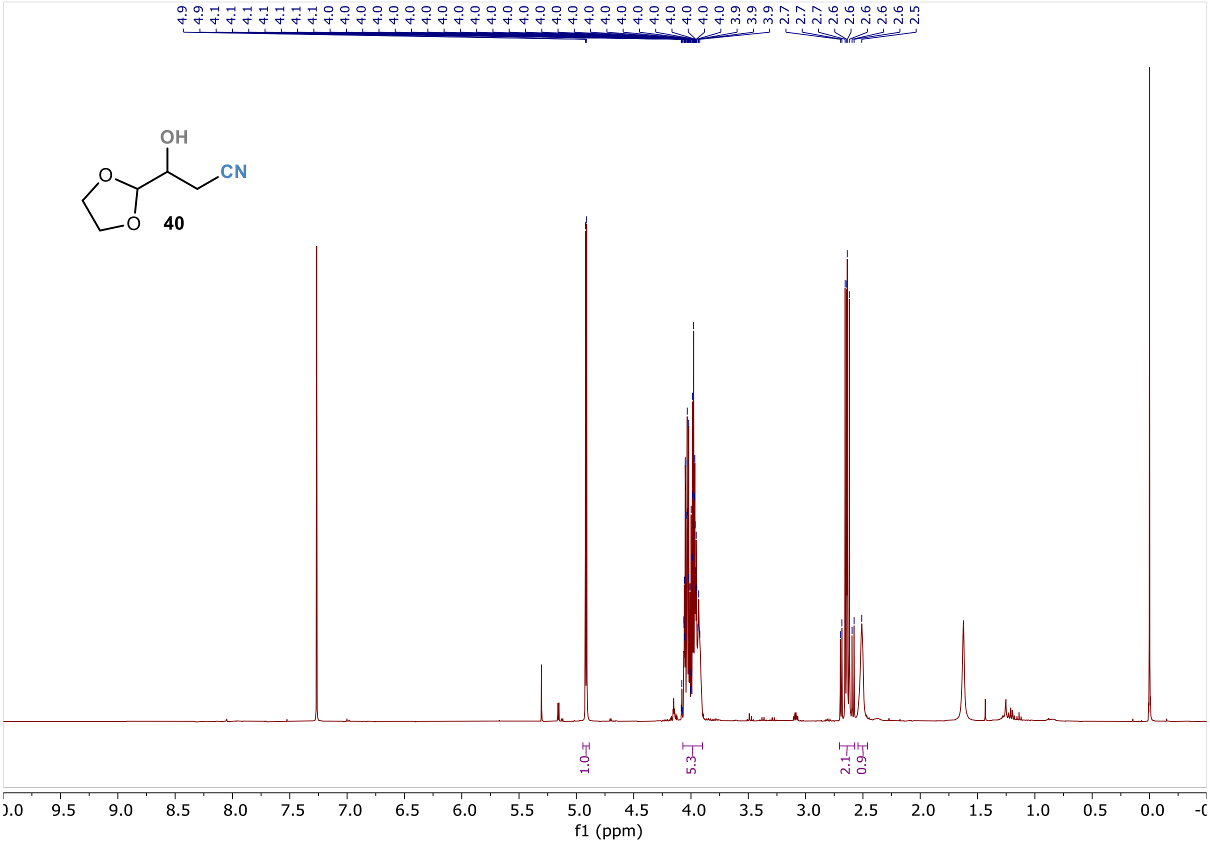

^{13}C NMR (101 MHz, $CDCl_3$) for compound **40**:

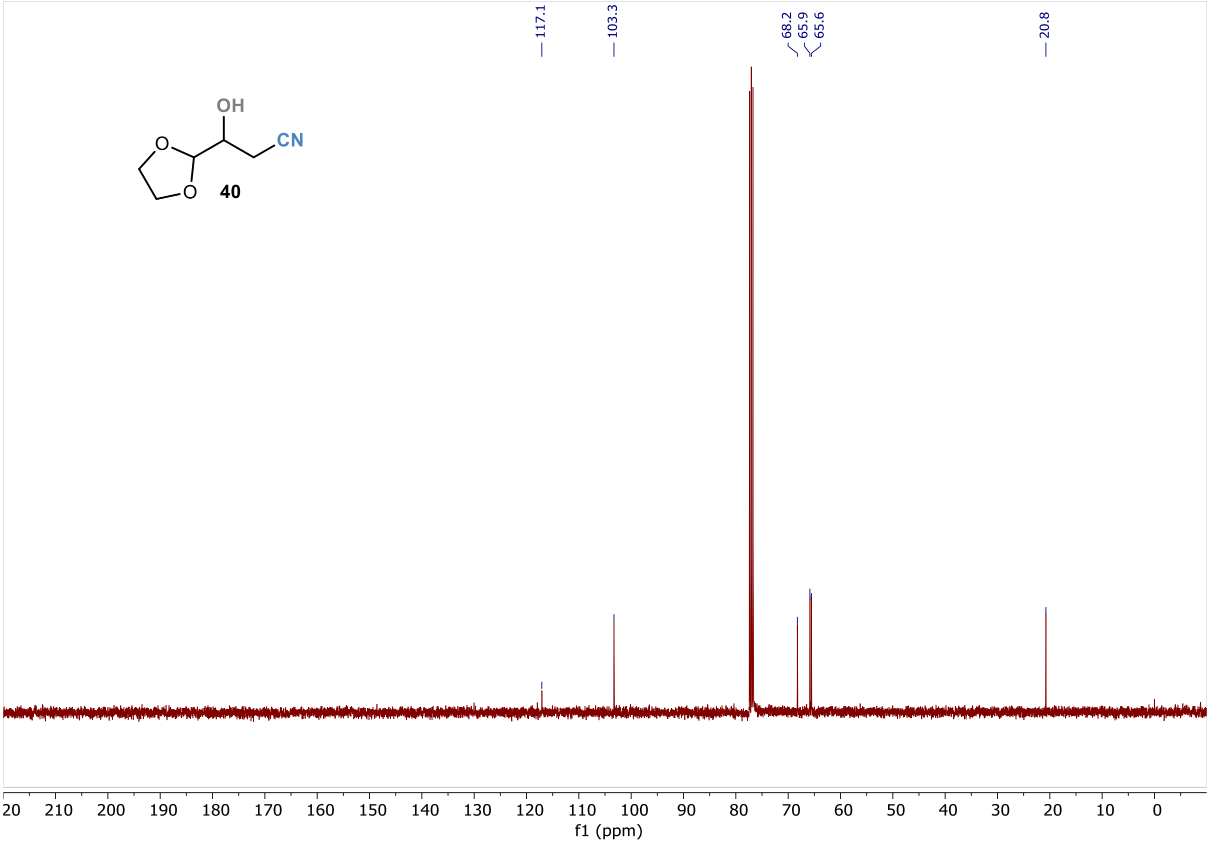

^{1}H NMR (400 MHz, $CDCl_3$) for compound **41**:

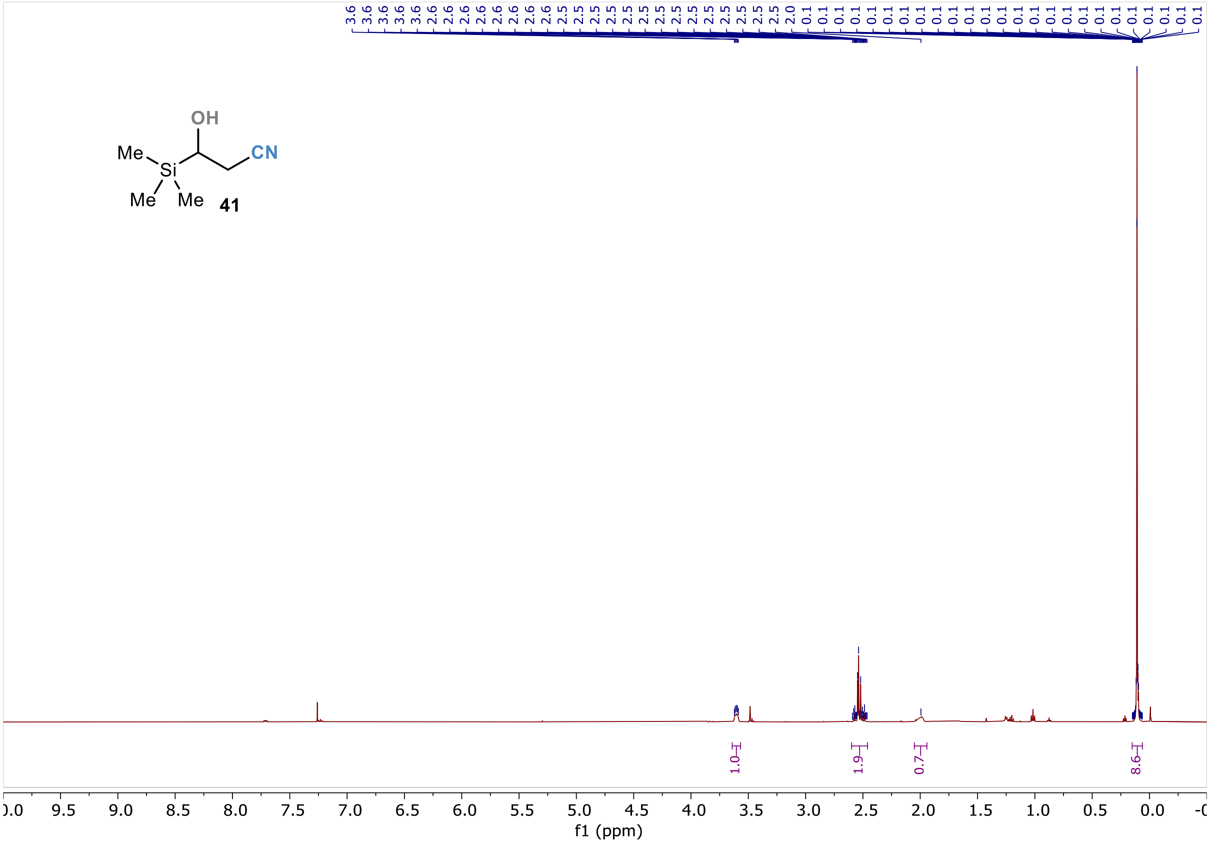

^{13}C NMR (101 MHz, $CDCl_3$) for compound **41**:

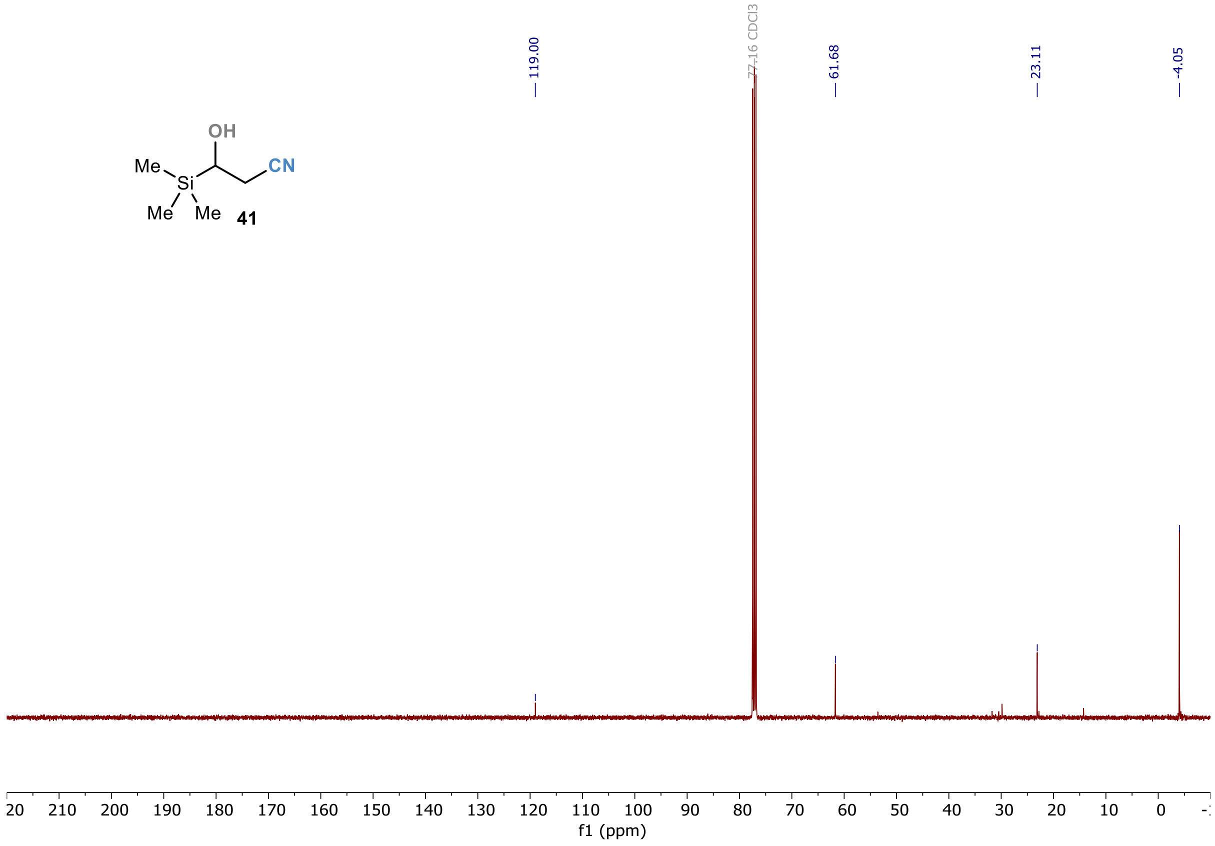

^{1}H NMR (400 MHz, $CDCl_3$) for compound **42**:

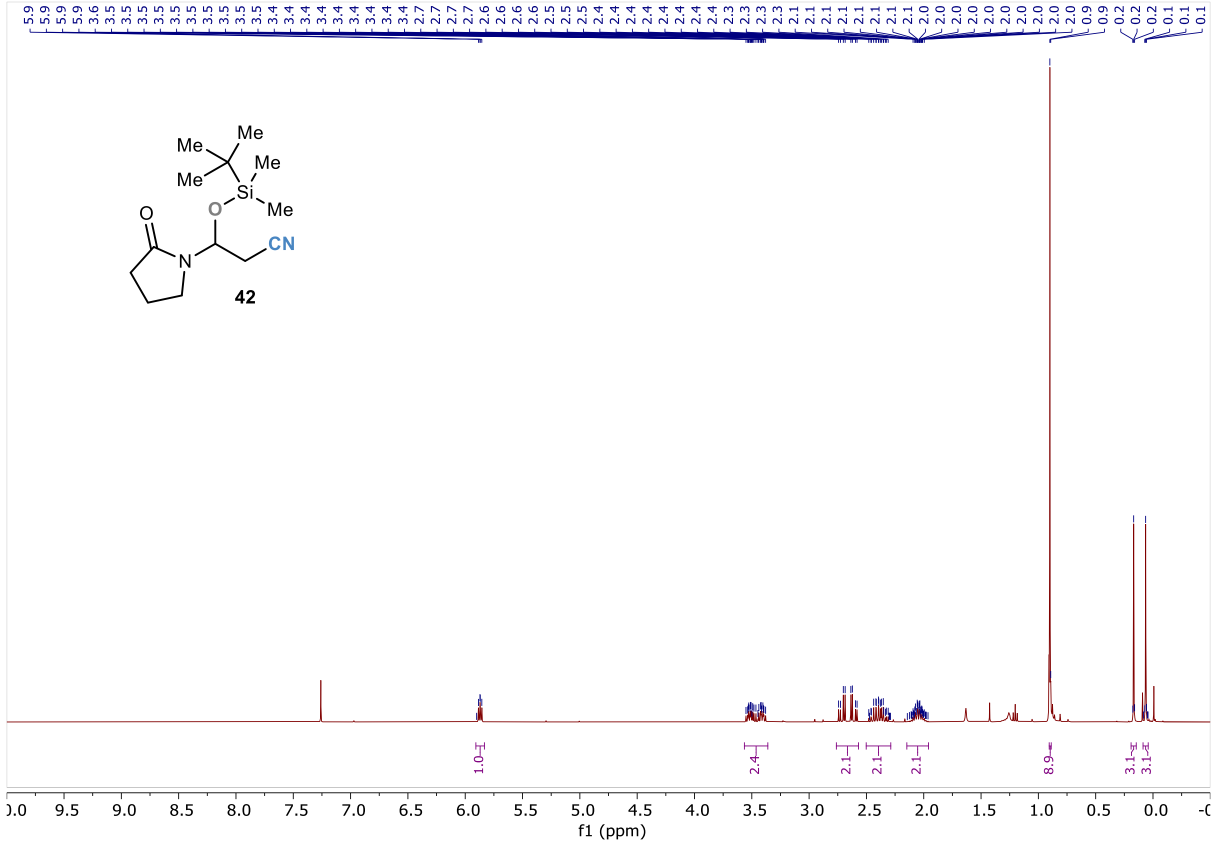

^{13}C NMR (101 MHz, $CDCl_3$) for compound **42**:

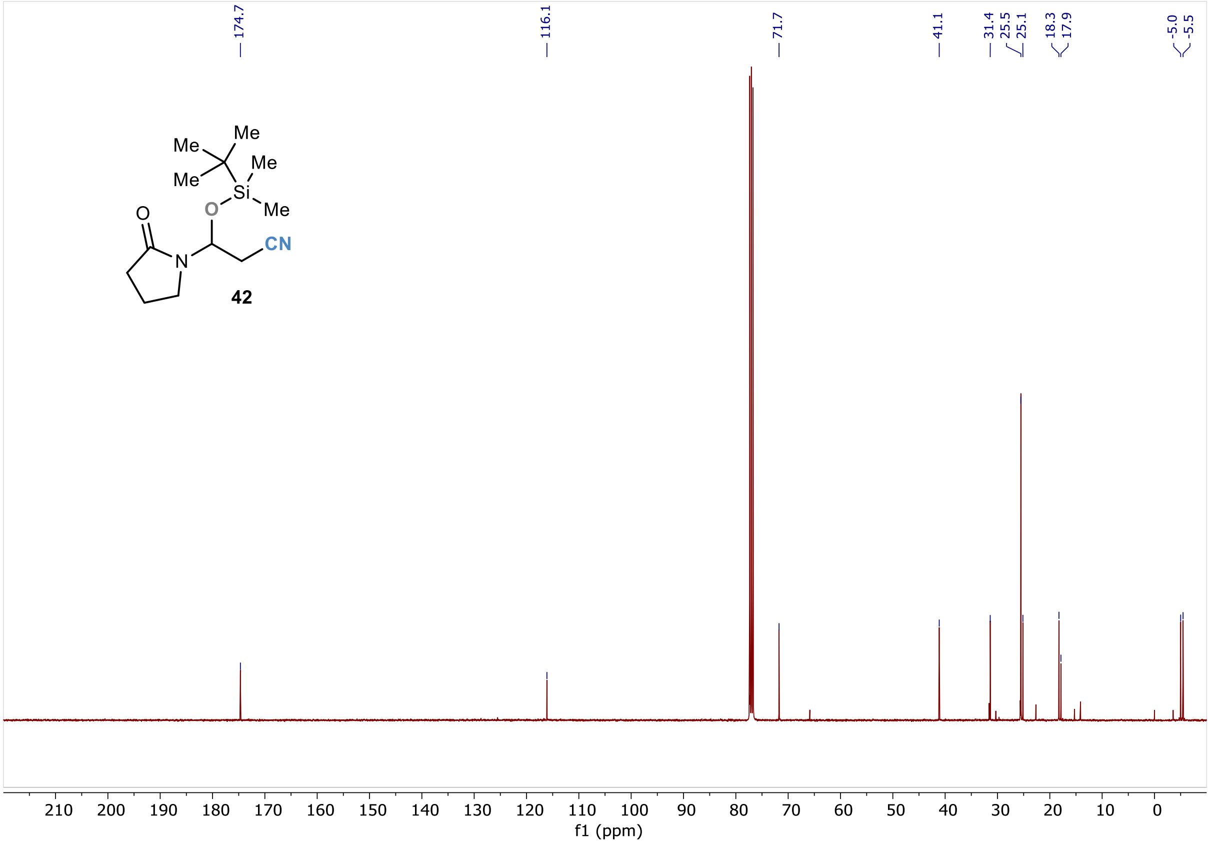

^{1}H NMR (400 MHz, $CDCl_3$) for compound **43**:

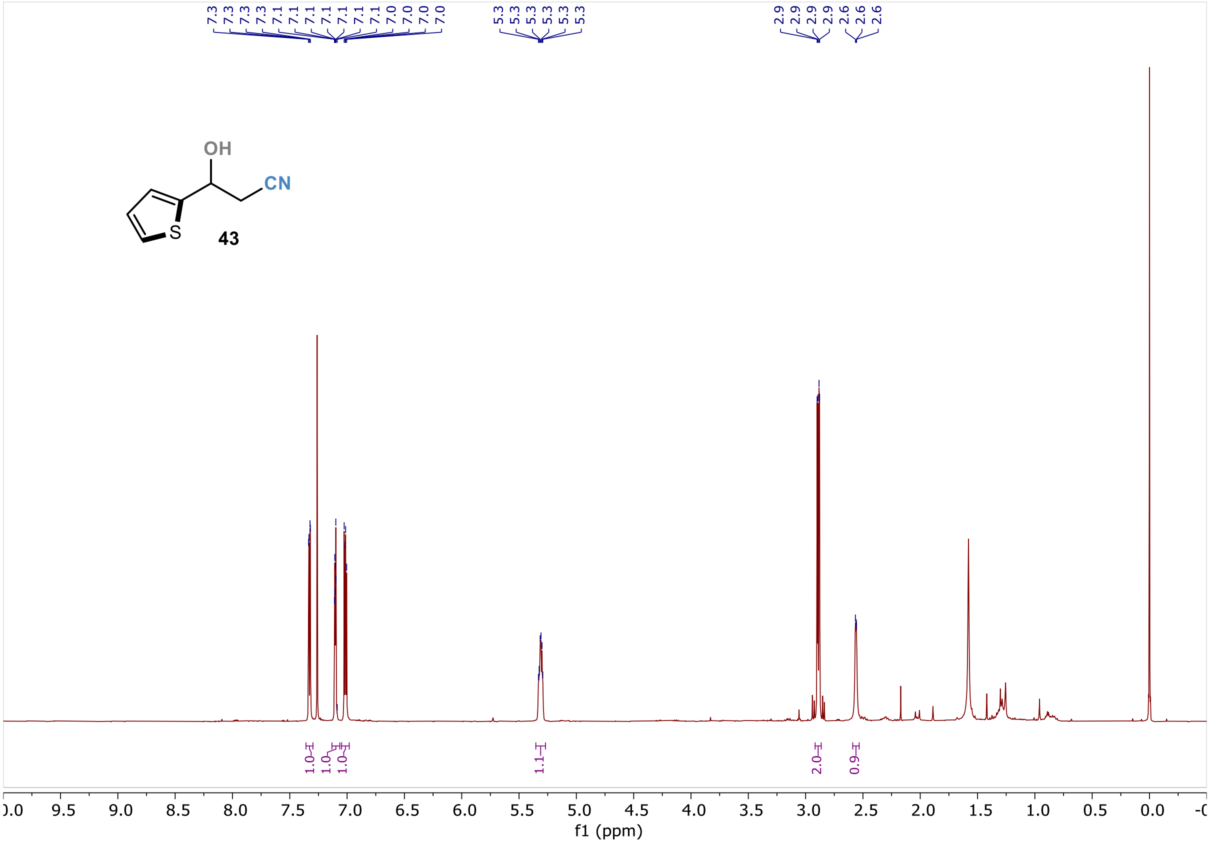

^{13}**C NMR** (101 MHz, $CDCl_3$) for compound **43**:

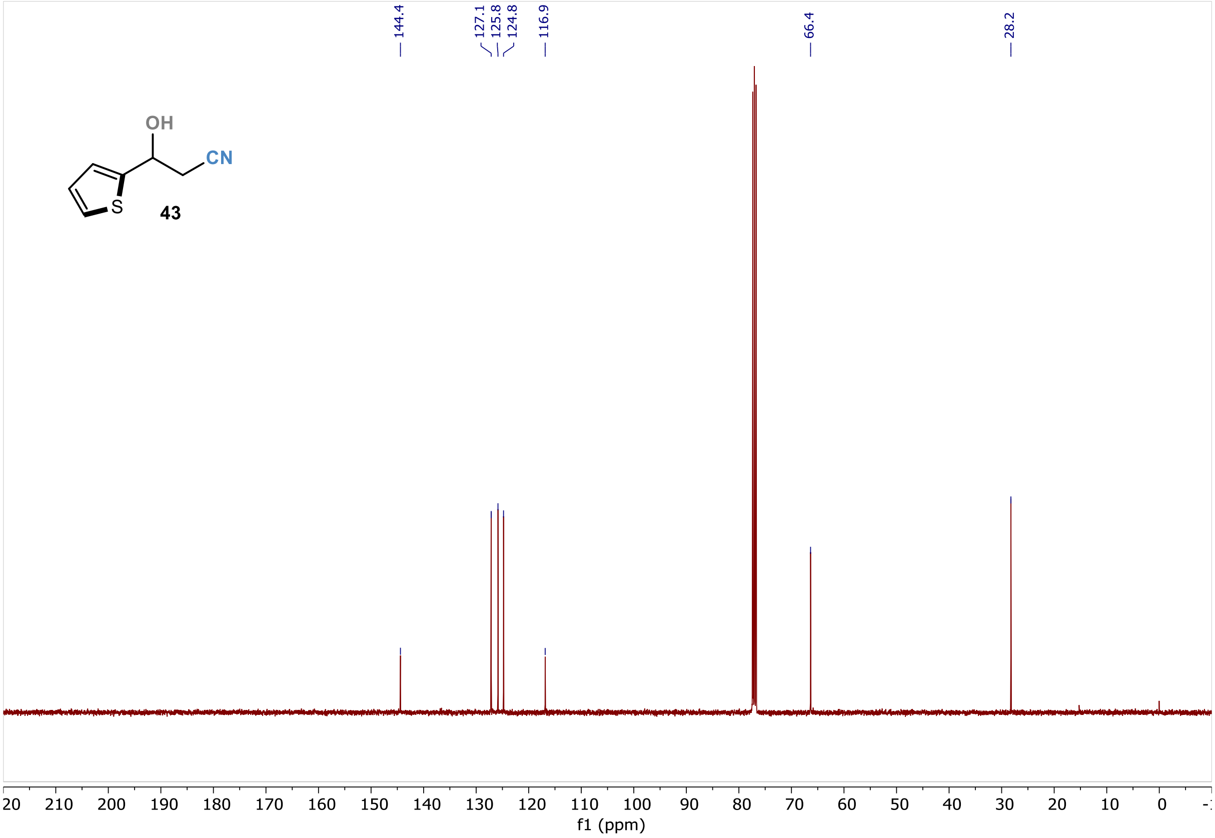

^{1}**H NMR** (400 MHz, $CDCl_3$) for compound **44**:

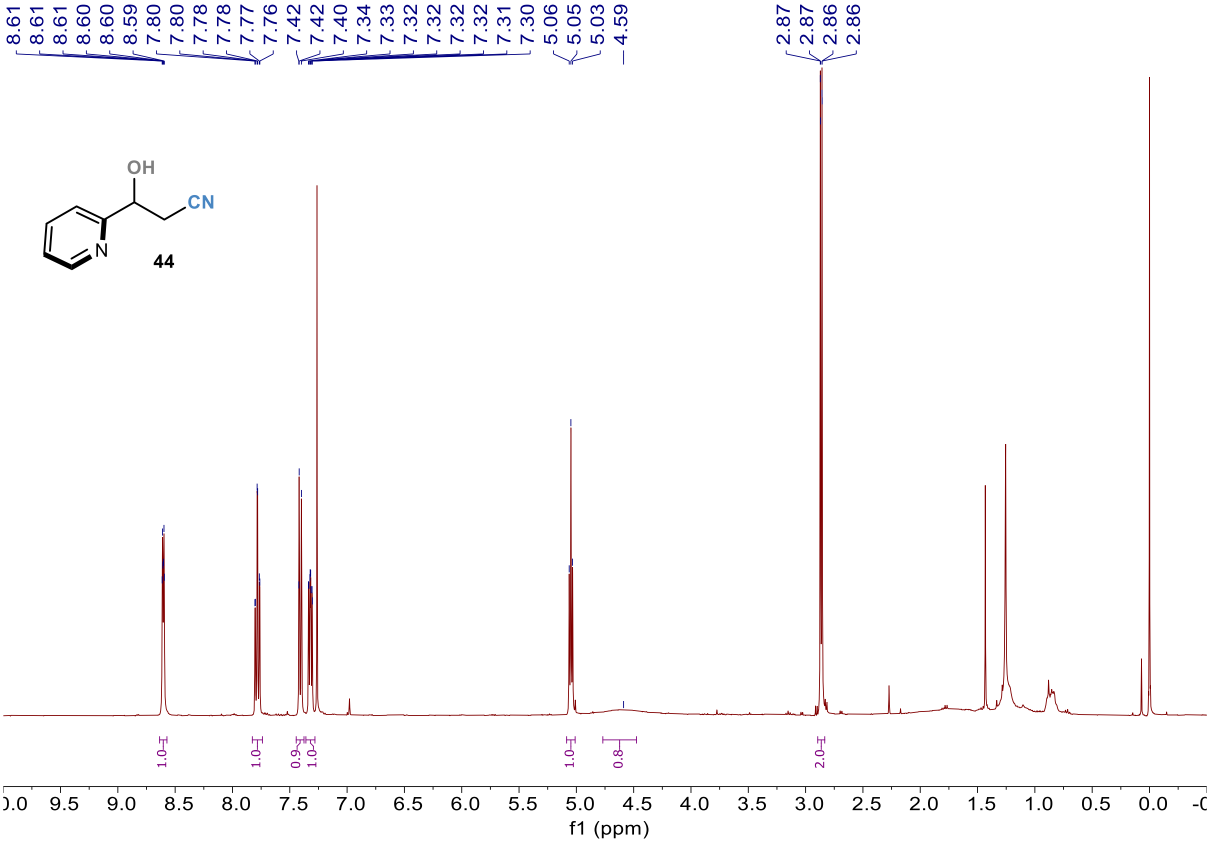

^{13}C NMR (101 MHz, $CDCl_3$) for compound **44**:

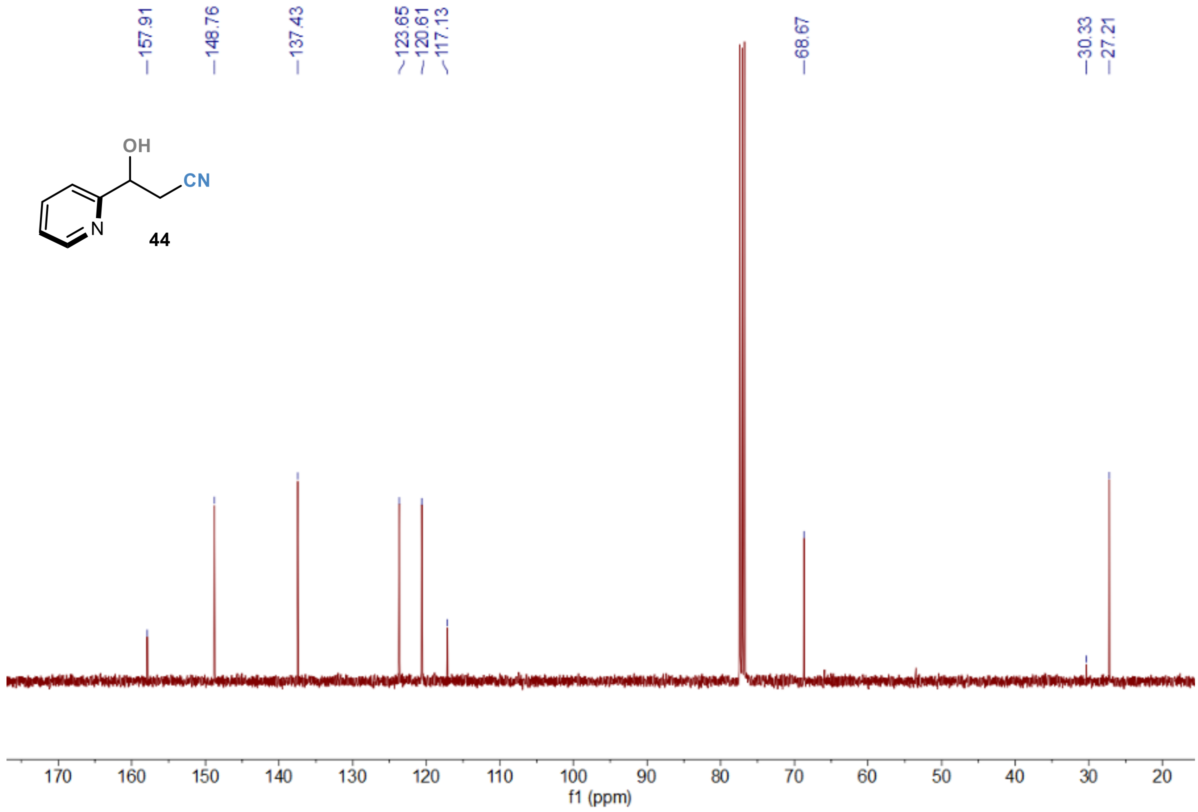

^{1}H NMR (500 MHz, $CDCl_3$) for compound **45**:

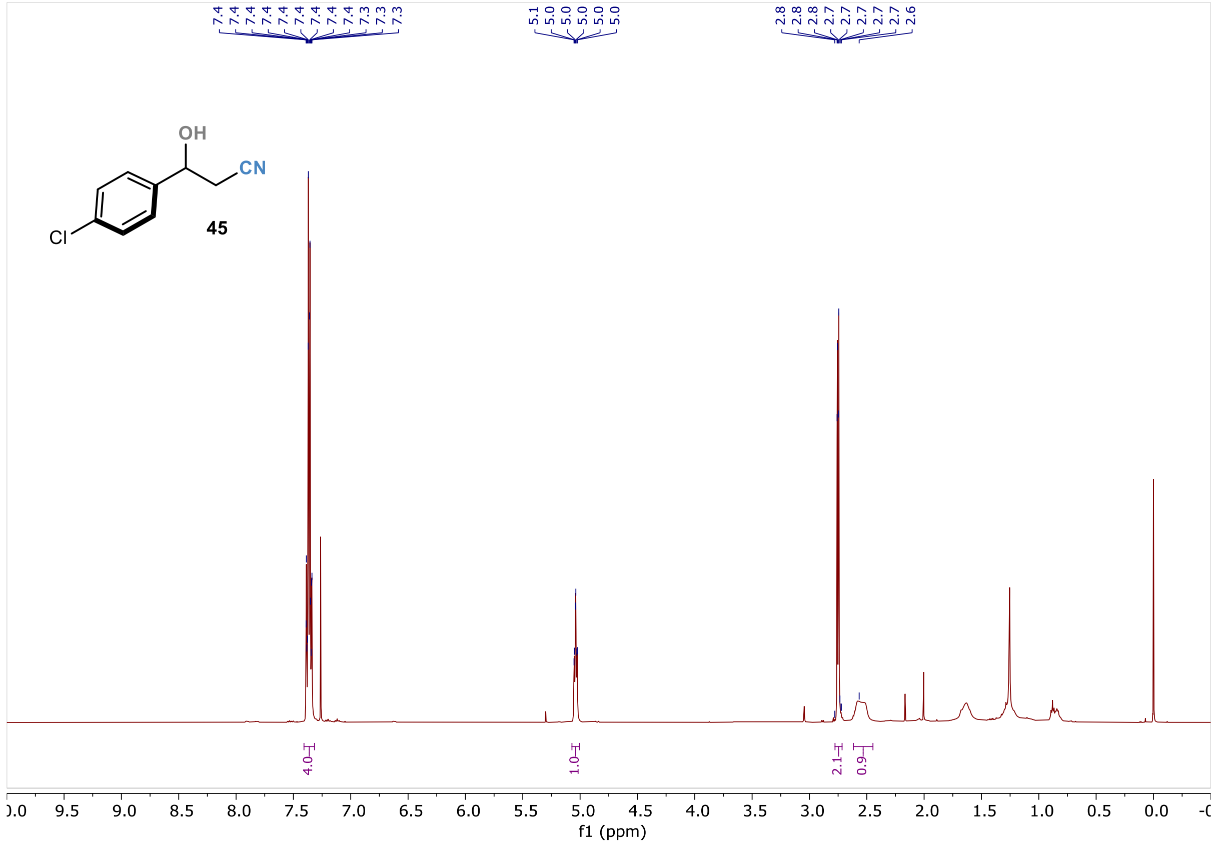

^{13}C NMR (126 MHz, $CDCl_3$) for compound **45**:

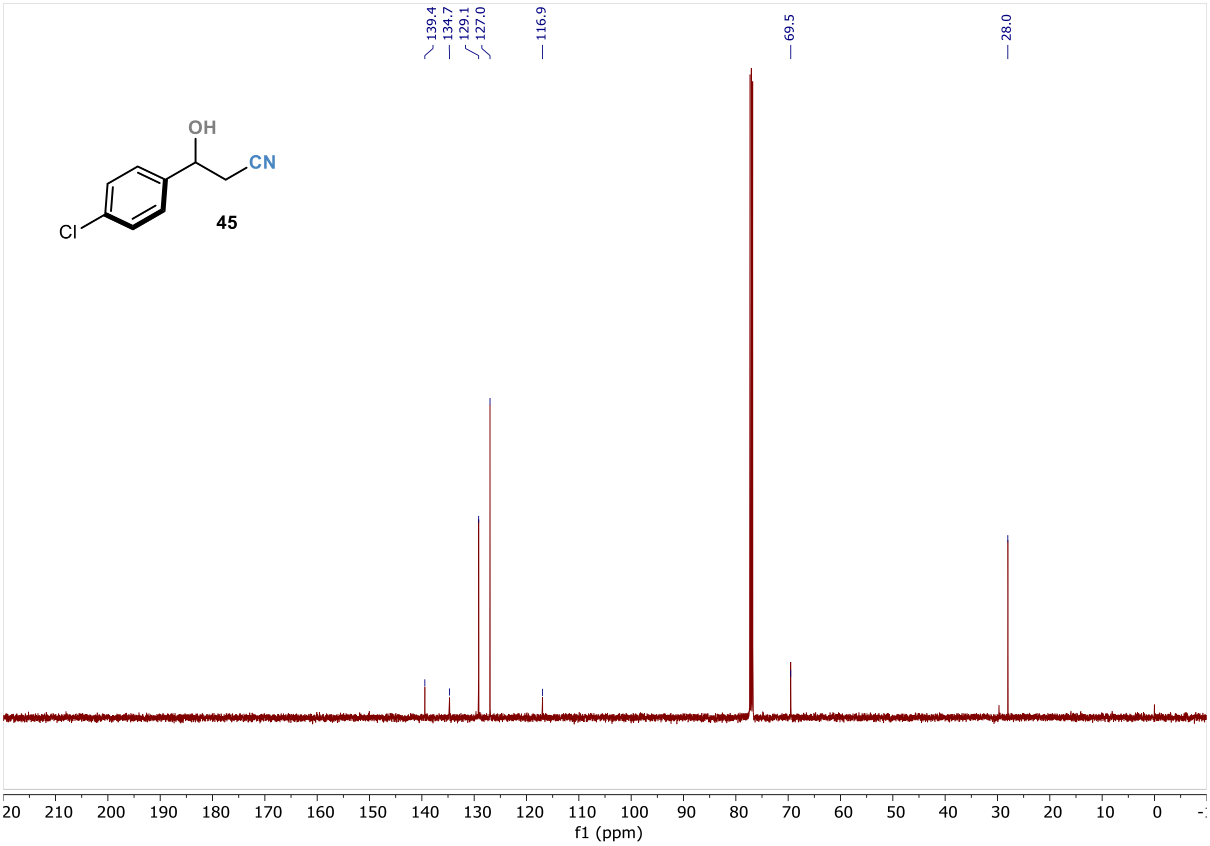

^{1}H NMR (400 MHz, $CDCl_3$) for compound **46**:

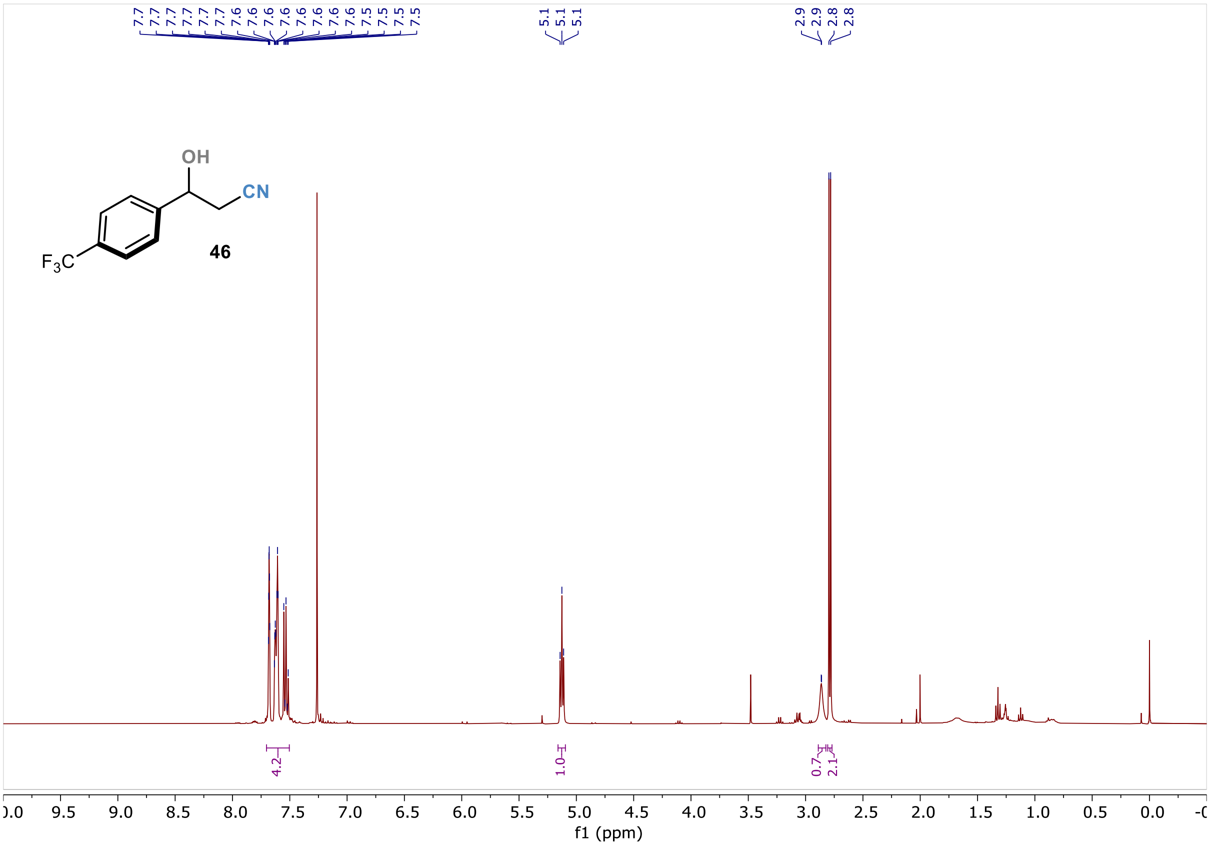

^{13}**C NMR** (101 MHz, $CDCl_3$) for compound **46**:

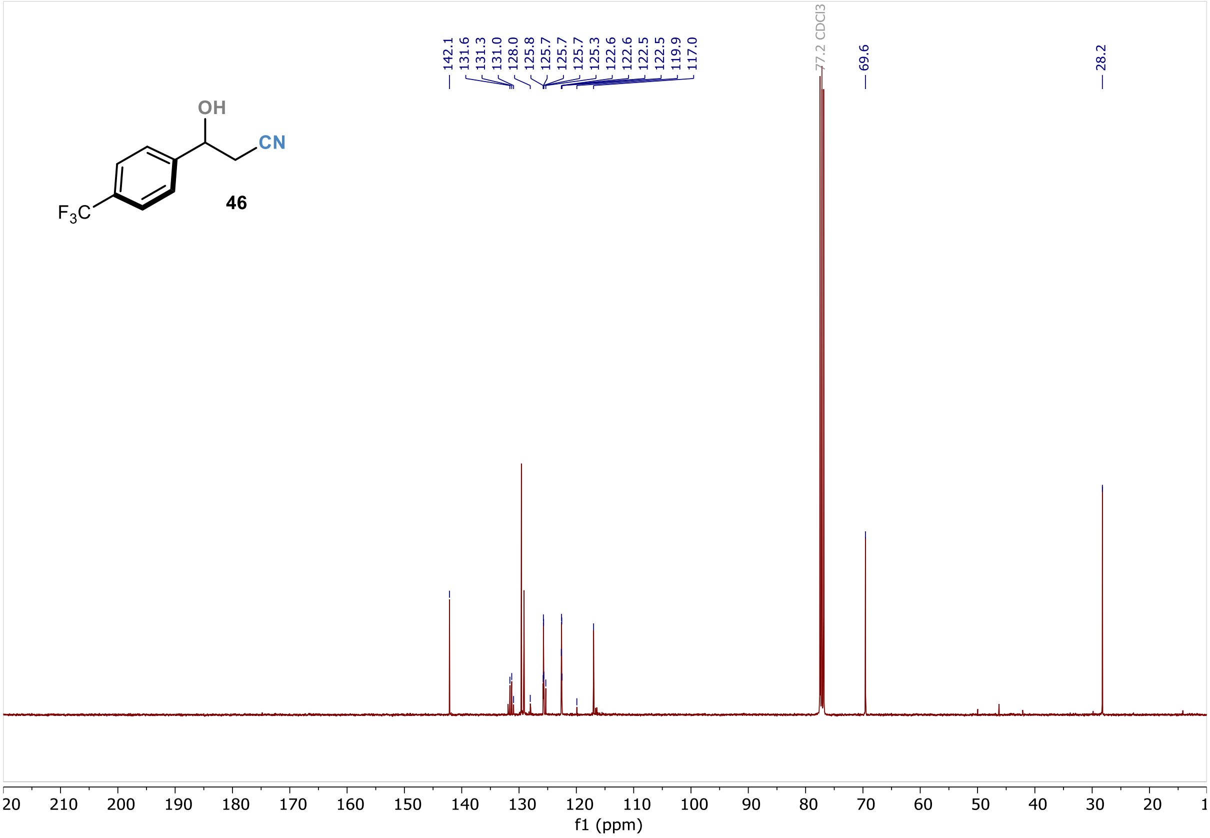

^{19}**F NMR** (376 MHz, $CDCl_3$) for compound **46**:

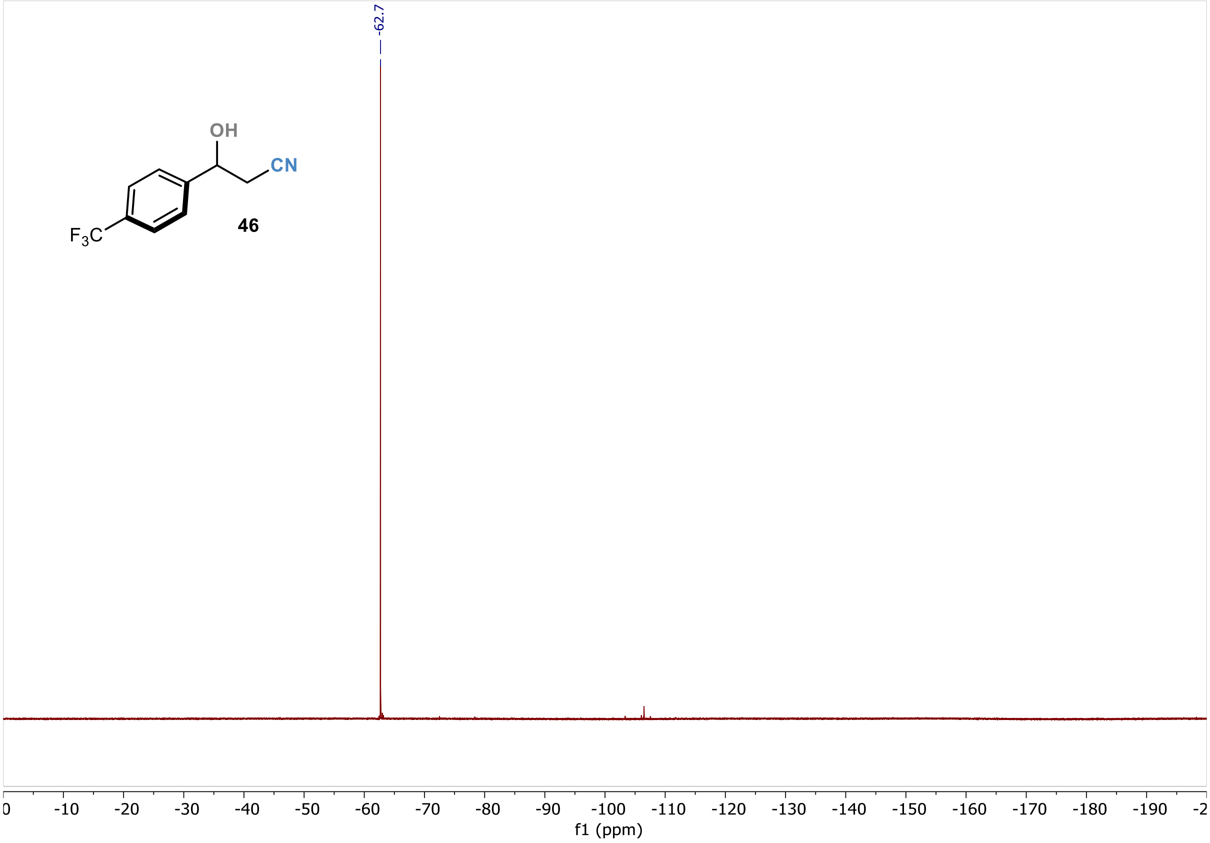

¹H NMR (500 MHz, $CDCl_3$) for compound **47**:

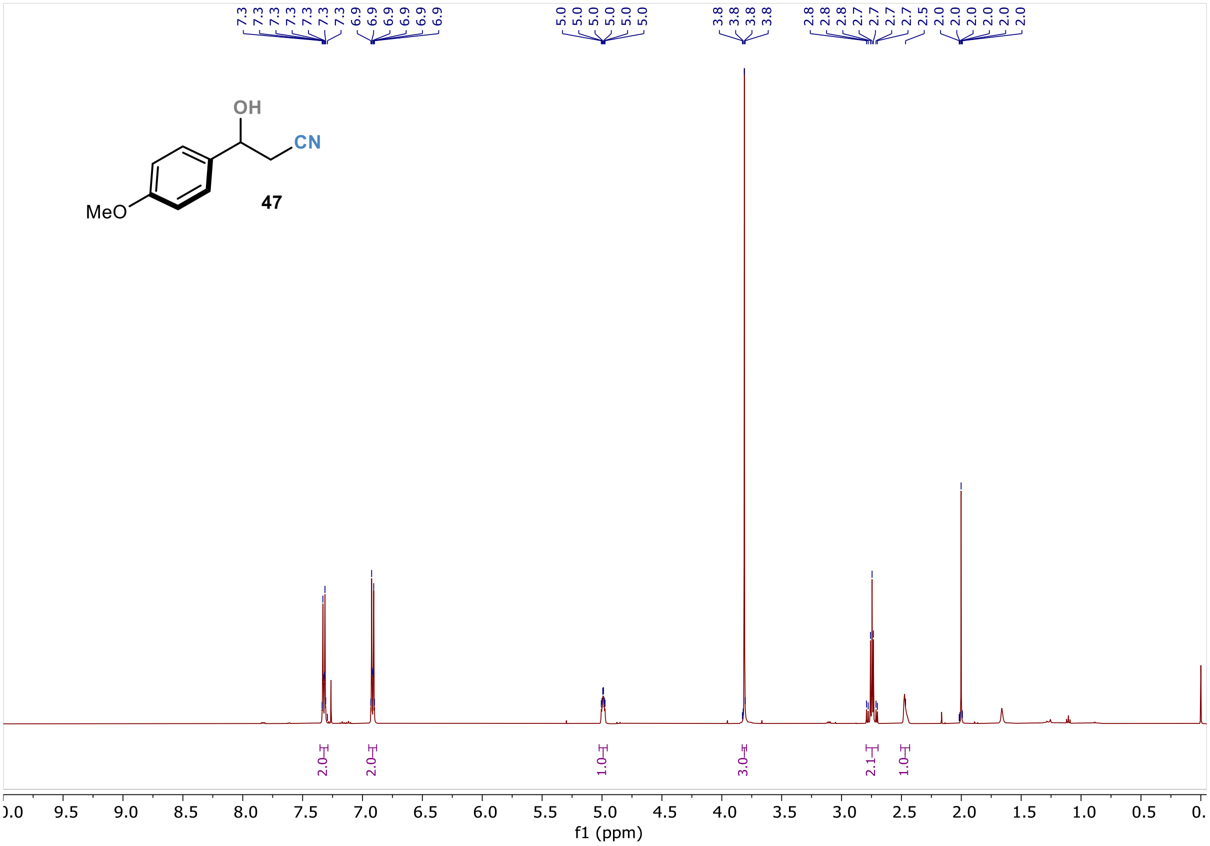

¹³C NMR (126 MHz, $CDCl_3$) for compound **47**:

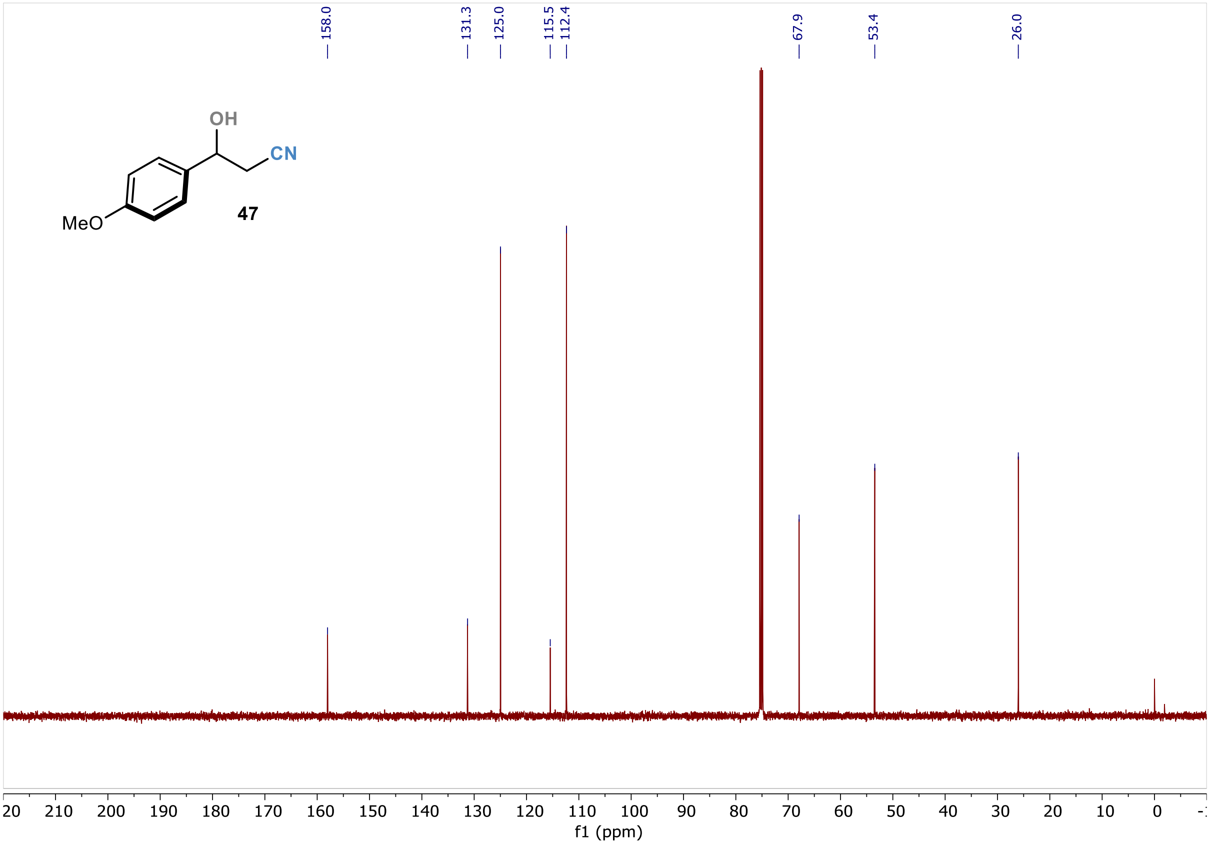

^{1}H NMR (400 MHz, $CDCl_3$) for compound **48**:

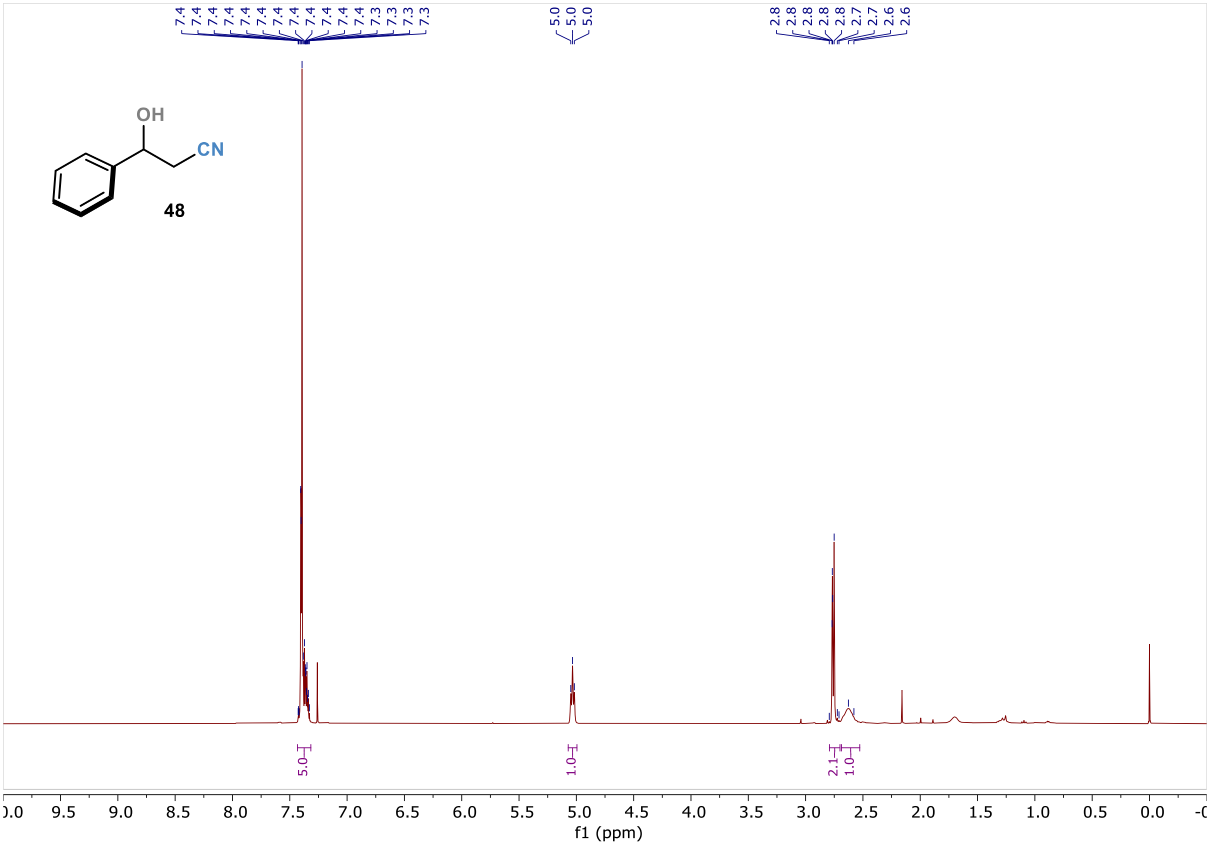

^{13}C NMR (101 MHz, $CDCl_3$) for compound **48**:

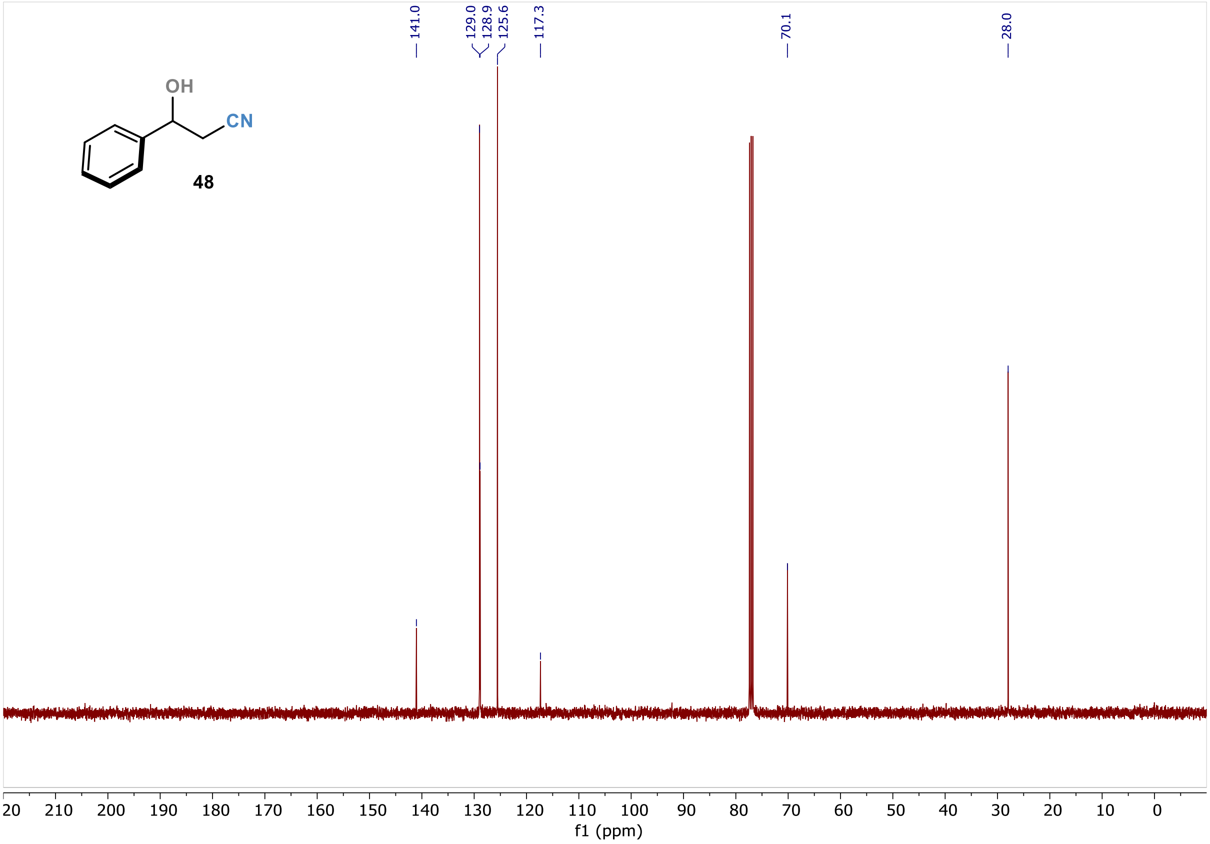

^{1}H NMR (500 MHz, $CDCl_3$) for compound **49**:

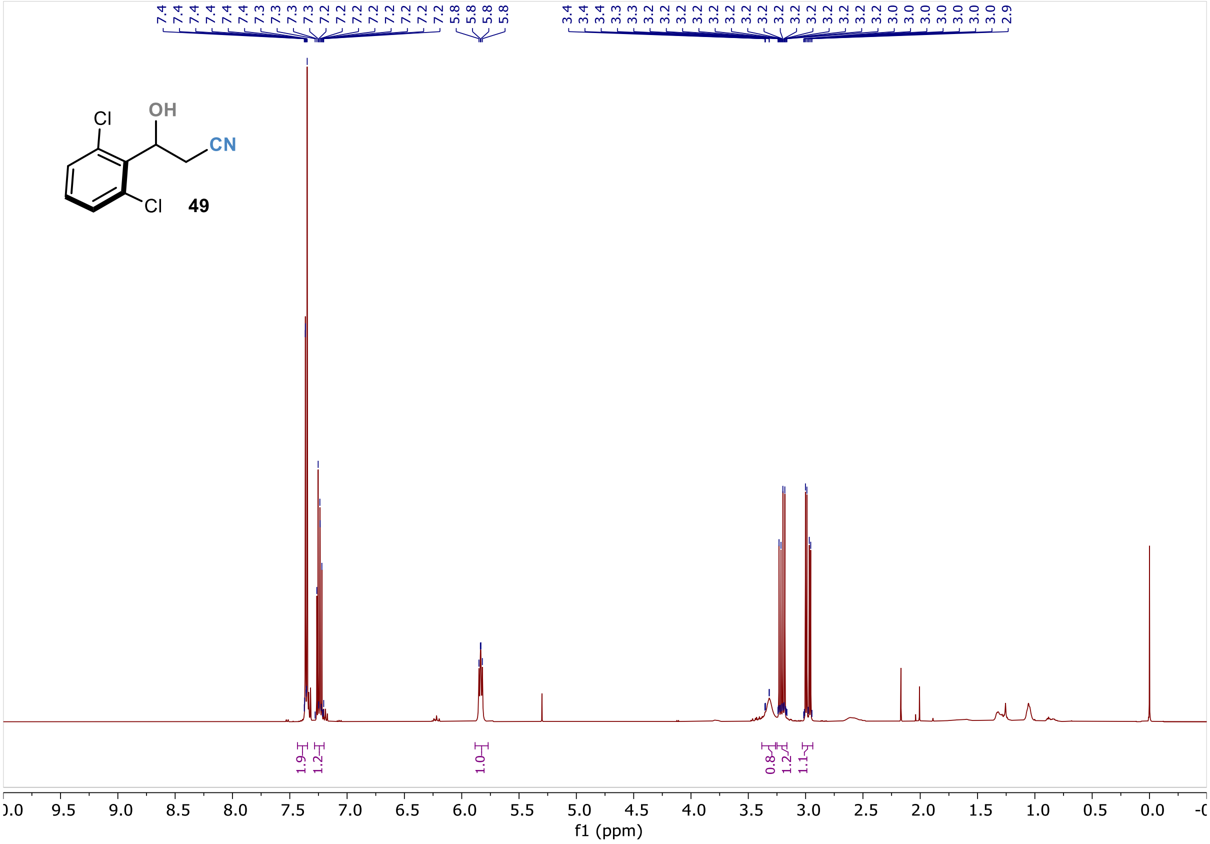

^{13}C NMR (126 MHz, $CDCl_3$) for compound **49**:

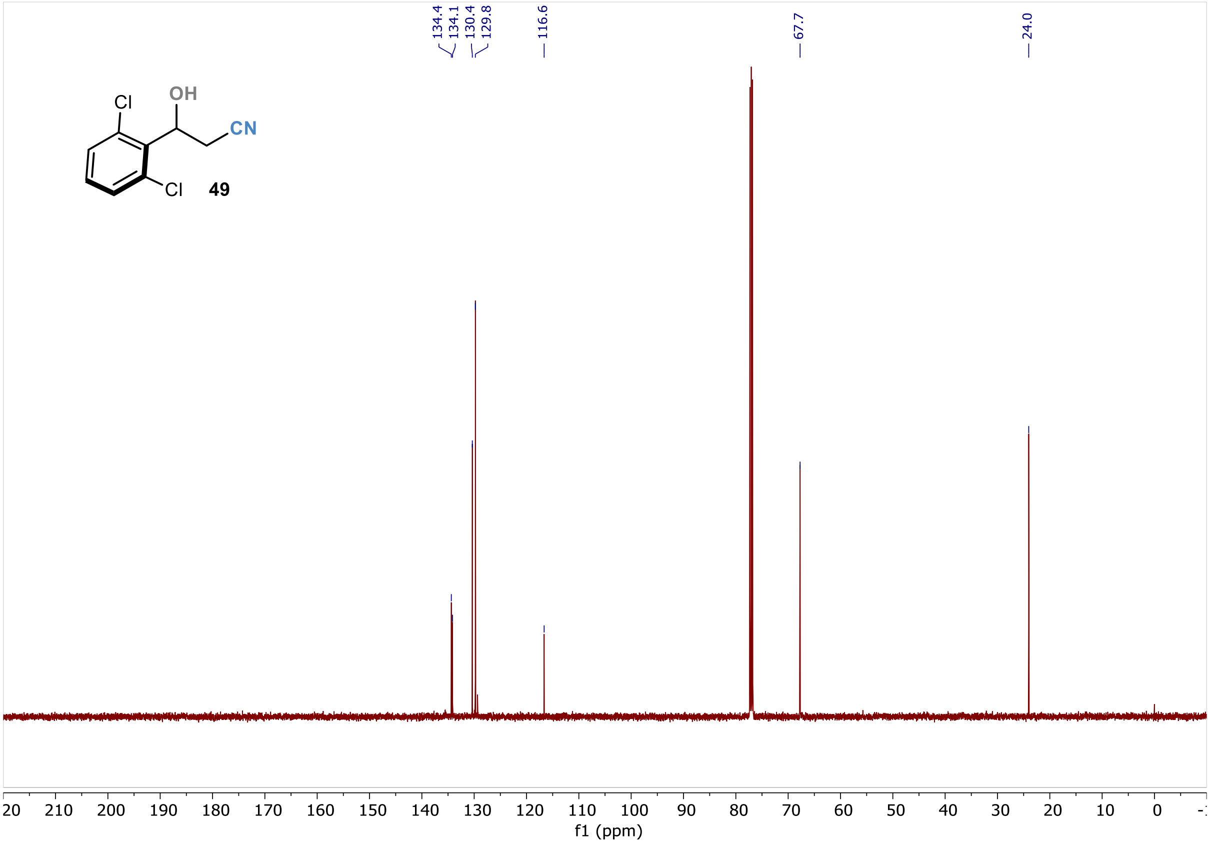

[1]**H NMR** (400 MHz, $CDCl_3$) for compound **50**:

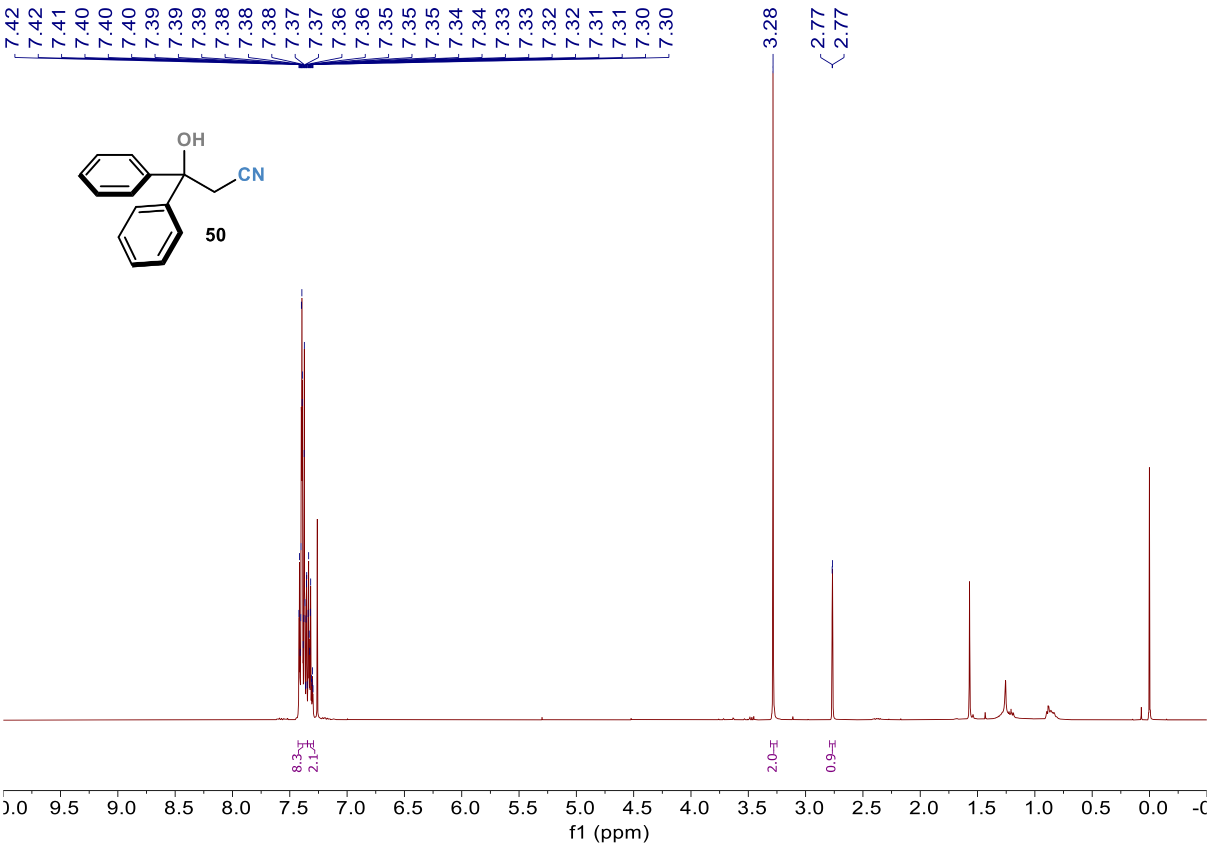

[13]**C NMR** (101 MHz, $CDCl_3$) for compound **50**:

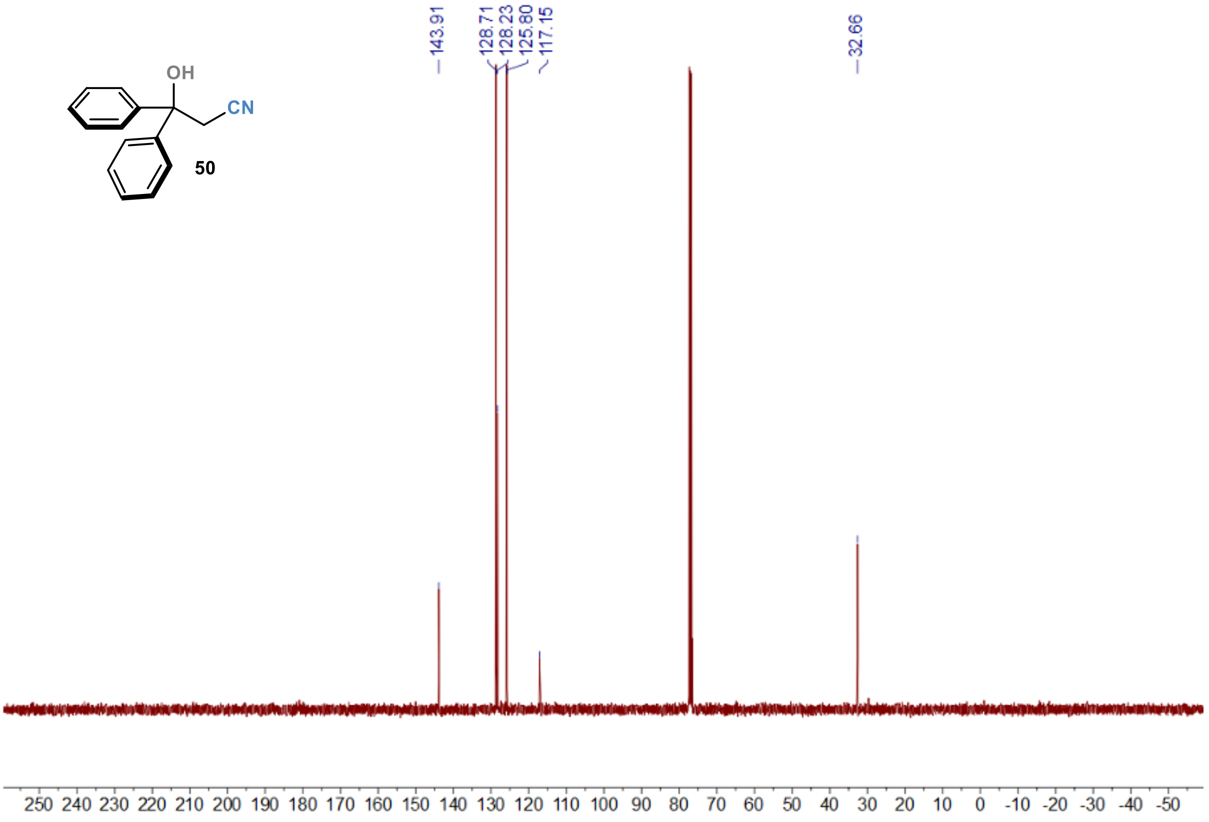

¹H NMR (500 MHz, $CDCl_3$) for compound **51**:

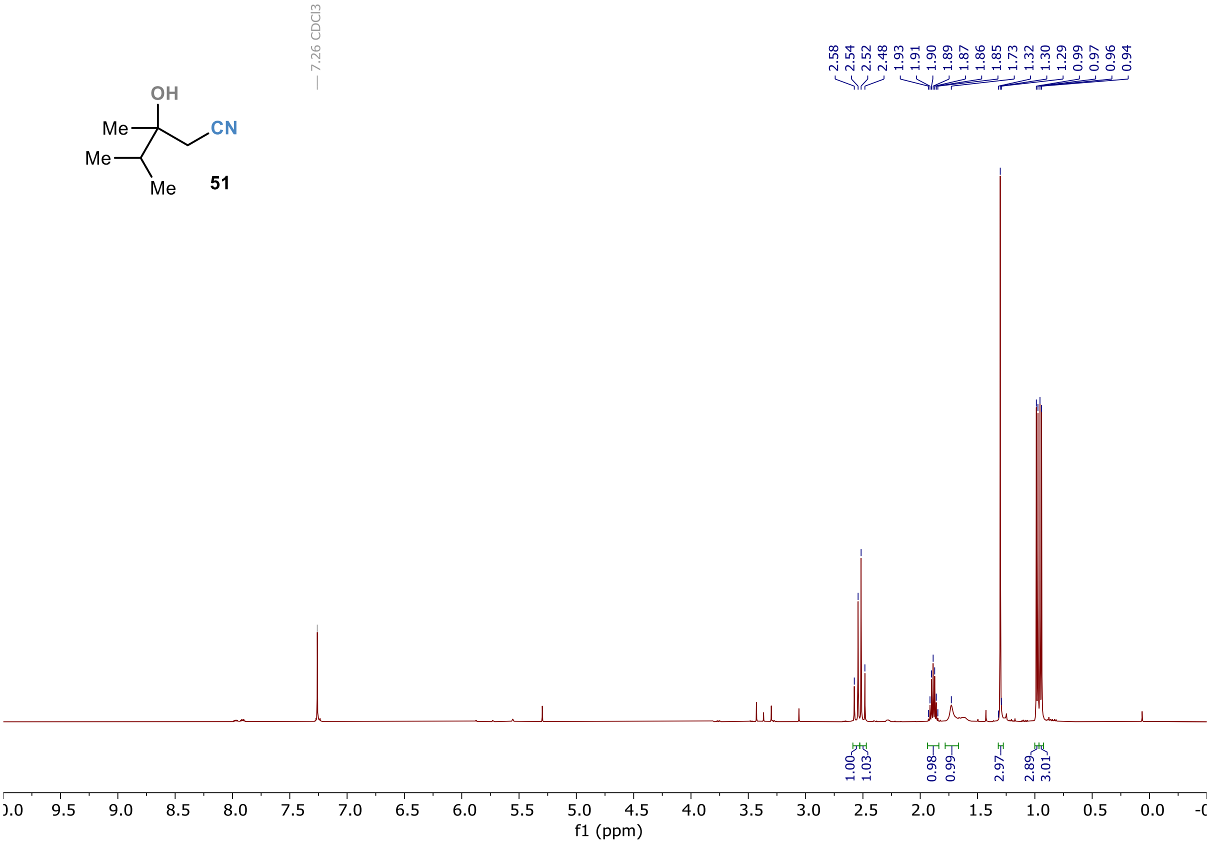

¹³C NMR (126 MHz, $CDCl_3$) for compound **51**:

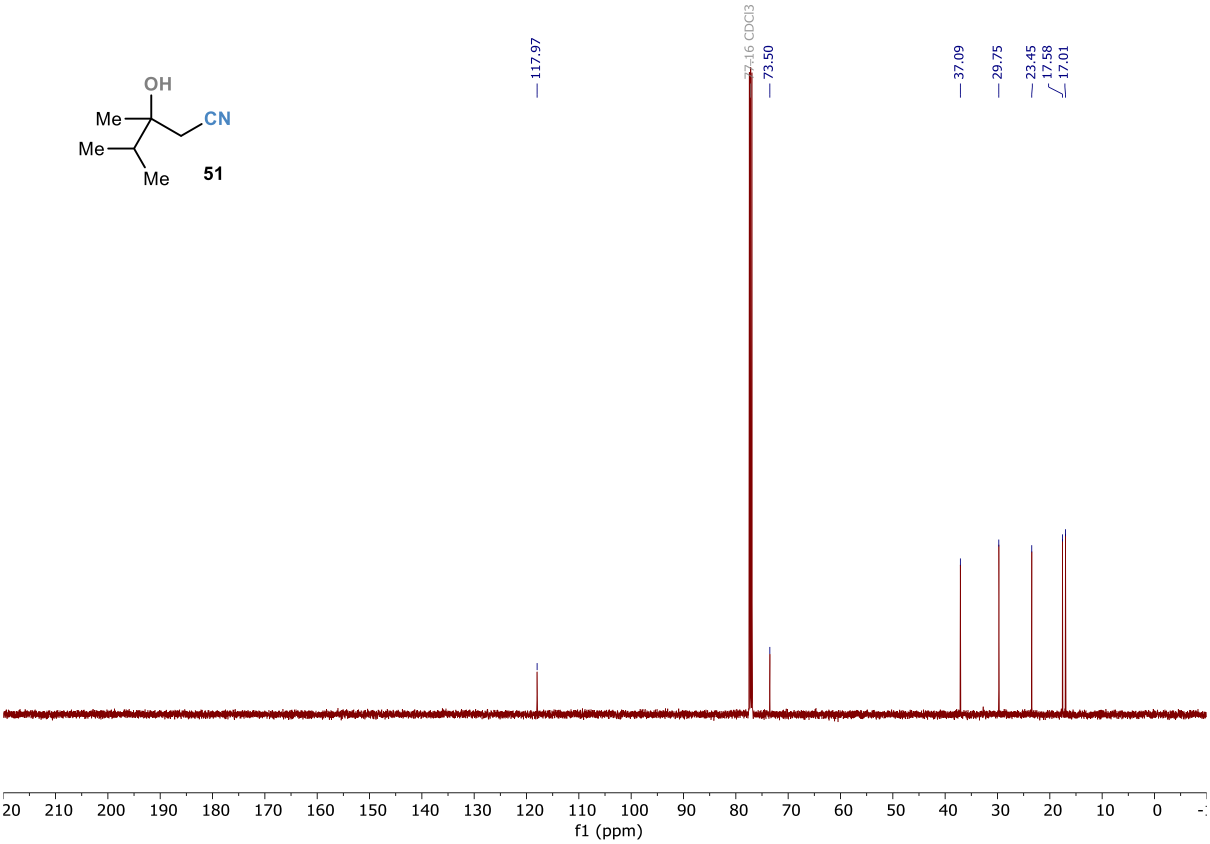

^{1}H NMR (400 MHz, $CDCl_3$) for compound **52**:

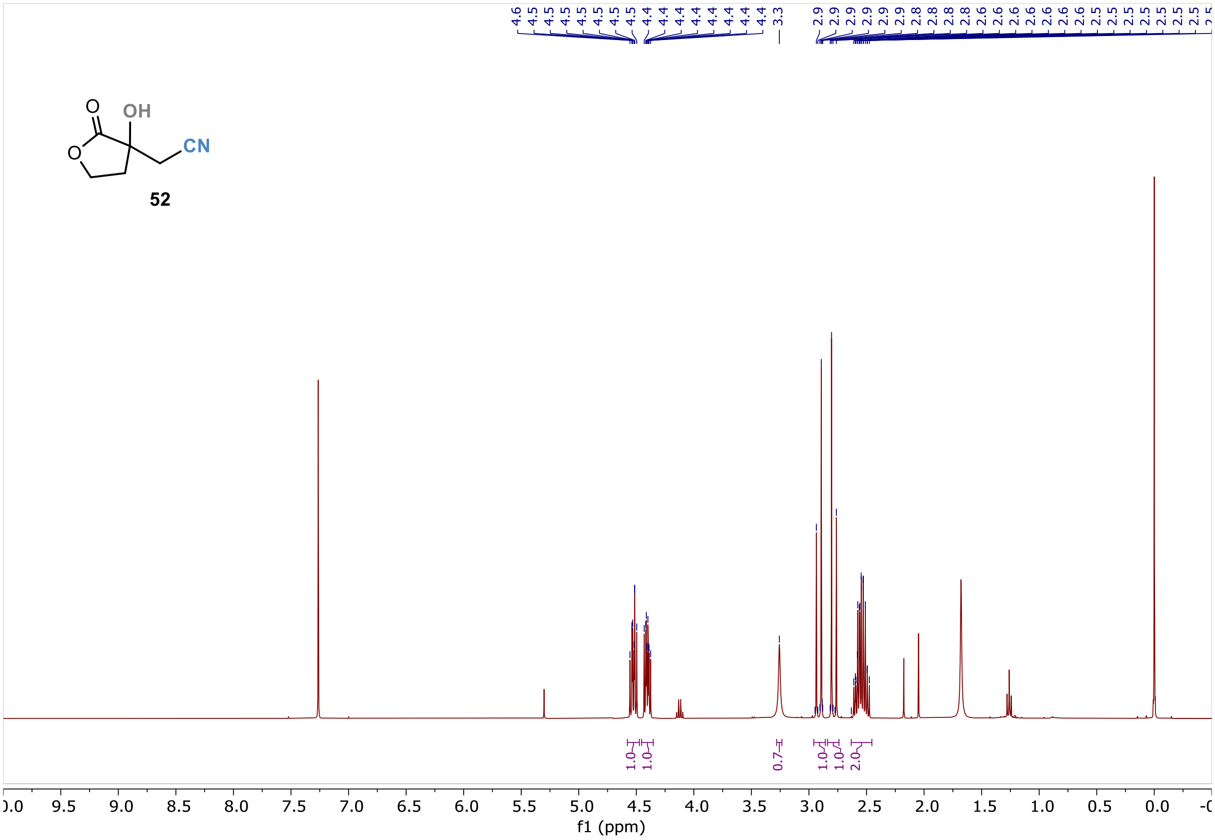

^{13}C NMR (101 MHz, $CDCl_3$) for compound **52**:

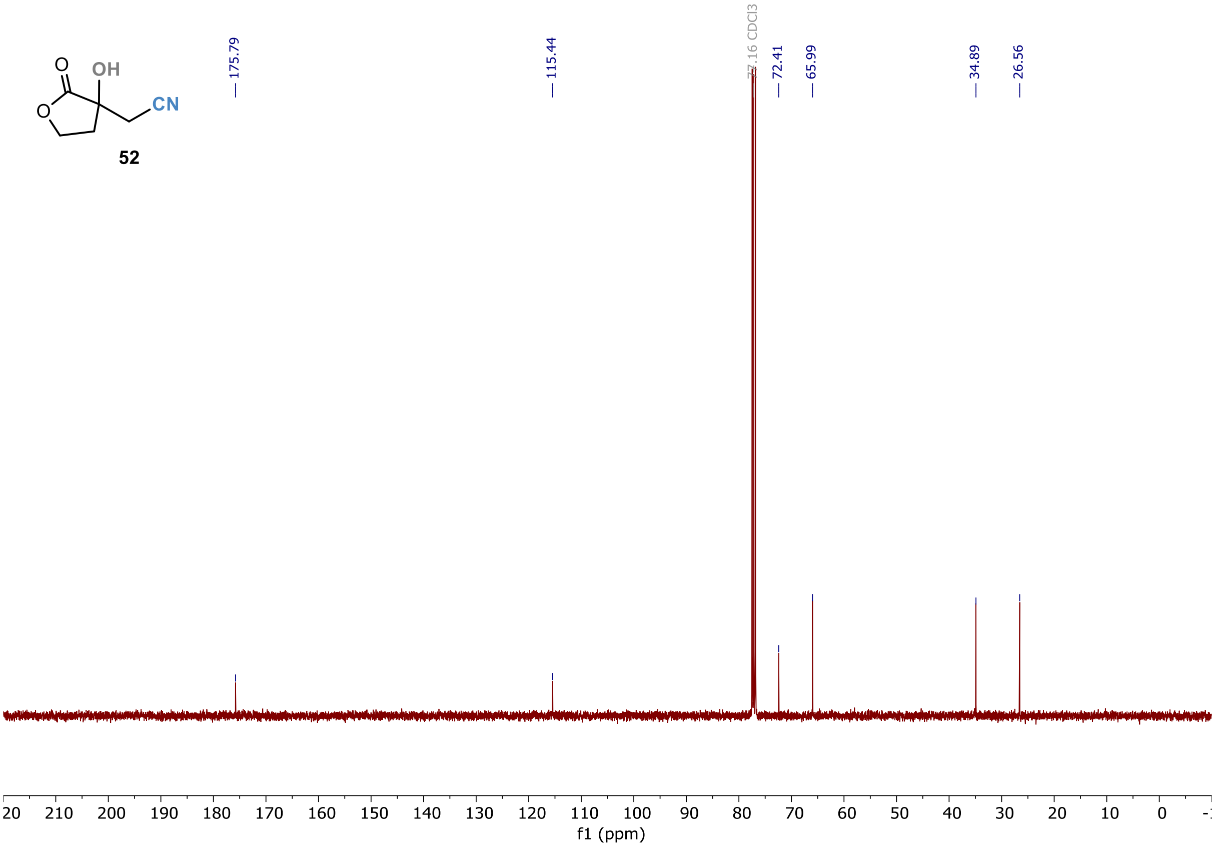

^{1}H NMR (400 MHz, $CDCl_3$) for compound **53**:

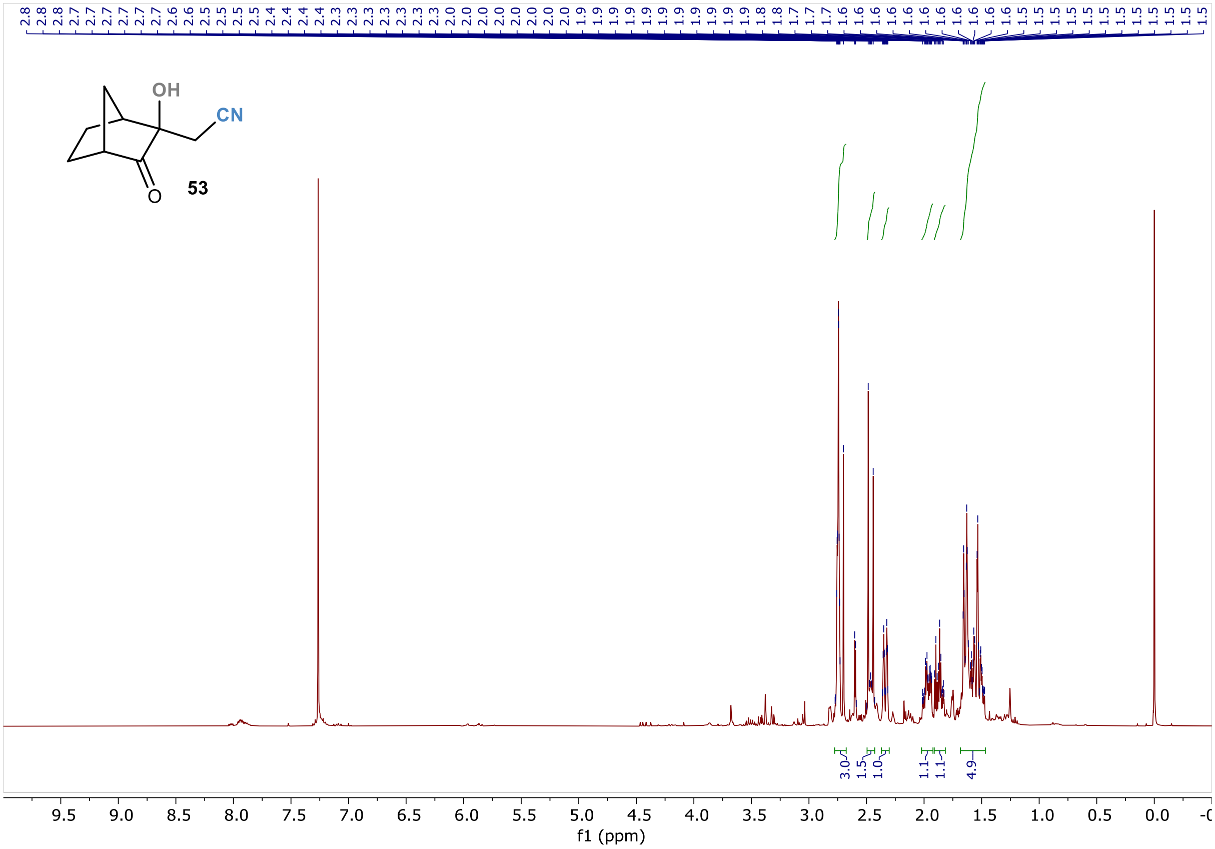

^{13}C NMR (101 MHz, $CDCl_3$) for compound **53**:

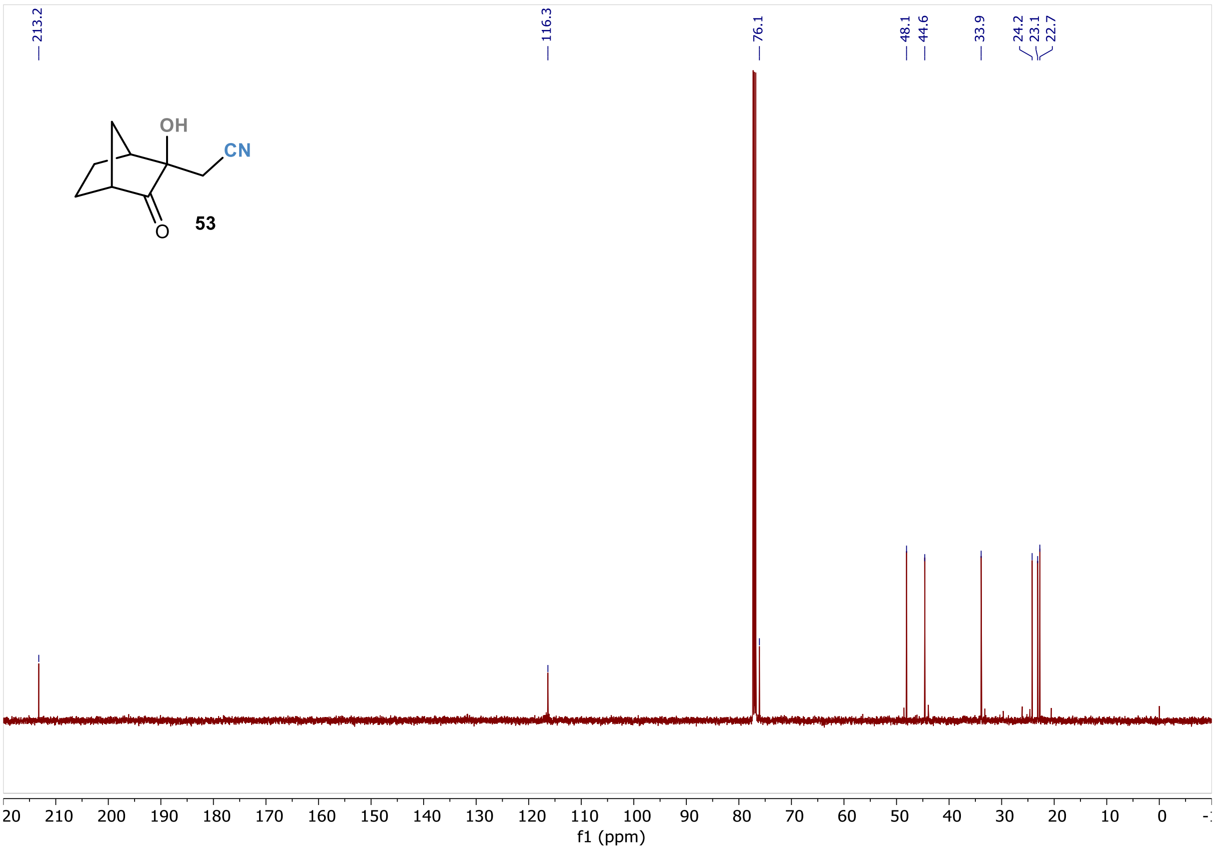

¹H NMR (400 MHz, CD_3OD) for compound **54**:

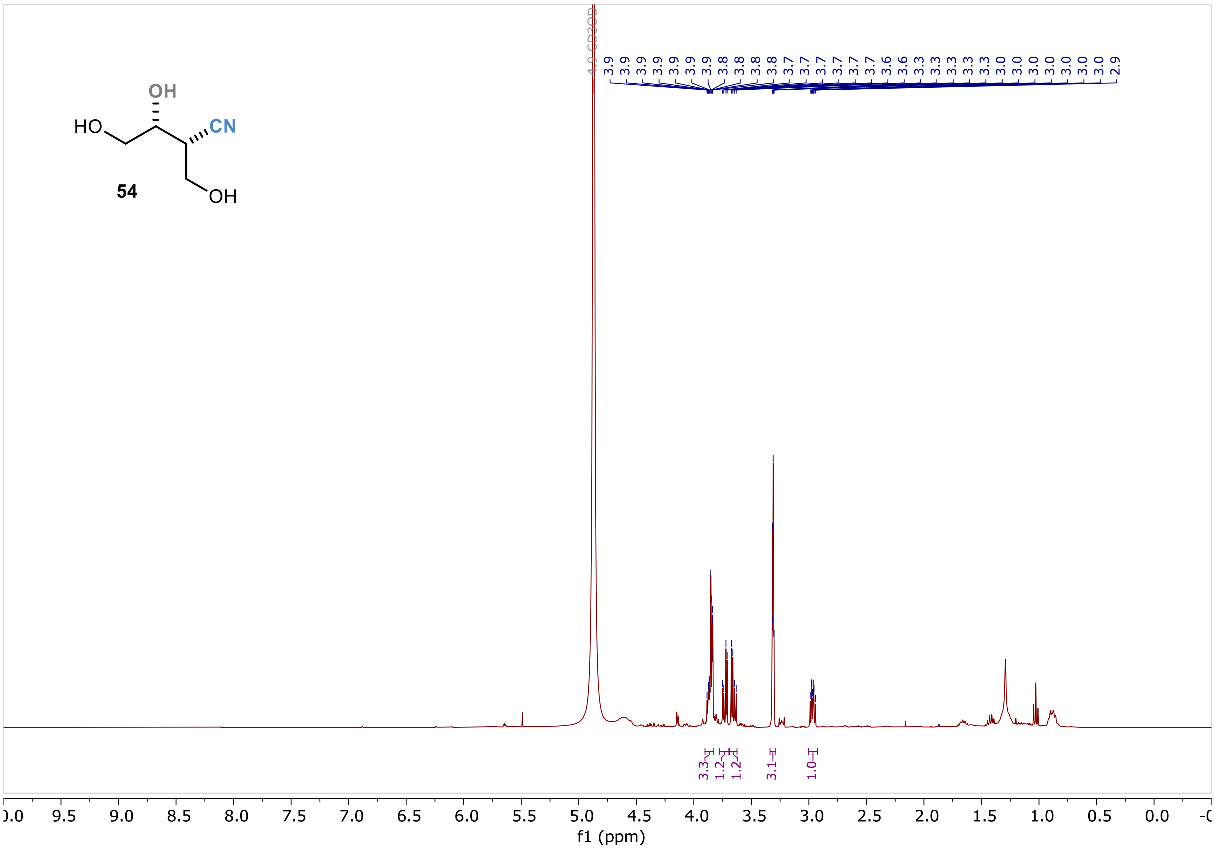

¹³C NMR (101 MHz, CD_3OD) for compound **54**:

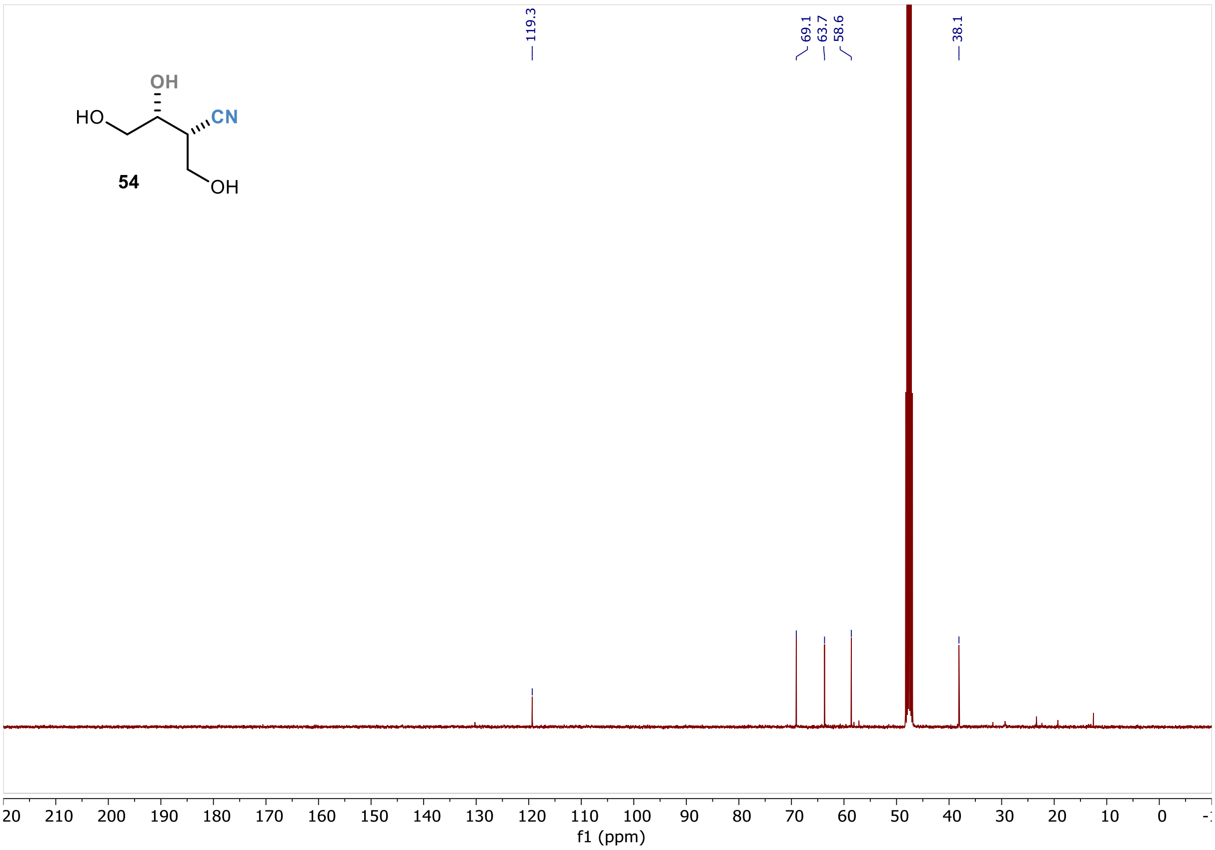

[1]**H NMR** (400 MHz, CD_3OD) for compound **55**:

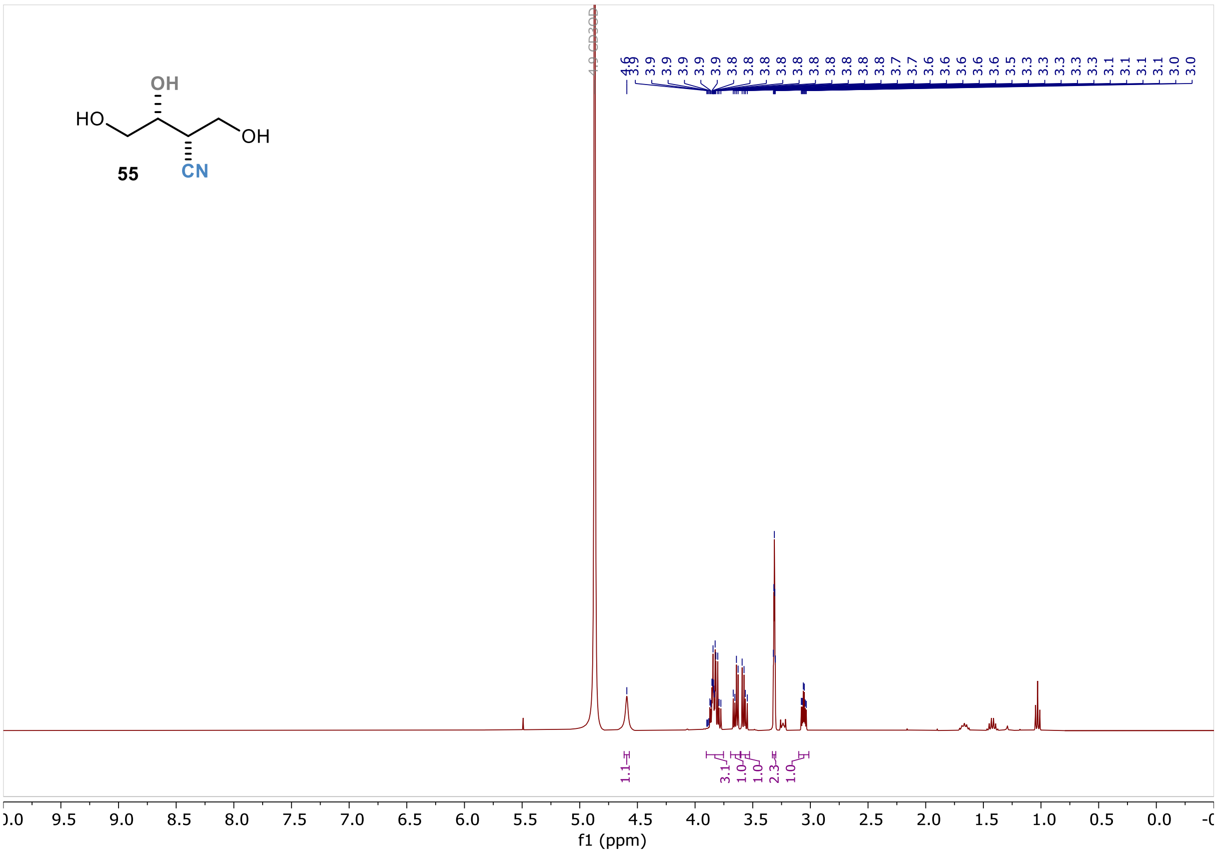

[13]**C NMR** (101 MHz, CD_3OD) for compound **55**:

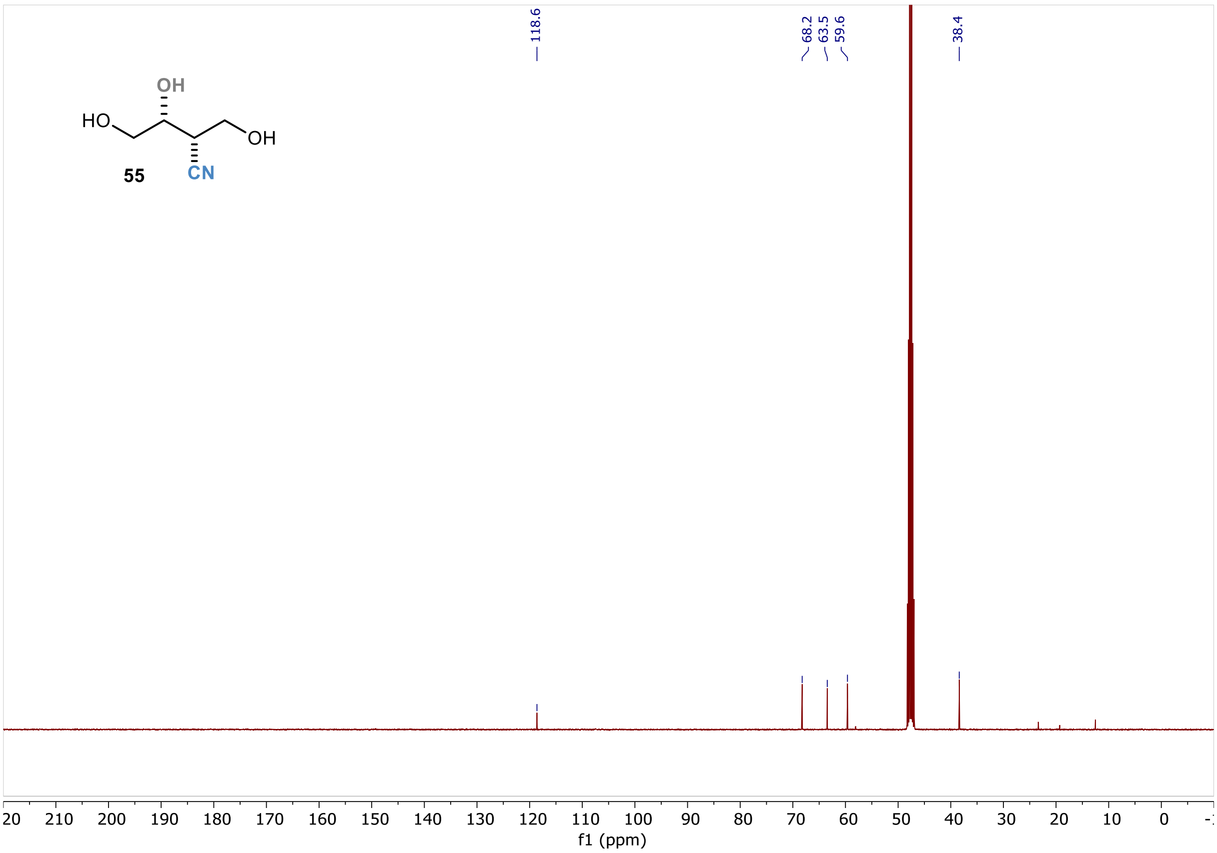

^{1}H NMR (400 MHz, $CDCl_3$) for compound **56**:

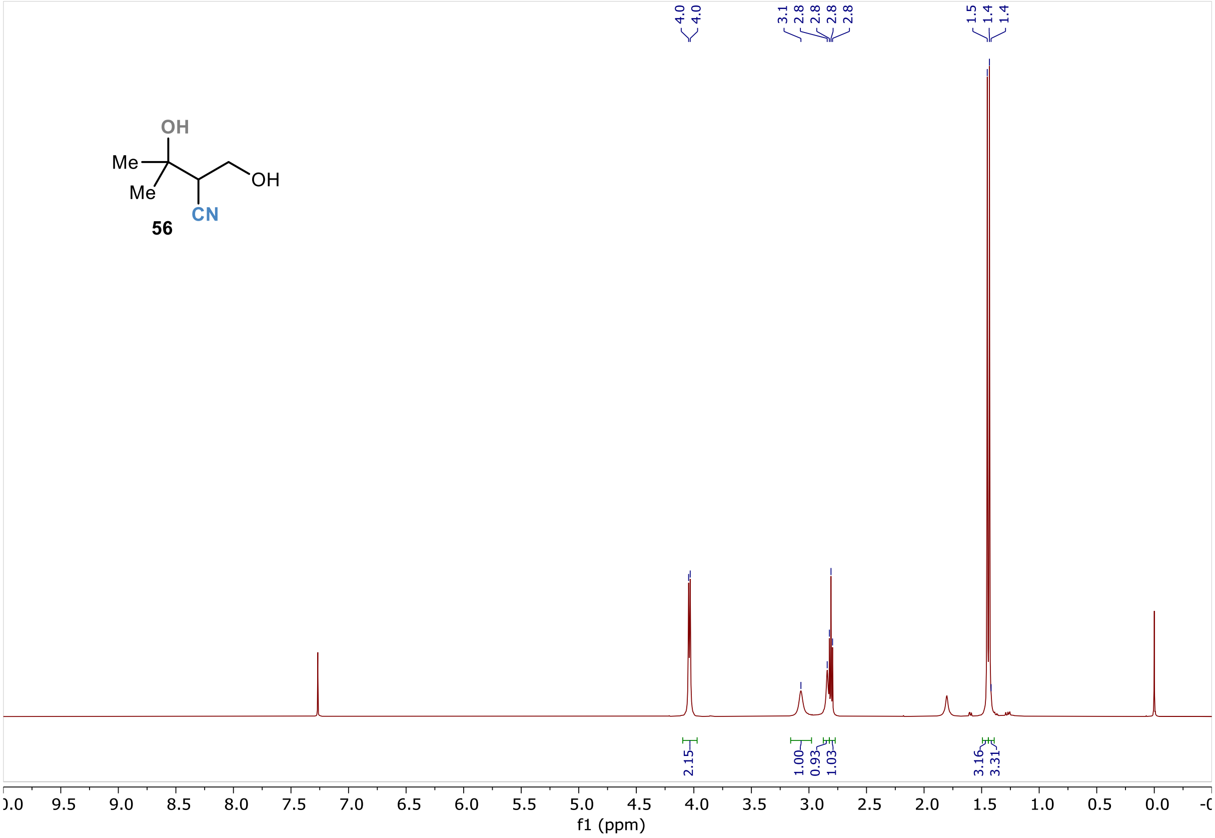

^{13}C NMR (101 MHz, $CDCl_3$) for compound **56**:

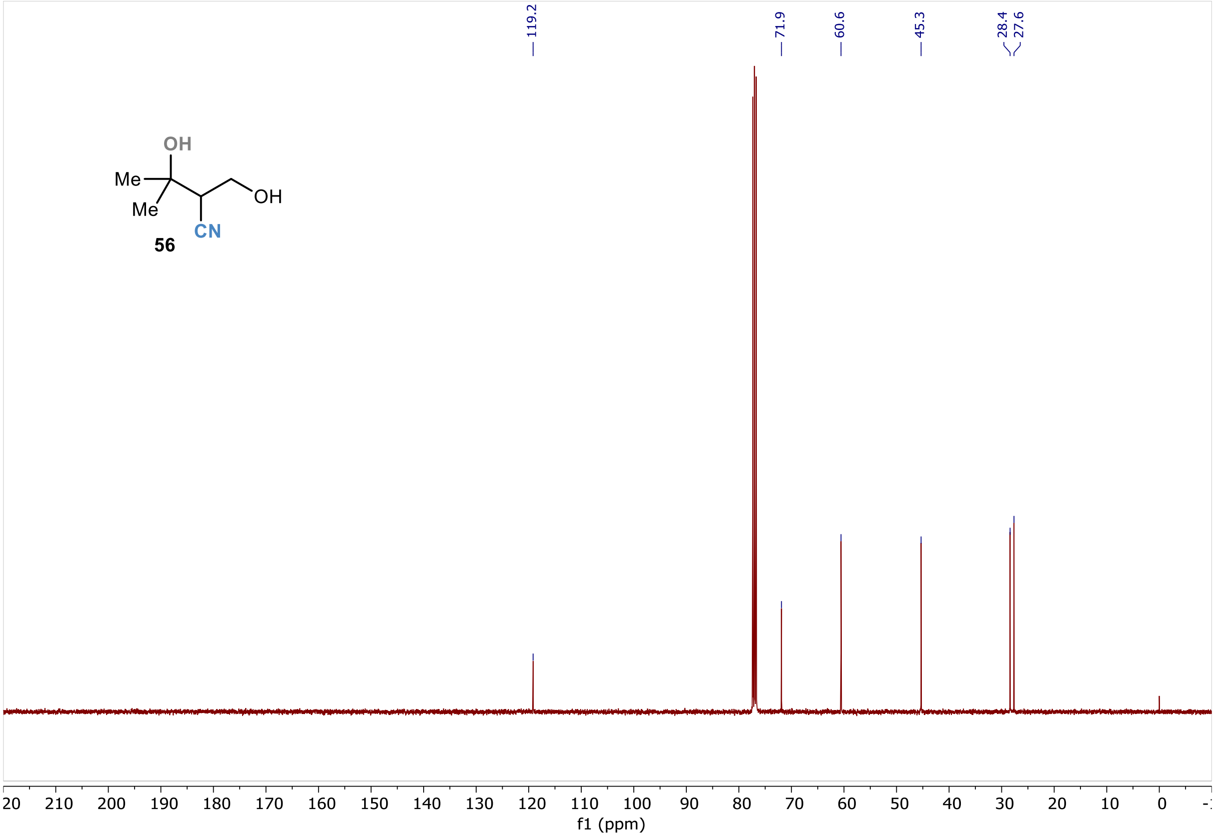

^{1}H NMR (400 MHz, $CDCl_3$) for compound **57-regioisomer 1**:

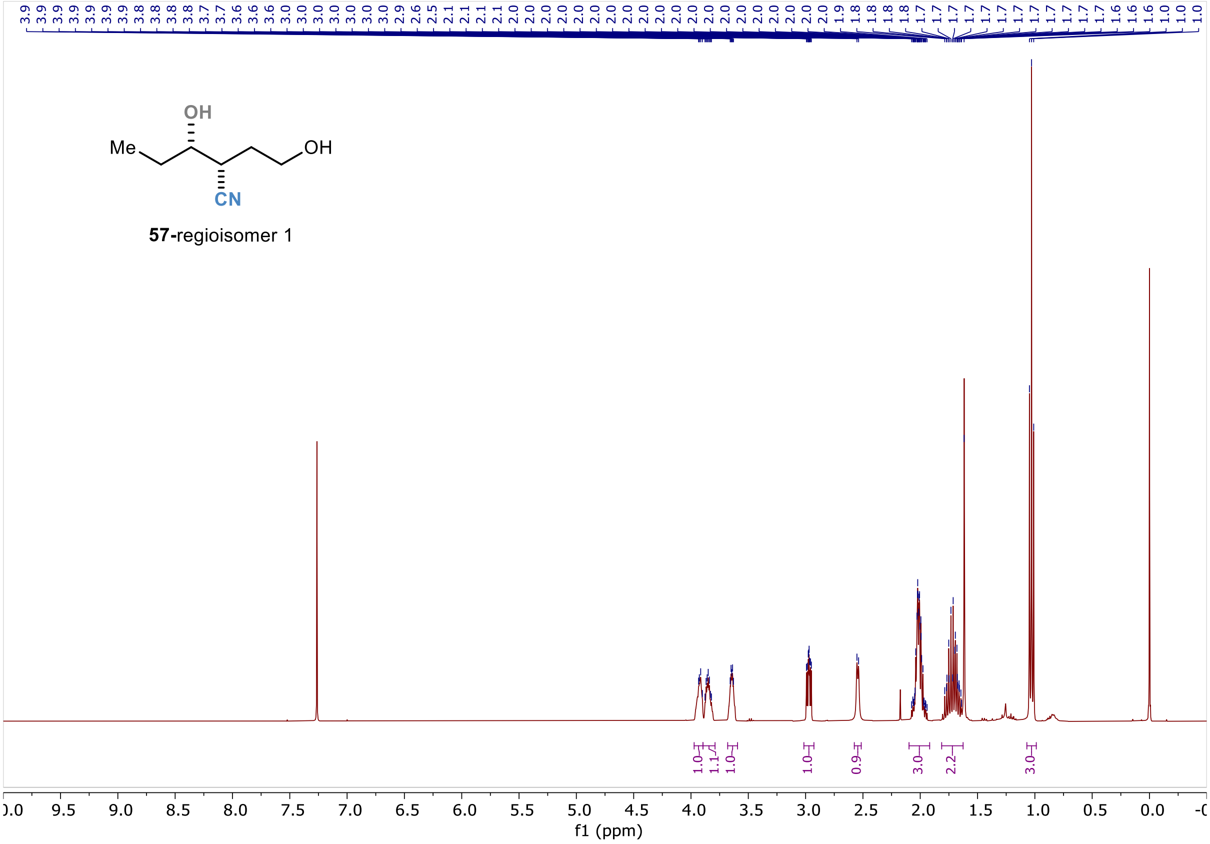

^{13}C NMR (101 MHz, $CDCl_3$) for compound **57-regioisomer 1**:

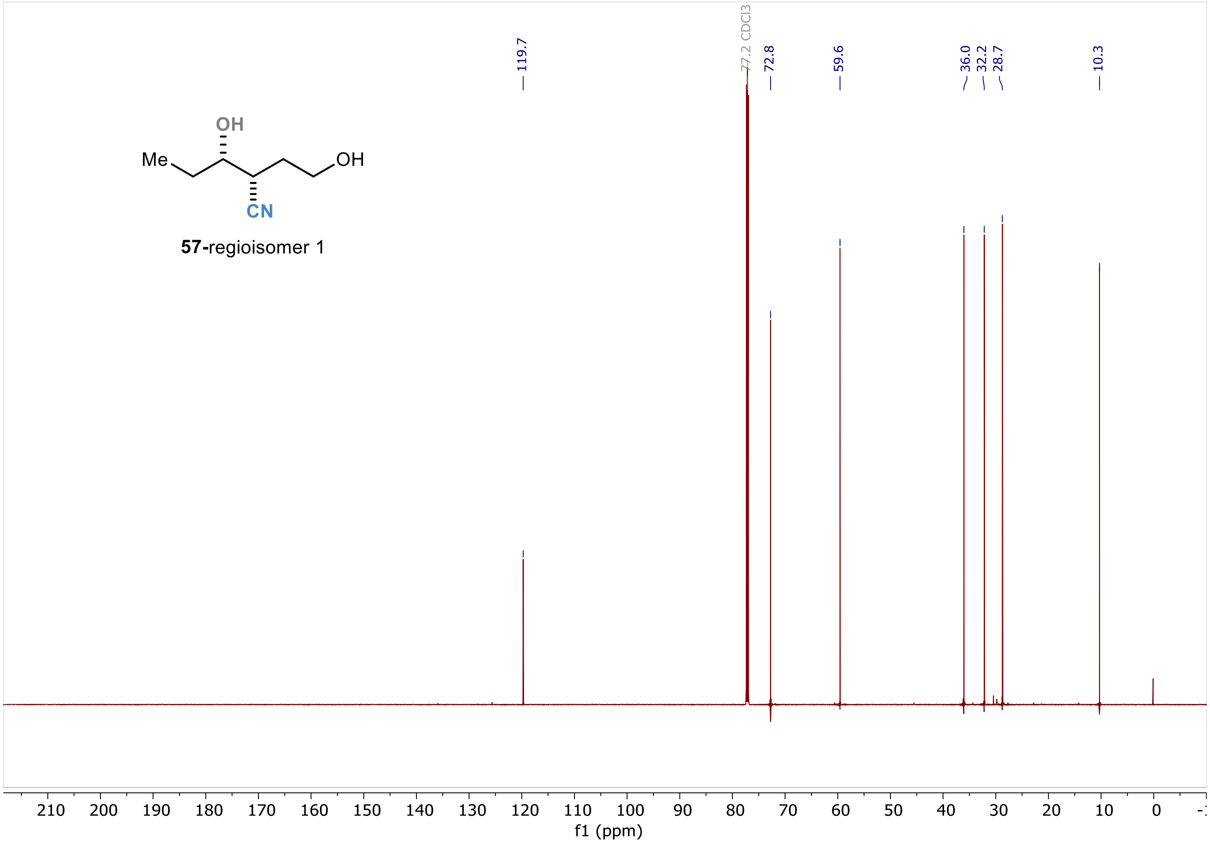

^{1}**H NMR** (400 MHz, $CDCl_3$) for compound **57-regioisomer 2**:

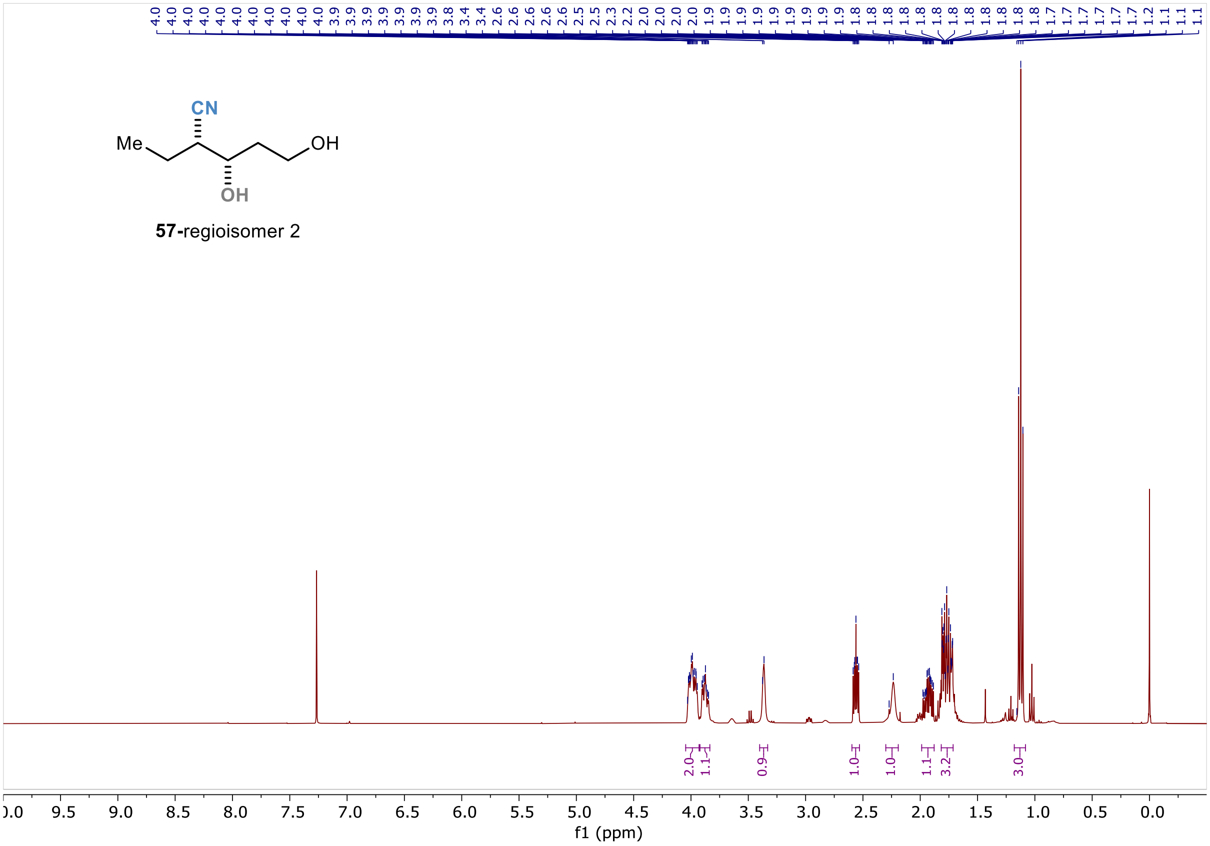

^{13}**C NMR** (101 MHz, $CDCl_3$) for compound **57-regioisomer 2**:

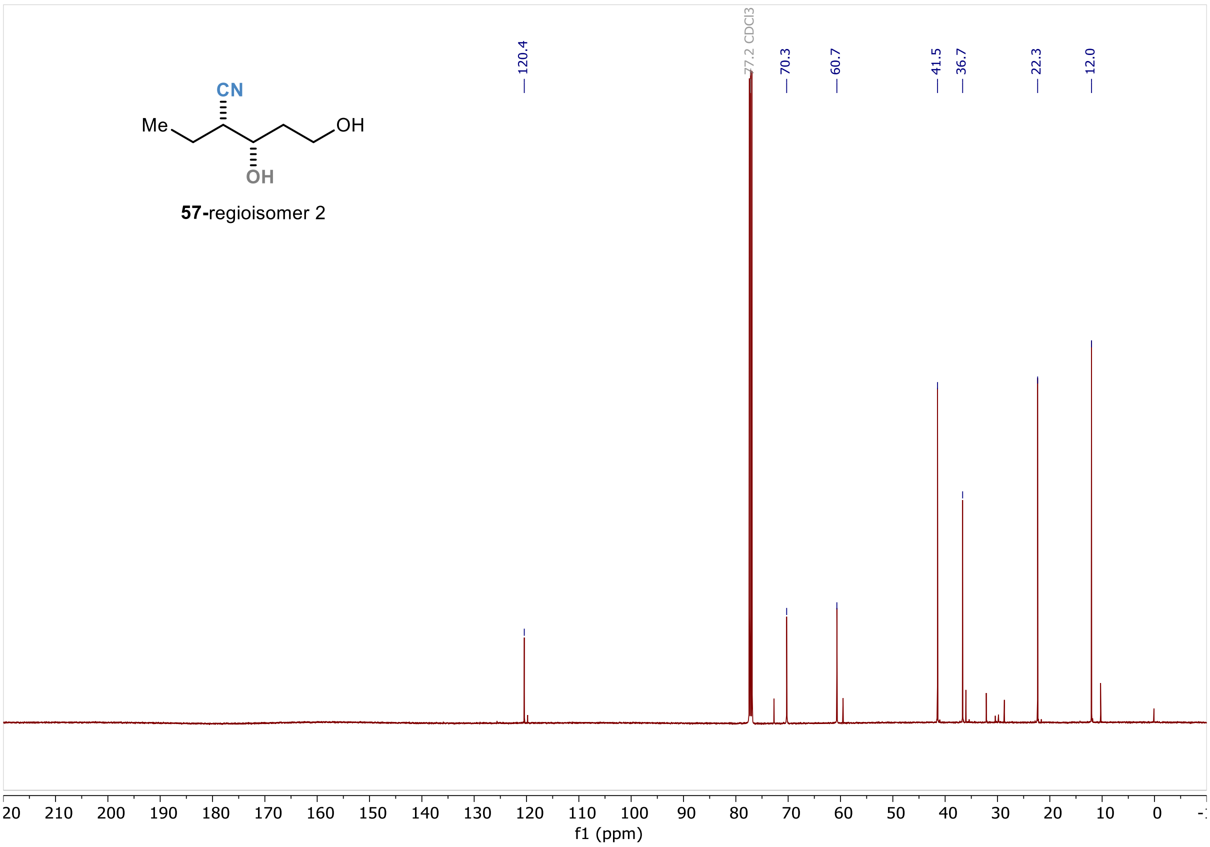

¹H NMR (400 MHz, $CDCl_3$) for compound **58**:

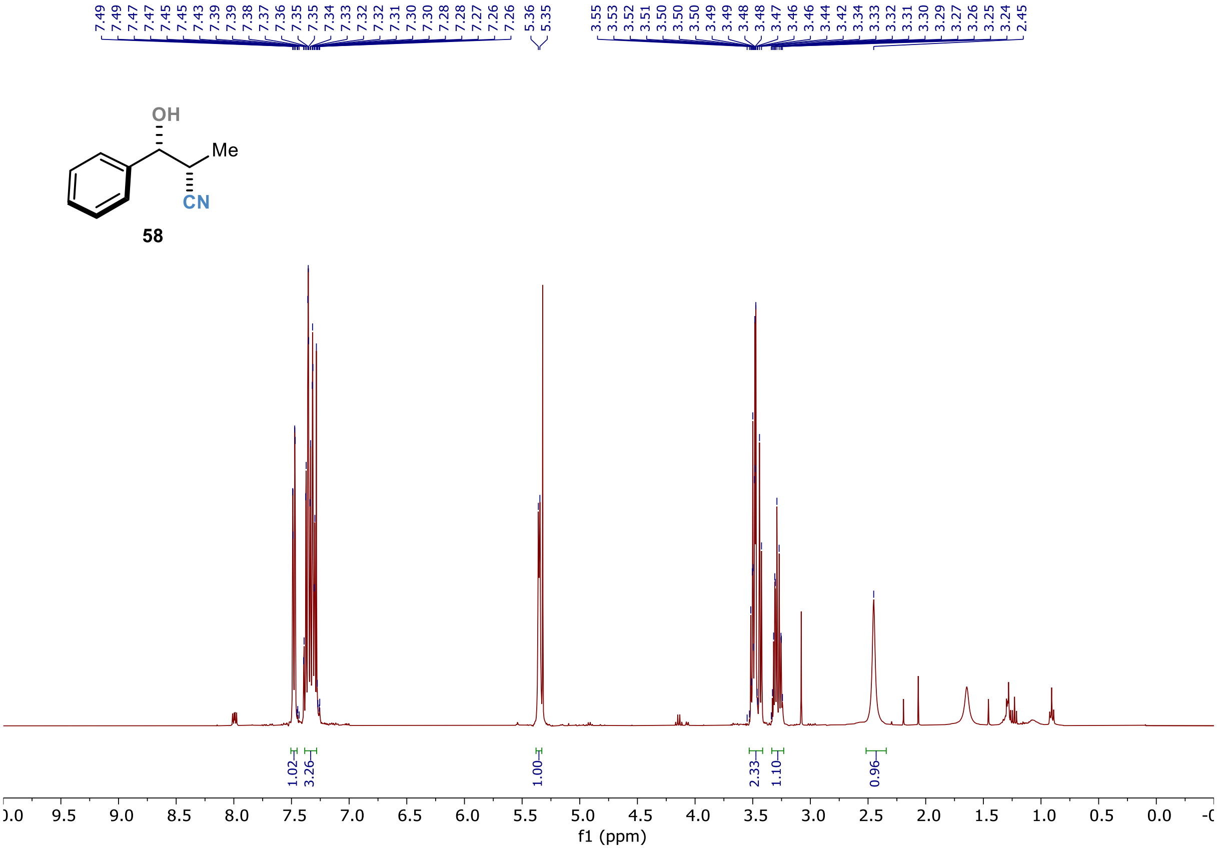

¹³C NMR (101 MHz, $CDCl_3$) for compound **58**:

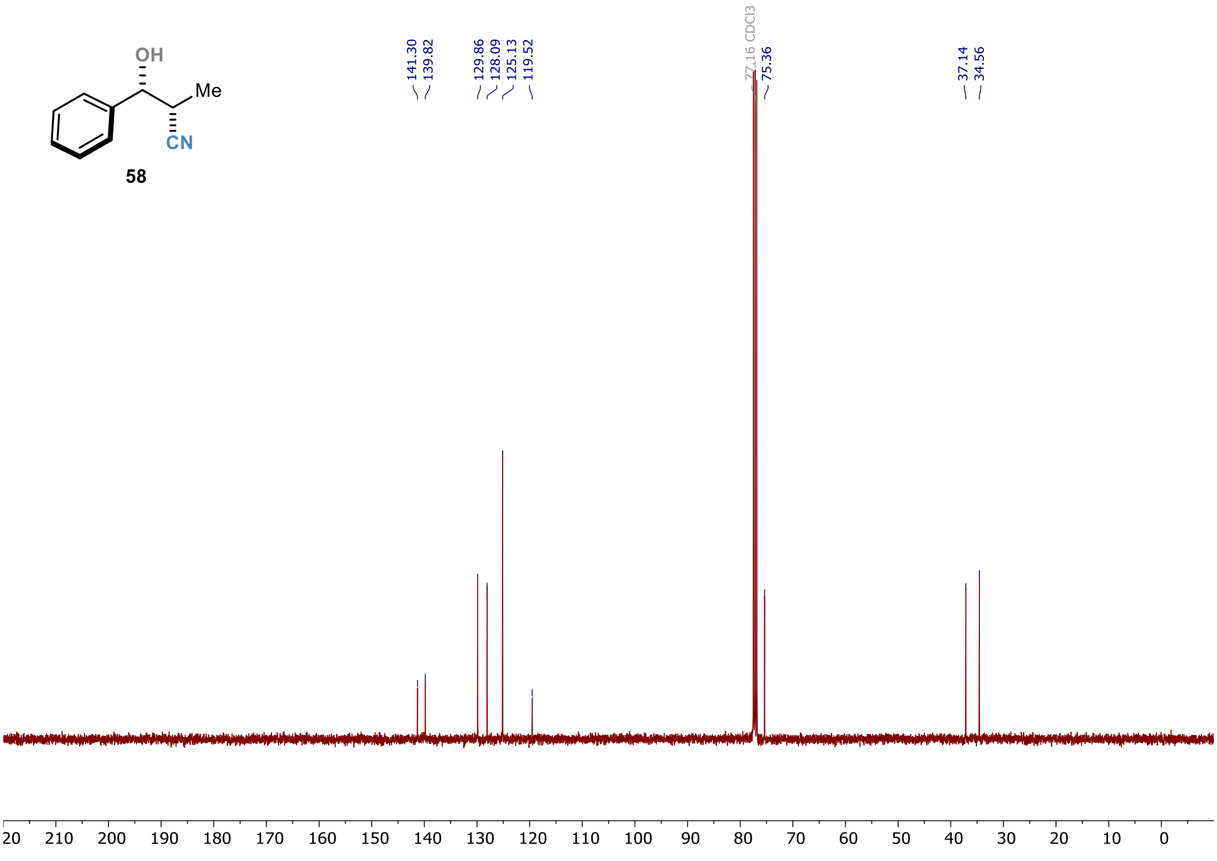

^{1}H NMR (400 MHz, $CDCl_3$) for compound **59**:

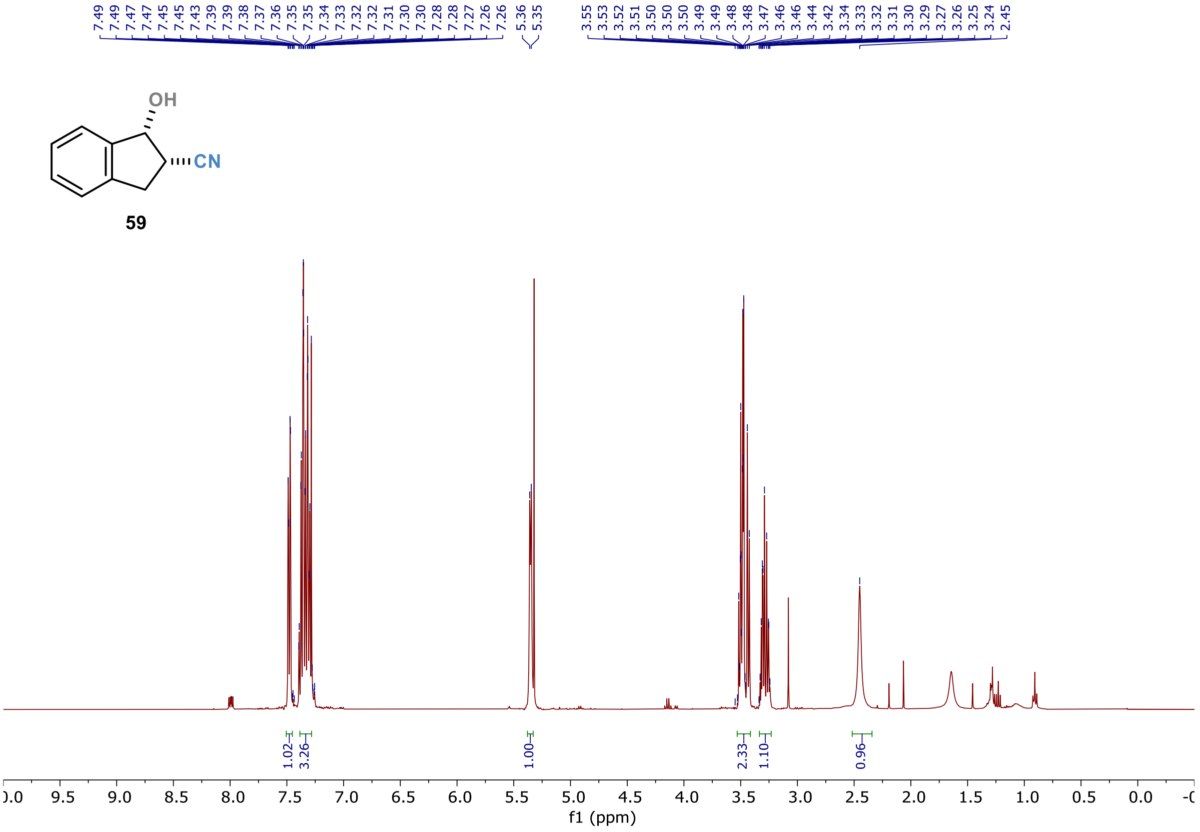

^{13}C NMR (101 MHz, $CDCl_3$) for compound **59**:

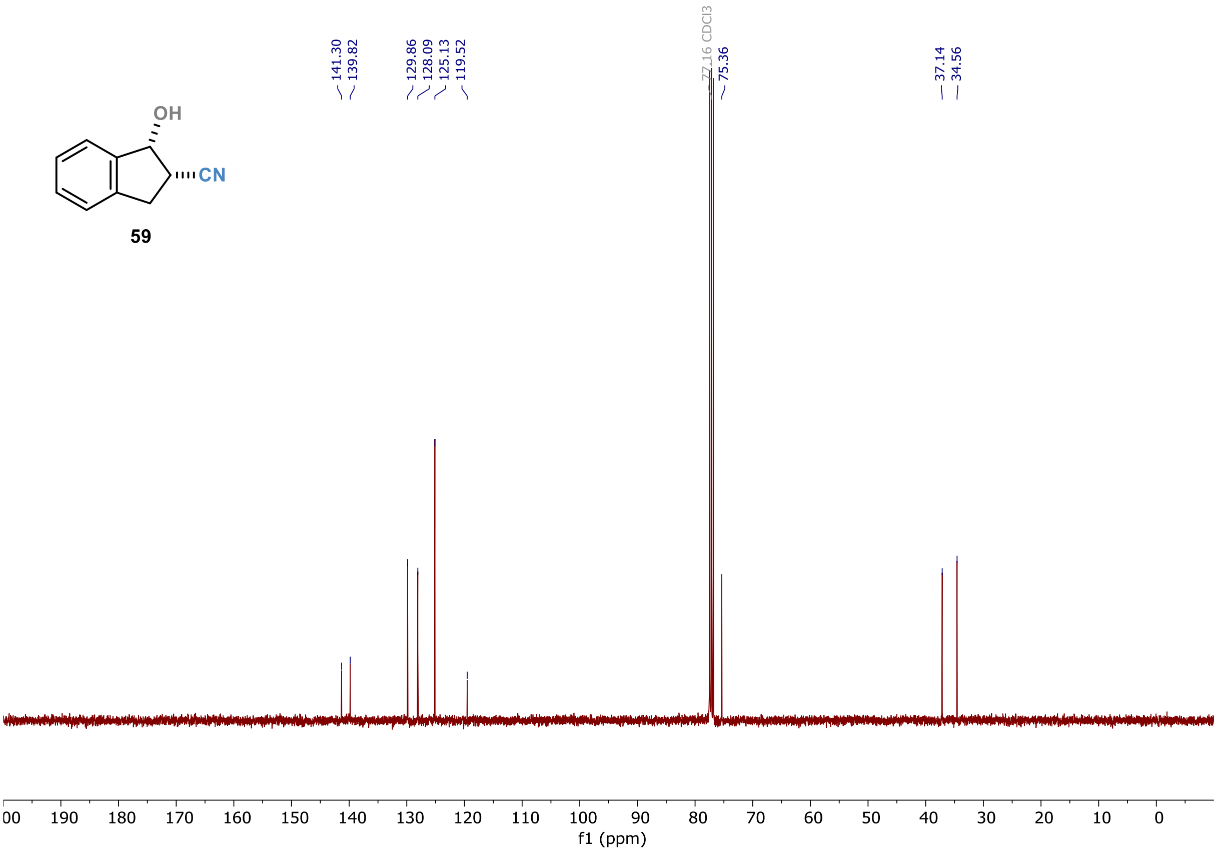

¹H NMR (500 MHz, $CDCl_3$) for compound **60**:

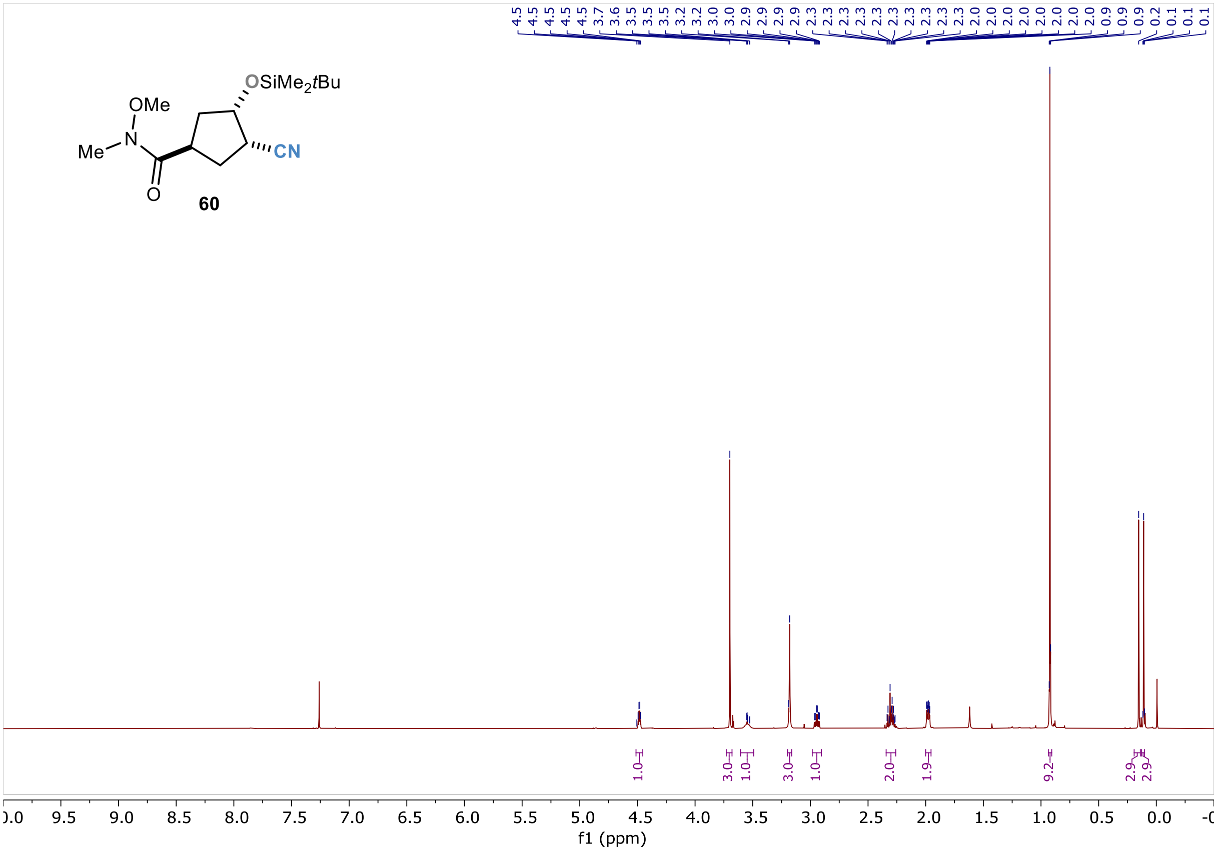

¹³C NMR (126 MHz, $CDCl_3$) for compound **60**:

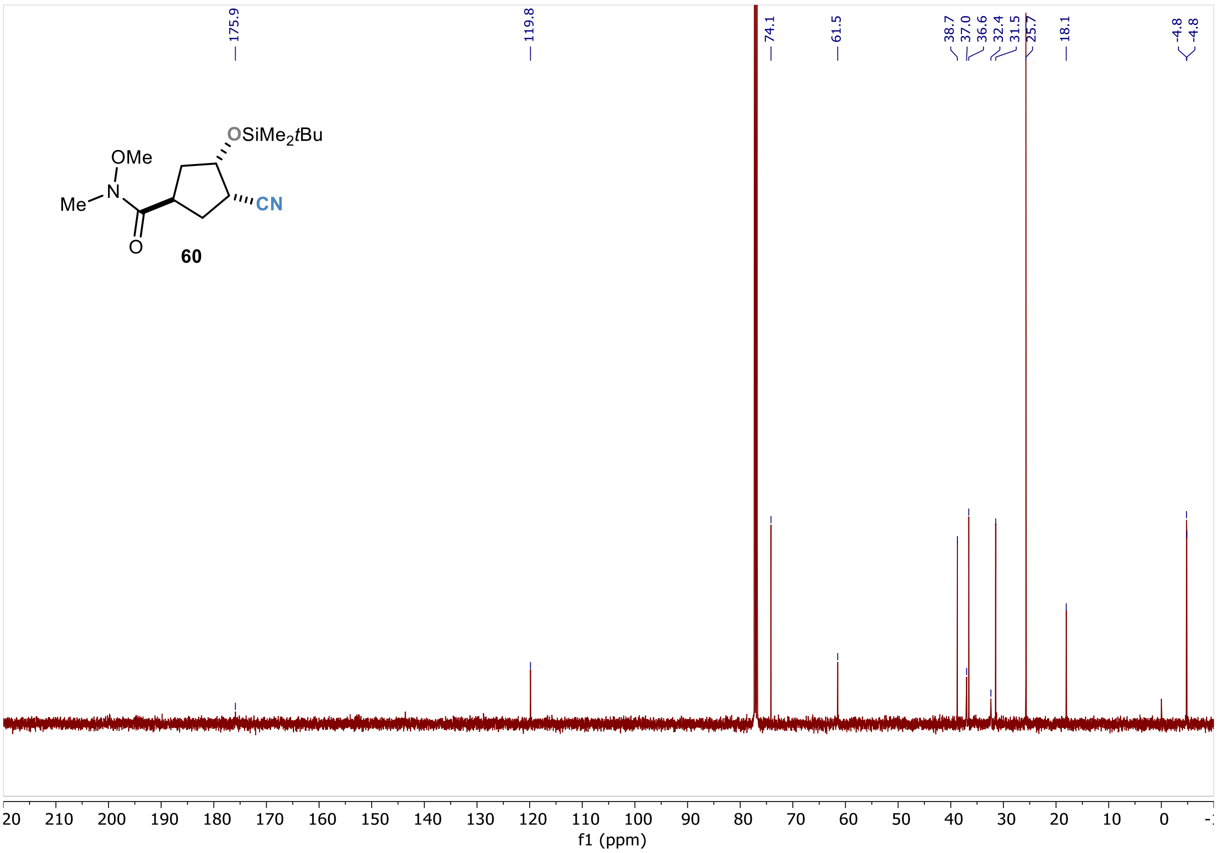

^{1}H NMR (400 MHz, $CDCl_3$) for compound **61**:

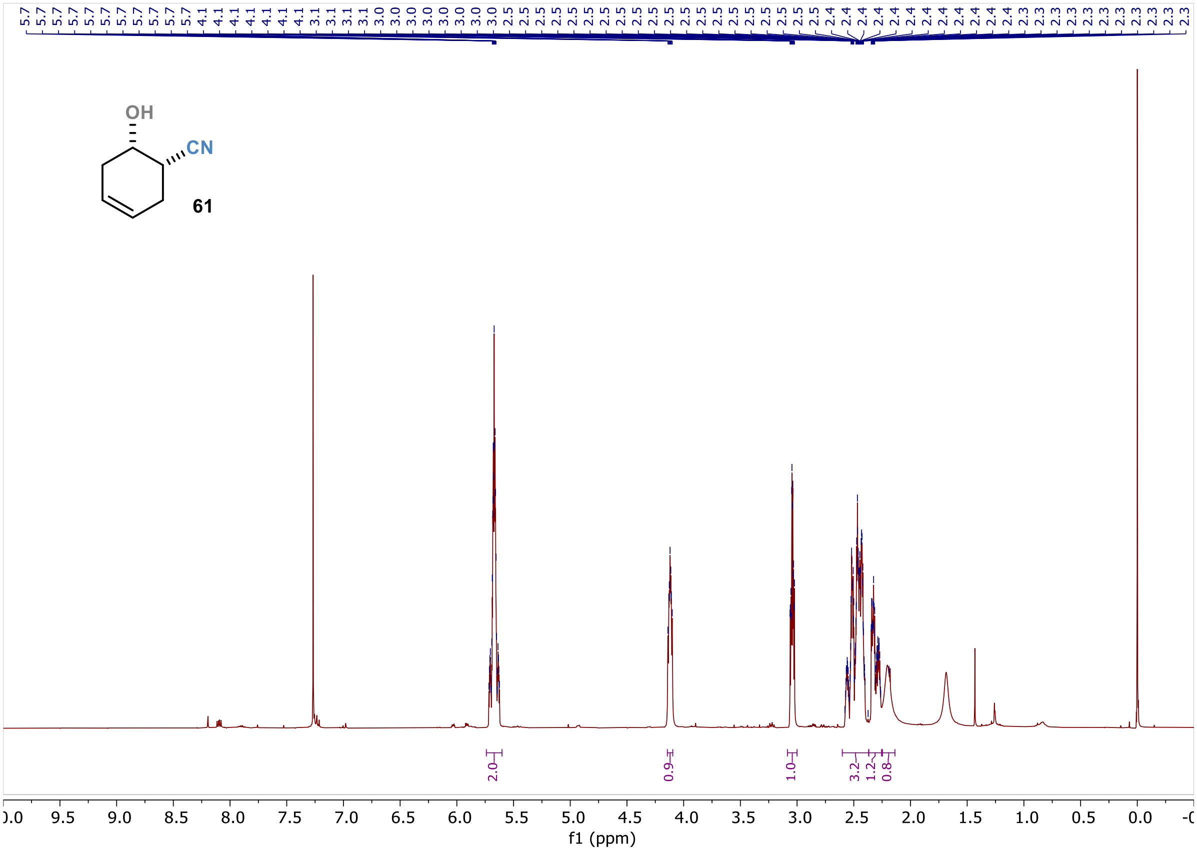

^{13}C NMR (101 MHz, $CDCl_3$) for compound **61**:

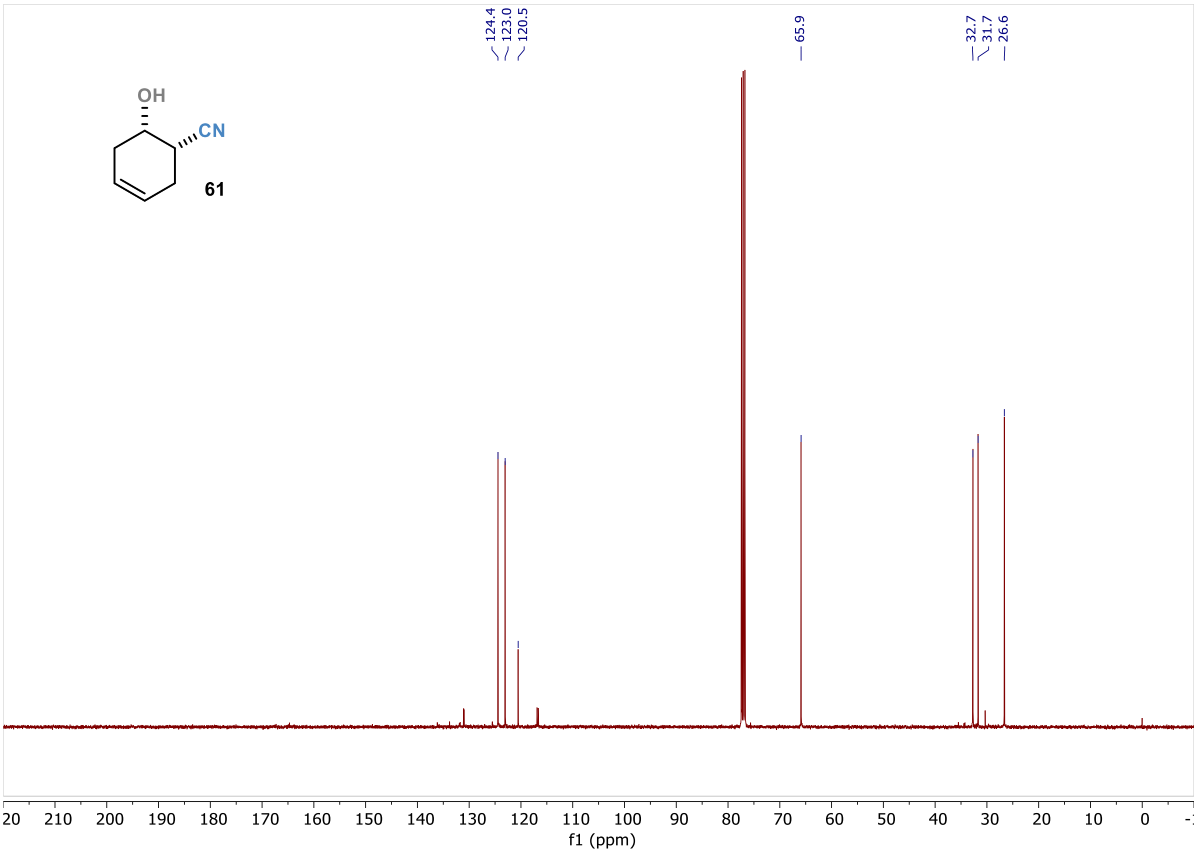

¹H NMR (400 MHz, $CDCl_3$) for compound **1,2-*syn*-1,5-*anti*-62**:

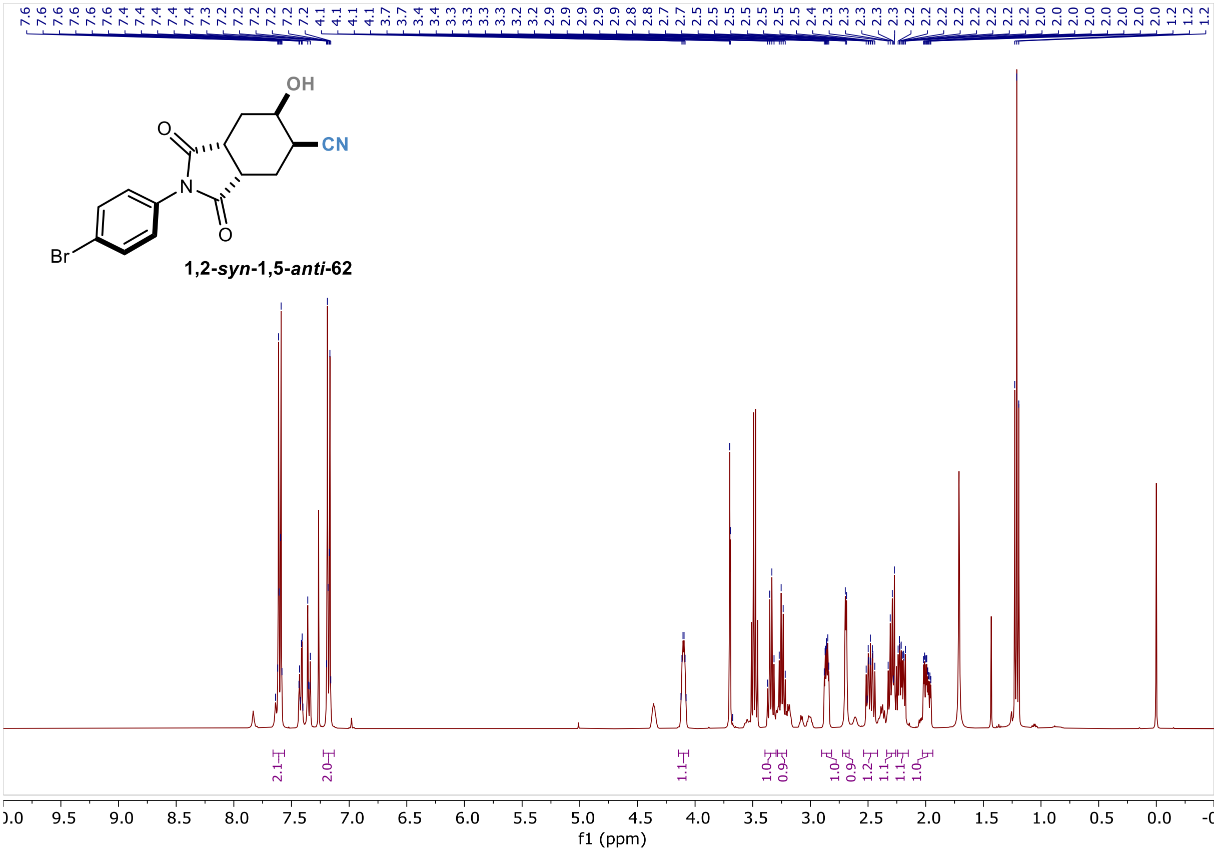

¹³C NMR (101 MHz, $CDCl_3$) for compound **1,2-*syn*-1,5-*anti*-62**:

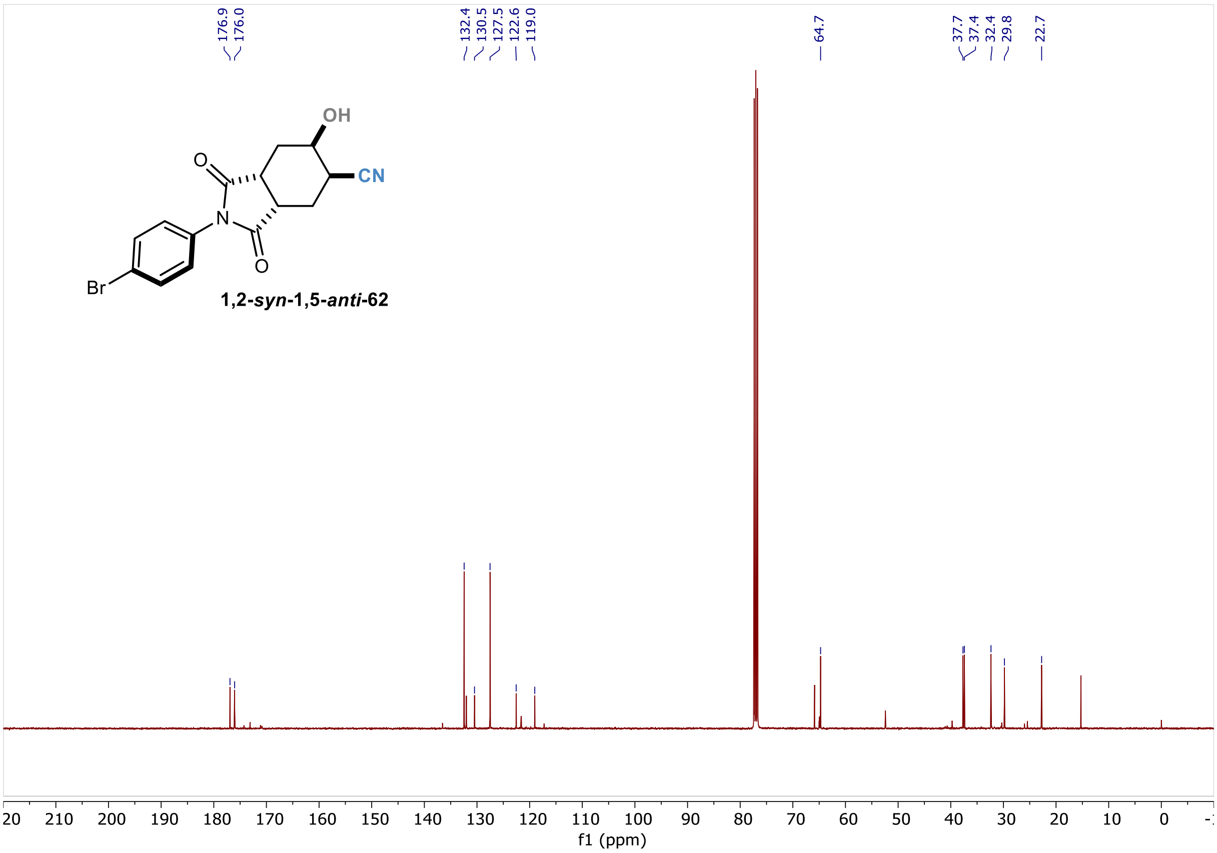

^{1}H NMR (400 MHz, $CDCl_3$) for compound ***1,2-syn*-1,5-*syn*-62**:

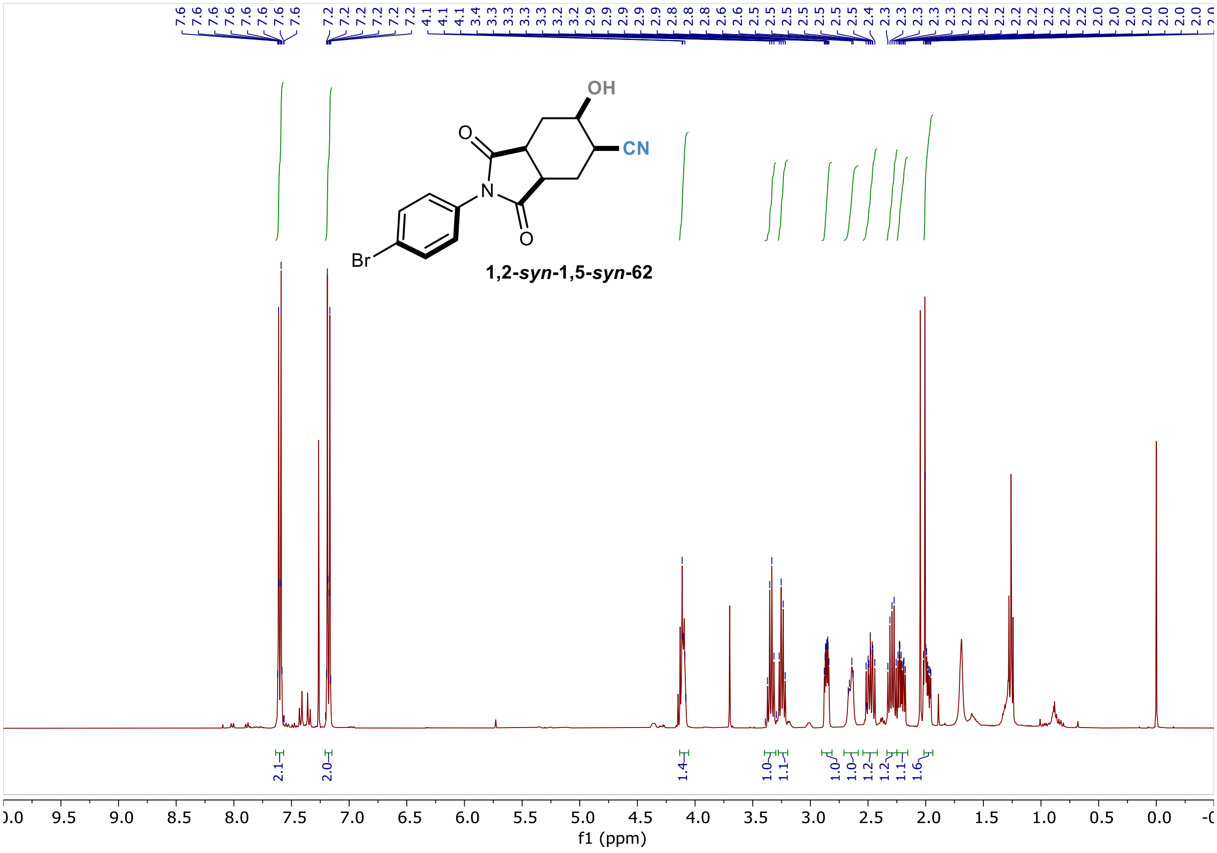

^{13}C NMR (101 MHz, $CDCl_3$) for compound ***1,2-syn*-1,5-*syn*-62**:

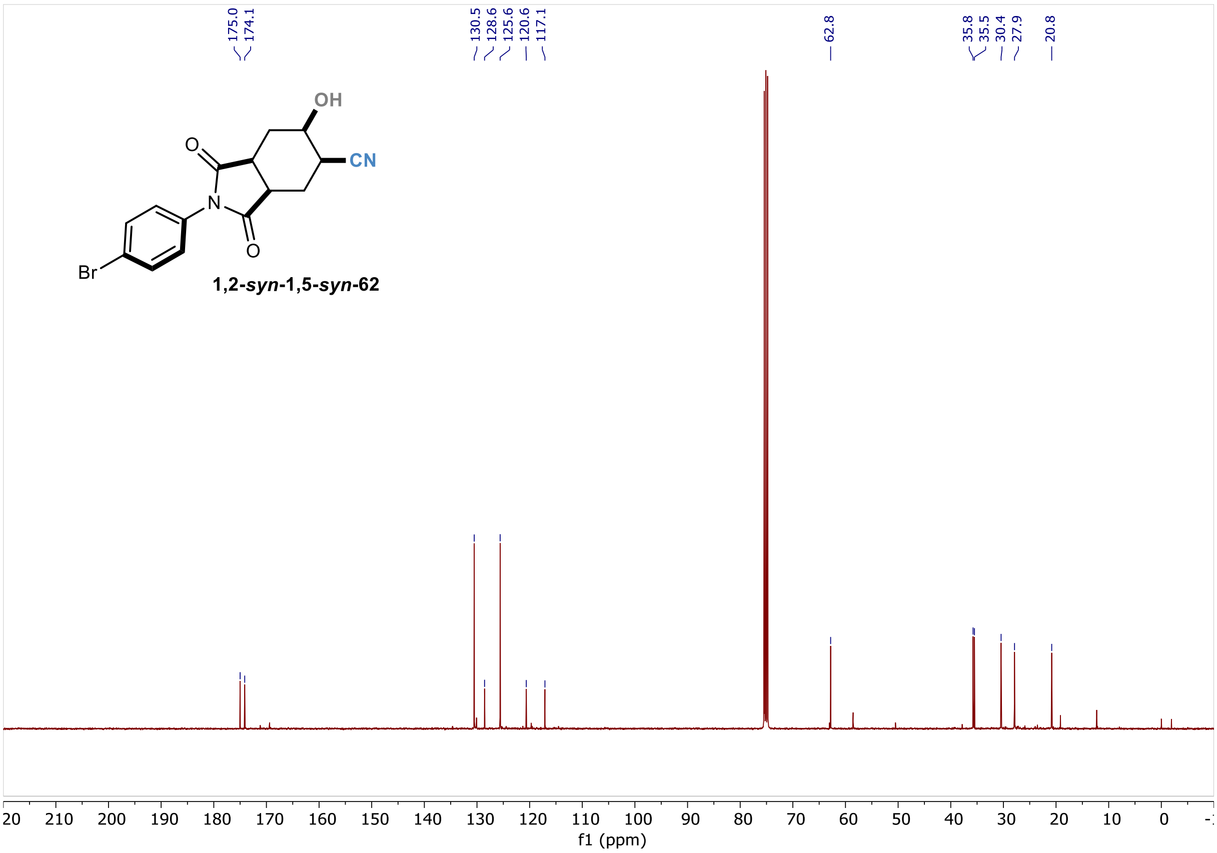

¹H NMR (400 MHz, $CDCl_3$) for compound **63**:

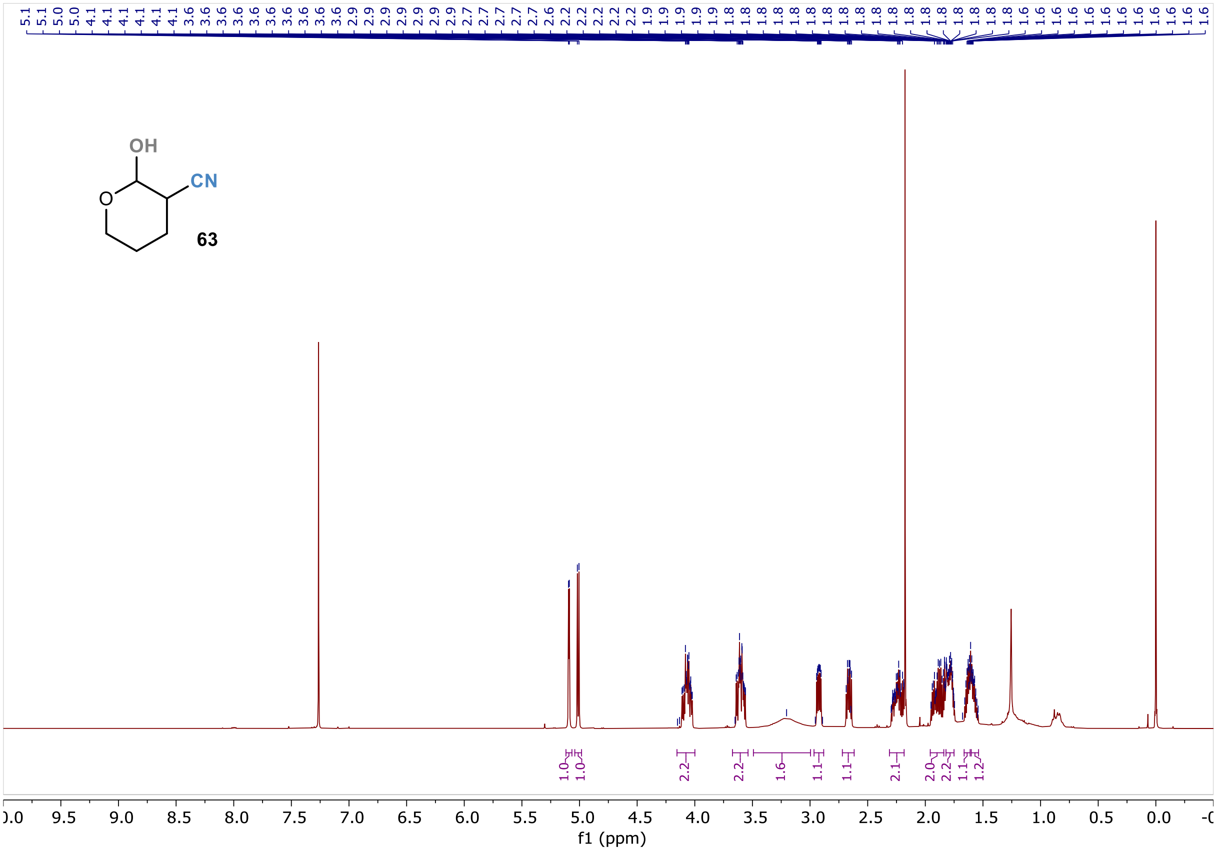

¹³C NMR (101 MHz, $CDCl_3$) for compound **63**:

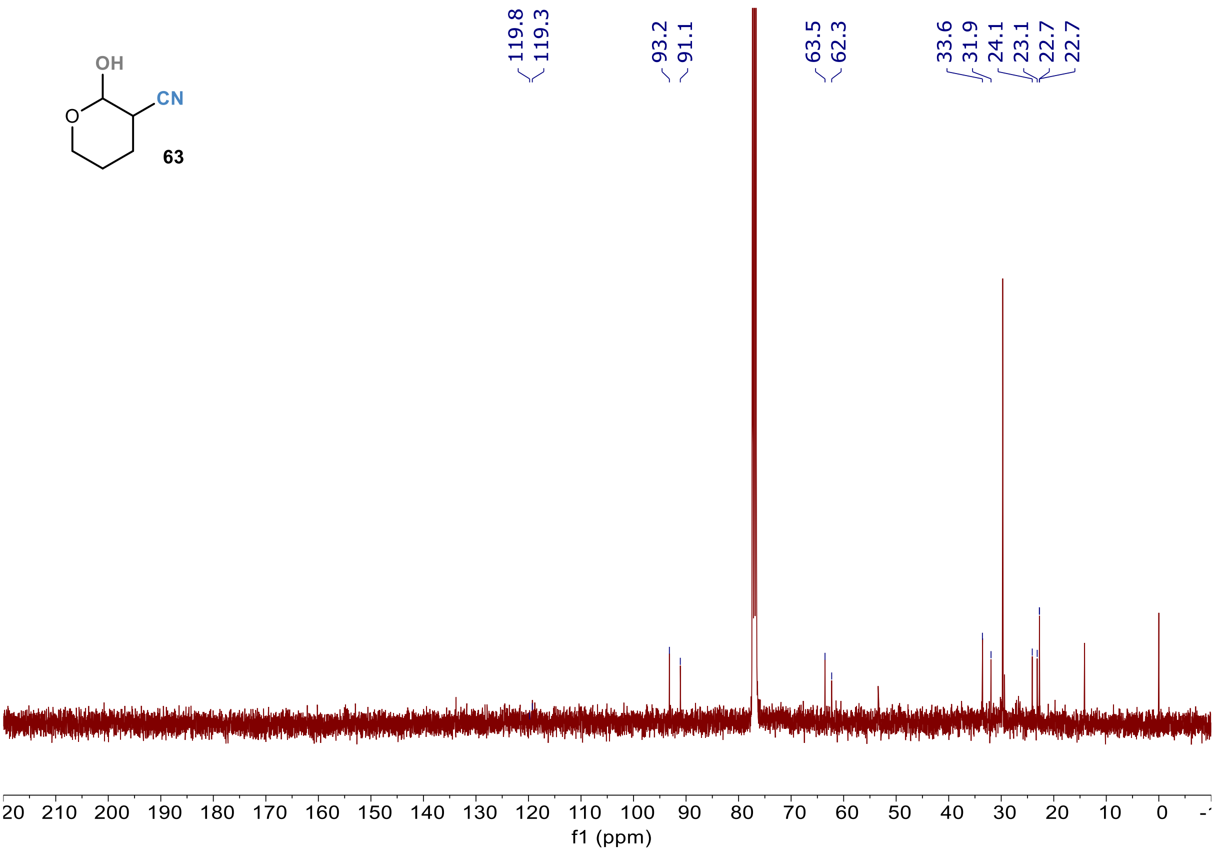

^{1}H NMR (400 MHz, $CDCl_3$) for compound **64**:

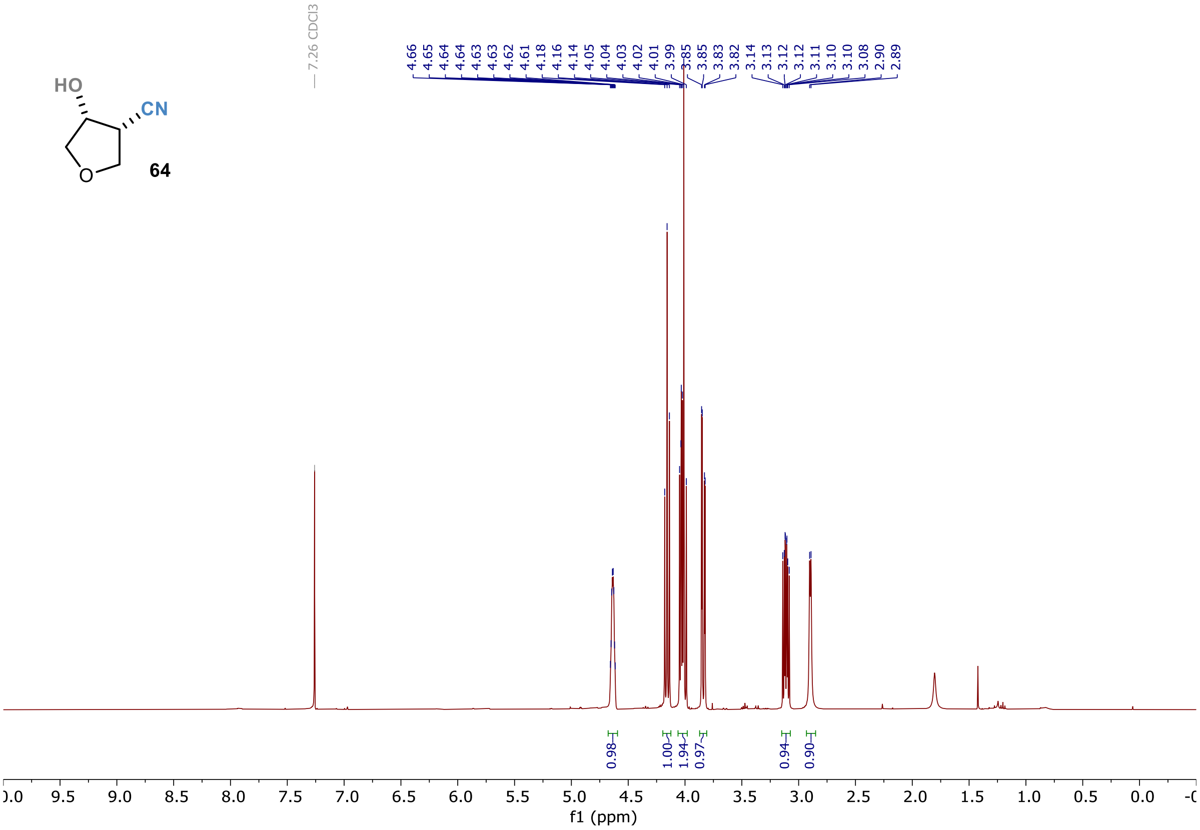

^{13}C NMR (101 MHz, $CDCl_3$) for compound **64**:

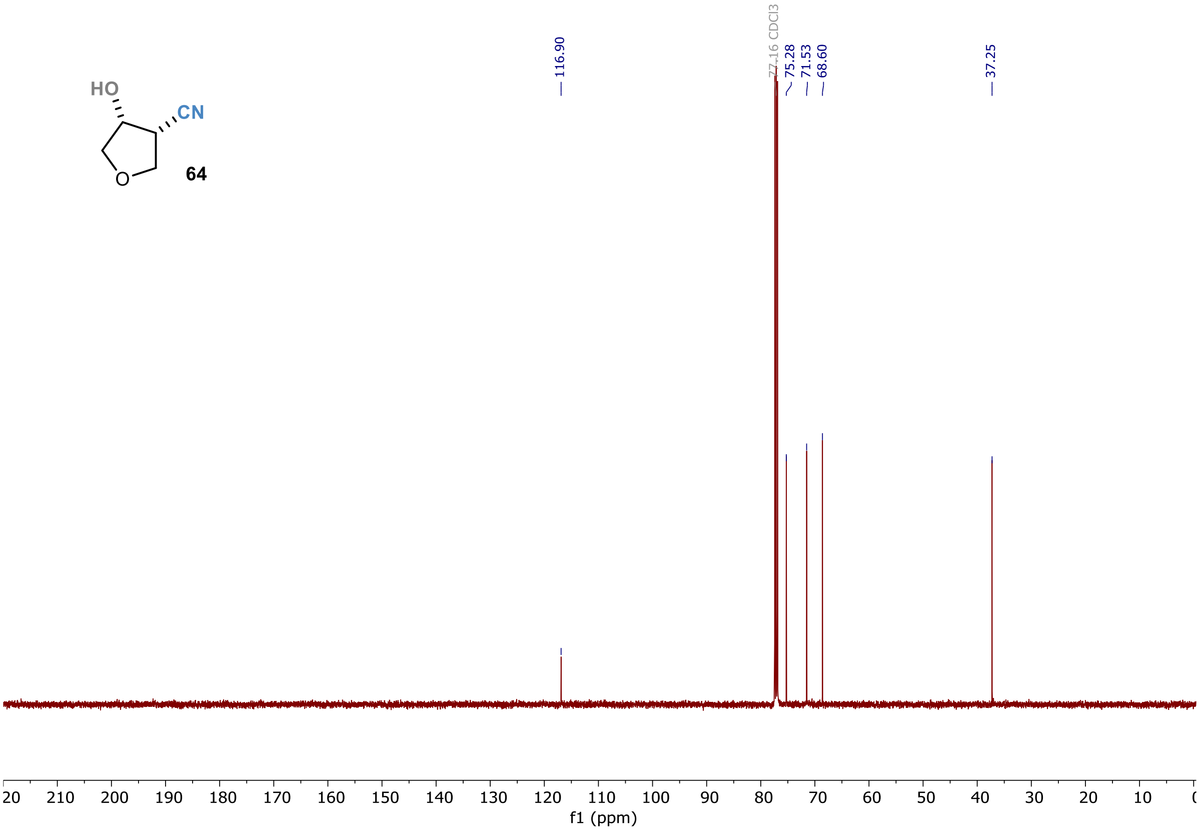

^{1}H NMR (500 MHz, $CDCl_3$) for compound **65**:

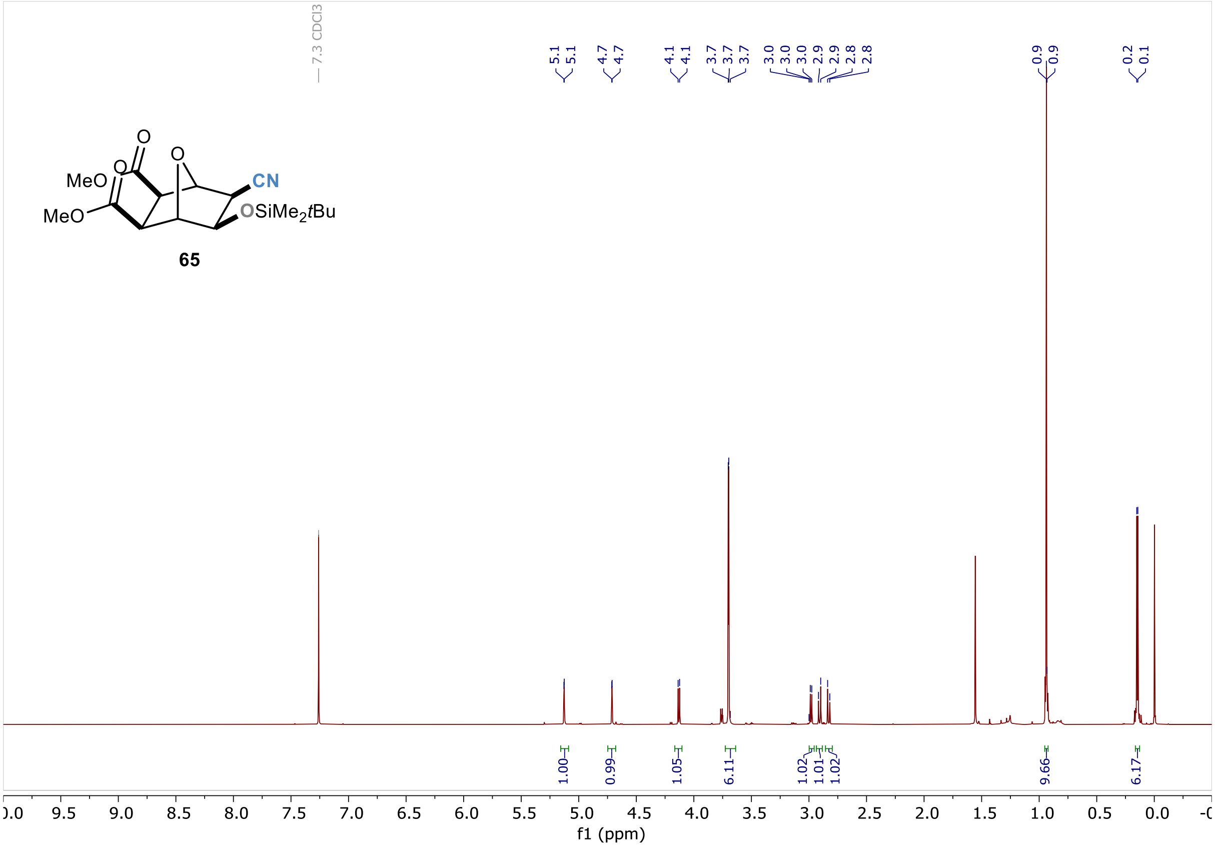

^{13}C NMR (126 MHz, $CDCl_3$) for compound **65**:

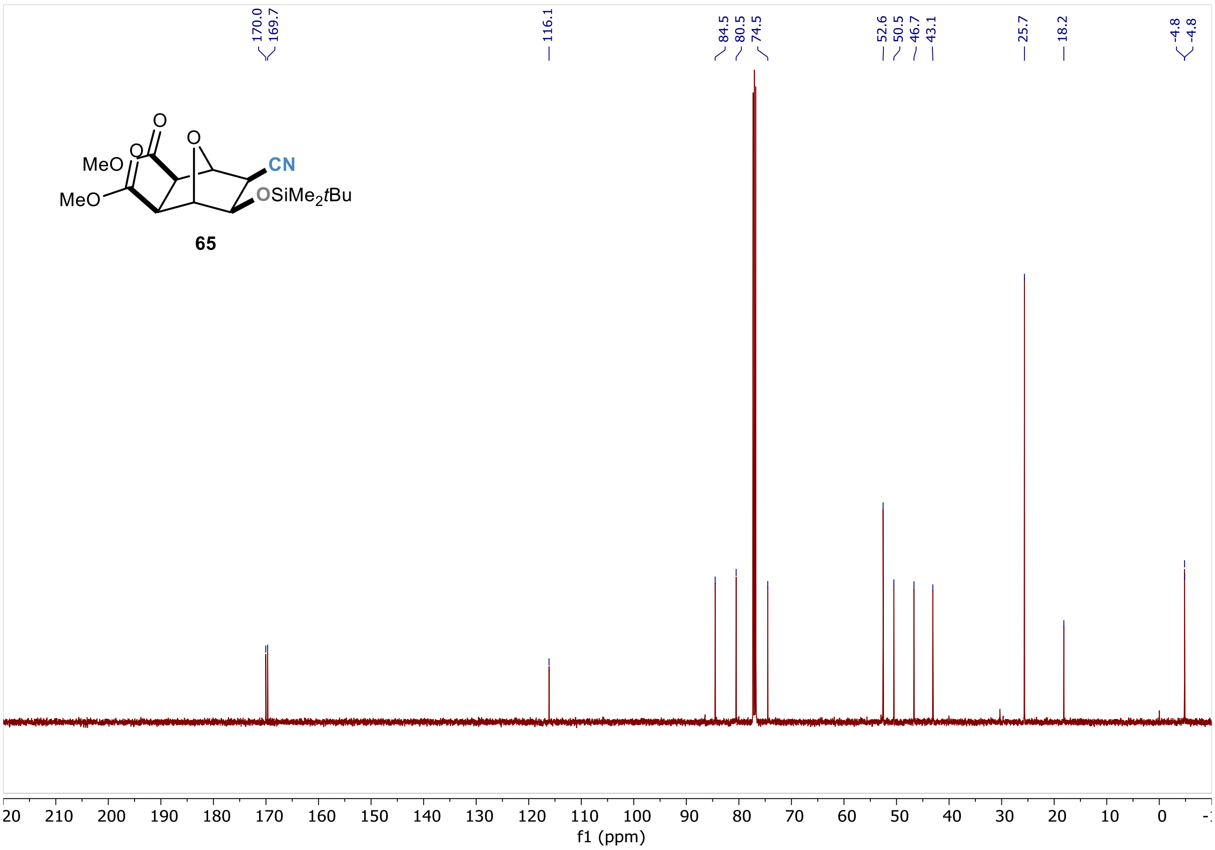

¹H NMR (400 MHz, $CDCl_3$) for compound **66**:

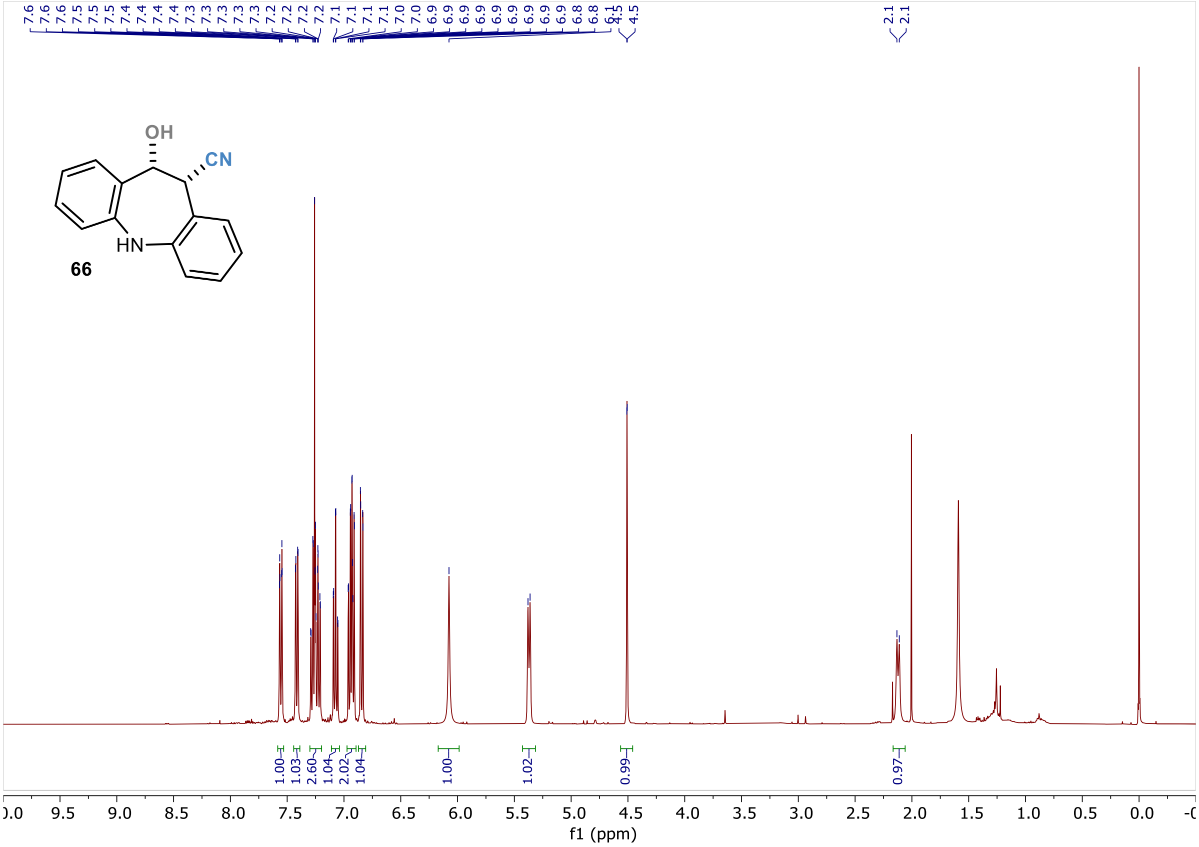

¹³C NMR (101 MHz, $CDCl_3$) for compound **66**:

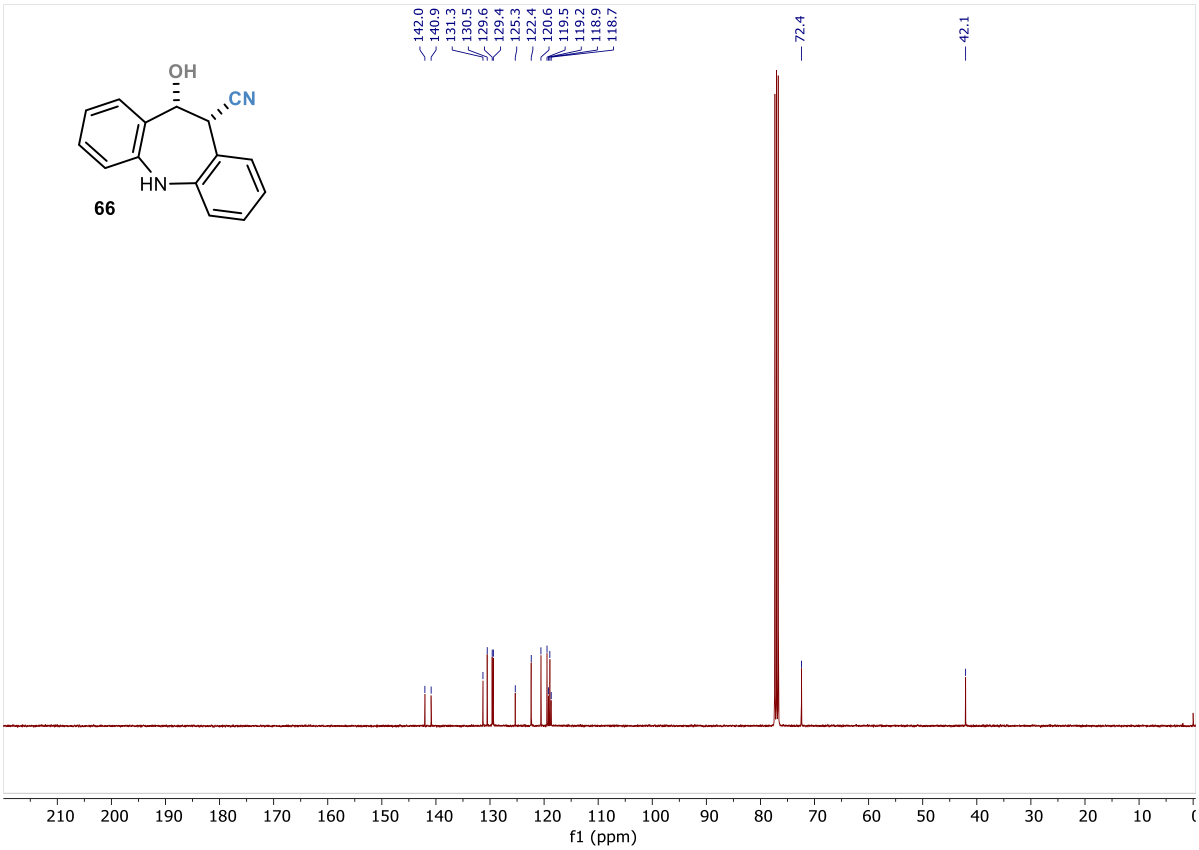

¹H NMR (400 MHz, $CDCl_3$) for compound **67**:

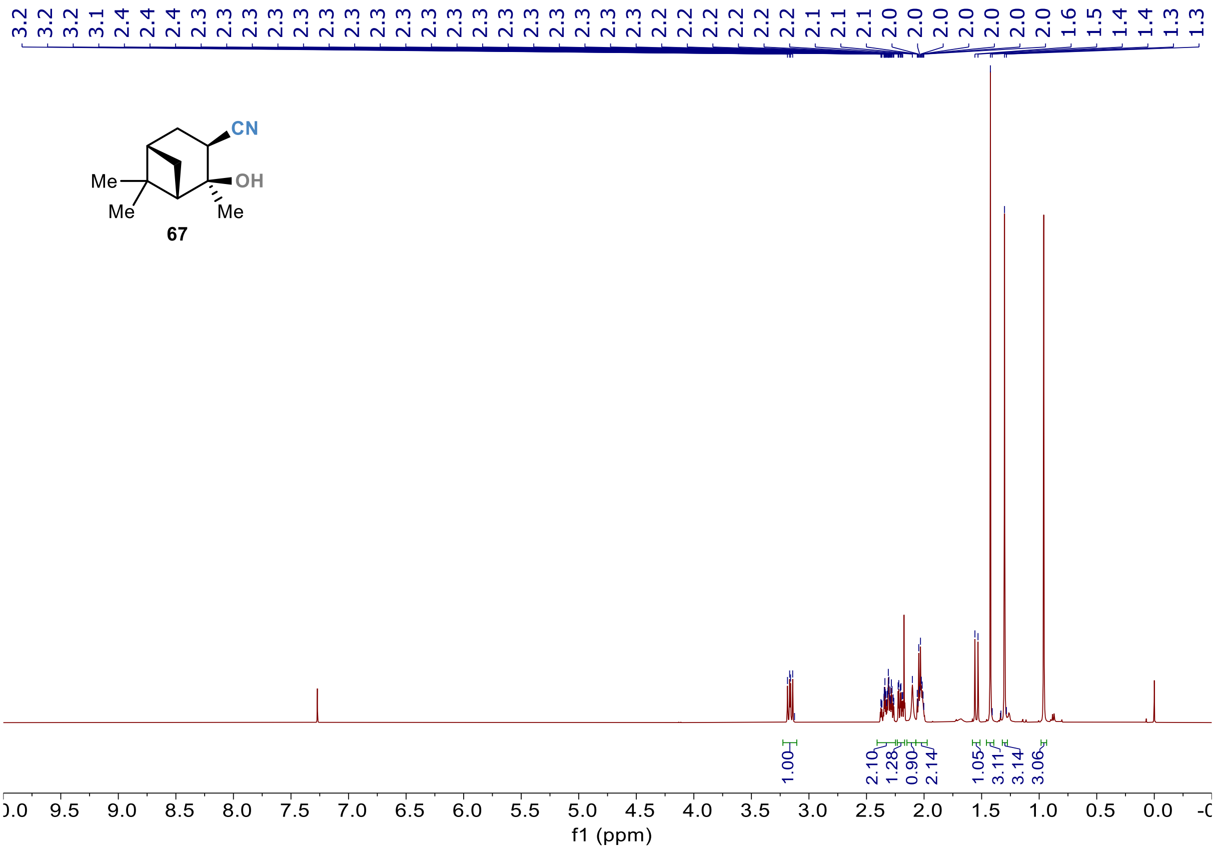

¹³C NMR (101 MHz, $CDCl_3$) for compound **67**:

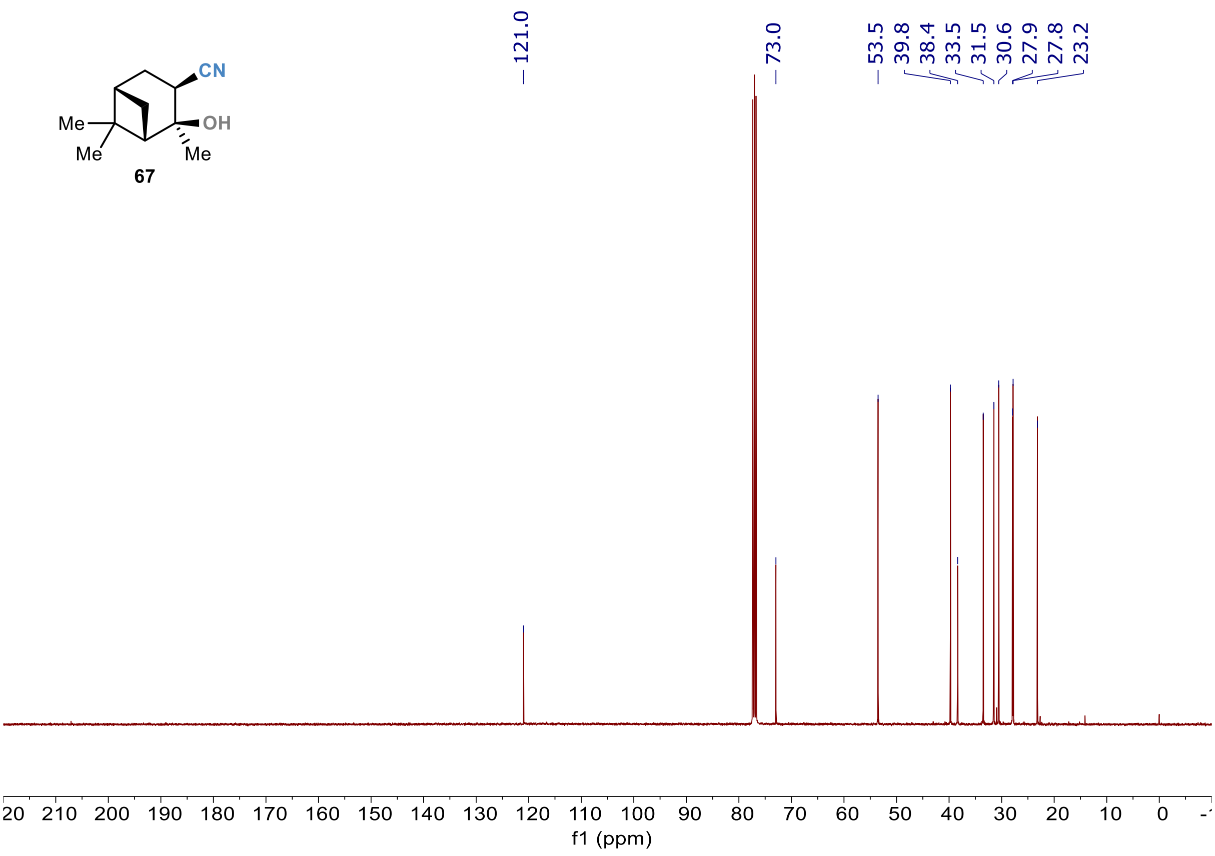

¹H NMR (400 MHz, $CDCl_3$) for compound **68**:

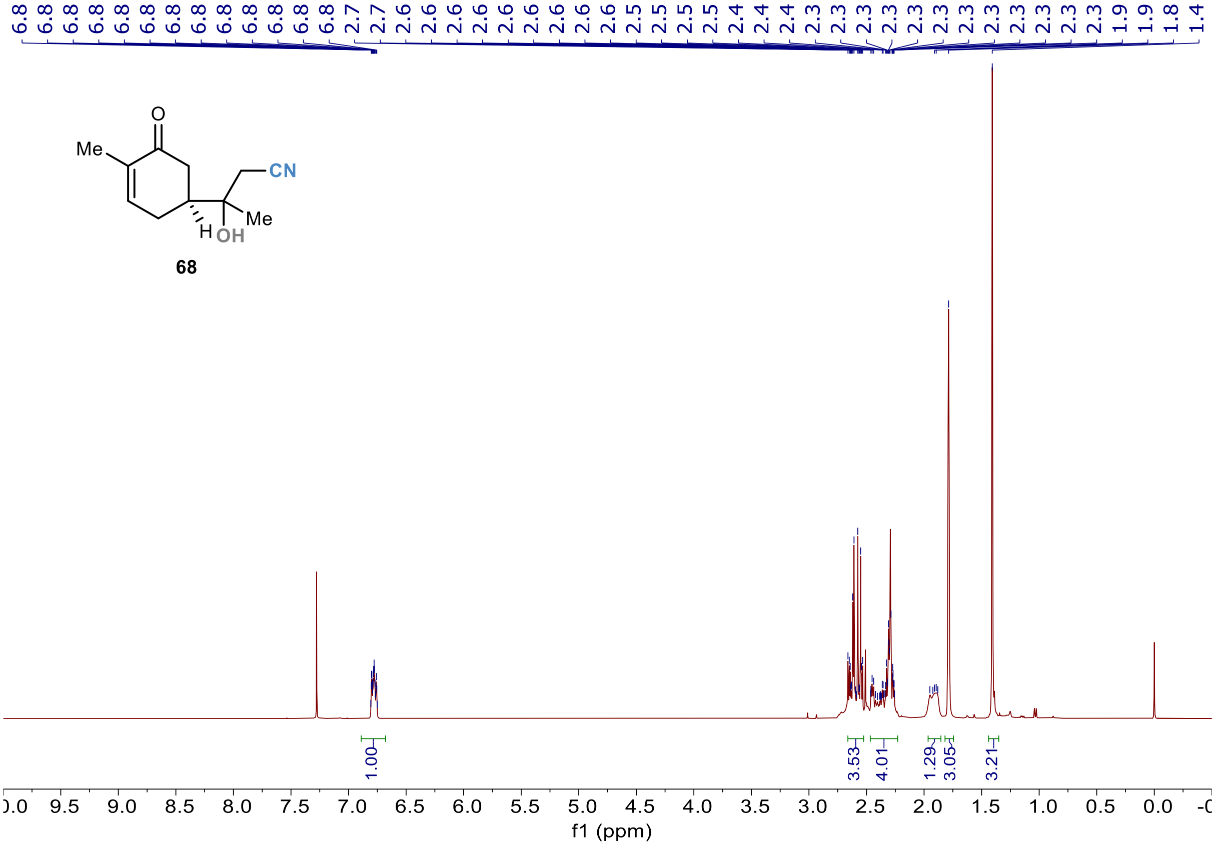

¹³C NMR (101 MHz, $CDCl_3$) for compound **68**:

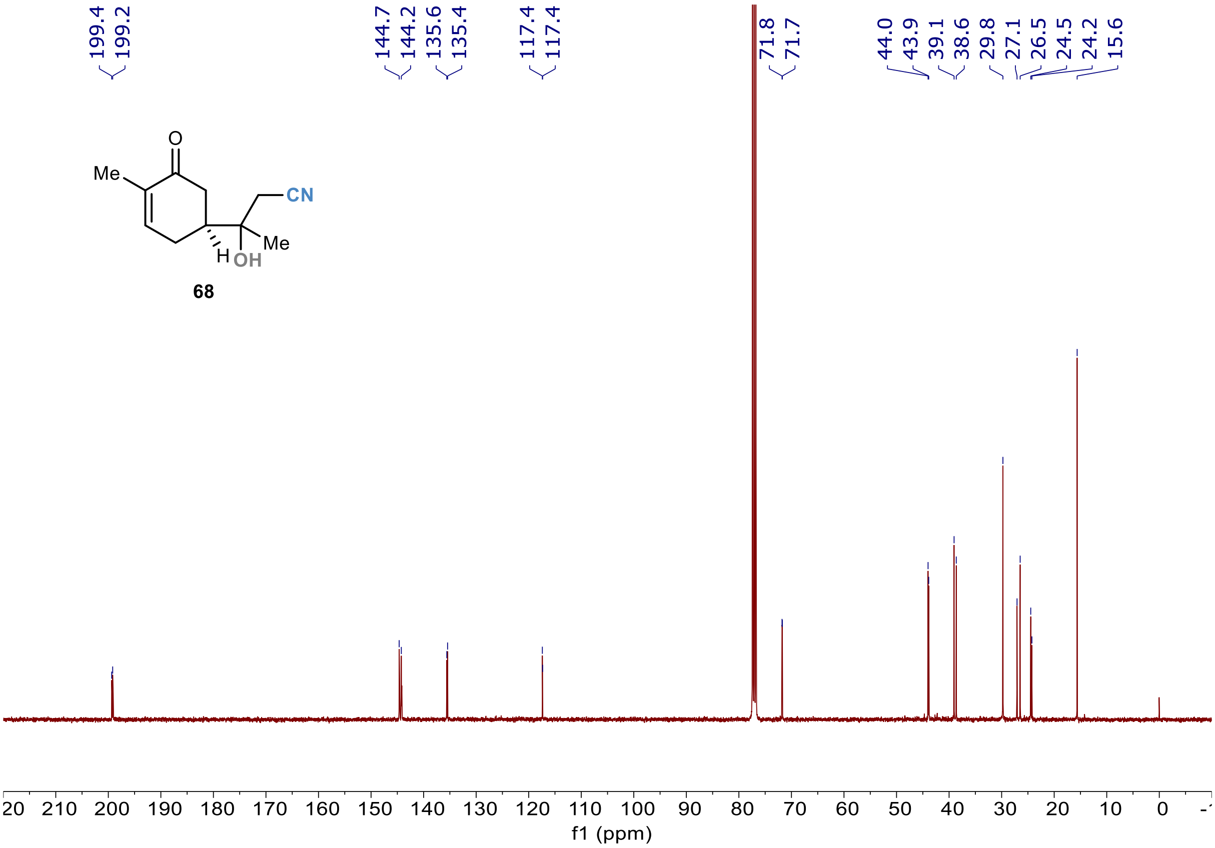

^{1}H NMR (400 MHz, $CDCl_3$) for compound **69**:

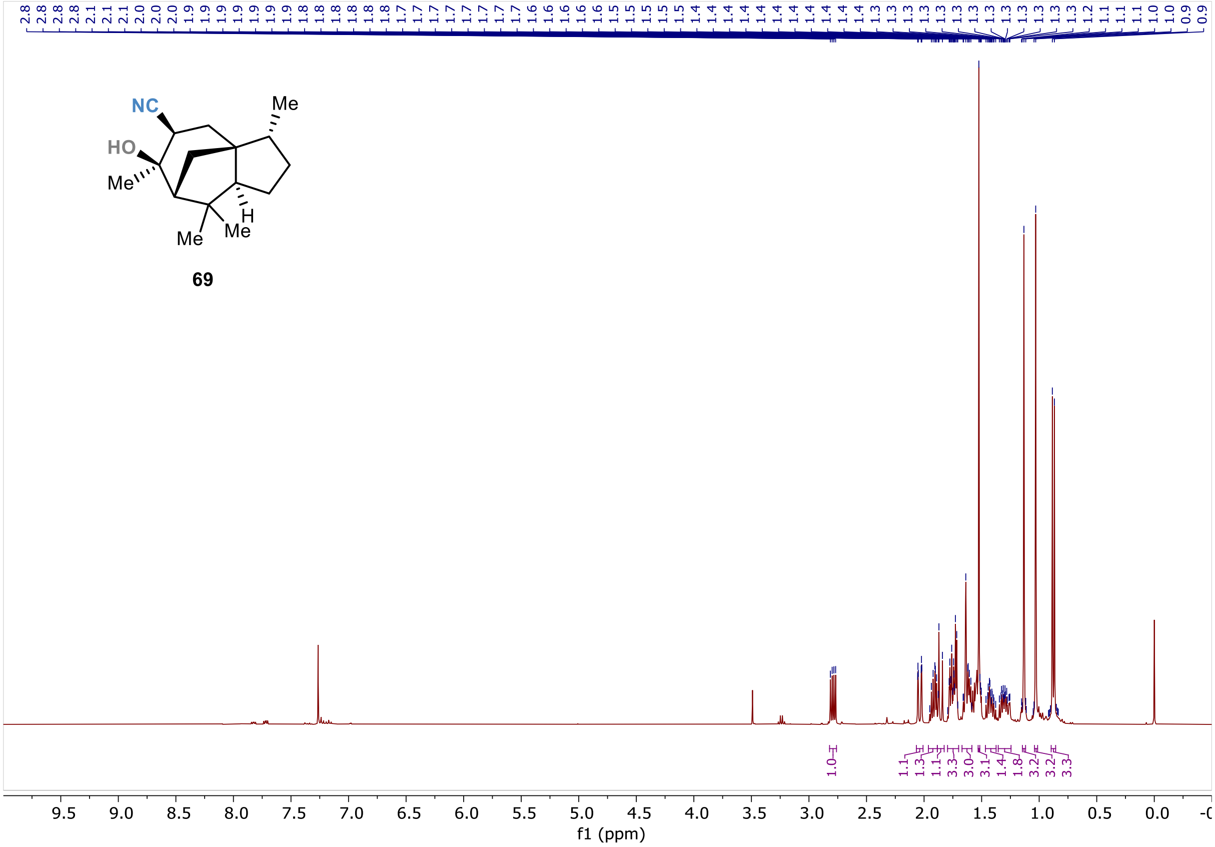

^{13}C NMR (101 MHz, $CDCl_3$) for compound **69**:

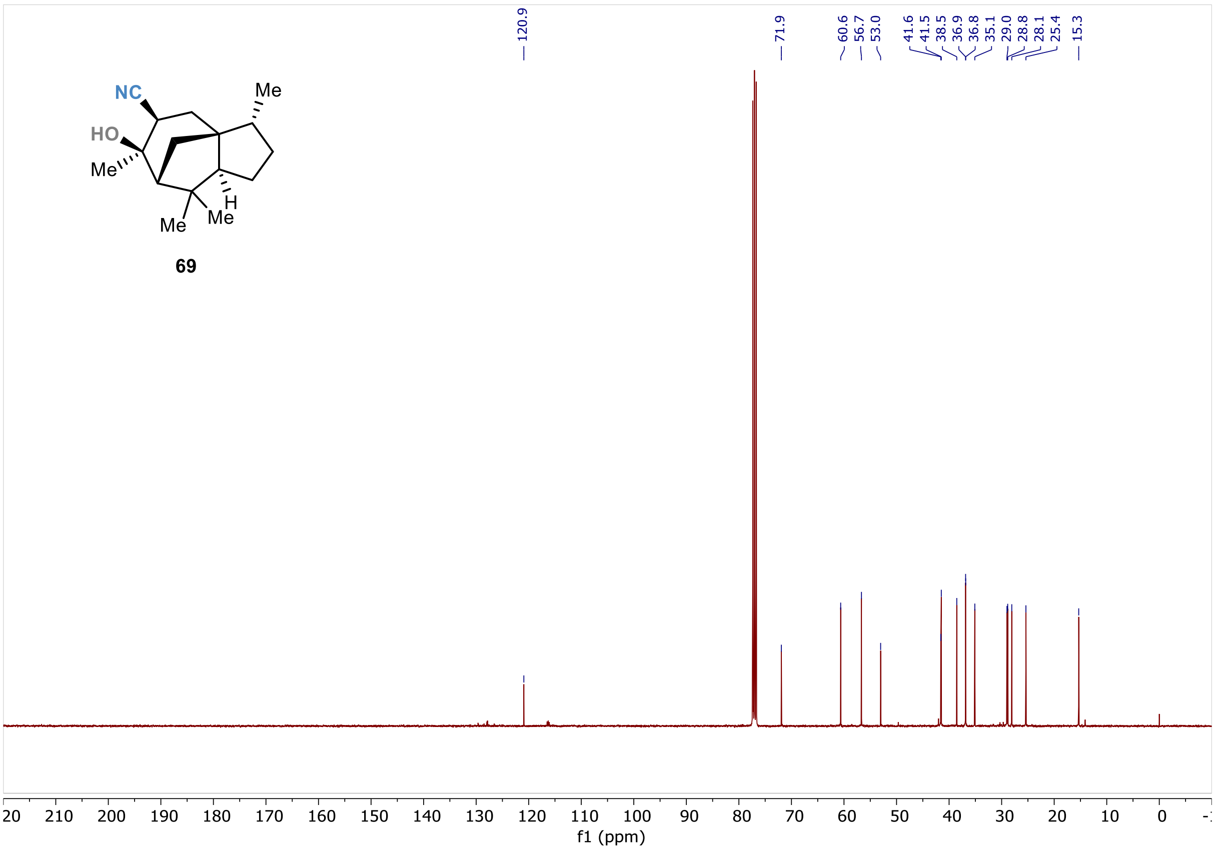

¹H NMR (400 MHz, $CDCl_3$) for compound **70**:

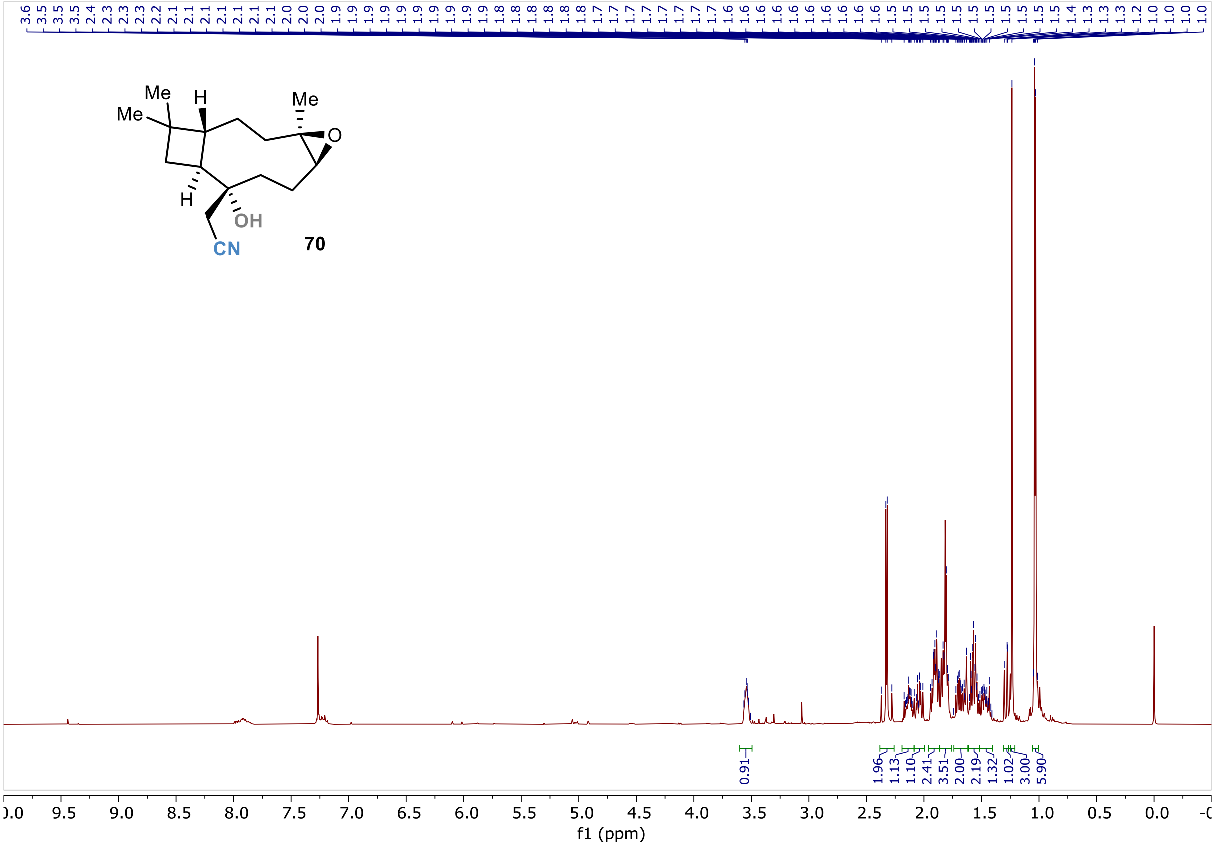

¹³C NMR (101 MHz, $CDCl_3$) for compound **70**:

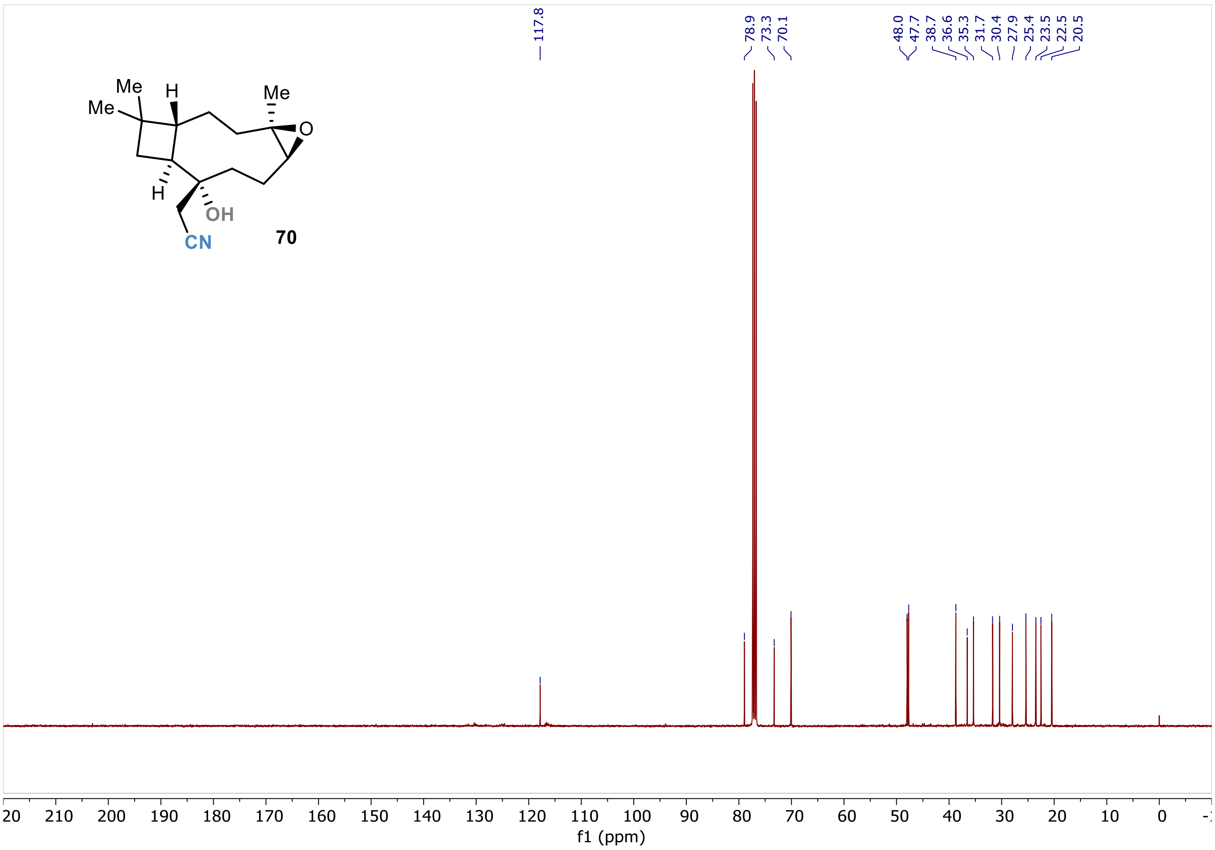

^{1}H NMR (400 MHz, $CDCl_3$) for compound **71**:

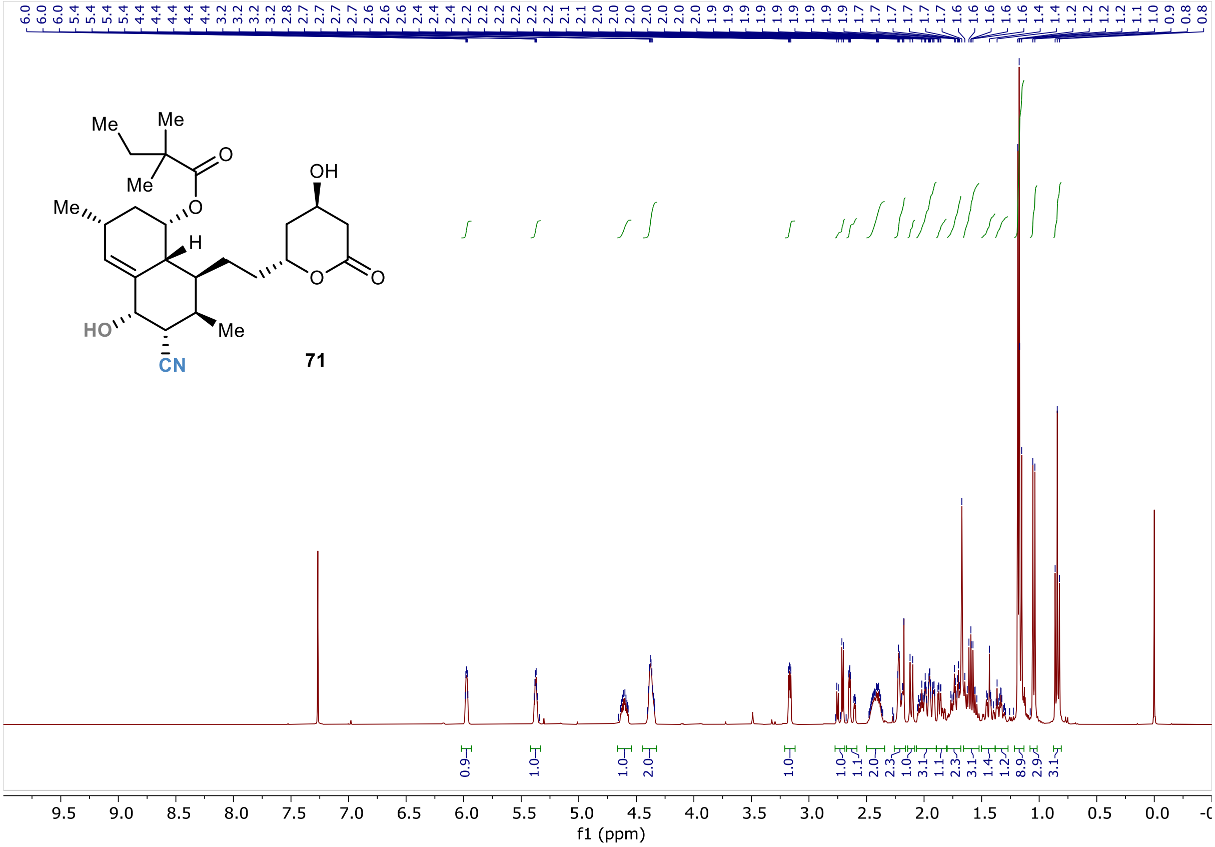

71

^{13}C NMR (101 MHz, $CDCl_3$) for compound **71**:

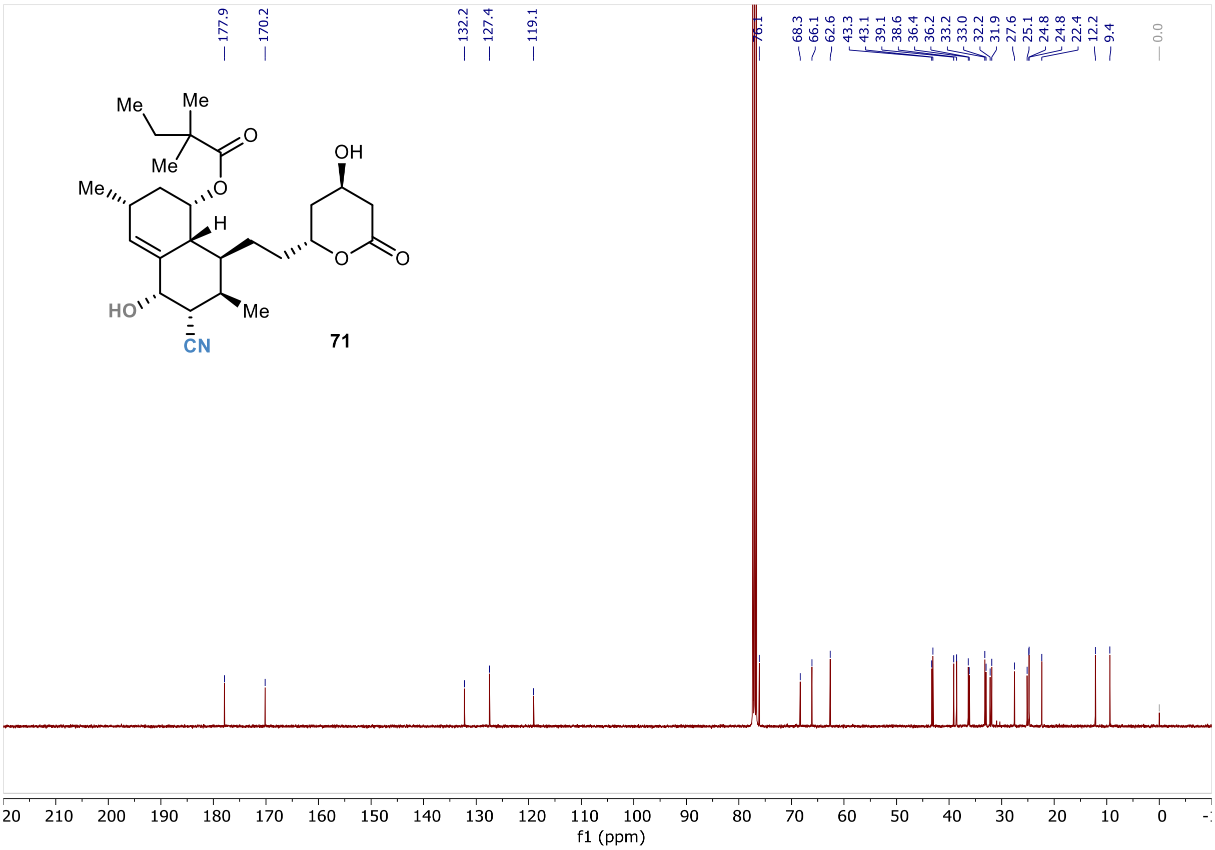

71

^{1}H NMR (400 MHz, $CDCl_3$) for compound **S-6**:

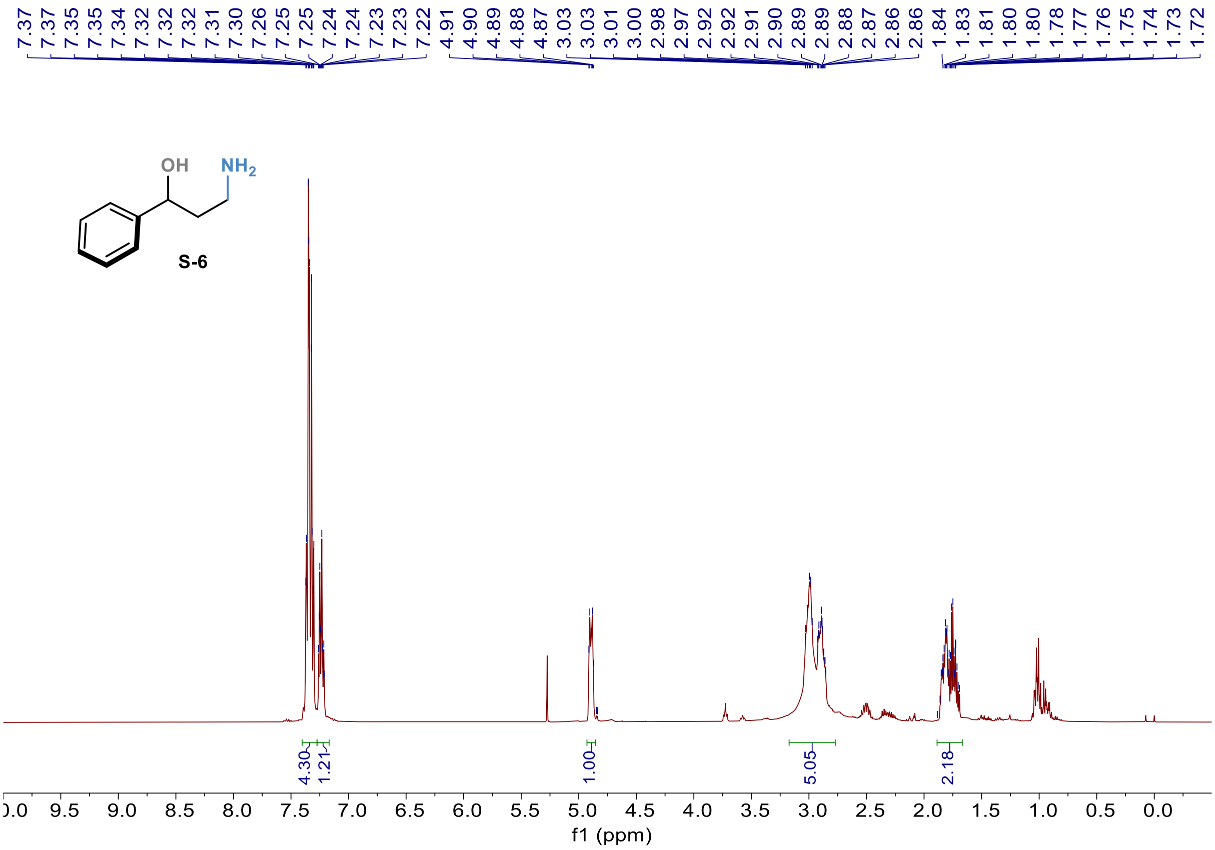

^{13}C NMR (101 MHz, $CDCl_3$) for compound **S-6**:

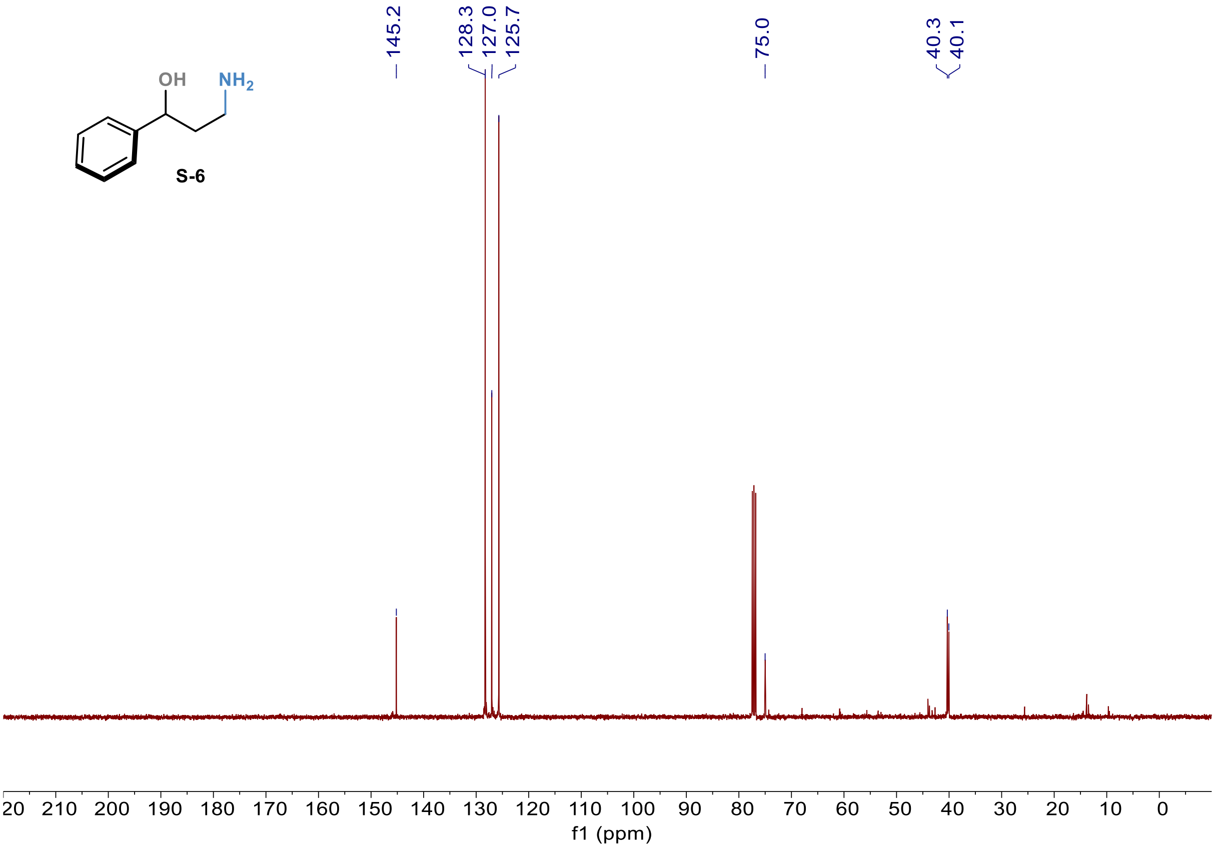

^{1}H NMR (500 MHz, $CDCl_3$) for compound **S-7**:

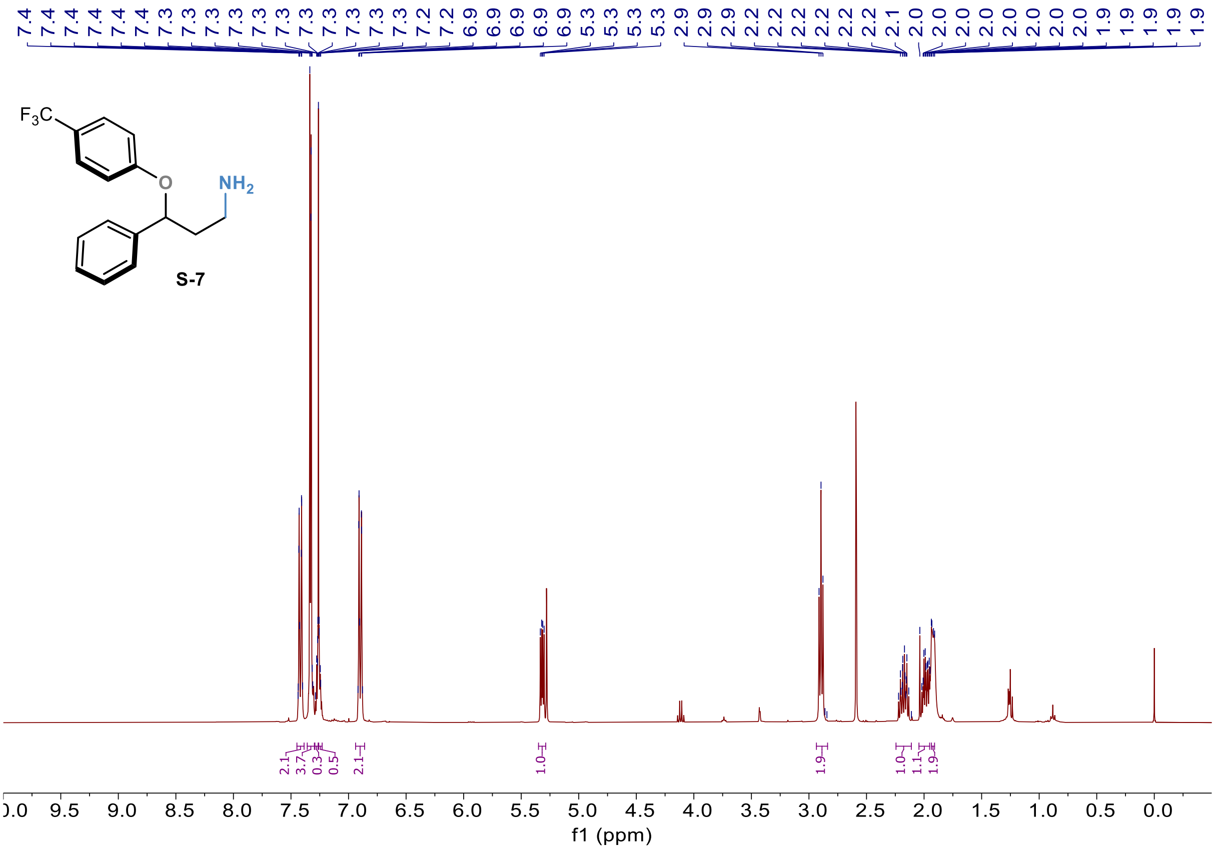

^{13}C NMR (126 MHz, $CDCl_3$) for compound **S-7**:

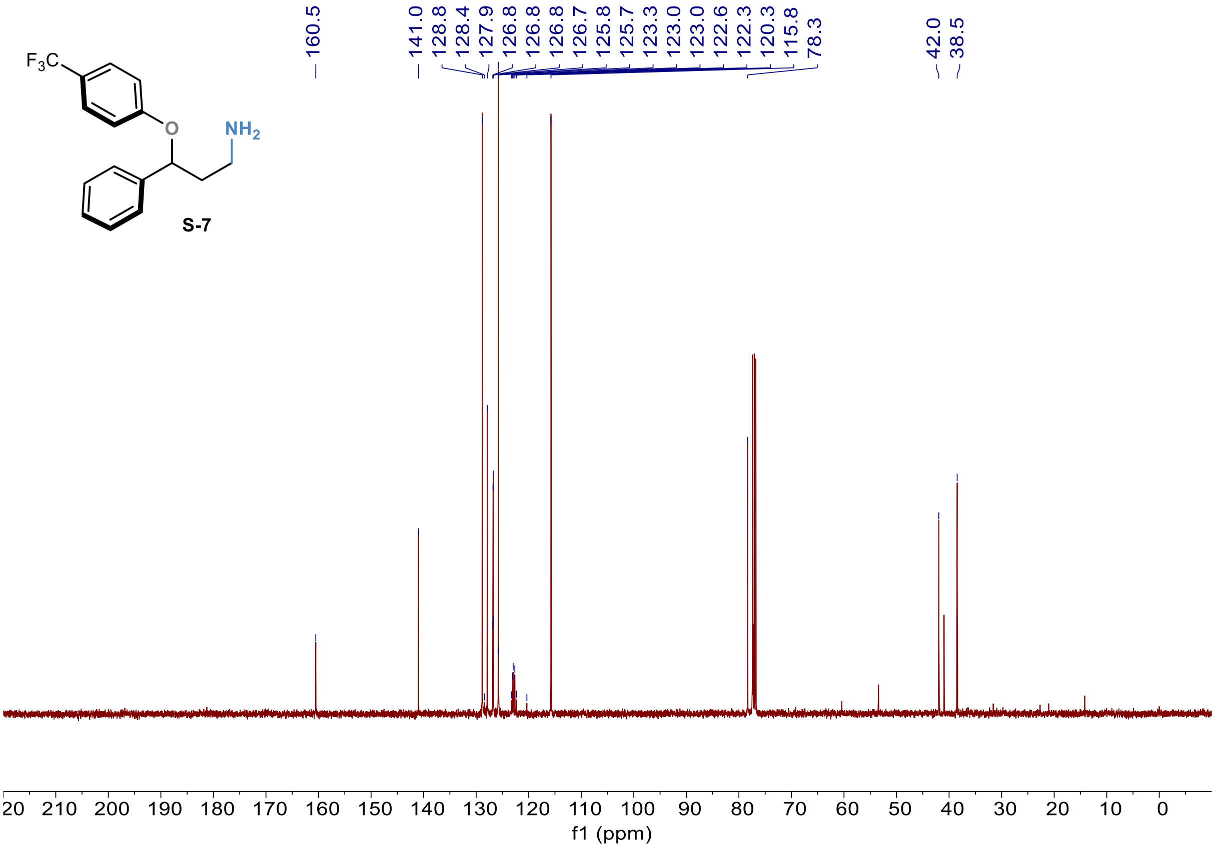

^{19}**F NMR** (376 MHz, $CDCl_3$) for compound **S-7**:

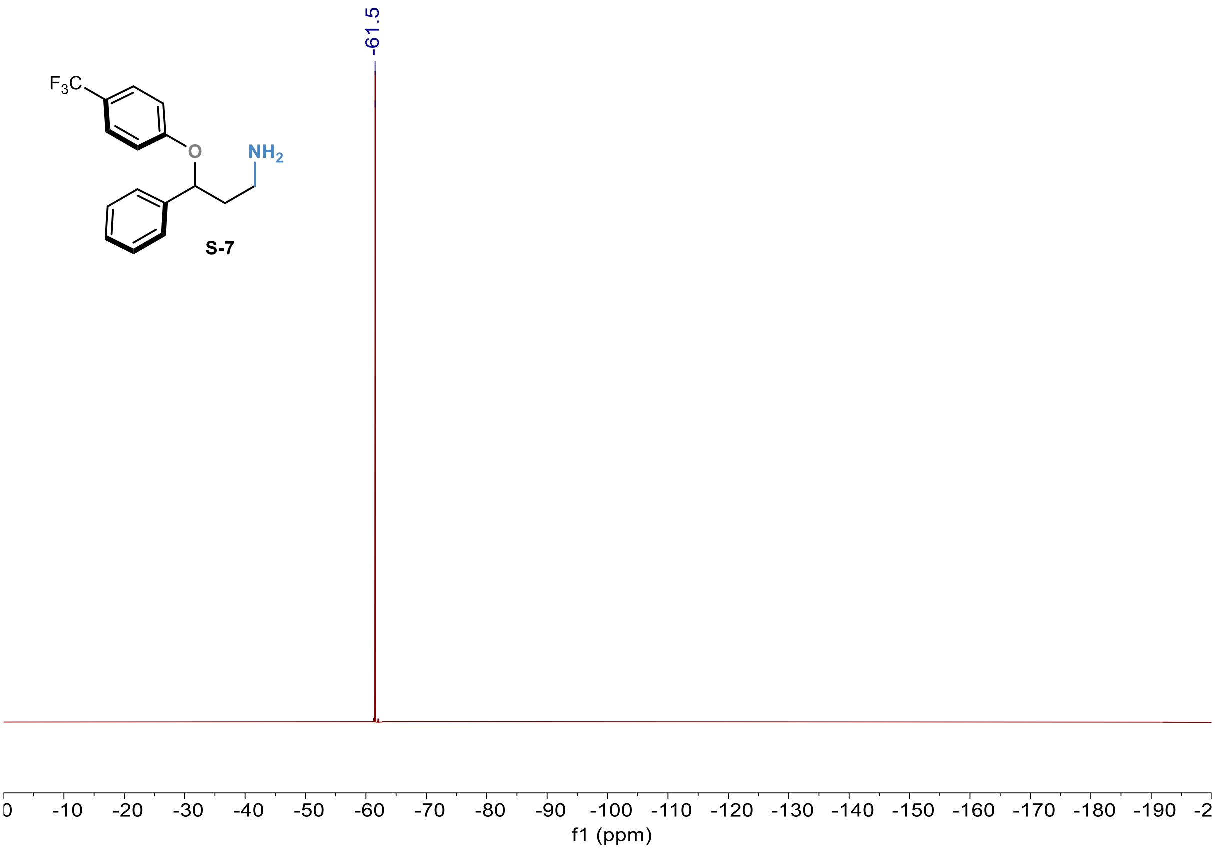

^{1}**H NMR** (400 MHz, $CDCl_3$) for compound **S-8**:

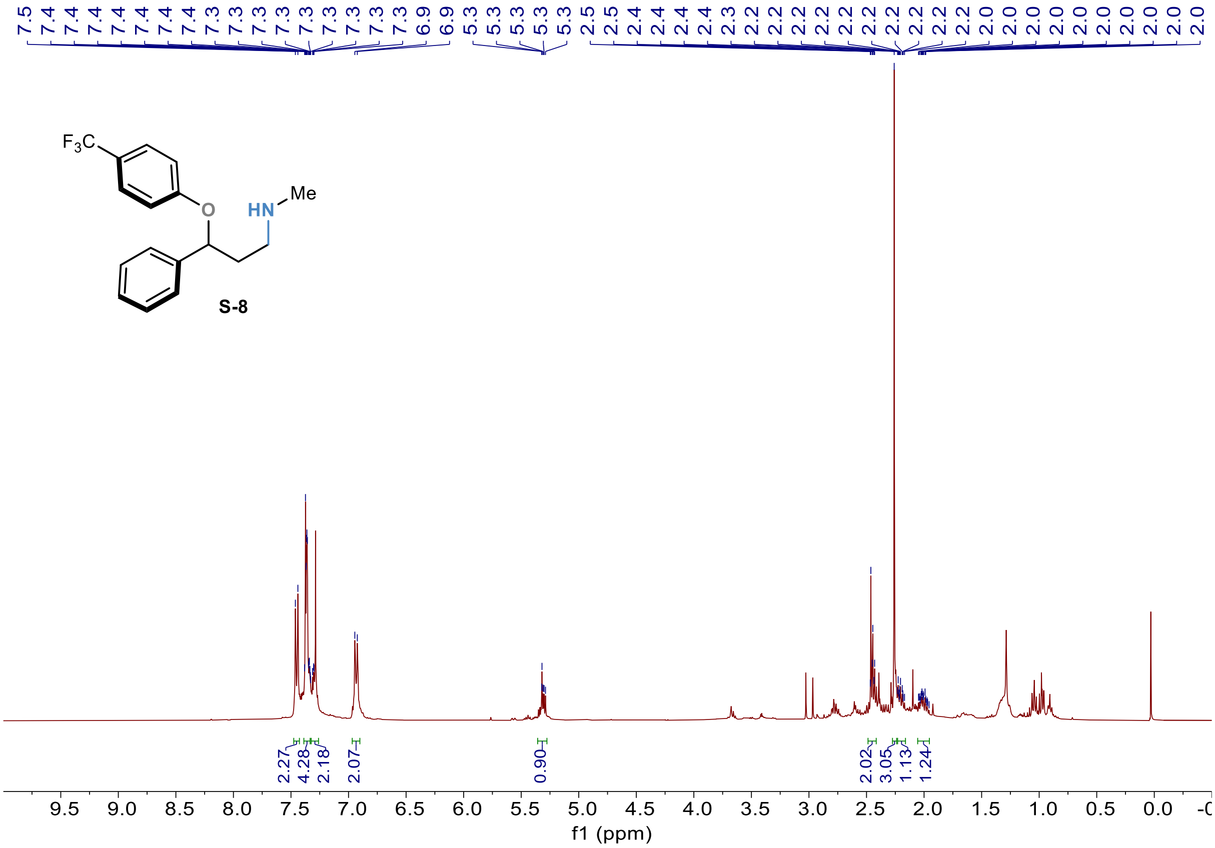

¹³C NMR (126 MHz, $CDCl_3$) for compound **S-8**:

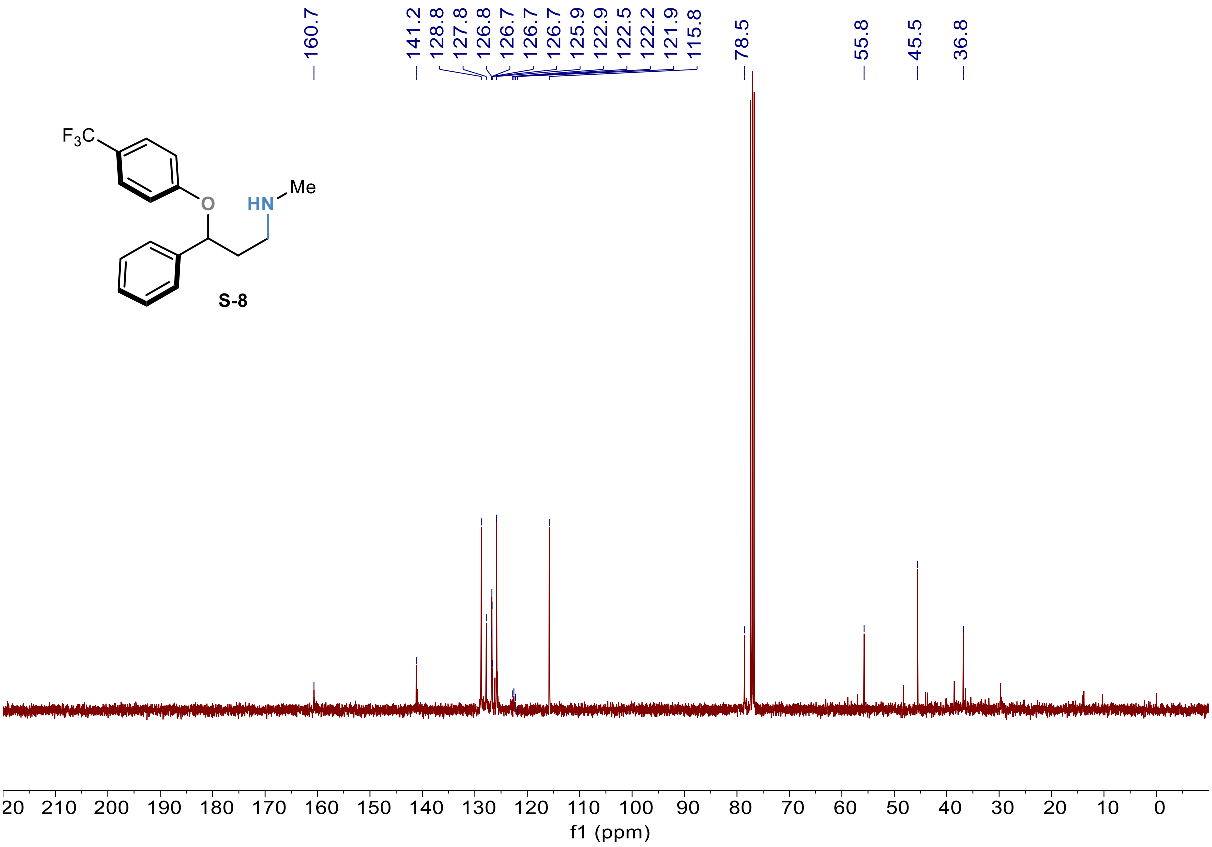

¹⁹F NMR (376 MHz, $CDCl_3$) for compound **S-8**:

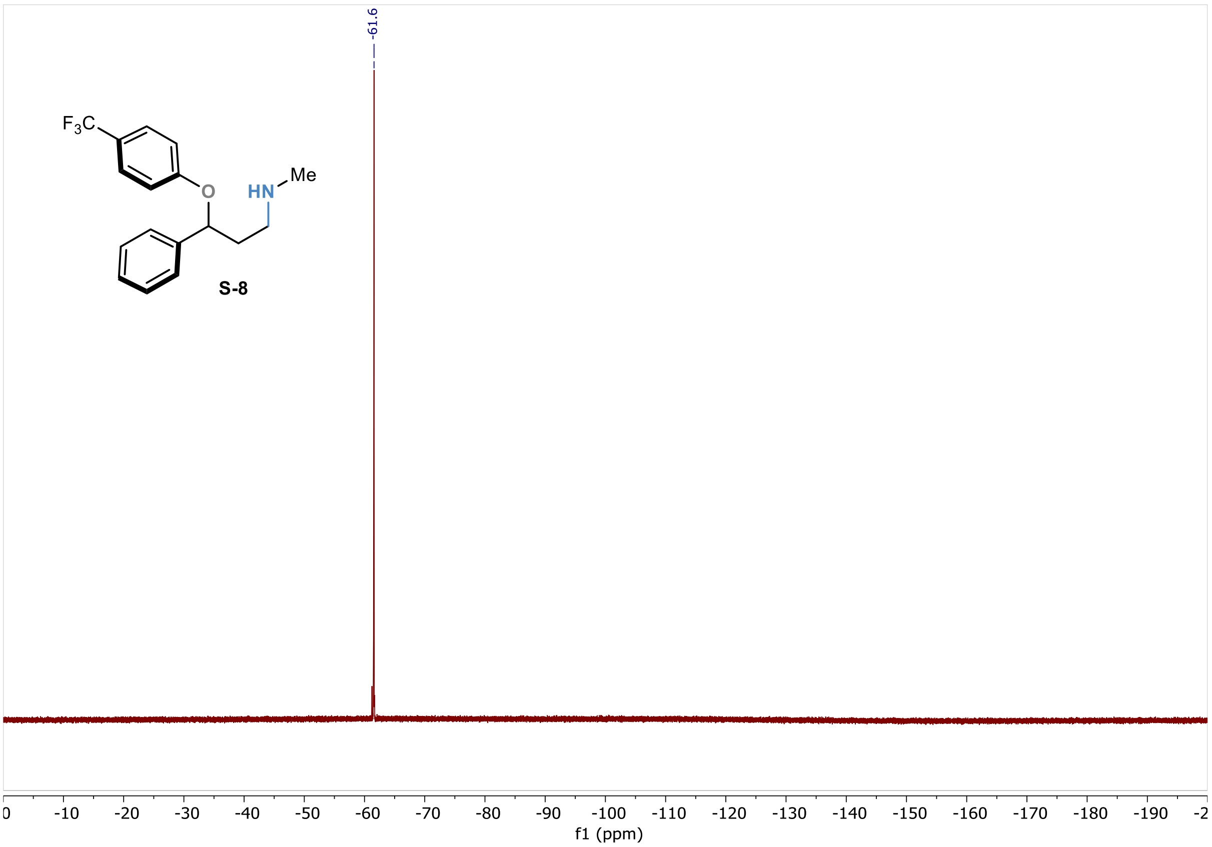

^{1}H NMR (500 MHz, $CDCl_3$) for compound **73**:

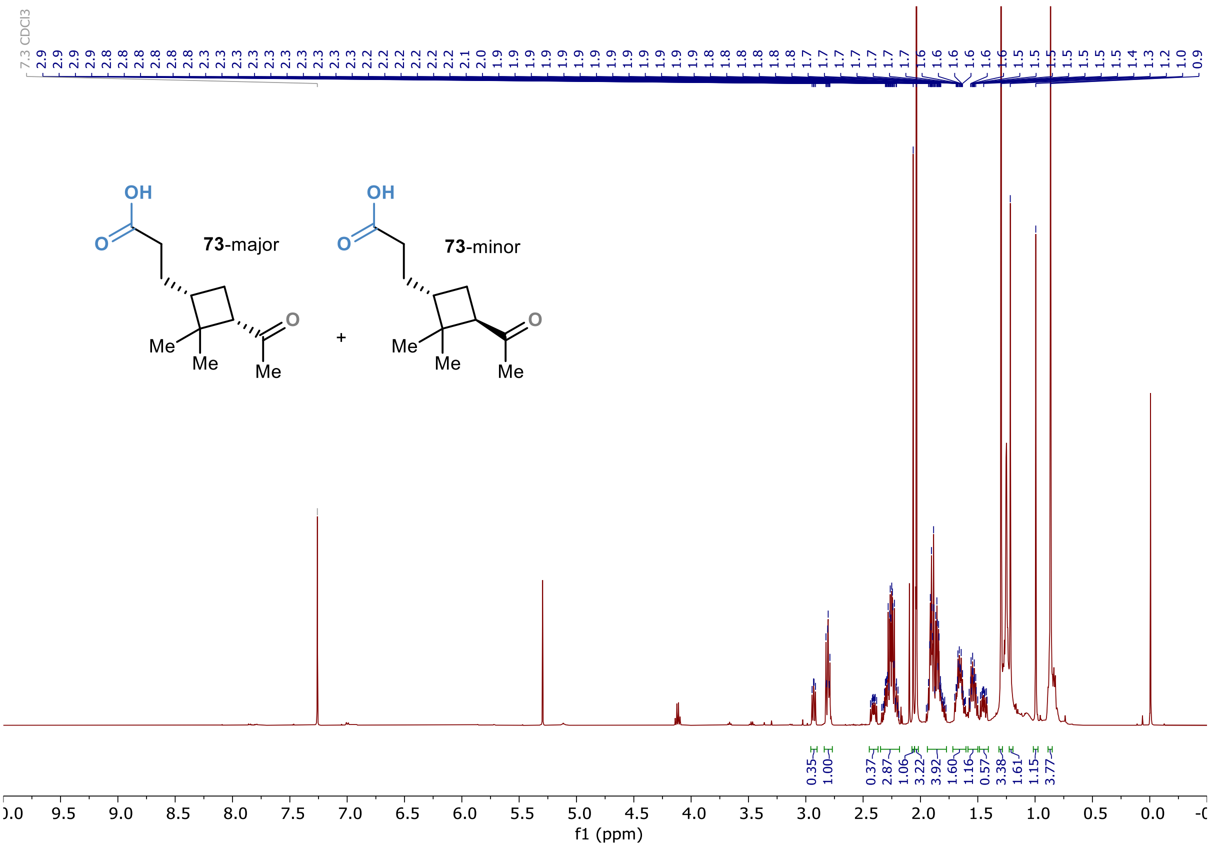

^{13}C NMR (126 MHz, $CDCl_3$) for compound **73**:

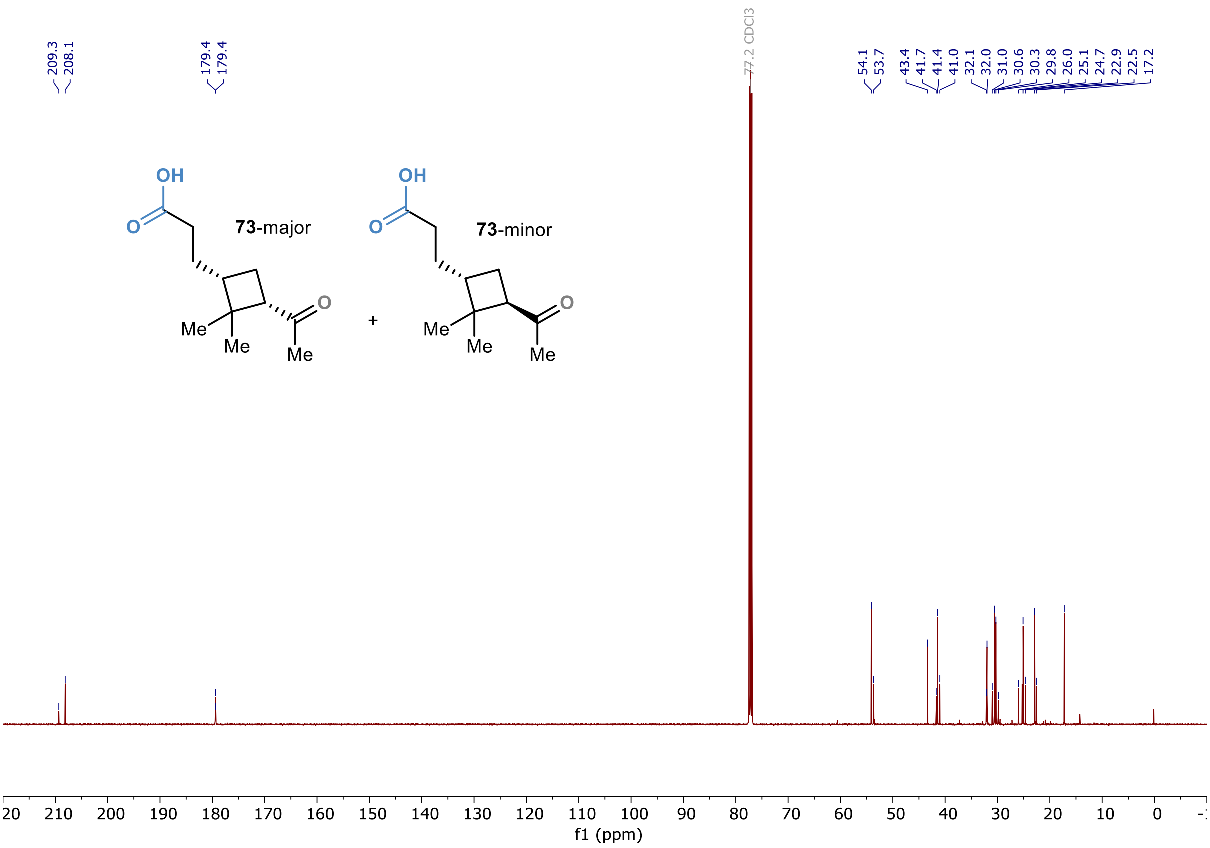

Supplement: Supplementary file 1 — ja4c13682_si_001.pdf [file ja4c13682_si_001.pdf]
